# Factors relating to mortality in septic patients in Vietnamese intensive care units from a subgroup analysis of MOSAICS II study

**DOI:** 10.1038/s41598-021-98165-8

**Published:** 2021-09-23

**Authors:** Son Ngoc Do, Chinh Quoc Luong, Dung Thi Pham, My Ha Nguyen, Nga Thi Nguyen, Dai Quang Huynh, Quoc Trong Ai Hoang, Co Xuan Dao, Trung Minh Le, Ha Nhat Bui, Hung Tan Nguyen, Hai Bui Hoang, Thuy Thi Phuong Le, Lien Thi Bao Nguyen, Phuoc Thien Duong, Tuan Dang Nguyen, Yen Hai Vu, Giang Thi Tra Pham, Tam Van Bui, Thao Thi Ngoc Pham, Hanh Trong Hoang, Cuong Van Bui, Nguyen Minh Nguyen, Giang Thi Huong Bui, Thang Dinh Vu, Nhan Duc Le, Trang Huyen Tran, Thang Quang Nguyen, Vuong Hung Le, Chi Van Nguyen, Bryan Francis McNally, Jason Phua, Anh Dat Nguyen

**Affiliations:** 1 Center for Emergency Medicine, Bach Mai Hospital, 78 Giai Phong road, Phuong Mai ward, Dong Da district, Hanoi, 100000 Vietnam; 2 Department of Emergency and Critical Care Medicine, Hanoi Medical University, Hanoi, 100000 Vietnam; 3 Faculty of Medicine, University of Medicine and Pharmacy, Vietnam National University, Hanoi, 100000 Vietnam; 4 Department of Nutrition and Food Safety, Faculty of Public Health, Thai Binh University of Medicine and Pharmacy, Thai Binh, 410000 Vietnam; 5 Department of Health Organization and Management, Faculty of Public Health, Thai Binh University of Medicine and Pharmacy, Thai Binh, 410000 Vietnam; 6 Department of Intensive Care and Poison Control, Vietnam–Czechoslovakia Friendship Hospital, Hai Phong, 180000 Vietnam; 7 Intensive Care Department, Cho Ray Hospital, Ho Chi Minh City, 700000 Vietnam; 8 Department of Critical Care, Emergency Medicine and Clinical Toxicology, Faculty of Medicine, Ho Chi Minh City University of Medicine and Pharmacy, Ho Chi Minh City, 700000 Vietnam; 9 Emergency Department, Hue Central General Hospital, Hue City, Thua Thien Hue 530000 Vietnam; 10 Department of Intensive Care, Bach Mai Hospital, Hanoi, 100000 Vietnam; 11 Intensive Care Unit, 115 People’s Hospital, Ho Chi Minh City, 700000 Vietnam; 12 Intensive Care Unit, Bai Chay General Hospital, Quang Ninh, 200000 Vietnam; 13 Intensive Care Unit, Da Nang Hospital, Da Nang, 550000 Vietnam; 14 Emergency and Critical Care Department, Hanoi Medical University Hospital, Hanoi Medical University, Hanoi, 100000 Vietnam; 15 Intensive Care Unit, Dong Da General Hospital, Hanoi, 100000 Vietnam; 16 Intensive Care Unit, Saint Paul General Hospital, Hanoi, 100000 Vietnam; 17 Intensive Care Unit, Can Tho Central General Hospital, Can Tho, 900000 Vietnam; 18 Intensive Care Unit, Vinmec Times City International Hospital, Hanoi, 100000 Vietnam; 19 Intensive Care Unit, Thai Nguyen Central General Hospital, Thai Nguyen, 250000 Vietnam; 20 Emergency Department, Thanh Nhan General Hospital, Hanoi, 100000 Vietnam; 21 Intensive Care Unit, Hue Central General Hospital, Hue City, Thua Thien Hue 530000 Vietnam; 22 Department of Emergency and Critical Care Medicine, Faculty of Medicine, Hue University of Medicine and Pharmacy, Hue City, Thua Thien Hue 530000 Vietnam; 23 Emory University Rollins School of Public Health, Atlanta, GA 30322 USA; 24 Department of Emergency Medicine, Emory University School of Medicine, Atlanta, GA 30322 USA; 25 FAST and Chronic Programmes, Alexandra Hospital, National University Health System, Singapore, 159964 Singapore; 26 Division of Respiratory and Critical Care Medicine, Department of Medicine, National University Health System, Singapore, 119228 Singapore

**Keywords:** Infectious diseases, Risk factors, Health policy, Health services, Outcomes research, Urological manifestations, Urinary tract infection, Renal replacement therapy, Acute kidney injury, Urinary tract infection, Antimicrobials, Bacteria, Pathogens, Infection

## Abstract

Sepsis is the most common cause of in-hospital deaths, especially from low-income and lower-middle-income countries (LMICs). This study aimed to investigate the mortality rate and associated factors from sepsis in intensive care units (ICUs) in an LMIC. We did a multicenter cross-sectional study of septic patients presenting to 15 adult ICUs throughout Vietnam on the 4 days representing the different seasons of 2019. Of 252 patients, 40.1% died in hospital and 33.3% died in ICU. ICUs with accredited training programs (odds ratio, OR: 0.309; 95% confidence interval, CI 0.122–0.783) and completion of the 3-h sepsis bundle (OR: 0.294; 95% CI 0.083–1.048) were associated with decreased hospital mortality. ICUs with intensivist-to-patient ratio of 1:6 to 8 (OR: 4.533; 95% CI 1.621–12.677), mechanical ventilation (OR: 3.890; 95% CI 1.445–10.474) and renal replacement therapy (OR: 2.816; 95% CI 1.318–6.016) were associated with increased ICU mortality, in contrast to non-surgical source control (OR: 0.292; 95% CI 0.126–0.678) which was associated with decreased ICU mortality. Improvements are needed in the management of sepsis in Vietnam such as increasing resources in critical care settings, making accredited training programs more available, improving compliance with sepsis bundles of care, and treating underlying illness and shock optimally in septic patients.

## Introduction

Sepsis is defined as life-threatening organ dysfunction caused by a dysregulated host response to infection and is an important global health problem^1,2^. Sepsis is the most common cause of in-hospital deaths and extracts a high economic and social cost^3–5^; mortality rates remain high at 30–45% and contribute to as much as 20% of all deaths worldwide^2,5–7^. Infection prevention efforts, including those targeting both community-acquired and healthcare-associated infections, can reduce sepsis incidence^8,9^. Sepsis is treatable, and timely implementation of targeted interventions improves outcomes^10–12^. However, accurate quantification of sepsis incidence and mortality remains a formidable challenge^13–15^. While sepsis epidemiology, including its prevalence and causes, differs between countries/regions^7,16^, most data are obtained from high-income countries (HICs) which constitute only 13% of the world’s population^13^.

Data on sepsis from the low-income (LICs) and lower-middle-income countries (LMICs) are lacking, with Asia being substantially under-represented. The Management of Severe sepsis in Asia’s Intensive Care unitS (MOSAICS I) study, including Vietnam, conducted in 2009, helped shed some light, but had limited participation by units from LICs and LMICs^17^. Similarly, in the international Extended Study on Prevalence of Infection in Intensive Care (EPIC III) conducted in 2017, fewer than 5% of the study population was from LICs and LMICs^16^. Importantly, the EPIC III study focused on the prevalence of infection rather than sepsis. In Southeast Asia, a multinational multicenter cross-sectional study of community-acquired sepsis and severe sepsis shows that sepsis is caused by a wide range of known and emerging pathogens, and is associated with substantial death rates (mortality rates of 1.8% [14/763] in pediatric and 13.3% [108/815] in adult patients), of which bacteremia was commonly observed in both age groups in the study population^18^.

Vietnam is an LMIC, ranked 15th in the world and 3rd in Southeast Asia by population with 96.462 million people^19^. Vietnam is also a hotspot for emerging infectious diseases in Southeast Asia, including the SARS-CoV^20^, avian influenza A(H5N1)^21,22^, and ongoing global COVID-19 outbreaks^23^. Additionally, severe dengue, *Streptococcus suis* infection and increased antibiotic resistance are other major causes of sepsis in ICUs across Vietnam^24–27^. Despite its recent economic growth spurt^28^, Vietnam is still struggling to provide either enough resources or adequate diagnostic and treatment strategies for patients with sepsis and septic shock in both local and central settings^29,30^. In addition, within the healthcare system in Vietnam, central hospitals are responsible for receiving patients who have difficulties being treated in local hospital settings^31^. Therefore, the initiation of treatment in patients with sepsis is often delayed, including the administration of antibiotics.

Understanding the country-specific etiologies and the disease risk and prognosis of sepsis and septic shock are crucial for reducing mortality in Vietnam, as well as in other countries that face challenges in clinical practice owing to limited medical resources. The aim of this study, therefore, was to investigate the mortality rate and associated factors from sepsis in the country.

## Methods

### Study design and setting

This multicenter observational, cross-sectional, point prevalence study is part of the Management of Severe sepsis in Asia’s Intensive Care unitS II (MOSAICS II) study^32^, which collects data on the management of sepsis in Asia. In this study, we used only data from Vietnam. A total of 15 adult ICUs (excluding predominantly neurosurgical, coronary, and cardiothoracic ICUs) participating in the MOSAICS II study from 14 hospitals, of which five are central and nine are provincial, district, or private hospitals, throughout Vietnam. Each ICU had one or two representatives. Participation was voluntary and unfunded.

### Participants

**Figure 1:**
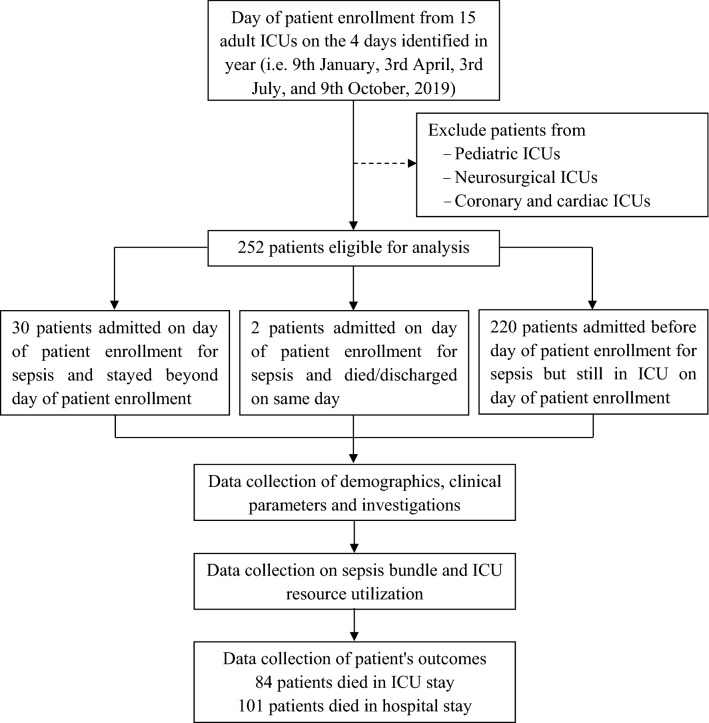
Flowchart of the study design, patient enrollment and follow up. *ICU* intensive care unit.

All patients admitted to participating ICUs on 4 days which represented the different seasons of 2019 (9th January, 3rd April, 3rd July, and 9th October) were screened for eligibility; there was no formal sample size calculation. We included all patients, aged ≥ 18 years old, who were admitted to the ICUs for sepsis, and who were still in the ICUs from 00:00 to 23:59 h of the study days (Fig. 1). We defined sepsis as infection with a Sequential Organ Failure Assessment (SOFA) score ≥ 2 from baseline (assumed to be 0 for patients without prior organ dysfunction)^1^.

### Data collection

We used a standardized classification and case record form to collect data on common variables. The data dictionary of the MOSAICS II study is available in Additional file 1. Data was entered into the database of the MOSAICS II study by the password-protected online case report forms. We checked the data for implausible outliers and missing fields and contacted ICU representatives for clarification. Representatives also completed a questionnaire to describe their centers’ characteristics (e.g. hospital and ICU type, open or closed ICU model, university affiliation status, presence of accredited training program, nurse-to-patient ratio, and intensivist-to-patient ratio among the closed ICUs). We then merged the data sets for the 14 hospitals.

### Variables

Prior to patient enrollment, representatives completed a questionnaire to describe their centers’ characteristics is found in Additional file 2. The case report form contained four sections which is available in Additional file 1. The first section focused on baseline characteristics (demographics, comorbidities, and details of admission). The second section comprised of vital signs upon ICU admission, laboratory parameters, and illness severity scores (e.g., SOFA score, systemic inflammatory response syndrome (SIRS) criteria and the Acute Physiology and Chronic Health Evaluation (APACHE) II score), site of infection, and microbiology. Only microorganisms detected via all cultures, serology, molecular, and histological investigations and deemed to be true pathogens rather than commensals or contaminants were recorded. The third section captured the timing of sepsis bundle elements referencing time zero, determined as follows: (a) time of triage in the emergency department (ED) for those presenting with sepsis to the ED; (b) time of clinical documentation of deterioration in the general wards or other non-ED areas for those who developed sepsis after hospital admission; (c) time of ICU admission for those in which (a) or (b) could not be determined from the clinical documentation. The bundle elements were based on the Surviving Sepsis Campaign’s 2018 update: antibiotics administration, blood cultures, lactate measurement, fluid administration (amount of fluids administered in the first and third hours from time zero) and vasopressor initiation^33^. The fourth section concerned life-sustaining treatments provided during the ICU stay. In addition, each ICU recorded the total number of ICU patients on each study day. We followed all patients till hospital discharge, death in the ICU/hospital, and up to 90 day post-enrollment, whichever was earliest.

### Outcomes

The primary outcome was hospital mortality. We also examined the following secondary outcomes: ICU mortality, and ICU and hospital lengths of stay (LOS).

### Statistical analyses

We used IBM^®^ SPSS^®^ Statistics 22.0 (IBM Corp., Armonk, United States of America) for data analysis. We report data as numbers and percentages for categorical variables and medians and interquartile ranges (IQRs) or means and standard deviations (SDs) for continuous variables. Comparisons were made between survival and death in the hospital for each variable, using the χ^2^ test or Fisher exact test for categorical variables and the Mann–Whitney U test, Kruskal–Wallis test, one-way analysis of variance for continuous variables.

We assessed factors associated with death in the hospital using logistic regression analysis. To reduce the number of predictors and the multicollinearity issue and resolve the overfitting, we used different ways to select variables as follows: first, we started the variable selection with the bivariate analysis of each variable (Table S10 as shown in Additional file 3) that included independent variables of hospital and intensive care unit characteristics, baseline characteristics, clinical and laboratory characteristics, and treatments if the P-value was < 0.05 in the bivariate analysis between survival and death in the hospital, as well as those that are clinically important. These variables included university affiliation, training program in ICU, comorbidities (i.e., cardiovascular disease, chronic neurological disease), the severity of illness (i.e., qSOFA, SOFA, and APACHE II scores), sites of infection (i.e., urinary tract, skin or cutaneous sites), pathogens detection (i.e., no pathogens detected, Gram negative bacteria), completion of the 1- or 3-h sepsis bundle, completion of the initial administration of antibiotics within 1 or 3 h, respiratory support (i.e., mechanical ventilation, high-flow nasal oxygen), and additional ICU support (e.g., renal replacement therapy, non-surgical source control); Second, we used a stepwise backward elimination method to select variables (Table S11 as shown in Additional file 3). Similarly, we used these methods of variable selection and analysis for assessing factors associated with death in the ICU (Tables S19 and S20 as shown in Additional file 3). We present odds ratios (ORs) and 95% confidence intervals (CIs).

For all analyses, significance levels were two-tailed, and we considered P < 0.05 as statistically significant.

### Ethical issues

The Bach Mai Hospital Scientific and Ethics Committees approved this study (approval number: 2919/QD–BM; project code: BM-2017-883-89). We also obtained permission from the heads of institutions and departments of all participating hospitals and their respective institutional review boards wherever available. The study was conducted according to the principles of the Declaration of Helsinki. The Bach Mai Hospital Scientific and Ethics Committees waived written informed consent for this noninterventional study, and public notification of the study was made by public posting. The authors who did the data analysis kept the data sets in password-protected systems and we present anonymized data.

## Results

Data on 252 patients with sepsis were submitted to the database of the MOSAICS II study (Fig. 1), in which there were little missing data (Table S46 as shown in Additional file 3). Of these patients, more than a third of them (39.3%; 99/252) were from university-affiliated hospitals, four-fifths (80.2%; 202/252) were from ICUs with accredited training programs, low rate of patients was from ICUs with the nurse-to-patient ratio of 1 or more:1 (2.8%; 7/252) or the ratio of 1:2 (74.2%; 187/252), only 65.5% (165/252) of patients were from ICUs with the intensivist-to-patient ratio of 1:5 or fewer and 29.8% (75/252) were from ICUs with the intensivist-to-patient ratio of 1:6 to 8. The characteristics of ICUs were compared between patients who survived and patients who died in the hospital, as shown in Table 1. Among 15 ICUs which provided data on the total number of patients during the study dates (Table S41 as shown in Additional file 3), the prevalence of sepsis was 16.2% (245/1515).

**Table 1:** Hospital and intensive care unit characteristics according to hospital survivability of patients with sepsis.

Variable	All cases	Survived	Died	P^a^
**Hospital characteristics**				
Type of hospital, no. (%)	n = 252	n = 151	n = 101	–
Rural	0	0	0	
Urban	252 (100)	151 (100)	101 (100)	
University affiliation, no. (%)	n = 252	n = 151	n = 101	< 0.001
No	153 (60.7)	105 (69.5)	48 (47.5)	
Yes	99 (39.3)	46 (30.5)	53 (52.5)	
**ICU characteristics**				
Nature of ICU, no. (%)	n = 252	n = 151	n = 101	–
Open	0	0	0	
Closed	252 (100)	151 (100)	101 (100)	
Type of ICU, no. (%)	n = 252	n = 151	n = 101	0.589
Medical	110 (43.7)	68 (45.0)	42 (41.6)	
Surgical	0	0	0	
Mixed	142 (56.3)	83 (55.0)	59 (58.4)	
Nurse to patient ratio, no. (%)	n = 252	n = 151	n = 101	0.079
1 or more nurses:1 patient	7 (2.8)	7 (4.6)	0	
1 nurse:2 patients	187 (74.2)	111 (73.5)	76 (75.2)	
1 nurse:3 patients	0	0	0	
1 nurse:4 or more patients	58 (23.0)	33 (21.9)	25 (24.8)	
Intensivist to patient ratio, no. (%)	n = 252	n = 151	n = 101	0.446
1 intensivist:5 or fewer patients	165 (65.5)	96 (63.6)	69 (68.3)	
1 intensivist:6–8 patients	75 (29.8)	49 (32.5)	26 (25.7)	
1 intensivist:9–11 patients	0	0	0	
1 intensivist:12 or more patients	12 (4.8)	6 (4.0)	6 (5.9)	
Training programme in ICU, no. (%)	n = 252	n = 151	n = 101	0.010
No	50 (19.8)	22 (14.6)	28 (27.7)	
Yes	202 (80.2)	129 (85.4)	73 (72.3)	

*ICU* intensive care unit, *no.* number.

^a^Comparison between survived and died patients with sepsis.

In our study cohort, 64.3% (162/252) were men and the median age was 65 years (IQR: 52–76.75) (Table 2). Among the total patients, the median SOFA score was 7 (IQR: 4.75–10) at the time of ICU admission and the median APACHE II score was 18 (IQR: 13–24) over the first 24 h of ICU admission (Table 3). Overall, 29.4% (74/252) of patients had septic shock (Table 3). Table 4 shows that the most common sites of infection included respiratory (56.7%; 143/252), urinary tract (14.7%; 37/252) and abdominal cavity (24.2%; 61/252), and Gram-negative bacteria were isolated in 61.9% (156/252) of patients (with *Klebsiella pneumonia*, *Acinetobacter baumannii*, *Escherichia coli*, and *Proteus* species predominating). Compliance to the 1-h bundle and 3-h bundle was 36.1% (87/241) and 44.8% (108/241), respectively (Table 5). Nearly a third (31.1%; 78/251) of patients had non-surgical source control while only a fifth (10%; 25/251) received surgical source control (Table 6). Mechanical ventilation (MV) was provided for 68.9% (173/251) of patients and renal replacement therapy (RRT) for 40.2% (101/251) (Table 6). The characteristics, severity of illness, sites of infection and microbiology, compliance with sepsis bundle elements, and life-sustaining treatments during ICU stay were compared between patients who survived and patients who died in the hospital, as shown in Tables 2, 3, 4, 5, and 6.

**Table 2:** Baseline characteristics according to hospital survivability of patients with sepsis.

Variable	All cases	Survived	Died	P^a^
Age (year), median (IQR), n = 252	65 (52–76.75)	65 (53–76)	65 (52–78)	0.810
Sex (male), no. (%)	162/252 (64.3)	93/151 (61.6)	69/101 (68.3)	0.275
Collection batch, no. (%)	n = 252	n = 151	n = 101	0.007
Collection 1 (Jan)	80 (31.7)	58 (38.4)	22 (21.8)	
Collection 2 (April)	62 (24.6)	27 (17.9)	35 (34.7)	
Collection 3 (July)	54 (21.4)	32 (21.2)	22 (21.8)	
Collection 4 (Oct)	56 (22.2)	34 (22.5)	22 (21.8)	
Admission type, no. (%)	n = 252	n = 151	n = 101	0.195
Medical	236 (93.7)	138 (91.4)	98 (97.0)	
Elective surgical	2 (0.8)	2 (1.3)	0	
Unscheduled surgical	14 (5.6)	11 (7.3)	3 (3.0)	
Admission source, no. (%)	n = 252	n = 151	n = 101	0.505
Emergency department	138 (54.8)	87 (57.6)	51 (50.5)	
Operating room	4 (1.6)	3 (2.0)	1 (1.0)	
General wards	56 (22.2)	33 (21.9)	23 (22.8)	
Other ICUs or HDU	16 (6.3)	10 (6.6)	6 (5.9)	
Inter-hospital transfer	37 (14.7)	18 (11.9)	19 (18.8)	
Others	1 (0.4)	0	1 (1.0)	
Comorbidities, no. (%)	n = 252	n = 151	n = 101	
Cardiovascular disease	78 (31.0)	41 (27.2)	37 (36.6)	0.111
Chronic lung disease	30 (11.9)	18 (11.9)	12 (1.9)	0.992
Chronic neurological disease	36 (14.3)	28 (18.5)	8 (7.9)	0.018
Chronic kidney disease	23 (9.1)	14 (9.3)	9 (8.9)	0.922
Peptic ulcer disease	9 (3.6)	5 (3.3)	4 (4.0)	> 0.999
Chronic liver disease	27 (10.7)	14 (9.3)	13 (12.9)	0.365
Diabetes mellitus	67 (26.6)	40 (26.5)	27 (26.7)	0.966
HIV infection	0	0	0	–
Connective tissue disease	3 (1.2)	2 (1.3)	1 (1.0)	> 0.999
Immunosuppression	10 (4.0)	7 (4.6)	3 (3.0)	0.744
Haematological malignancies	5 (2.0)	3 (2.0)	2 (2.0)	> 0.999
Solid malignant tumours	12 (4.8)	6 (4.0)	6 (5.9)	0.551

*HDU* high dependency unit, *ICU* intensive care unit, *IQR* interquartile range, *no.* number.

^a^Comparison between survived and died patients with sepsis.

**Table 3:** Clinical and laboratory characteristics and severity of illness according to hospital survivability of patients with sepsis.

Variable	All cases n = 252	Survived n = 151	Died n = 101	P^a^
**Vital signs (on admission into ICU)**				
GCS, median (IQR), n = 251	13 (9–15)	14 (10–15)	10 (8–14)	< 0.001
HR (beats per min), median (IQR)	110 (95.25–125.75)	110 (92–125)	110 (100–129.5)	0.083
Temperature (^o^C), mean (SD)	37.79 (1.01)	37.80 (1.08)	37.77 (0.91)	0.871
MBP (mmHg), mean(SD)	75.82 (22.08)	79.75 (22.88)	69.93 (19.51)	0.002
SBP (mmHg), mean (SD)	106.45 (29.96)	111.39 (29.44)	99.07 (29.35)	0.004
RR (breaths per min), median (IQR)	25 (22–30)	25 (22–30)	25 (20–30)	0.693
**Blood investigations**				
Total WBC (× 10^9^/L), mean (SD)	15.73 (9.20)	15.63 (8.67)	15.88 (9.98)	0.914
PLT (× 10^9^/L), mean (SD)	185.98 (137.85)	200.71 (129.67)	163.95 (147.15)	0.002
Hb (g/dL), mean (SD), n = 251	11.14 (2.59)	11.36 (2.68)	10.82 (2.44)	0.088
Hct (%), mean (SD)	34.31 (7.75)	35.08 (7.92)	33.17 (7.38)	0.031
K^+^ (mmol/L), mean (SD)	3.89 (0.79)	3.90 (0.80)	3.87 (0.77)	0.865
Na^+^ (mmol/L), mean (SD)	136.05 (8.24)	135.62 (8.81)	136.69 (7.80)	0.068
Creatinine (µmol/L), mean (SD)	187.85 (151.92)	186.15 (171.60)	190.38 (117.27)	0.030
Bilirubin (µmol/L), mean (SD), n = 232	32.80 (61.49)	31.74 (72.67)	34.35 (40.09)	0.007
pH, mean (SD), n = 249	7.37 (0.50)	7.41 (0.64)	7.32 (0.14)	0.004
PaO_2_ (mmHg), mean (SD), n = 244	116.17 (74.28)	110.23 (56.25)	124.73 (94.07)	0.665
FiO_2_, mean (SD), n = 245	0.50 (0.22)	0.44 (0.18)	0.58 (0.24)	< 0.001
PaO_2_/FiO_2_ ratio, mean (SD), n = 243	262.48 (149.58)	281.52 (149.39)	235.26 (146.32)	0.003
**Severity of illness scores**				
SOFA, median (IQR), n = 250	7 (4.75–10)	6 (4–9)	9 (6–12)	< 0.001
APACHE II, median (IQR)	18 (13–24)	15 (12–21)	22 (16–27)	< 0.001
Septic Shock	74 (29.4)	35 (23.2)	39 (38.6)	0.008

*APACHE II* acute physiologic assessment and chronic health evaluation II, *FiO*_*2*_ fraction of inspired oxygen, *GCS* Glasgow coma scale, *Hb* hemoglobin, *Hct* hematocrit, *HDU* high dependency unit, *ICU* intensive care unit, *IQR* interquartile range, *MBP* mean blood pressure, *no.* number, *PaO*_*2*_ partial pressure of oxygen, *PLT* platelet, *qSOFA* quick sequential organ failure assessment, *RR* respiratory rate, *SBP* systolic blood pressure, *SD* standard deviation, *SIRS* systemic inflammatory response syndrome, *SOFA* sequential organ failure assessment, *WBC* white blood cell.

^a^Comparison between survived and died patients with sepsis.

**Table 4:** Sites of infection and microbiology according to hospital survivability of patients with sepsis.

Variable	All cases n = 252	Survived n = 151	Died n = 101	P^a^
**Site of infection**				
Respiratory, no. (%)	143 (56.7)	82 (54.3)	61 (60.4)	0.339
Urinary tract, no. (%)	37 (14.7)	30 (19.9)	7 (6.9)	0.004
Abdominal, no. (%)	61 (24.2)	34 (22.5)	27 (26.7)	0.444
Neurological, no. (%)	12 (4.8)	8 (5.3)	4 (4.0)	0.767
Bones or joints, no. (%)	2 (0.8)	2 (1.3)	0	0.518
Skin or cutaneous sites, no. (%)	19 (7.5)	7 (4.6)	12 (11.9)	0.033
Intravascular catheter, no. (%)	1 (0.4)	1 (0.7)	0	> 0.999
Infective endocarditis, no. (%)	1 (0.4)	0	1 (1.0)	0.401
Primary bacteraemia, no. (%)	7 (2.8)	5 (3.3)	2 (2.0)	0.705
Systemic, no. (%)	6 (2.4)	4 (2.6)	2 (2.0)	> 0.999
Others, no. (%)	–	–	–	–
**Microbiology**				
No pathogens detected, no. (%)	67 (26.6)	47 (31.1)	20 (19.8)	0.046
Gram negative bacteria, no. (%)	156 (61.9)	88 (58.3)	68 (67.3)	0.147
* Klebsiella pneumonia*	27 (10.7)	16 (10.6)	11 (10.9)	0.941
* Acinetobacter baumannii*	45 (17.9)	21 (13.9)	24 (23.8)	0.045
* Escherichia coli*	44 (17.5)	26 (17.2)	18 (17.8)	0.902
* Pseudomonas aeruginosa*	24 (9.5)	17 (11.3)	7 (6.9)	0.251
* Stenotrophomonas maltophilia*	2 (0.8)	0	2 (2.0)	0.160
* Proteus* species	47 (18.7)	25 (16.6)	22 (21.8)	0.297
* Enterobacter cloacae*	3 (1.2)	3 (2.0)	0	0.277
* Bulkholderia pseudomallei*	1 (0.4)	0	1 (1.0)	0.221
Gram positive bacteria, no. (%)	34 (13.5)	22 (14.6)	12 (11.9)	0.540
* Enterococcus*	5 (2.0)	5 (3.3)	0	0.085
MSSA	5 (2.0)	3 (2.0)	2 (2.0)	> 0.999
MRSA	10 (4.0)	6 (4.0)	4 (4.0)	> 0.999
Other *Streptococcus* species	12 (4.8)	6 (4.0)	6 (5.9)	0.551
* Streptococcus pneumonia*	2 (0.8)	2 (1.3)	0	0.518
Fungi, no. (%)	7 (2.8)	4 (2.6)	3 (3.0)	> 0.999
* Candida* species	7 (2.8)	4 (2.6)	3 (3.0)	> 0.999
Viruses, no. (%)	2 (0.8)	0	2 (2.0)	0.160
Influenza	1 (0.4)	0	1 (1.0)	0.401
Dengue	1 (0.4)	0	1 (1.0)	0.401
Other pathogens, no. (%)				
* Mycobacterium tuberculosis*	4 (1.6)	3 (2.0)	1 (1.0)	0.651

*MRSA* methicillin-resistant *Staphylococcus aureus*, *MSSA* methicillin-susceptible *Staphylococcus aureus*, *no.* number.

^a^Comparison between survived and died patients with sepsis.

**Table 5:** Completion of the sepsis bundle of care and the administration of antibiotics according to the hospital survivability of patients with sepsis and septic shock.

Variable	All cases	Survived	Died	P^a^
**Patients with sepsis**	n = 241	n = 146	n = 95	
Completion of the 1-h sepsis bundle of care, no. (%)	87 (36.1)	53 (36.3)	34 (35.8)	0.936
Completion of the administration of antibiotics within 1 h, no. (%)	173 (71.8)	109 (74.7)	64 (63.4)	0.219
Completion of the 3-h sepsis bundle of care, no. (%)	108 (44.8)	66 (45.2)	42 (44.2)	0.879
Completion of the administration of antibiotics within 3 h, no. (%)	205 (85.1)	131 (89.7)	74 (77.9)	0.012
**Patients with septic shock**	n = 72	n = 35	n = 37	
Completion of the 1-h sepsis bundle of care, no. (%)	20 (27.8)	8 (22.9)	12 (32.4)	0.365
Completion of the administration of antibiotics within 1 h, no. (%)	51 (70.8)	23 (65.7)	28 (75.7)	0.353
Completion of the 3-h sepsis bundle of care, no. (%)	27 (37.5)	11 (31.4)	16 (43.2)	0.301
Completion of the administration of antibiotics within 3 h, no. (%)	63 (87.5)	29 (82.9)	34 (91.9)	0.247

*no.* number.

^a^Comparison between survived and died patients with sepsis.

**Table 6:** Life-sustaining treatments during ICU stay and outcomes according to hospital survivability of patients with sepsis.

Variable	All cases	Survived	Died	P
**Life-sustaining treatments during ICU stay**				
Respiratory support				
Mechanical ventilation, no. (%)	173/251 (68.9)	82/150 (54.7)	91/101 (90.1)	< 0.001
Duration of mechanical ventilation, median (IQR), days, n = 251	8 (4–15)	9 (4–15)	7(3–14)	0.153
Non-invasive ventilation, no. (%)	20/251 (8.0)	13/150 (8.7)	7/101 (6.9)	0.618
Duration of non-invasive ventilation, median (IQR), days, n = 251	2 (2–3.75)	2 (1–2)	5 (2–7)	0.004
High-flow nasal oxygen, no. (%)	38/251 (15.1)	29/150 (19.3)	9/101 (8.9)	0.024
Duration of high-flow nasal oxygen, median (IQR), days, n = 251	2 (1–3)	2 (1–3)	2 (1–3)	> 0.999
Additional ICU support				
Vasopressors/inotropes, no. (%)	163/250 (64.7)	82/151 (54.3)	81/101 (80.2)	< 0.001
Renal replacement therapy, no. (%)	101/251 (40.2)	43/150 (28.7)	58/101 (57.4)	< 0.001
Red blood cell transfusion, no. (%)	93/251 (37.1)	48/150 (32.0)	45/101 (44.6)	0.043
Platelet transfusion, no. (%)	50/251 (19.9)	20/150 (13.3)	30/101 (29.7)	0.001
Fresh frozen plasma transfusion, no. (%)	58/251 (23.1)	28/150 (18.7)	30/101 (29.7)	0.042
Surgical source control, no. (%)	25/251 (10.0)	19/150 (12.7)	6/101 (5.9)	0.081
Non-surgical source control, no. (%)	78/251 (31.1)	54/150 (36.0)	24/101 (23.8)	0.040
**Outcomes**				
Length of stay (day), median (IQR)				
Hospital, n = 251	16 (10–25)	17 (11–24.25)	13 (7–26)	0.027
ICU, n = 251	10 (6–18)	10.5 (6–17)	10 (5–21)	0.740

*ICU* intensive care unit, *IQR* interquartile range, *no.* number.

^a^Comparison between survived and died patients with sepsis.

Overall, 40.1% (101/252) of patients with sepsis died during the hospital stay, 33.3% (84/252) of whom died in the ICU (Fig. 1). The median ICU and hospital LOS were 10 (IQR: 6–18) and 16 (IQR: 10–25) days, respectively (Table 6). Among patients with septic shock, hospital and ICU mortality rates were 52.7% (39/74) and 41.9% (31/74), respectively (Tables S21 and S32 as shown in Additional file 3).

Several factors were independently associated with death during the hospital stay in patients with sepsis, including ICUs with accredited training programs (OR: 0.309; 95% CI 0.122–0.783); SOFA score of 12 or higher (OR: 7.381; CI 2.050–26.577); completion of the 3-h sepsis bundle of care (OR: 0.294; 95% CI 0.083–1.048) and the initial administration of antibiotics within 3 h (OR: 0.294; 95% CI 0.083–1.048); and MV (OR: 7.861; 95% CI 3.116–19.830). There were also several factors independently associated with death during the ICU stay, including ICUs with accredited training programs (OR: 0.274; 95% CI 0.111–0.672); ICUs with the intensivist-to-patient ratio of 1:6 to 8 (OR: 4.533; 95% CI 1.621–12.677); MV (OR: 3.890; 95% CI 1.445–10.474); RRT (OR: 2.816; 95% CI 1.318–6.016); and non-surgical source control (OR: 0.292; 95% CI 0.126–0.678). Factors were independently associated with mortality in patients with sepsis during the hospital and ICU stay, as shown in Table 7 and Table S20 (Additional file 3).

**Table 7:** Factors associated with mortality in patients with sepsis: multivariate logistic regression analyses.

Factor	Unit	OR	95.0% CI for OR		P
			Lower	Upper	
**Factors associated with hospital mortality in patients with sepsis**					
Training programme in ICU	%	0.309	0.122	0.783	0.013
Comorbidities					
Cardiovascular disease	%	2.293	1.039	5.060	0.040
Chronic neurological disease	%	0.196	0.060	0.636	0.007
SOFA score					
0–3	%	–	–	–	< 0.001
4–7	%	0.633	0.224	1.794	0.390
8–9	%	2.461	0.742	8.167	0.141
10–11	%	1.520	0.475	4.860	0.480
≥ 12	%	7.381	2.050	26.577	0.002
Site of infection					
Urinary tract	%	0.294	0.083	1.048	0.059
Completion of the 3-h sepsis bundle of care	%	0.294	0.083	1.048	0.017
Completion of the administration of antibiotics within 3 h	%	0.294	0.083	1.048	< 0.001
Respiratory support					
Mechanical ventilation	%	7.861	3.116	19.830	< 0.001
Additional ICU support					
Surgical source control	%	0.331	0.102	1.073	0.065
Non-surgical source control	%	0.488	0.233	1.023	0.057
Constant		1.279			0.750
**Factors associated with hospital mortality in patients with septic shock**					
Training programme in ICU	%	0.165	0.035	0.768	0.022
Respiratory support					
Mechanical ventilation	%	12.005	1.355	106.387	0.026
Additional ICU support					
Fresh frozen plasma transfusion	%	4.361	1.296	14.671	0.017
Constant		0.328			0.364

*CI* confidence interval, *ICU* intensive care unit, *OR* odds ratio, *SOFA* sequential organ failure assessment.

## Discussion

In this cross-sectional, 4-day point prevalence study, 16.2% of ICU admissions in Vietnam were due to sepsis (Table S41 as shown in Additional file 3). Our figure for the prevalence of sepsis is in line with the figure reported in the worldwide Intensive Care over Nations (ICON) study (13.6% [134/982] to 39.3% [372/946] in the different regions)^5^, but lower than the figure reported in the EPIC III study (43.0% [141/328] in Australasia to 60.1% [1892/3150] in Asia and the Middle East)^5,16^. These differences are because the EPIC III included ICU-acquired infection and not specifically sepsis^16^.

Of 252 patients with sepsis included in our analysis, about a third (33.3%) died in the ICU and two-fifths (40.1%) died during the hospital stay (Fig. 1). Our figures for the ICU and hospital mortality rates also are in line with the figures reported in the ICON study (11.9% [16/439] to 39.5 [15/141], and 19.3% [26/439] to 47.2% [17/141], respectively) and in a Brazilian nationwide study (31.8% [1431/4505]) and 41.4% [1867/4505], respectively)^5,34^. In our study, however, the ICU and hospital mortality rates are lower than that reported in the MOSAICS I study (36.7% [471/1285] and 44.5% [572/1285], respectively)^17^. Along with the definition and the management of sepsis have evolved tremendously in the past decade^1,33,35,36^, the compelling nature of the evidence in the literature which has demonstrated an association between compliance with the 1-h, 3-h, or 6-h sepsis bundle of care and the improved survival in patients with sepsis and septic shock^12,33,36,37^. Our study shows that the rates of compliance with the bundles for sepsis were low (Table 5), but higher than that reported in the MOSAICS I (2.3% [4/176], 6.9% [37/540], and 10.0% [57/569] in LICs, MICs, and HICs, respectively)^17^. In addition, our data reveal that the 3-h sepsis bundle of care and administration of antibiotics within the 3 h were associated with the decreased risk of deaths during the ICU and hospital stay (Table 7 and Table S20 in Additional file 3). These associations also are found in the previous study^12^. These findings, therefore, might explain why the hospital and ICU mortality rates were lower in our study compared to that in the MOSAICS I and highlight that compliance with the sepsis bundles of care need to be enhanced.

Despite the distinct inclusion criteria, our median SOFA score upon admission into the ICU is in line with those reported in the EPIC III (7 points; IQR: 4–11)^16^. In contrast, our proportions for the ICU and hospital mortality were higher than rates reported in the EPIC III (23.6% [1870/7936] and 30.3% [2404/7936], respectively)^16^. The present study also shows that a substantial rate of patients was from ICUs with the low nurse-to-patient and intensivist-to-patient ratios (Table 1), especially the ICUs with the intensivist-to-patient ratio of 1:6 to 8 was associated with increased risk of deaths in ICUs (Table S20 as shown in Additional file 3). Economic and political reforms have spurred rapid economic growth in Vietnam^28^. However, healthcare providers still had difficulty in caring for patients with sepsis in both local and central settings because of low resources, such as the low nurse-to-patient and/or intensivist-to-patient ratios, and inadequate treatment strategies for critical care^27,29,30^. At the same time, healthcare providers may not be sufficiently trained or experienced enough to be able to recognize early severe sepsis in their patients and provide the required critical care^29,30^. A previous study shows that ICUs with critical care training programs are generally associated with improving patient outcomes than ICUs without such training programs^38^. However, our study shows that only two-fifths of patients with sepsis were from university-affiliated hospitals and only four-fifths from ICUs with accredited training programs (Table 1). Therefore, these findings might impact negatively the outcomes, explain why ICU and hospital mortality rates were higher in our study compared to that in the EPIC III, and highlight the need for increasing resources in critical care settings and educational interventions to help healthcare providers to care for critically ill patients. In addition, our study shows that the ICU with accredited training programs was inversely and independently associated with the risk of deaths during the ICU and hospital stay (Table 7 and Table S20 in Additional file 3). Thus, to reduce mortality, more healthcare providers should be trained in accredited critical care training programs, such as the evidence-based and interactive critical care training short courses for non-specialty and specialty healthcare providers in resource-limited settings^39^.

In our study, the most common pathogens were consistently Gram-negative bacteria (e.g., *Acinetobacter baumannii*), followed by Gram-positive bacteria, fungi, and viruses (Table 4). The EPIC III also shows that the proportion of infection caused by *Acinetobacter* species in ICUs was highest in Asia (25.6%; 309/1207), which was more than 25 times in compared with North America (1%; 4/396)^16^. This highlights the importance of considering empirical antibiotics based on antibiotic-resistance patterns. In Vietnam, *Acinetobacter baumannii* is the most common nosocomial pathogen (24.4%; 177/726) in ICUs, the rate of infections with Carbapenem resistance *Acinetobacter baumannii* is very high (89.2%; 149/167), and there are many factors independently associated with the increased risk of HAIs in the ICU, including intubation, urinary catheter, central vascular catheter, and peripheral vascular catheter^27^. The present study shows that *Acinetobacter baumannii*, was significantly less often isolated from patients who survived than those who died in the hospital (Table 4). In our study, however, non-surgical source control was independently associated with the decreased risk of deaths in the ICUs (Table S20 as shown in Additional file 3). Thus, to reduce mortality, improvements are needed in infection prevention and control in ICUs.

In our study, invasive organ support therapies during ICU stay (i.e., MV and RRT) were more often given to patients who died than that to patients who survived (Table 6 and Table S18 in Additional file 3). These could be due to the severity of the illness (i.e., SOFA and APACHE II scores) were significantly worse in patients who received invasive organ support therapies than that in patients who did not receive invasive organ support therapies during the ICU stay (Tables S8, S9, S28 and S29 as shown in Additional file 3). However, we found that RRT was independently associated with ICU mortality of patients with sepsis and septic shock (Tables S20 and S40 as shown in Additional file 3). The previous studies showed no benefit from increasing intensity of RRT for acute kidney injury and sepsis^40–42^. In fact, the increasing intensity of RRT is not innocuous. There are several known adverse consequences that are associated with a greater dose including electrolyte abnormalities such as hypophosphatemia and hypokalemia^43^; enhanced elimination of antibiotics that leads to inadequate dosing^44^; excessive nutrient losses such as amino acids and proteins^45^; and lower urine output^46^. These findings might negatively impact outcomes. Thus, to decrease mortality, more optimal management of underlying illness and shock in patients with sepsis are needed.

Our study has some limitations. First, due to the absence of a national registry of ICUs to allow systematic recruitment of units, we used a snowball method to identify suitable units, which might have led to the selection of centers with a greater interest in sepsis management. Therefore, our data are subject to selection bias^47^ and might not fully reflect intensive care throughout Vietnam. Second, due to the study’s real-world nature, we did not protocolize microbiological investigations. Moreover, we mainly evaluated resources utilized in ICUs; therefore, the data detailing the point-of-care testing (e.g., lactate clearance) and life-sustaining treatments (e.g., fluid balance, administration of steroids, and modalities of RRT and MV) were not available. Third, to improve the feasibility of conducting the study in busy ICUs, we opted not to collect data on antibiotic resistance and appropriateness. Fourth, we studied the low events data, some binary dependent variables with dozens of times fewer ones (events) than zeros ("non-events"). Rare events data (defined as variables with 5% lower events than non-events) results in statistical procedures, such as logistic regression, that might sharply underestimate the probability of events^48^. In our study, however, most variables with 5% higher events (e.g., APACHE II score of 0–9, surgical source control) than non-events were included in the multivariable prediction model if the P-value was < 0.05 in the bivariate analysis. Therefore, the underestimation bias in our model, though possible, is less likely to impact the event probability estimation significantly. Finally, although an advantage of the present study was data from the multicenter, which had little missing data, the sample size was relatively small that might lead to overfitting in the multivariable prediction model^49^. Thus, further studies with larger sample sizes might be needed to consolidate the conclusions.

In summary, this was a selected cohort of patients with sepsis admitted to the ICUs with low nurse-to-patient and/or intensivist-to-patient ratios in Vietnam with high mortality. ICUs with accredited training programs, completion of the 3-h sepsis bundle of care and the initial administration of antibiotics within 3 h were inversely and independently associated with death in the hospital. ICUs with intensivist-to-patient ratio of 1:6–8, MV and RRT were independently associated with death in the ICU, in contrast to non-surgical source control which was inversely and independently associated with death in the ICU. To decrease mortality in patients with sepsis in ICUs, the management of sepsis in Vietnam needs to be enhanced through, for example increasing resources in critical care settings; making accredited critical care training programs more available; improving compliance with sepsis bundles of care; and treating underlying illness and shock optimally in patients with sepsis.

## Supplementary Information

Supplementary Information 1.

Supplementary Information 2.

Supplementary Information 3.

## Data Availability

All data generated or analyzed during this study are included in this published article (and its Supplementary Information files).
