# Supplementary material for: Factors relating to mortality in septic patients in Vietnamese intensive care units from a subgroup analysis of MOSAICS II study
Source: Sci Rep. 2021 Sep 23;11:18924. doi: 10.1038/s41598-021-98165-8 (PMC8460806; doi:10.1038/s41598-021-98165-8)
Supplement: Supplementary file 1 — Supplementary Information 1. [file 41598_2021_98165_MOESM1_ESM.pdf]

MOSAICS II Study

Please fill in all blanks before submission

Country: ____________________________ Hospital/ICU: ____

Patient Index Number: ____________________________________ Age of Patient: ___________

Admission Date to your hospital (DD/MM/YYYY): ______________ Gender (Tick one): ☐ Male ☐ Female

Admission Date to your ICU (DD/MM/YYYY): ______________

1. Comorbidities *Please circle yes or no*

Cardiovascular disease	Ischaemic heart disease (IHD), heart failure	Yes/No
Chronic Lung Disease	Chronic obstructive pulmonary disease (COPD), asthma, bronchiectasis, post-tuberculosis related lung disease, interstitial lung disease (ILD), excluding primary or secondary lung malignancy. Patients who are undergoing treatment for tuberculosis or non-tuberculosis mycobacterium (NTM) prior to ICU admission should be included in this category	Yes/No
Chronic neurological disease	Strokes, neuromuscular disease, epilepsy, movement disorders, excluding brain tumors	Yes/No
Chronic Kidney Disease	Kidney damage $\geq$ 3 months (abnormal blood/urine composition or radiological renal abnormalities or glomerular filtration rate < 60mL/min/1.73m2), excluding renal cell carcinoma	Yes/No
Peptic Ulcer Disease	Gastric and duodenal ulcers	Yes/No
Chronic liver disease	Prolonged course of hepatic disease > 6 months, excluding hepatocellular cancer	Yes/No
Diabetes mellitus	Any type of diabetes mellitus	Yes/No
Human immunodeficiency virus (HIV) infection	Positive HIV serology with or without acquired immunodeficiency syndrome (AIDS)-defining illness	Yes/No
Connective tissue disease	Presence of appropriate clinical symptoms and high titres of specific autoantibodies that fulfill the criteria of different connective tissue diseases. Examples include systemic lupus erythematosus (SLE), rheumatoid arthritis (RA), gout, systemic sclerosis, scleroderma	Yes/No
Immunosuppression	Patients on long term steroids or other immunosuppressants (excluding patients with haematological conditions or other malignancies)	Yes/No
Haematological malignancies	Include leukaemia, lymphoma, multiple myeloma	Yes/No
Solid malignant tumours	Such as breast, colon, lung, prostate, skin, etc	Yes/No
Others (Please specify):		

1a. Type of Admission (Tick one):

☐ Medical
☐ Elective Surgical
☐ Unscheduled Surgical

1b. Source of Admission (Tick one):

- ☐ Emergency Department
- ☐ Operating Room
- ☐ General Wards
- ☐ Other ICUs or High-dependency Units
- ☐ Inter-hospital Transfer
- ☐ Others

2. Vital Signs (Upon admission into ICU)

Mean blood or arterial pressure (mmHg):		Systolic blood pressure (mmHg):	
Heart rate (beats per min):		Temperature (degree Celsius):	
Respiratory rate (breaths per min):		Glasgow Coma Scale:	

3. Blood Investigations

Record those that are obtained within the first 24 hours of ICU admission. The results closest to the time of ICU admission should be the ones recorded. If no such investigations are available within the 1st 24 hours of ICU admission, record the results which are obtained within 4 hours prior to ICU admission, with preference being given to those closest to the time of ICU admission

Total white cell count ($x10^9$/L):		Platelets ($x10^9$/L):	
Haemoglobin (g/dl):		Haematocrit (%):	
Potassium (mmol/l):		Sodium (mmol/l):	
Creatinine (umol/l):		Bilirubin (umol/l):	
pH:		PaO_2 (mmHg):	
FiO_2:		PaO_2/FiO_2 ratio:	

Oxygen tank flow rate in liters/min	FiO_2
Nasal cannula	
0 L/min	0.21
1 L/min	0.24
2 L/min	0.28
3 L/min	0.32
4 L/min	0.36
5 L/min	0.40
6 L/min	0.44
Face mask	
5 L/min	0.4
6 L/min	0.5
7-8 L/min	0.6
Nasopharyngeal catheter	
4 L/min	0.4
5 L/min	0.5
6 L/min	0.6
Venturi mask	FiO_2 as set on the apparatus
Face mask with reservoir	
6 L/min	0.6
7 L/min	0.7
8 L/min	0.8
9 L/min	0.9
10 L/min	0.95
15 L/min (non-rebreather mask)	1.0
High flow nasal cannula, non-invasive ventilation, mechanical ventilation	
Regardless of inspiratory flow, positive end-expiratory pressure (PEEP)	FiO_2 as set on the apparatus

4. Severity of Illness Scores

qSOFA at time of ICU admission ________

SIRS at time of ICU admission ________

SOFA at time of ICU admission ________

APACHE II (over first 24 hours of ICU admission) ________

5. Site of Infection

Definition of infections

- *Pneumonia requires the presence of radiographic infiltrates and features including fever or hypothermia, leukocytosis or leukopenia and purulent respiratory secretions.*
- *Intra-abdominal infections include but are not limited to intra-abdominal abscesses, peritonitis, biliary tract infections, pancreatic infections, enteritis, and colitis.*
- *Urinary tract infection requires typical features of fever, urgency, frequency, dysuria, pyuria and haematuria, together with confirmatory radiological features and/or positive culture results.*
- *Soft tissue and skin infections include surgical site infections, septic arthritis, cellulitis and necrotizing fasciitis.*
- *Catheter-related blood stream infection is defined as bacteremia with an intravascular device in situ and no other apparent source for the bloodstream infection, with culture results.*
- *Systemic infections refer to infections without a clear primary site of infection, as is often the case in infections such as dengue and malaria.*

Please tick all that apply

- ☐ Respiratory
- ☐ Urinary Tract
- ☐ Abdominal
- ☐ Neurological
- ☐ Bones or Joints
- ☐ Skin or Cutaneous Sites
- ☐ Intravascular Catheter
- ☐ Infective Endocarditis
- ☐ Primary Bacteraemia
- ☐ Systemic
- ☐ Others, Please Specify: ____

6. Positive Cultures

Please tick all that apply

**Gram-positive**	**Gram-negative**	**Fungal**
☐ Methicillin-sensitive *Staphylococcus aureus*	☐ *Klebsiella pneumoniae*	☐ *Candida albicans*
☐ Methicillin-resistant *Staphylococcus aureus*	☐ *Escherichia coli*	☐ *Candida* non-albicans
☐ *Streptococcus pneumoniae*	☐ *Pseudomonas aeruginosa*	☐ *Aspergillus* species
☐ Other *Streptococcus* species	☐ *Acinetobacter baumannii*	
☐ *Enterococcus*	☐ *Bulkholderia pseudomallei*	
	☐ *Enterobacter cloacae*	
	☐ *Haemophilus influenza*	
	☐ *Salmonella* species	
	☐ *Citrobacter* species	
	☐ *Stenotrophomonas maltophilia*	
	☐ *Proteus* species	
	☐ *Bacteroides fragilis*	

- ☐ Negative Cultures
- ☐ Others, Please Specify: ____

7. Positive Serologies, Molecular, or Histological Tests

Please tick all that apply

Tetanus		☐
Tuberculosis	Positive culture from any medium	☐
	Histological diagnosis	☐
	Polymerase chain reaction (PCR) tests	☐
Non-tuberculous mycobacteria	Positive culture from any medium	☐
	Histological diagnosis	☐
Aspergillosis		☐
Blastomycosis		☐
Histoplasmosis	Antigen testing (Urine)	☐
	Antigen testing (Serum)	☐
Cryptococcus		☐
Malaria	Thick and thin blood films	☐
	Rapid diagnostic tests (Serum)	☐
Measles	Serum serology (presence of IgM)	☐
	PCR tests	☐
Chikungunya	Serum	☐
	CSF serology (presence of IgM)	☐
	PCR tests	☐
Dengue	Serum antigen	☐
	Serology (presence of IgM)	☐
	PCR tests	☐
Influenza	PCR tests	☐
	Immunofluorescence assays from BAL	☐
	Endotracheal aspirate	☐
	Nasopharyngeal swabs	☐
	Nasal swabs	☐
	Throat swabs	☐
Other respiratory viruses apart from influenza	PCR tests	☐
	Immunofluorescence assays from BAL	☐
	Endotracheal aspirate	☐
	Nasopharyngeal swabs	☐
	Nasal swabs	☐
	Throat swabs	☐
Rabies		☐
Leptospirosis		☐
Q fever		☐
Rickettsia	Serum	☐
	Eschar serology (presence of IgM)	☐

Please circle yes or no

7a. Clinical diagnosis made and persisted with due to strong clinical suspicion even though cultures, serologies, molecular, and/or histological tests were performed and negative	Yes / No
7b. Clinical diagnosis made and persisted with due to strong clinical suspicion because cultures, serologies, molecular, and/or histological tests are not available in the ICU	Yes / No

8. Measurements around Time Zero

8a. Time Zero (DD/MM/YY (HH:MM)) : _______________(__________)

Time zero is the onset of sepsis. This is determined according to the patient's location within the hospital when sepsis is diagnosed:

i. For patients presenting to the emergency department with sepsis, time zero is defined as the time of triage.

ii. For patients who develop sepsis in the wards or other non-emergency department units, time zero is determined by searching the clinical documentation for the time of diagnosis of sepsis. This may include, for example, a physician's note or timed and dated orders, a timed and dated note of a nurse's discussion of sepsis with a physician, or timed records initiating referral to the ICU for sepsis.

iii. If no time and date can be found by searching the chart, the default time of presentation is the time of admission to the ICU.

iv. In the rare event that the patient does not present with sepsis in the emergency department, but deteriorates and develops sepsis in the emergency department while being observed or while waiting for a hospital bed, time zero is determined by searching the clinical documentation for the time of diagnosis of sepsis. This may include, for example, a physician's note or timed and dated orders, a timed and dated note of a nurse's discussion of sepsis with a physician, or timed records initiating referral to the ICU for sepsis.

For the following questions, please circle yes or no

<u>Blood Culture</u>

8.1. Was blood culture performed between 1 hour before time zero to 24 hours after time zero?	Yes / No

8.2. If yes, time of blood culture (DD/MM/YY (HHMM)) : _______________(__________)

<u>Lactate Measurement</u>

8.3. Was lactate measured between 1 hour before time zero to 24 hours after time zero?	Yes / No

8.3a. If yes:

Time of lactate measurement (DD/MM/YY (HHMM)) : _______________(__________)

Lactate value (mmol/l): _____________

Antibiotic Administration

8.4: Was antibiotic administered between 1 hour before time zero to 24 hours after time zero? Yes / No

8.4.a: If yes, time of antibiotic administration (DD/MM/YY (HHMM)) : _____________(_________)

Fluid Bolus

8.5: Was there any episode of hypotension (SBP < 90 mmHg or mean blood pressure or MAP < 65 mmHg) between time zero and 1 hour after time zero? Yes / No

8.5.a: If the answer to 8.5 is no, was there any episode of hypotension (SBP < 90 mmHg or mean blood pressure or MAP < 65 mmHg) between time zero and 3 hours after time zero? Yes / No

8.5.b: If answer to questions 8.5 & 8.5.a is yes, was any vasopressor started between 1 hour before time zero to 24 hours after time zero? Yes / No

8.5.c: If answer to 8.5.b is yes, time of starting vasopressor (DD/MM/YY (HHMM)) : _____________(_________)

8.5.d: Amount of fluid bolus administered within 1 hour from time zero (mLs): ____________

8.5.e: Amount of fluid bolus administered within 3 hours from time zero (mLs): ____________

9. Resources used in ICU (Anytime during ICU stay)

For the following questions, please circle yes or no

9.1: Vasopressors / Intropes Yes / No

9.2: Mechanical ventilation (MV), performed through a laryngeal mask, an endotracheal, endobronchial or tracheostomy tube Yes / No

If answer is no, skip next question

9.3: If yes, duration of MV, defined as from the time of starting invasive MV until the patient has been successfully extubated or breathing on a tracheostomy mask for ≥ 48 hours, whichever comes first (days) __________

9.4: Noninvasive ventilation (NIV) using NIPPV or CPAP (excludes NIV used peri-intubation and extubation) Yes / No

If answer is no, skip next question

9.5: If yes, duration of NIV, defined as from the time of starting NIV until the patient has been successfully weaned off for > 24 hours or required subsequent intubation, whichever comes first (days) __________

9.6: High-flow nasal cannula (HFNC) (excludes HFNC used peri-intubation and extubation) Yes / No

If answer is no, skip next question

9.7: If yes, duration of HFNC, defined as from the time of starting HFNC until patient has been successfully weaned off for > 24 hours or required subsequent intubation, whichever comes first (days) ____________

9.8: Renal replacement therapy (IHD, PD, SLED or CRRT) Yes / No

9.9: Transfusion of packed red blood cells Yes / No

9.10: Transfusion of platelets Yes / No

9.11: Transfusion of fresh frozen plasma Yes / No

9.12: Non-surgical source control measure implemented e.g. removal of infected intravascular or other catheters, insertion of ascitic drains, pleural drains, percutaneous drains, and others Yes / No

9.13: Surgical source control measure implemented e.g. debridement of infected necrotic tissue Yes / No

9.14: Time of first source control measure, if any (DD/MM/YY (HHMM)) : ____________(_______)

10. Limitations of Life-Sustaining Treatments

Please tick all that apply

- ☐ Do-not-resuscitate (DNR) order
- ☐ Withdrawal of life-sustaining treatments
- ☐ Withholding of life-sustaining treatments

11. Outcome

Please pick only one

- ☐ Alive upon current hospital stay discharge
- ☐ Alive upon discharge from current ICU stay, but died in current hospital stay
- ☐ Died in current ICU stay

Discharge date from current ICU stay or death date in your current ICU stay (DD/MM/YYYY): _________

Discharge date from current hospital stay or death date in your current hospital stay (DD/MM/YYYY): _________
