# Supplementary material for: Factors relating to mortality in septic patients in Vietnamese intensive care units from a subgroup analysis of MOSAICS II study
Source: Sci Rep. 2021 Sep 23;11:18924. doi: 10.1038/s41598-021-98165-8 (PMC8460806; doi:10.1038/s41598-021-98165-8)
Supplement: Supplementary file 2 — Supplementary Information 2. [file 41598_2021_98165_MOESM2_ESM.pdf]

ICU Questionnaire

Please fill in all blanks before submission

Name of person entering data: ____

Country: ____________________ Hospital: ____________________ ICU: ________________________

Type of hospital (tick one):
- ☐ Rural
- ☐ Urban

University or university-affiliated hospital (tick one):
- ☐ Yes
- ☐ No

** Rural is defined as non-metropolitan areas, catering to patients not in the city as defined by the country's healthcare system*

Number of beds in hospital: ______

Number of beds in ICU: ______

Type of ICU (tick one):
- ☐ Medical
- ☐ Surgical
- ☐ Mixed medical & surgical
- ☐ Others (excluding paediatric, coronary and neurosurgical ICUs)

Nurse to ICU patient ratio (Tick one; only 1 choice allowed, choose the ratio most frequently seen in your ICU)

- ☐ 1 or more nurses : 1 patient
- ☐ 1 nurse : 2 patients
- ☐ 1 nurse : 3 patients
- ☐ 1 nurse : 4 or more patients

Nature of ICU (Tick one; only 1 choice allowed)

- ☐ Closed ICU = All patients are cared for by 1 team of intensivists in collaboration with a primary service. Only intensivists have admitting privileges to the ICU
- ☐ Open ICU = Any physician can admit patients to the ICU. The primary service (not intensivists) takes main responsibility for care of patients. If an ICU functions as an open ICU some of the time, and as a closed ICU some of the time, please tick "Open ICU"

Only for closed ICUs: Intensivist to ICU patient ratio (Tick one; only 1 choice allowed, choose the ratio most frequently seen in your ICU)

- ☐ 1 intensivist : 5 or fewer patients
- ☐ 1 intensivist: 6 to 8 patients
- ☐ 1 intensivist: 9 to 11 patients
- ☐ 1 intensivist: 12 to 14 patients
- ☐ 1 intensivist: 15 or more patients

Is the ICU part of an accredited intensive care fellowship programme? (Tick one)

- ☐ Yes
- ☐ No

Capabilities (Tick all that apply)

- ☐ Able to perform routine blood, urine, stool, CSF, synovial, fluid cultures
- ☐ Able to process AFB smear and cultures
- ☐ Able to perform PCR testing for tuberculosis
- ☐ Able to perform serology or PCR testing for dengue
- ☐ Able to perform serology or PCR testing for influenza
- ☐ Able to test for galactomannan
- ☐ Able to perform blood film identification for malaria

Possible additional practices in the general ward outside of the ICU and outside of any high dependency or intermediate care ward or dialysis unit (Tick all that apply)

- ☐ Able to support patients on noninvasive ventilation in the general ward
- ☐ Able to support patients on invasive mechanical ventilation in the general ward
- ☐ Able to support patients on vasopressor/inotrope infusions in the general ward
- ☐ Able to support patients on dialysis/renal replacement therapy in the general ward

Please send completed form to:	Wizlink Consulting Pte Ltd<br>Vision Exchange, #16-16<br>2 Venture Drive<br>Singapore 608526
