# Supplementary material for: Factors relating to mortality in septic patients in Vietnamese intensive care units from a subgroup analysis of MOSAICS II study
Source: Sci Rep. 2021 Sep 23;11:18924. doi: 10.1038/s41598-021-98165-8 (PMC8460806; doi:10.1038/s41598-021-98165-8)
Supplement: Supplementary file 3 — Supplementary Information 3. [file 41598_2021_98165_MOESM3_ESM.docx]

**SUPPLEMENTARY RESULTS**

Day of patient enrollment from 15 adult ICUs on the 4 days identified in year (i.e. 9th January, 3rd April, 3rd July, and 9th October, 2019)

Exclude patients from

- Pediatric ICUs
- Neurosurgical ICUs
- Coronary and cardiac ICUs

252 patients eligible for analysis

2 patients admitted on day of patient enrollment for sepsis and died/discharged on same day

220 patients admitted before day of patient enrollment for sepsis but still in ICU on day of patient enrollment

30 patients admitted on day of patient enrollment for sepsis and stayed beyond day of patient enrollment

Data collection of demographics, clinical parameters and investigations

Data collection on sepsis bundle and ICU resource utilization

Data collection of patient's outcomes

84 patients died in ICU stay

101 patients died in hospital stay

**Figure S1.** Flowchart of the study design, patient enrollment and follow up. Abbreviations: ICU, intensive care unit.

**Table S1.** Hospital and intensive care unit characteristics according to hospital survivability of patients with sepsis

| Variable | All cases  n=252 | Survived  n=151 | Died  n=101 | P |
| --- | --- | --- | --- | --- |
| Participating hospital, no. (%) |  |  |  | - |
| 115 People's | 25 (9.9) | 6 (4.0) | 19 (18.8) |  |
| Bach Mai | 26 (10.3) | 14 (9.3) | 12 (11.9) |  |
| Bai Chay | 14 (5.6) | 10 (6.6) | 4 (4.0) |  |
| Can Tho | 7 (2.8) | 1 (0.7) | 6 (5.9) |  |
| Cho Ray | 41 (16.3) | 19 (12.6) | 22 (21.8) |  |
| Da Nang | 12 (4.8) | 6 (4.0) | 6 (5.9) |  |
| Dong Da | 9 (3.6) | 6 (4.0) | 3 (3.0) |  |
| Hanoi Medical University | 12 (4.8) | 6 (4.0) | 6 (5.9) |  |
| Hue | 39 (15.5) | 26 (17.2) | 13 (12.9) |  |
| Saint Paul | 9 (3.6) | 9 (6.0) | 0 |  |
| Thai Nguyen | 2 (0.8) | 1 (0.7) | 1 (1.0) |  |
| Thanh Nhan | 1 (0.4) | 0 | 1 (1.0) |  |
| Vietnam–Czechoslovakia Friendship | 48 (19.0) | 40 (26.5) | 8 (7.9) |  |
| Vinmec Times City International | 7 (2.8) | 7 (4.6) | 0 |  |
| **Hospital characteristics** | | | | |
| Type of hospital, no. (%) |  |  |  | - |
| Rural | 0 | 0 | 0 |  |
| Urban | 252 (100) | 151 (100) | 101 (100) |  |
| University affiliation, no. (%) |  |  |  | <0.001 |
| No | 153 (60.7) | 105 (69.5) | 48 (47.5) |  |
| Yes | 99 (39.3) | 46 (30.5) | 53 (52.5) |  |
| **ICU characteristics** | | | | |
| Nature of ICU, no. (%) |  |  |  | - |
| Open | 0 | 0 | 0 |  |
| Closed | 252 (100) | 151 (100) | 101 (100) |  |
| Type of ICU, no. (%) |  |  |  | 0.589 |
| Medical | 110 (43.7) | 68 (45.0) | 42 (41.6) |  |
| Surgical | 0 | 0 | 0 |  |
| Mixed | 142 (56.3) | 83 (55.0) | 59 (58.4) |  |
| Nurse to patient ratio, no. (%) |  |  |  | 0.079 |
| 1 or more nurses : 1 patient | 7 (2.8) | 7 (4.6) | 0 |  |
| 1 nurse : 2 patients | 187 (74.2) | 111 (73.5) | 76 (75.2) |  |
| 1 nurse : 3 patients | 0 | 0 | 0 |  |
| 1 nurse : 4 or more patients | 58 (23.0) | 33 (21.9) | 25 (24.8) |  |
| Intensivist to patient ratio, no. (%) |  |  |  | 0.446 |
| 1 intensivist : 5 or fewer patients | 165 (65.5) | 96 (63.6) | 69 (68.3) |  |
| 1 intensivist : 6 to 8 patients | 75 (29.8) | 49 (32.5) | 26 (25.7) |  |
| 1 intensivist : 9 to 11 patients | 0 | 0 | 0 |  |
| 1 intensivist : 12 or more patients | 12 (4.8) | 6 (4.0) | 6 (5.9) |  |
| Training programme in ICU, no. (%) |  |  |  | 0.010 |
| No | 50 (19.8) | 22 (14.6) | 28 (27.7) |  |
| Yes | 202 (80.2) | 129 (85.4) | 73 (72.3) |  |

**Table S2.** Baseline characteristics according to hospital survivability of patients with sepsis

| Variable | All cases  n=252 | Survived  n=151 | Died  n=101 | P |
| --- | --- | --- | --- | --- |
| Age (year), median (IQR) | 65 (52-76.75) | 65 (53-76) | 65 (52-78) | 0.810 |
| Age (year), no. (%) |  |  |  | 0.865 |
| < 20 | 3 (1.2) | 2 (1.3) | 1 (1.0) |  |
| 20 - 39 | 19 (7.5) | 10 (6.6) | 9 (8.9) |  |
| 40 - 59 | 74 (29.4) | 43 (28.5) | 31 (30.7) |  |
| ≥ 60 | 156 (61.9) | 96 (63.6) | 60 (59.4) |  |
| Sex (male), no. (%) | 162 (64.3) | 93 (61.6) | 69 (68.3) | 0.275 |
| Collection batch, no. (%) |  |  |  | 0.007 |
| Collection 1 (Jan) | 80 (31.7) | 58 (38.4) | 22 (21.8) |  |
| Collection 2 (April) | 62 (24.6) | 27 (17.9) | 35 (34.7) |  |
| Collection 3 (July) | 54 (21.4) | 32 (21.2) | 22 (21.8) |  |
| Collection 4 (Oct) | 56 (22.2) | 34 (22.5) | 22 (21.8) |  |
| Admission type, no. (%) |  |  |  | 0.195 |
| Medical | 236 (93.7) | 138 (91.4) | 98 (97.0) |  |
| Elective surgical | 2 (0.8) | 2 (1.3) | 0 |  |
| Unscheduled surgical | 14 (5.6) | 11 (7.3) | 3 (3.0) |  |
| Admission source, no. (%) |  |  |  | 0.505 |
| Emergency department | 138 (54.8) | 87 (57.6) | 51 (50.5) |  |
| Operating room | 4 (1.6) | 3 (2.0) | 1 (1.0) |  |
| General wards | 56 (22.2) | 33 (21.9) | 23 (22.8) |  |
| Other ICUs or HDU | 16 (6.3) | 10 (6.6) | 6 (5.9) |  |
| Inter-hospital transfer | 37 (14.7) | 18 (11.9) | 19 (18.8) |  |
| Others | 1 (0.4) | 0 | 1 (1.0) |  |
| Comorbidities, no. (%) |  |  |  |  |
| Cardiovascular disease | 78 (31.0) | 41 (27.2) | 37 (36.6) | 0.111 |
| Chronic lung disease | 30 (11.9) | 18 (11.9) | 12 (1.9) | 0.992 |
| Chronic neurological disease | 36 (14.3) | 28 (18.5) | 8 (7.9) | 0.018 |
| Chronic kidney disease | 23 (9.1) | 14 (9.3) | 9 (8.9) | 0.922 |
| Peptic ulcer disease | 9 (3.6) | 5 (3.3) | 4 (4.0) | >0.999 |
| Chronic liver disease | 27 (10.7) | 14 (9.3) | 13 (12.9) | 0.365 |
| Diabetes mellitus | 67 (26.6) | 40 (26.5) | 27 (26.7) | 0.966 |
| HIV infection | 0 | 0 | 0 | - |
| Connective tissue disease | 3 (1.2) | 2 (1.3) | 1 (1.0) | >0.999 |
| Immunosuppression | 10 (4.0) | 7 (4.6) | 3 (3.0) | 0.744 |
| Haematological malignancies | 5 (2.0) | 3 (2.0) | 2 (2.0) | >0.999 |
| Solid malignant tumours | 12 (4.8) | 6 (4.0) | 6 (5.9) | 0.551 |

**Table S3.** Clinical and laboratory characteristics and severity of illness according to hospital survivability of patients with sepsis

| Variable | All cases | Survived | Died | P |
| --- | --- | --- | --- | --- |
| **Vital signs** (on admission into ICU) | | | | |
| GCS, median (IQR) | 13 (9-15) | 14 (10-15) | 10 (8-14) | <0.001 |
| HR (beats per min), median (IQR) | 110 (95.25-125.75) | 110 (92-125) | 110 (100-129.5) | 0.083 |
| Temperature (^o^C), mean (SD) | 37.79 (1.01) | 37.80 (1.08) | 37.77 (0.91) | 0.871 |
| MBP (mmHg), mean(SD) | 75.82 (22.08) | 79.75 (22.88) | 69.93 (19.51) | 0.002 |
| SBP (mmHg), mean (SD) | 106.45 (29.96) | 111.39 (29.44) | 99.07 (29.35) | 0.004 |
| RR (breaths per min), median (IQR) | 25 (22-30) | 25 (22-30) | 25 (20-30) | 0.693 |
| **Blood investigations** | | | | |
| Total WBC (x10^9^/L), mean (SD) | 15.73 (9.20) | 15.63 (8.67) | 15.88 (9.98) | 0.914 |
| PLT (x10^9^/L), mean (SD) | 185.98 (137.85) | 200.71 (129.67) | 163.95 (147.15) | 0.002 |
| Hb (g/dL), mean (SD) | 11.14 (2.59) | 11.36 (2.68) | 10.82 (2.44) | 0.088 |
| Hct (%), mean (SD) | 34.31 (7.75) | 35.08 (7.92) | 33.17 (7.38) | 0.031 |
| K^+^ (mmol/L), mean (SD) | 3.89 (0.79) | 3.90 (0.80) | 3.87 (0.77) | 0.865 |
| Na^+^ (mmol/L), mean (SD) | 136.05 (8.24) | 135.62 (8.81) | 136.69 (7.80) | 0.068 |
| Creatinine (µmol/L), mean (SD) | 187.85 (151.92) | 186.15 (171.60) | 190.38 (117.27) | 0.030 |
| Bilirubin (µmol/l), mean (SD) | 32.80 (61.49) | 31.74 (72.67) | 34.35 (40.09) | 0.007 |
| pH, mean (SD) | 7.37 (0.50) | 7.41 (0.64) | 7.32 (0.14) | 0.004 |
| PaO_2_ (mmHg), mean (SD) | 116.17 (74.28) | 110.23 (56.25) | 124.73 (94.07) | 0.665 |
| FiO_2_, mean (SD) | 0.50 (0.22) | 0.44 (0.18) | 0.58 (0.24) | <0.001 |
| PaO_2_/FiO_2_ ratio, mean (SD) | 262.48 (149.58) | 281.52 (149.39) | 235.26 (146.32) | 0.003 |
| **Severity of illness scores** | | | | |
| qSOFA, median (IQR) | 2 (1-2) | 2 (1-2) | 2 (2-3) | 0.001 |
| qSOFA, no. (%) |  |  |  | 0.055 |
| 0 - 1 | 69 (27.4) | 48 (31.8) | 21 (20.8) |  |
| 2 - 3 | 183 (72.6) | 103 (68.2) | 80 (79.2) |  |
| SIRS, median (IQR) | 3 (2-4) | 3 (2-4) | 3 (2-4) | 0.937 |
| SOFA, median (IQR) | 7 (4.75-10) | 6 (4-9) | 9 (6-12) | <0.001 |
| SOFA, no. (%) |  |  |  | <0.001 |
| 0 - 1 | 0 | 0 | 0 |  |
| 2 - 3 | 46 (18.4) | 33 (22.1) | 13 (12.9) |  |
| 4 - 5 | 36 (14.4) | 28 (18.8) | 8 (7.9) |  |
| 6 - 7 | 58 (23.2) | 43 (28.9) | 15 (14.9) |  |
| 8 - 9 | 32 (12.8) | 16 (10.7) | 16 (15.8) |  |
| 10 - 11 | 38 (15.2) | 20 (13.4) | 18 (17.8) |  |
| 12 - 14 | 29 (11.6) | 8 (5.4) | 21 (20.8) |  |
| > 14 | 11 (4.4) | 1 (0.7) | 10 (9.9) |  |
| SOFA, no. (%) |  |  |  | <0.001 |
| 0 - 3 | 46 (18.4) | 33 (22.1) | 13 (12.9) |  |
| 4 - 7 | 94 (37.6) | 71 (47.7) | 23 (22.8) |  |
| 8 - 9 | 32 (12.8) | 16 (10.7) | 16 (15.8) |  |
| 10 - 11 | 38 (15.2) | 20 (13.4) | 18 (17.8) |  |
| ≥ 12 | 40 (16.0) | 9 (6.0) | 31 (30.7) |  |
| SOFA, no. (%) |  |  |  | <0.001 |
| 0 - 6 | 109 (43.6) | 81 (54.4) | 28 (27.7) |  |
| 7 - 9 | 63 (25.2) | 39 (26.2) | 24 (23.8) |  |
| 10 - 11 | 38 (15.2) | 20 (13.4) | 18 (17.8) |  |
| 12 - 14 | 29 (11.6) | 8 (5.4) | 21 (20.8) |  |
| > 14 | 11 (4.4) | 1 (0.7) | 10 (9.9) |  |
| APACHE II, median (IQR) | 18 (13-24) | 15 (12-21) | 22 (16-27) | <0.001 |
| APACHE II, no. (%) |  |  |  | <0.001 |
| 0 - 4 | 3 (1.2) | 3 (2.0) | 0 |  |
| 5 - 9 | 22 (8.7) | 16 (10.6) | 6 (5.9) |  |
| 10 - 14 | 61 (24.2) | 50 (33.1) | 11(10.9) |  |
| 15 - 19 | 52 (20.6) | 33 (21.9) | 19 (18.8) |  |
| 20 - 24 | 58 (23.0) | 27 (17.9) | 31 (30.7) |  |
| 25 - 29 | 28 (11.1) | 9 (6.0) | 19 (18.8) |  |
| 30 - 34 | 19 (7.5) | 10 (6.6) | 9 (8.9) |  |
| > 34 | 9 (3.6) | 3 (2.0) | 6 (5.9) |  |
| APACHE II, no. (%) |  |  |  | <0.001 |
| 0 - 9 | 25 (9.9) | 19 (12.6) | 6 (5.9) |  |
| 10 - 14 | 61 (24.2) | 50 (33.1) | 11 (10.9) |  |
| 15 - 19 | 52 (20.6) | 33 (21.9) | 19 (18.8) |  |
| 20 - 24 | 58 (23.0) | 27 (19.7) | 31 (30.7) |  |
| 25 - 29 | 28 (11.1) | 9 (6.0) | 19 (18.8) |  |
| ≥ 30 | 28 (11.1) | 13 (8.6) | 15 (14.9) |  |
| Septic Shock | 74 (29.4) | 35 (23.2) | 39 (38.6) | 0.008 |

**Table S4.** Sites of infection and microbiology according to hospital survivability of patients with sepsis

| Variable | All cases  n=252 | Survived  n=151 | Died  n=101 | P |
| --- | --- | --- | --- | --- |
| **Site of Infection** | | | | |
| Respiratory, no. (%) | 143 (56.7) | 82 (54.3) | 61 (60.4) | 0.339 |
| Urinary tract, no. (%) | 37 (14.7) | 30 (19.9) | 7 (6.9) | 0.004 |
| Abdominal, no. (%) | 61 (24.2) | 34 (22.5) | 27 (26.7) | 0.444 |
| Neurological, no. (%) | 12 (4.8) | 8 (5.3) | 4 (4.0) | 0.767 |
| Bones or joints, no. (%) | 2 (0.8) | 2 (1.3) | 0 | 0.518 |
| Skin or cutaneous sites, no. (%) | 19 (7.5) | 7 (4.6) | 12 (11.9) | 0.033 |
| Intravascular catheter, no. (%) | 1 (0.4) | 1 (0.7) | 0 | >0.999 |
| Infective endocarditis, no. (%) | 1 (0.4) | 0 | 1 (1.0) | 0.401 |
| Primary bacteraemia, no. (%) | 7 (2.8) | 5 (3.3) | 2 (2.0) | 0.705 |
| Systemic, no. (%) | 6 (2.4) | 4 (2.6) | 2 (2.0) | >0.999 |
| Others, no. (%) | - | - | - | - |
| **Microbiology** | | | | |
| No pathogens detected, no. (%) | 67 (26.6) | 47 (31.1) | 20 (19.8) | 0.046 |
| Gram negative bacteria, no. (%) | 156 (61.9) | 88 (58.3) | 68 (67.3) | 0.147 |
| *Klebsiella pneumonia* | 27 (10.7) | 16 (10.6) | 11 (10.9) | 0.941 |
| *Acinetobacter baumannii* | 45 (17.9) | 21 (13.9) | 24 (23.8) | 0.045 |
| *Escherichia coli* | 44 (17.5) | 26 (17.2) | 18 (17.8) | 0.902 |
| *Pseudomonas aeruginosa* | 24 (9.5) | 17 (11.3) | 7 (6.9) | 0.251 |
| *Stenotrophomonas maltophilia* | 2 (0.8) | 0 | 2 (2.0) | 0.160 |
| *Proteus species* | 47 (18.7) | 25 (16.6) | 22 (21.8) | 0.297 |
| *Enterobacter cloacae* | 3 (1.2) | 3 (2.0) | 0 | 0.277 |
| *Bulkholderia pseudomallei* | 1 (0.4) | 0 | 1 (1.0) | 0.221 |
| *Others* | 0 | 0 | 0 | - |
| Gram positive bacteria, no. (%) | 34 (13.5) | 22 (14.6) | 12 (11.9) | 0.540 |
| *Enterococcus* | 5 (2.0) | 5 (3.3) | 0 | 0.085 |
| *MSSA* | 5 (2.0) | 3 (2.0) | 2 (2.0) | >0.999 |
| *MRSA* | 10 (4.0) | 6 (4.0) | 4 (4.0) | >0.999 |
| *Other Streptococcus species* | 12 (4.8) | 6 (4.0) | 6 (5.9) | 0.551 |
| *Streptococcus pneumonia* | 2 (0.8) | 2 (1.3) | 0 | 0.518 |
| Fungi, no. (%) | 7 (2.8) | 4 (2.6) | 3 (3.0) | >0.999 |
| *Candida species* | 7 (2.8) | 4 (2.6) | 3 (3.0) | >0.999 |
| *Aspergillus species* | 0 | 0 | 0 | - |
| *Others* | 0 | 0 | 0 | - |
| Viruses, no. (%) | 2 (0.8) | 0 | 2 (2.0) | 0.160 |
| *Influenza* | 1 (0.4) | 0 | 1 (1.0) | 0.401 |
| *Dengue* | 1 (0.4) | 0 | 1 (1.0) | 0.401 |
| *Others* | 0 | 0 | 0 | - |
| Other pathogens, no. (%) |  |  |  |  |
| *Anaerobes* | 0 | 0 | 0 | - |
| *Mycobacterium tuberculosis* | 4 (1.6) | 3 (2.0) | 1 (1.0) | 0.651 |
| *Malaria* | 0 | 0 | 0 | - |

**Table S5.** Completion of sepsis bundle elements according to the hospital survivability of patients with sepsis

| Variable | All cases  n=252 | Survived  n=151 | Died  n=101 | P |
| --- | --- | --- | --- | --- |
| **Timing of antibiotics administration** | | | | |
| Performed within 24 hours, no. (%) | n=225 | n=141 | n=84 | 0.348 |
| 0-60 minutes | 173 (76.9) | 109 (77.3) | 64 (76.2) |  |
| 61-120 minutes | 21 (9.3) | 13 (9.2) | 8 (9.5) |  |
| 121-180 minutes | 14 (6.2) | 11 (7.8) | 3 (3.6) |  |
| >180 minutes | 17 (7.6) | 8 (5.7) | 9 (10.7) |  |
| Not performed within 24 hours, no. (%) | 0 | 0 | 0 | - |
| Timing of antibiotics administration, median (IQR), minutes | 30 (11-60) | 35 (13.5-60) | 30 (10-60) | 0.794 |
| **Timing of obtaining blood cultures** | | | | |
| Performed within 24 hours, no. (%) | n=197 | n=114 | n=83 | 0.838 |
| 0-60 minutes | 135 (68.5) | 77 (67.5) | 58 (69.9) |  |
| 61-120 minutes | 14 (7.1) | 7 (6.1) | 7 (8.4) |  |
| 121-180 minutes | 10 (5.1) | 6 (5.3) | 4 (4.8) |  |
| >180 minutes | 38 (19.3) | 24 (21.1) | 14 (16.9) |  |
| Not performed within 24 hours, no. (%) | 0 | 0 | 0 | - |
| Timing of obtaining blood cultures, median (IQR), minutes | 30.0 (15-114.5) | 30 (14-130.5) | 30 (15-90) | 0.493 |
| **Timing of obtaining lactate measurement** | | | | |
| Performed within 24 hours, no. (%) | n=198 | n=121 | n=77 | 0.790 |
| 0-60 minutes | 141 (71.2) | 85 (70.2) | 56 (72.7) |  |
| 61-120 minutes | 10 (5.1) | 6 (5.0) | 4 (5.2) |  |
| 121-180 minutes | 6 (3.0) | 5 (4.1) | 1 (1.3) |  |
| >180 minutes | 41 (20.7) | 25 (20.7) | 16 (20.8) |  |
| Not performed within 24 hours, no. (%) | 0 | 0 | 0 | - |
| Timing of obtaining lactate measurement, median (IQR), minutes | 30 (10-92) | 30 (10-139.5) | 30 (10-75.5) | 0.583 |

**Table S6.** Completion of the sepsis bundle of care and the initial administration of antibiotics according to the hospital survivability of patients with sepsis

| Variable | All cases  n=252 | Survived  n=151 | Died  n=101 | P |
| --- | --- | --- | --- | --- |
| Completion of the sepsis bundle within 1 hour, no. (%), n=241 | 87 (36.1) | 53 (36.3) | 34 (35.8) | 0.936 |
| Completion of the initial administration of antibiotics within 1 hour, no. (%), n=241 | 173 (71.8) | 109 (74.7) | 64 (63.4) | 0.219 |
| Permutations of the completed elements within 1 hour, no. (%) | n=241 | n=146 | n=95 | 0.196 |
| No elements completed | 20 (8.3) | 12 (8.2) | 8 (8.4) |  |
| Antibiotics only | 44 (18.3) | 30 (20.5) | 14 (14.7) |  |
| Blood cultures only | 13 (5.4) | 9 (6.2) | 4 (4.2) |  |
| Lactate only | 23 (9.5) | 11 (7.5) | 12 (12.6) |  |
| Antibiotics + Lactate | 17 (7.1) | 14 (9.6) | 3 (3.2) |  |
| Antibiotics + Blood cultures | 25 (10.4) | 12 (8.2) | 13 (13.7) |  |
| Blood cultures + Lactate | 12 (5.0) | 5 (3.4) | 7 (7.4) |  |
| Antibiotics + Blood cultures + Lactate | 87 (36.1) | 53 (36.3) | 34 (35.8) |  |
| Completion of the sepsis bundle within 3 hours, no. (%), n=241 | 108 (44.8) | 66 (45.2) | 42 (44.2) | 0.879 |
| Completion of the initial administration of antibiotics within 3 hours, no. (%), n=241 | 205 (85.1) | 131 (89.7) | 74 (779) | 0.012 |
| Permutation of the completed elements of 3-hour sepsis bundle, no. (%) | n=241 | n=146 | n=95 | 0.028 |
| No elements completed | 8 (3.3) | 3 (2.1) | 5 (5.3) |  |
| Antibiotics only | 37 (15.4) | 28 (19.2) | 9 (9.5) |  |
| Blood cultures only | 5 (2.1) | 2 (1.4) | 3 (3.2) |  |
| Lactate only | 16 (6.6) | 8 (5.5) | 8 (8.4) |  |
| Antibiotics + Lactate | 24 (10.0) | 19 (13.0) | 5 (5.3) |  |
| Antibiotics + Blood cultures | 36 (14.9) | 18 (12.3) | 18 (18.9) |  |
| Blood cultures + Lactate | 7 (2.9) | 2 (1.4) | 5 (5.3) |  |
| Antibiotics + Blood cultures + Lactate | 108 (44.8) | 66 (45.2) | 42 (44.2) |  |

**Table S7.** Life-sustaining treatments during ICU stay and outcomes according to hospital survivability of patients with sepsis

| Variable | All cases  n=252 | Survived  n=151 | Died  n=101 | P |
| --- | --- | --- | --- | --- |
| **Life-sustaining treatments during ICU stay** | | | | |
| Respiratory support, no. (%) and median (IQR), days |  |  |  |  |
| Mechanical ventilation | 173/251 (68.9) | 82/150 (54.7) | 91/101 (90.1) | <0.001 |
| Duration of mechanical ventilation | 8 (4-15) | 9 (4-15) | 7(3-14) | 0.153 |
| Non-invasive ventilation | 20/251 (8.0) | 13/150 (8.7) | 7/101 (6.9) | 0.618 |
| Duration of non-invasive ventilation | 2 (2-3.75) | 2 (1-2) | 5 (2-7) | 0.004 |
| High-flow nasal oxygen | 38/251 (15.1) | 29/150 (19.3) | 9/101 (8.9) | 0.024 |
| Duration of high-flow nasal oxygen | 2 (1-3) | 2 (1-3) | 2 (1-3) | >0.999 |
| Additional ICU support, no. (%) |  |  |  |  |
| Vasopressors/inotropes | 163 (64.7) | 82 (54.3) | 81 (80.2) | <0.001 |
| Renal replacement therapy | 101/251 (40.2) | 43/150 (28.7) | 58/101 (57.4) | <0.001 |
| Red blood cell transfusion | 93/251 (37.1) | 48/150 (32.0) | 45/101 (44.6) | 0.043 |
| Platelet transfusion | 50/251 (19.9) | 20/150 (13.3) | 30/101 (29.7) | 0.001 |
| Fresh frozen plasma transfusion | 58/251 (23.1) | 28/150 (18.7) | 30/101 (29.7) | 0.042 |
| Surgical source control | 25/251 (10.0) | 19/150 (12.7) | 6/101 (5.9) | 0.081 |
| Non-surgical source control | 78/251 (31.1) | 54/150 (36.0) | 24/101 (23.8) | 0.040 |
| Length of of surgical source control, median (IQR), minutes | 295.0 (190.0-637.5) | 290.0 (105.0-630.0) | 430.0 (270.0-1587.5) | 0.241 |
| Length of of surgical source control, n (%) | n=24 | n=19 | n=5 | 0.208 |
| <12 hours | 23 (95.8) | 19 (100) | 4 (80.0) |  |
| 12-24 hours | 1 (4.2) | 0 | 1 (20.0) |  |
| >24 hours | 0 | 0 | 0 |  |
| **Outcomes** | | | | |
| Outcomes |  |  |  | <0.001 |
| Alive upon current hospital discharge, no. (%) | 150 (59.5) | 150 (99.3) | 0 |  |
| Alive upon discharge from current ICU stay, but died in current hospital stay, no. (%) | 17 (6.7) | 0 | 17 (16.8) |  |
| Alive upon discharge from current ICU stay, but still in current hospital stay after 90 days, no. (%) | 1 (0.4) | 1 (0.7) | 0 |  |
| Still in current ICU stay after 90 days, no. (%) | 0 | 0 | 0 |  |
| Died in current ICU stay, no. (%) | 84 (33.3) | 0 | 84 (83.2) |  |
| Length of stay, median days (IQR) |  |  |  |  |
| Hospital | 16 (10-25) | 17 (11-24.25) | 13 (7-26) | 0.027 |
| ICU | 10 (6-18) | 10.5 (6-17) | 10 (5-21) | 0.740 |

**Table S8**. Clinical and laboratory characteristics and severity of illness according to methods of mechanical ventilation for patients with sepsis

| Variable | All cases  n=251 | No mechanical ventilation  n=78 | Mechanical ventilation  n=173 | P^a^ |
| --- | --- | --- | --- | --- |
| **Vital signs** (on admission into ICU) | | | | |
| GCS, median (IQR) | 13 (9-15) | 15 (12-15) | 12 (8-15) | <0.001 |
| HR (beats per min), median (IQR) | 110 (95.25-125.75) | 100 (90-116) | 112 (100-130) | <0.001 |
| Temperature (^o^C), mean (SD) | 37.79 (1.01) | 37.87 (1.09) | 37.75 (0.98) | 0.536 |
| MBP (mmHg), mean(SD) | 75.82 (22.08) | 81.07 (22.87) | 73.52 (21.42) | 0.014 |
| SBP (mmHg), mean (SD) | 106.45 (29.96) | 110.89 (27.87) | 104.55 (30.80) | 0.090 |
| RR (breaths per min), median (IQR) | 25 (22-30) | 25 (21-28) | 26 (22-30) | 0.111 |
| **Blood investigations** | | | | |
| Total WBC (x10^9^/L), mean (SD) | 15.73 (9.20) | 15.78 (9.25) | 15.58 (9.08) | 0.836 |
| PLT (x10^9^/L), mean (SD) | 185.98 (137.85) | 181.72 (120.35) | 187.08 (145.33) | 0.728 |
| Hb (g/dL), mean (SD) | 11.14 (2.59) | 11.51 (2.74) | 11 (2.50) | 0.080 |
| Hct (%), mean (SD) | 34.31 (7.75) | 35.27 (8.26) | 33.94 (7.47) | 0.116 |
| K^+^ (mmol/L), mean (SD) | 3.89 (0.79) | 3.83 (0.79) | 3.90 (0.78) | 0.546 |
| Na^+^ (mmol/L), mean (SD) | 136.05 (8.24) | 133.04 (6.81) | 137.42 (8.50) | <0.001 |
| Creatinine (µmol/L), mean (SD) | 187.85 (151.92) | 185.09 (166.16) | 189.38 (145.95) | 0.295 |
| Bilirubin (µmol/l), mean (SD) | 32.80 (61.49) | 45.38 (100.70) | 27.63 (33.21) | 0.822 |
| pH, mean (SD) | 7.37 (0.50) | 7.50 (0.88) | 7.32 (0.13) | <0.001 |
| PaO_2_ (mmHg), mean (SD) | 116.17 (74.28) | 103.48 (52.99) | 121.66 (81.32) | 0.163 |
| FiO_2_, mean (SD) | 0.50 (0.22) | 0.38 (0.17) | 0.55 (0.22) | <0.001 |
| PaO_2_/FiO_2_ ratio, mean (SD) | 262.48 (149.58) | 306.63 (163.77) | 243.14 (139.44) | 0.002 |
| **Severity of illness scores** | | | | |
| SOFA, median (IQR) | 7 (4.75-10) | 5 (3-7) | 9 (6-11) | <0.001 |
| APACHE II, median (IQR) | 18 (13-24) | 13 (9.75-18) | 21 (15-26) | <0.001 |
| Septic Shock | 74 (29.5) | 13 (16.7) | 61 (35.3) | 0.003 |
| **Life-sustaining treatments during ICU stay** | | | | |
| Additional ICU support, no. (%) |  |  |  |  |
| Vasopressors/inotropes | 163 (64.9) | 26 (33.3) | 137 (79.2) | <0.001 |
| Renal replacement therapy | 101 (40.2) | 13 (16.7) | 88 (50.9) | <0.001 |
| Red blood cell transfusion | 93 (37.1) | 14 (17.9) | 79 (45.7) | <0.001 |
| Platelet transfusion | 50 (19.9) | 5 (6.4) | 45 (26.0) | <0.001 |
| Fresh frozen plasma transfusion | 58 (23.1) | 10 (12.8) | 48 (27.7) | 0.009 |
| Surgical source control | 25 (10.0) | 6 (7.7) | 19 (11.0) | 0.420 |
| Non-surgical source control | 78 (31.1) | 19(24.4) | 59 (34.1) | 0.123 |
| **Outcome** | | | | |
| Length of stay, median days (IQR) |  |  |  |  |
| ICU | 10 (6-18) | 7 (5-13) | 13 (6-21) | <0.001 |
| Hospital | 16 (10-25) | 14 (9.5-20) | 18 (11-28) | 0.016 |
| Mortality, no. (%) |  |  |  |  |
| ICU | 84 (33.5) | 8 (10.3) | 76 (43.9) | <0.001 |
| Hospital | 101 (40.2) | 10 (12.8) | 91 (52.6) | <0.001 |
| ^a^ Comparison between no renal replacement therapy and renal replacement therapy.  Abbreviations: **APACHE II**, acute physiologic assessment and chronic health evaluation II; **FiO_2_**, fraction of inspired oxygen; **GCS**, Glasgow coma scale; **Hb**, hemoglobin; **Hct**, hematocrit; **HDU**, high dependency unit; **ICU**, intensive care unit; **IQR**, interquartile range; **MBP**, mean blood pressure; **no.**, number; **PaO_2_**, partial pressure of oxygen; **PLT**, platelet; **qSOFA**, quick sequential organ failure assessment; **RR**, respiratory rate; **SBP**, systolic blood pressure; **SD**, standard deviation; **SIRS**, systemic inflammatory response syndrome; **SOFA**, sequential organ failure assessment; **WBC**, white blood cell. | | | | |

**Table S9**. Clinical and laboratory characteristics and severity of illness according to methods of renal replacement therapy for patients with sepsis

| Variable | All cases  n=251 | No renal replacement therapy  n=150 | Renal replacement therapy  n=101 | P^a^ |
| --- | --- | --- | --- | --- |
| **Vital signs** (on admission into ICU) | | | | |
| GCS, median (IQR) | 13 (9-15) | 13 (9.5-15) | 13 (9-15) | 0.366 |
| HR (beats per min), median (IQR) | 110 (95.25-125.75) | 110 (93.5-120) | 111 (98.5-129.5) | 0.115 |
| Temperature (^o^C), mean (SD) | 37.79 (1.01) | 37.9 (1.06) | 37.63 (0.92) | 0.098 |
| MBP (mmHg), mean(SD) | 75.82 (22.08) | 79.22 (19.83) | 70.89 (24.38) | <0.001 |
| SBP (mmHg), mean (SD) | 106.45 (29.96) | 110.95 (28.16) | 99.94 (31.56) | 0.002 |
| RR (breaths per min), median (IQR) | 25 (22-30) | 25 (22-30) | 26 (22-30) | 0.528 |
| **Blood investigations** | | | | |
| Total WBC (x10^9^/L), mean (SD) | 15.73 (9.20) | 15.52 (8.72) | 15.82 (9.72) | 0.966 |
| PLT (x10^9^/L), mean (SD) | 185.98 (137.85) | 194.29 (132.94) | 172.23 (144.45) | 0.067 |
| Hb (g/dL), mean (SD) | 11.14 (2.59) | 11.27 (2.62) | 11.00 (2.52) | 0.377 |
| Hct (%), mean (SD) | 34.31 (7.75) | 34.51 (7.78) | 34.13 (7.69) | 0.527 |
| K^+^ (mmol/L), mean (SD) | 3.89 (0.79) | 3.77 (0.72) | 4.05 (0.84) | 0.015 |
| Na^+^ (mmol/L), mean (SD) | 136.05 (8.24) | 135.71 (8.94) | 136.58 (7.13) | 0.207 |
| Creatinine (µmol/L), mean (SD) | 187.85 (151.92) | 139.54 (99.51) | 260.09 (185.42) | <0.001 |
| Bilirubin (µmol/l), mean (SD) | 32.80 (61.49) | 31.70 (72.16) | 34.47 (42.93) | 0.100 |
| pH, mean (SD) | 7.37 (0.50) | 7.42 (0.64) | 7.31 (0.14) | <0.001 |
| PaO_2_ (mmHg), mean (SD) | 116.17 (74.28) | 113.45 (66.36) | 120.31 (84.82) | 0.886 |
| FiO_2_, mean (SD) | 0.50 (0.22) | 0.46 (0.19) | 0.54 (0.24) | 0.010 |
| PaO_2_/FiO_2_ ratio, mean (SD) | 262.48 (149.58) | 267.50 (141.14) | 253.63 (160.96) | 0.196 |
| **Severity of illness scores** | | | | |
| SOFA, median (IQR) | 7 (4.75-10) | 6 (4-8) | 10 (7-12.5) | <0.001 |
| APACHE II, median (IQR) | 18 (13-24) | 15 (12-21.25) | 22 (16-27) | <0.001 |
| Septic Shock | 74 (29.5) | 31 (20.7) | 43 (42.6) | <0.001 |
| **Life-sustaining treatments during ICU stay** | | | | |
| Respiratory support, no. (%) and median (IQR), days |  |  |  |  |
| Mechanical ventilation | 173 (68.9) | 85 (56.7) | 88 (87.1) | <0.001 |
| Duration of mechanical ventilation | 8 (4-15) | 8.5 (3.-12.25) | 8 (4.5-17.5) | 0.267 |
| Non-invasive ventilation | 20 (8.0) | 7 (4.7) | 13 (12.9) | 0.019 |
| Duration of non-invasive ventilation | 2 (2-3.75) | 2 (2-3) | 2 (1.5-4.5) | 0.967 |
| High-flow nasal oxygen | 38 (15.1) | 30 (20.0) | 8 (7.9) | 0.009 |
| Duration of high-flow nasal oxygen | 2 (1-3) | 2 (1-3) | 2.5 (1.25-4) | 0.515 |
| **Outcome** | | | | |
| Length of stay, median days (IQR) |  |  |  |  |
| ICU | 10 (6-18) | 9 (5-15) | 13 (6-21) | 0.029 |
| Hospital | 16 (10-25) | 16 (11-23) | 15 (8-27) | 0.947 |
| Mortality, no. (%) |  |  |  |  |
| ICU | 84 (33.5) | 31 (20.7) | 53 (52.5) | <0.001 |
| Hospital | 101 (40.2) | 43 (28.7) | 58 (57.4) | <0.001 |
| ^a^ Comparison between no renal replacement therapy and renal replacement therapy.  Abbreviations: **APACHE II**, acute physiologic assessment and chronic health evaluation II; **FiO_2_**, fraction of inspired oxygen; **GCS**, Glasgow coma scale; **Hb**, hemoglobin; **Hct**, hematocrit; **HDU**, high dependency unit; **ICU**, intensive care unit; **IQR**, interquartile range; **MBP**, mean blood pressure; **no.**, number; **PaO_2_**, partial pressure of oxygen; **PLT**, platelet; **qSOFA**, quick sequential organ failure assessment; **RR**, respiratory rate; **SBP**, systolic blood pressure; **SD**, standard deviation; **SIRS**, systemic inflammatory response syndrome; **SOFA**, sequential organ failure assessment; **WBC**, white blood cell. | | | | |

**Table S10.** Factors associated with hospital mortality in patients with sepsis: bivariate regression analyses

| Factor | Frequency | OR | 95.0% CI for OR | | P |
| --- | --- | --- | --- | --- | --- |
|  |  |  | Lower | Upper |  |
| Participating hospital |  |  |  |  |  |
| 115 People's | 25 | - | - | - | 0.004 |
| Bach Mai | 26 | 0.271 | 0.082 | 0.898 | 0.033 |
| Bai Chay | 14 | 0.126 | 0.029 | 0.554 | 0.006 |
| Can Tho | 7 | 1.895 | 0.189 | 19.039 | 0.587 |
| Cho Ray | 41 | 0.366 | 0.121 | 1.103 | 0.074 |
| Da Nang | 12 | 0.316 | 0.074 | 1.356 | 0.121 |
| Dong Da | 9 | 0.158 | 0.030 | 0.832 | 0.030 |
| Hanoi Medical University | 12 | 0.316 | 0.074 | 1.356 | 0.121 |
| Hue | 39 | 0.158 | 0.051 | 0.491 | 0.001 |
| Saint Paul | 9 | 0.000 | 0.000 | - | 0.999 |
| Thai Nguyen | 2 | 0.316 | 0.017 | 5.854 | 0.439 |
| Thanh Nhan | 1 | 510149957.2 | 0.000 | - | >0.999 |
| Vietnam–Czechoslovakia Friendship | 48 | 0.063 | 0.019 | 0.208 | <0.001 |
| Vinmec Times City International | 7 | 0.000 | 0.000 | - | 0.999 |
| **Hospital characteristics** |  |  |  |  |  |
| University affiliation |  |  |  |  |  |
| No | 153 | - | - | - | - |
| Yes | 99 | 2.520 | 1.495 | 4.248 | 0.001 |
| **ICU characteristics** |  |  |  |  |  |
| Type of ICU |  |  |  |  |  |
| Medical | 110 | - | - | - | - |
| Mixed | 142 | 1.151 | 0.692 | 1.915 | 0.589 |
| Nurse to patient ratio |  |  |  |  |  |
| 1 nurse : 4 or more patients | 58 | - | - | - | 0.946 |
| 1 or more nurses : 1 patient | 7 | 0.000 | 0.000 | - | 0.999 |
| 1 nurse : 2 patients | 187 | 0.904 | 0.498 | 1.640 | 0.739 |
| Intensivist to patient ratio |  |  |  |  |  |
| 1 intensivist : 5 or fewer patients | 165 | - | - | - | 0.449 |
| 1 intensivist : 6 to 8 patients | 75 | 0.738 | 0.419 | 1.302 | 0.294 |
| 1 intensivist : 12 or more patients | 12 | 1.391 | 0.430 | 4.497 | 0.581 |
| Training programme in ICU |  |  |  |  |  |
| No | 50 | - | - | - | - |
| Yes | 202 | 0.445 | 0.237 | 0.833 | 0.011 |
| **Baseline characteristics** |  |  |  |  |  |
| Age (year) | 252 | 1.001 | 0.986 | 1.016 | 0.921 |
| Age (year) group |  |  |  |  |  |
| < 20 | 3 | - | - | - | 0.863 |
| 20 - 39 | 19 | 1.800 | 0.139 | 23.374 | 0.653 |
| 40 - 59 | 74 | 1.442 | 0.125 | 16.617 | 0.769 |
| ≥ 60 | 156 | 1.250 | 0.111 | 14.086 | 0.857 |
| Sex (male) | 162 | 1.345 | 0.790 | 2.290 | 0.275 |
| Collection batch |  |  |  |  |  |
| Collection 1 (Jan) | 80 | - | - | - | 0.008 |
| Collection 2 (April) | 62 | 3.418 | 1.694 | 6.896 | 0.001 |
| Collection 3 (July) | 54 | 1.812 | 0.872 | 3.768 | 0.111 |
| Collection 4 (Oct) | 56 | 1.706 | 0.825 | 3.529 | 0.150 |
| Admission type |  |  |  |  |  |
| Medical | 236 | - | - | - | 0.355 |
| Elective surgical | 2 | 0.000 | 0.000 | - | 0.999 |
| Unscheduled surgical | 14 | 0.384 | 0.104 | 1.413 | 0.150 |
| Admission source |  |  |  |  |  |
| Emergency department | 138 | - | - | - | 0.714 |
| Operating room | 4 | 0.569 | 0.058 | 5.612 | 0.629 |
| General wards | 56 | 1.189 | 0.630 | 2.243 | 0.593 |
| Other ICUs or HDU | 16 | 1.024 | 0.351 | 2.983 | 0.966 |
| Inter-hospital transfer | 37 | 1.801 | 0.866 | 3.742 | 0.115 |
| Others | 1 | 2755810063 | 0.000 | - | >0.999 |
| Comorbidities |  |  |  |  |  |
| Cardiovascular disease | 78 | 1.551 | 0.903 | 2.664 | 0.112 |
| Chronic lung disease | 30 | 0.996 | 0.458 | 2.169 | 0.992 |
| Chronic neurological disease | 36 | 0.378 | 0.165 | 0.867 | 0.022 |
| Chronic kidney disease | 23 | 0.957 | 0.398 | 2.304 | 0.922 |
| Peptic ulcer disease | 9 | 1.204 | 0.315 | 4.597 | 0.786 |
| Chronic liver disease | 27 | 1.446 | 0.649 | 3.220 | 0.367 |
| Diabetes mellitus | 67 | 1.012 | 0.573 | 1.790 | 0.966 |
| Connective tissue disease | 3 | 0.745 | 0.067 | 8.326 | 0.811 |
| Immunosuppression | 10 | 0.630 | 0.159 | 2.495 | 0.510 |
| Haematological malignancies | 5 | 0.997 | 0.164 | 6.073 | 0.997 |
| Solid malignant tumours | 12 | 1.526 | 0.478 | 4.873 | 0.475 |
| **Vital signs** |  |  |  |  |  |
| GCS | 251 | 0.849 | 0.786 | 0.918 | <0.001 |
| HR (beats per min) | 252 | 1.010 | 0.997 | 1.022 | 0.140 |
| Temperature (^o^C) | 252 | 0.975 | 0.760 | 1.252 | 0.845 |
| MBP (mmHg) | 252 | 0.978 | 0.965 | 0.991 | 0.001 |
| SBP (mmHg) | 252 | 0.986 | 0.977 | 0.995 | 0.002 |
| RR (breaths per min) | 252 | 0.987 | 0.944 | 1.031 | 0.555 |
| **Blood investigations** |  |  |  |  |  |
| Total WBC (x10^9^/L) | 252 | 1.003 | 0.976 | 1.031 | 0.833 |
| PLT (x10^9^/L) | 252 | 0.998 | 0.996 | 1.000 | 0.040 |
| Hb (g/dL) | 251 | 0.922 | 0.835 | 1.018 | 0.109 |
| Hct (%) | 252 | 0.968 | 0.936 | 1.001 | 0.057 |
| K^+^ (mmol/L) | 252 | 0.961 | 0.697 | 1.324 | 0.805 |
| Na^+^ (mmol/L) | 252 | 1.016 | 0.985 | 1.048 | 0.313 |
| Creatinine (µmol/L) | 252 | 1.000 | 0.999 | 1.002 | 0.828 |
| Bilirubin (µmol/l) | 232 | 1.001 | 0.996 | 1.005 | 0.752 |
| pH | 248 | 0.045 | 0.005 | 0.389 | 0.005 |
| PaO_2_ (mmHg) | 244 | 1.003 | 0.999 | 1.006 | 0.142 |
| FiO_2_ | 245 | 26.892 | 7.081 | 102.133 | <0.001 |
| PaO_2_/FiO_2_ ratio | 243 | 0.998 | 0.996 | 1.000 | 0.020 |
| **Severity of illness scores** |  |  |  |  |  |
| qSOFA | 252 | 1.697 | 1.203 | 2.393 | 0.003 |
| qSOFA |  |  |  |  |  |
| 0 - 1 | 69 | - | - | - | - |
| 2 - 3 | 183 | 1.775 | 0.984 | 3.203 | 0.057 |
| SIRS | 252 | 1.004 | 0.776 | 1.300 | 0.974 |
| SOFA | 250 | 1.219 | 1.130 | 1.315 | <0.001 |
| SOFA |  |  |  |  |  |
| 2 - 3 | 46 | - | - | - | <0.001 |
| 4 - 5 | 36 | 0.725 | 0.263 | 2.000 | 0.535 |
| 6 - 7 | 58 | 0.886 | 0.371 | 2.114 | 0.784 |
| 8 - 9 | 32 | 2.538 | 0.987 | 6.528 | 0.053 |
| 10 - 11 | 38 | 2.285 | 0.925 | 5.642 | 0.073 |
| 12 - 14 | 29 | 6.663 | 2.363 | 18.793 | <0.001 |
| > 14 | 11 | 25.385 | 2.947 | 218.685 | 0.003 |
| SOFA |  |  |  |  |  |
| 0 - 6 | 109 | - | - | - | <0.001 |
| 7 - 9 | 63 | 1.780 | 0.915 | 3.464 | 0.090 |
| 10 - 12 | 38 | 2.604 | 1.208 | 5.613 | 0.015 |
| 13 - 14 | 29 | 7.594 | 3.024 | 19.069 | <0.001 |
| > 14 | 11 | 28.929 | 3.542 | 236.248 | 0.002 |
| SOFA |  |  |  |  |  |
| 0 - 3 | 46 | - | - | - | <0.001 |
| 4 - 7 | 94 | 0.822 | 0.371 | 1.822 | 0.630 |
| 8 - 9 | 32 | 2.538 | 0.987 | 6.528 | 0.053 |
| 10 - 11 | 38 | 2.285 | 0.925 | 5.642 | 0.073 |
| ≥ 12 | 40 | 8.744 | 3.278 | 23.324 | <0.001 |
| APACHE II | 252 | 1.088 | 1.050 | 1.127 | <0.001 |
| APACHE II |  |  |  |  |  |
| 0 - 4 | 3 | - | - | - | <0.001 |
| 5 - 9 | 22 | 605807962.9 | 0.000 | - | 0.999 |
| 10 - 14 | 61 | 355407338.2 | 0.000 | - | 0.999 |
| 15 - 19 | 52 | 930129397.5 | 0.000 | - | 0.999 |
| 20 - 24 | 58 | 1854819442 | 0.000 | - | 0.999 |
| 25 - 29 | 28 | 3410474458 | 0.000 | - | 0.999 |
| 30 - 34 | 19 | 1453939111 | 0.000 | - | 0.999 |
| > 34 | 9 | 3230975802 | 0.000 | - | 0.999 |
| APACHE II |  |  |  |  | <0.001 |
| 0 - 9 | 25 | - | - | - |  |
| 10 - 14 | 61 | 0.697 | 0.226 | 2.149 | 0.529 |
| 15 - 19 | 52 | 1.823 | 0.621 | 5.355 | 0.275 |
| 20 - 24 | 58 | 3.636 | 1.269 | 10.420 | 0.016 |
| 25 - 29 | 28 | 6.685 | 1.987 | 22.487 | 0.002 |
| ≥ 30 | 28 | 3.654 | 1.122 | 11.899 | 0.031 |
| **Site of Infection** |  |  |  |  |  |
| Respiratory | 143 | 1.283 | 0.769 | 2.140 | 0.339 |
| Urinary tract | 37 | 0.300 | 0.126 | 0.714 | 0.006 |
| Abdominal | 61 | 1.256 | 0.701 | 2.249 | 0.444 |
| Neurological | 12 | 0.737 | 0.216 | 2.516 | 0.626 |
| Bones or joints | 2 | 0.000 | 0.000 | - | 0.999 |
| Skin or cutaneous sites | 19 | 2.774 | 1.053 | 7.309 | 0.039 |
| Intravascular catheter | 1 | 0.000 | 0.000 | - | 1.000 |
| Infective endocarditis | 1 | 2439367045 | 0.000 | - | >0.999 |
| Primary bacteraemia | 7 | 0.590 | 0.112 | 3.101 | 0.533 |
| Systemic | 6 | 0.742 | 0.133 | 4.131 | 0.734 |
| **Microbiology** |  |  |  |  |  |
| Pathogens detection |  |  |  |  |  |
| No pathogens detected | 67 | 0.546 | 0.300 | 0.994 | 0.048 |
| Gram negative bacteria | 156 | 1.475 | 0.871 | 2.498 | 0.148 |
| Gram positive bacteria | 34 | 0.791 | 0.372 | 1.679 | 0.541 |
| Fungi | 7 | 1.125 | 0.246 | 5.137 | 0.879 |
| Viruses | 2 | 2464007117 | 0.000 | - | 0.999 |
| Other pathogens | 4 | 0.493 | 0.051 | 4.810 | 0.543 |
| **Completion of sepsis bundle elements** |  |  |  |  |  |
| Timing of antibiotics administration |  |  |  |  |  |
| 0-60 minutes | 173 | - | - | - | 0.367 |
| 61-120 minutes | 21 | 1.048 | 0.412 | 2.665 | 0.921 |
| 121-180 minutes | 14 | 0.464 | 0.125 | 1.727 | 0.252 |
| >180 minutes | 17 | 1.916 | 0.704 | 5.214 | 0.203 |
| Timing of obtaining blood cultures |  |  |  |  |  |
| 0-60 minutes | 135 | - | - | - | 0.939 |
| 61-120 minutes | 14 | 1.328 | 0.441 | 3.995 | 0.614 |
| 121-180 minutes | 10 | 0.885 | 0.239 | 3.281 | 0.855 |
| >180 minutes | 38 | 0.774 | 0.369 | 1.626 | 0.500 |
| Timing of obtaining lactate measurement |  |  |  |  |  |
| 0-60 minutes | 141 | - | - | - | 0.763 |
| 61-120 minutes | 10 | 1.012 | 0.273 | 3.748 | 0.986 |
| 121-180 minutes | 6 | 0.304 | 0.035 | 2.668 | 0.282 |
| >180 minutes | 41 | 0.971 | 0.476 | 1.981 | 0.936 |
| Completion of the sepsis bundle within 1 hour | 87 | 0.978 | 0.571 | 1.675 | 0.936 |
| Completion of the administration of antibiotics within 1 hour | 173 | 0.701 | 0.397 | 1.237 | 0.220 |
| Permutations of the completed elements within 1 hour |  |  |  |  |  |
| No elements completed | 20 | - | - | - | 0.228 |
| Antibiotics only | 44 | 0.700 | 0.234 | 2.096 | 0.524 |
| Blood cultures only | 13 | 0.667 | 0.152 | 2.926 | 0.591 |
| Lactate only | 23 | 1.636 | 0.487 | 5.500 | 0.426 |
| Antibiotics + Lactate | 17 | 0.321 | 0.069 | 1.491 | 0.147 |
| Antibiotics + Blood cultures | 25 | 1.625 | 0.491 | 5.341 | 0.424 |
| Blood cultures + Lactate | 12 | 2.100 | 0.490 | 8.998 | 0.318 |
| Antibiotics + Blood cultures + Lactate | 87 | 0.962 | 0.357 | 2.597 | 0.939 |
| Completion of the sepsis bundle within 3 hours | 108 | 0.961 | 0.571 | 1.615 | 0.879 |
| Completion of the administration of antibiotics within 3 hours | 205 | 0.403 | 0.196 | 0.830 | 0.014 |
| Permutation of the completed elements of 3-hour sepsis bundle |  |  |  |  |  |
| No elements completed | 8 | - | - | - | 0.049 |
| Antibiotics only | 37 | 0.193 | 0.038 | 0.971 | 0.046 |
| Blood cultures only | 5 | 0.900 | 0.091 | 8.899 | 0.928 |
| Lactate only | 16 | 0.600 | 0.106 | 3.400 | 0.564 |
| Antibiotics + Lactate | 24 | 0.158 | 0.028 | 0.897 | 0.037 |
| Antibiotics + Blood cultures | 36 | 0.600 | 0.124 | 2.894 | 0.525 |
| Blood cultures + Lactate | 7 | 0.500 | 0.170 | 13.225 | 0.715 |
| Antibiotics + Blood cultures + Lactate | 108 | 0.382 | 0.087 | 1.682 | 0.203 |
| **Life-sustaining treatments during ICU stay** |  |  |  |  |  |
| Respiratory support |  |  |  |  |  |
| Mechanical ventilation | 173 | 7.546 | 3.645 | 15.625 | <0.001 |
| Duration of mechanical ventilation | 167 | 0.998 | 0.969 | 1.028 | 0.909 |
| Non-invasive ventilation | 20 | 0.785 | 0.302 | 2.041 | 0.619 |
| Duration of non-invasive ventilation | 20 | 4.738 | 1.076 | 20.863 | 0.040 |
| High-flow nasal oxygen | 38 | 0.408 | 0.184 | 0.904 | 0.027 |
| Duration of high-flow nasal oxygen | 33 | 1.154 | 0.862 | 1.546 | 0.336 |
| Additional ICU support |  |  |  |  |  |
| Vasopressors/inotropes | 163 | 3.408 | 1.899 | 6.116 | <0.001 |
| Renal replacement therapy | 101 | 3.356 | 1.976 | 5.702 | <0.001 |
| Red blood cell transfusion | 93 | 1.708 | 1.014 | 2.876 | 0.044 |
| Platelet transfusion | 50 | 2.746 | 1.455 | 5.185 | 0.002 |
| Fresh frozen plasma transfusion | 58 | 1.841 | 1.018 | 3.329 | 0.043 |
| Surgical source control | 25 | 0.435 | 0.168 | 1.132 | 0.088 |
| Non-surgical source control | 78 | 0.554 | 0.314 | 0.977 | 0.041 |

**Table S11.** Factors associated with hospital mortality in patients with sepsis: multivariate logistic regression analyses (backward elimination)

| Step | Factor | Unit | OR | 95.0% CI for OR | | P |
| --- | --- | --- | --- | --- | --- | --- |
|  |  |  |  | Lower | Upper |  |
| 1 | University affiliation |  |  |  |  |  |
|  | No | % | - | - | - | - |
|  | Yes | % | 1.642 | 0.688 | 3.918 | 0.264 |
|  | Training programme in ICU |  |  |  |  |  |
|  | No | % | - | - | - | - |
|  | Yes | % | 0.241 | 0.080 | 0.719 | 0.011 |
|  | Comorbidities |  |  |  |  |  |
|  | Cardiovascular disease | % | 2.255 | 0.960 | 5.296 | 0.062 |
|  | Chronic neurological disease | % | 0.207 | 0.055 | 0.779 | 0.020 |
|  | qSOFA |  |  |  |  |  |
|  | 0 - 1 | % | - | - | - | - |
|  | 2 - 3 | % | 0.903 | 0.363 | 2.242 | 0.825 |
|  | SOFA |  |  |  |  |  |
|  | 0 - 3 | % | - | - | - | 0.127 |
|  | 4-7 | % | 0.648 | 0.191 | 2.198 | 0.486 |
|  | 8-9 | % | 1.666 | 0.372 | 7.469 | 0.505 |
|  | 10 - 11 | % | 1.035 | 0.242 | 4.417 | 0.963 |
|  | ≥12 | % | 3.645 | 0.717 | 18.541 | 0.119 |
|  | APACHE II |  |  |  |  |  |
|  | 0 - 9 | % | - | - | - | 0.294 |
|  | 10 - 14 | % | 0.327 | 0.067 | 1.580 | 0.164 |
|  | 15 - 19 | % | 0.537 | 0.117 | 2.467 | 0.424 |
|  | 20 - 24 | % | 0.974 | 0.214 | 4.427 | 0.973 |
|  | 25 - 29 | % | 1.632 | 0.251 | 10.620 | 0.608 |
|  | ≥ 30 | % | 0.622 | 0.107 | 3.606 | 0.597 |
|  | Site of Infection |  |  |  |  |  |
|  | Urinary tract | % | 0.224 | 0.057 | 0.875 | 0.031 |
|  | Skin or cutaneous sites | % | 1.969 | 0.535 | 7.249 | 0.308 |
|  | Pathogens detection |  |  |  |  |  |
|  | No pathogens detected | % | 1.034 | 0.256 | 4.175 | 0.963 |
|  | Gram negative bacteria | % | 1.487 | 0.418 | 5.289 | 0.540 |
|  | Completion of the sepsis bundle within 1 hour | % | 1.636 | 0.238 | 11.231 | 0.617 |
|  | Completion of the administration of antibiotics within 1 hour | % | 0.975 | 0.241 | 3.948 | 0.971 |
|  | Completion of the sepsis bundle within 3 hours | % | 1.708 | 0.283 | 10.298 | 0.559 |
|  | Completion of the administration of antibiotics within 3 hours | % | 0.163 | 0.034 | 0.771 | 0.022 |
|  | Respiratory support |  |  |  |  |  |
|  | Mechanical ventilation | % | 4.797 | 1.598 | 14.398 | 0.005 |
|  | High-flow nasal oxygen | % | 0.718 | 0.223 | 2.312 | 0.579 |
|  | Additional ICU support |  |  |  |  |  |
|  | Vasopressors/inotropes | % | 1.545 | 0.551 | 4.331 | 0.408 |
|  | Renal replacement therapy | % | 1.387 | 0.603 | 3.194 | 0.441 |
|  | Red blood cell transfusion | % | 1.063 | 0.453 | 2.495 | 0.888 |
|  | Platelet transfusion | % | 0.935 | 0.340 | 2.573 | 0.896 |
|  | Fresh frozen plasma transfusion | % | 1.290 | 0.486 | 3.420 | 0.609 |
|  | Surgical source control | % | 0.259 | 0.069 | 0.967 | 0.044 |
|  | Non-surgical source control | % | 0.424 | 0.184 | 0.975 | 0.044 |
|  | Constant |  | 1.634 |  |  | 0.663 |
| 2 | University affiliation |  |  |  |  |  |
|  | No | % | - | - | - | - |
|  | Yes | % | 1.646 | 0.696 | 3.892 | 0.257 |
|  | Training programme in ICU |  |  |  |  |  |
|  | No | % | - | - | - | - |
|  | Yes | % | 0.240 | 0.081 | 0.713 | 0.010 |
|  | Comorbidities |  |  |  |  |  |
|  | Cardiovascular disease | % | 2.258 | 0.964 | 5.288 | 0.061 |
|  | Chronic neurological disease | % | 0.207 | 0.055 | 0.779 | 0.020 |
|  | qSOFA |  |  |  |  |  |
|  | 0 - 1 | % | - | - | - | - |
|  | 2 - 3 | % | 0.904 | 0.365 | 2.238 | 0.827 |
|  | SOFA |  |  |  |  |  |
|  | 0 - 3 | % | - | - | - | 0.126 |
|  | 4-7 | % | 0.647 | 0.191 | 2.198 | 0.486 |
|  | 8-9 | % | 1.664 | 0.372 | 7.446 | 0.506 |
|  | 10 - 11 | % | 1.034 | 0.242 | 4.416 | 0.964 |
|  | ≥12 | % | 3.639 | 0.718 | 18.453 | 0.119 |
|  | APACHE II |  |  |  |  |  |
|  | 0 - 9 | % | - | - | - | 0.290 |
|  | 10 - 14 | % | 0.327 | 0.068 | 1.579 | 0.164 |
|  | 15 - 19 | % | 0.537 | 0.117 | 2.466 | 0.424 |
|  | 20 - 24 | % | 0.974 | 0.214 | 4.427 | 0.973 |
|  | 25 - 29 | % | 1.628 | 0.252 | 10.522 | 0.609 |
|  | ≥ 30 | % | 0.621 | 0.108 | 3.588 | 0.595 |
|  | Site of Infection |  |  |  |  |  |
|  | Urinary tract | % | 0.224 | 0.057 | 0.874 | 0.031 |
|  | Skin or cutaneous sites | % | 1.969 | 0.535 | 7.249 | 0.308 |
|  | Pathogens detection |  |  |  |  |  |
|  | No pathogens detected | % | 1.033 | 0.256 | 4.165 | 0.964 |
|  | Gram negative bacteria | % | 1.485 | 0.419 | 5.271 | 0.540 |
|  | Completion of the sepsis bundle within 1 hour | % | 1.597 | 0.384 | 6.647 | 0.520 |
|  | Completion of the sepsis bundle within 3 hours | % | 1.743 | 0.412 | 7.373 | 0.451 |
|  | Completion of the administration of antibiotics within 3 hours | % | 0.160 | 0.052 | 0.489 | 0.001 |
|  | Respiratory support |  |  |  |  |  |
|  | Mechanical ventilation | % | 4.792 | 1.599 | 14.364 | 0.005 |
|  | High-flow nasal oxygen | % | .718 | 0.223 | 2.313 | 0.579 |
|  | Additional ICU support |  |  |  |  |  |
|  | Vasopressors/inotropes | % | 1.548 | 0.554 | 4.321 | 0.404 |
|  | Renal replacement therapy | % | 1.389 | 0.604 | 3.191 | 0.439 |
|  | Red blood cell transfusion | % | 1.062 | 0.453 | 2.489 | 0.889 |
|  | Platelet transfusion | % | .932 | 0.343 | 2.535 | 0.890 |
|  | Fresh frozen plasma transfusion | % | 1.294 | 0.498 | 3.367 | 0.597 |
|  | Surgical source control | % | 0.259 | 0.069 | 0.967 | 0.044 |
|  | Non-surgical source control | % | 0.423 | 0.185 | 0.968 | 0.042 |
|  | Constant |  | 1.633 |  |  | 0.663 |
| 3 | University affiliation |  |  |  |  |  |
|  | No | % | - | - | - | - |
|  | Yes | % | 1.648 | 0.698 | 3.891 | 0.255 |
|  | Training programme in ICU |  |  |  |  |  |
|  | No | % | - | - | - | - |
|  | Yes | % | 0.240 | 0.081 | 0.713 | 0.010 |
|  | Comorbidities |  |  |  |  |  |
|  | Cardiovascular disease | % | 2.248 | 0.980 | 5.157 | 0.056 |
|  | Chronic neurological disease | % | 0.208 | 0.055 | 0.779 | 0.020 |
|  | qSOFA |  |  |  |  |  |
|  | 0 - 1 | % | - | - | - | - |
|  | 2 - 3 | % | 0.907 | 0.372 | 2.215 | 0.831 |
|  | SOFA |  |  |  |  |  |
|  | 0 - 3 | % | - | - | - | 0.124 |
|  | 4-7 | % | 0.646 | 0.191 | 2.187 | 0.483 |
|  | 8-9 | % | 1.660 | 0.372 | 7.400 | 0.506 |
|  | 10 - 11 | % | 1.029 | 0.246 | 4.306 | 0.969 |
|  | ≥12 | % | 3.635 | 0.717 | 18.426 | 0.119 |
|  | APACHE II |  |  |  |  |  |
|  | 0 - 9 | % | - | - | - | 0.290 |
|  | 10 - 14 | % | 0.327 | 0.068 | 1.579 | 0.164 |
|  | 15 - 19 | % | 0.539 | 0.118 | 2.462 | 0.425 |
|  | 20 - 24 | % | 0.974 | 0.214 | 4.427 | 0.973 |
|  | 25 - 29 | % | 1.628 | 0.252 | 10.527 | 0.609 |
|  | ≥ 30 | % | 0.621 | 0.108 | 3.589 | 0.595 |
|  | Site of Infection |  |  |  |  |  |
|  | Urinary tract | % | 0.224 | 0.057 | .874 | 0.031 |
|  | Skin or cutaneous sites | % | 1.950 | 0.566 | 6.715 | 0.290 |
|  | Pathogens detection |  |  |  |  |  |
|  | Gram negative bacteria | % | 1.452 | 0.648 | 3.254 | 0.364 |
|  | Completion of the sepsis bundle within 1 hour | % | 1.596 | 0.384 | 6.642 | 0.520 |
|  | Completion of the sepsis bundle within 3 hours | % | 1.740 | 0.412 | 7.357 | 0.451 |
|  | Completion of the administration of antibiotics within 3 hours | % | 0.160 | 0.052 | 0.488 | 0.001 |
|  | Respiratory support |  |  |  |  |  |
|  | Mechanical ventilation | % | 4.785 | 1.599 | 14.318 | 0.005 |
|  | High-flow nasal oxygen | % | 0.719 | 0.223 | 2.313 | 0.580 |
|  | Additional ICU support |  |  |  |  |  |
|  | Vasopressors/inotropes | % | 1.553 | 0.563 | 4.286 | 0.395 |
|  | Renal replacement therapy | % | 1.389 | 0.605 | 3.192 | 0.439 |
|  | Red blood cell transfusion | % | 1.059 | 0.456 | 2.462 | 0.893 |
|  | Platelet transfusion | % | 0.932 | 0.343 | 2.535 | 0.890 |
|  | Fresh frozen plasma transfusion | % | 1.294 | 0.497 | 3.366 | 0.597 |
|  | Surgical source control | % | 0.260 | 0.070 | 0.966 | 0.044 |
|  | Non-surgical source control | % | 0.423 | 0.185 | 0.967 | 0.041 |
|  | Constant |  | 1.672 |  |  | 0.607 |
| 4 | University affiliation |  |  |  |  |  |
|  | No | % | - | - | - | - |
|  | Yes | % | 1.641 | 0.696 | 3.870 | 0.258 |
|  | Training programme in ICU |  |  |  |  |  |
|  | No | % | - | - | - | - |
|  | Yes | % | 0.241 | 0.081 | 0.714 | 0.010 |
|  | Comorbidities |  |  |  |  |  |
|  | Cardiovascular disease | % | 2.242 | 0.978 | 5.139 | 0.056 |
|  | Chronic neurological disease | % | 0.209 | 0.056 | 0.781 | 0.020 |
|  | qSOFA |  |  |  |  |  |
|  | 0 - 1 | % | - | - | - | - |
|  | 2 - 3 | % | 0.909 | 0.373 | 2.217 | 0.834 |
|  | SOFA |  |  |  |  |  |
|  | 0 - 3 | % | - | - | - | 0.125 |
|  | 4-7 | % | 0.650 | 0.193 | 2.191 | 0.487 |
|  | 8-9 | % | 1.656 | 0.372 | 7.379 | 0.508 |
|  | 10 - 11 | % | 1.033 | 0.247 | 4.316 | 0.965 |
|  | ≥12 | % | 3.650 | 0.721 | 18.481 | 0.118 |
|  | APACHE II |  |  |  |  |  |
|  | 0 - 9 | % | - | - | - | 0.291 |
|  | 10 - 14 | % | 0.329 | 0.068 | 1.583 | 0.165 |
|  | 15 - 19 | % | 0.537 | 0.118 | 2.453 | 0.423 |
|  | 20 - 24 | % | 0.973 | 0.214 | 4.421 | 0.972 |
|  | 25 - 29 | % | 1.620 | 0.251 | 10.470 | 0.612 |
|  | ≥ 30 | % | 0.620 | 0.107 | 3.585 | 0.594 |
|  | Site of Infection |  |  |  |  |  |
|  | Urinary tract | % | .223 | 0.057 | .868 | 0.030 |
|  | Skin or cutaneous sites | % | 1.953 | 0.567 | 6.726 | 0.289 |
|  | Pathogens detection |  |  |  |  |  |
|  | Gram negative bacteria | % | 1.465 | 0.660 | 3.251 | 0.348 |
|  | Completion of the sepsis bundle within 1 hour | % | 1.595 | 0.383 | 6.635 | 0.521 |
|  | Completion of the sepsis bundle within 3 hours | % | 1.739 | 0.411 | 7.355 | 0.452 |
|  | Completion of the administration of antibiotics within 3 hours | % | 0.160 | 0.052 | 0.488 | 0.001 |
|  | Respiratory support |  |  |  |  |  |
|  | Mechanical ventilation | % | 4.796 | 1.605 | 14.334 | 0.005 |
|  | High-flow nasal oxygen | % | 0.716 | 0.223 | 2.298 | 0.574 |
|  | Additional ICU support |  |  |  |  |  |
|  | Vasopressors/inotropes | % | 1.561 | 0.568 | 4.292 | 0.388 |
|  | Renal replacement therapy | % | 1.407 | 0.625 | 3.167 | 0.410 |
|  | Platelet transfusion | % | 0.947 | 0.358 | 2.506 | 0.913 |
|  | Fresh frozen plasma transfusion | % | 1.307 | 0.508 | 3.360 | 0.578 |
|  | Surgical source control | % | 0.262 | 0.071 | 0.968 | 0.045 |
|  | Non-surgical source control | % | 0.424 | 0.186 | 0.969 | 0.042 |
|  | Constant |  | 1.666 |  |  | 0.609 |
| 5 | University affiliation |  |  |  |  |  |
|  | No | % | - | - | - | - |
|  | Yes | % | 1.629 | 0.698 | 3.803 | 0.259 |
|  | Training programme in ICU |  |  |  |  |  |
|  | No | % | - | - | - | - |
|  | Yes | % | 0.242 | 0.082 | 0.714 | 0.010 |
|  | Comorbidities |  |  |  |  |  |
|  | Cardiovascular disease | % | 2.239 | 0.978 | 5.127 | 0.056 |
|  | Chronic neurological disease | % | 0.210 | 0.056 | 0.782 | 0.020 |
|  | qSOFA |  |  |  |  |  |
|  | 0 - 1 | % | - | - | - | - |
|  | 2 - 3 | % | 0.906 | 0.372 | 2.205 | 0.828 |
|  | SOFA |  |  |  |  |  |
|  | 0 - 3 | % | - | - | - | 0.124 |
|  | 4-7 | % | 0.650 | 0.193 | 2.190 | 0.487 |
|  | 8-9 | % | 1.651 | 0.371 | 7.344 | 0.510 |
|  | 10 - 11 | % | 1.028 | 0.247 | 4.287 | 0.969 |
|  | ≥12 | % | 3.618 | 0.721 | 18.153 | 0.118 |
|  | APACHE II |  |  |  |  |  |
|  | 0 - 9 | % | - | - | - | 0.280 |
|  | 10 - 14 | % | 0.328 | 0.068 | 1.573 | 0.163 |
|  | 15 - 19 | % | 0.537 | 0.118 | 2.451 | 0.422 |
|  | 20 - 24 | % | 0.973 | 0.215 | 4.414 | 0.972 |
|  | 25 - 29 | % | 1.625 | 0.252 | 10.499 | 0.610 |
|  | ≥ 30 | % | 0.617 | 0.107 | 3.557 | 0.589 |
|  | Site of Infection |  |  |  |  |  |
|  | Urinary tract | % | 0.222 | 0.057 | 0.861 | 0.030 |
|  | Skin or cutaneous sites | % | 1.950 | 0.566 | 6.720 | 0.290 |
|  | Pathogens detection |  |  |  |  |  |
|  | Gram negative bacteria | % | 1.461 | 0.659 | 3.238 | 0.351 |
|  | Completion of the sepsis bundle within 1 hour | % | 1.592 | 0.383 | 6.616 | 0.522 |
|  | Completion of the sepsis bundle within 3 hours | % | 1.737 | 0.411 | 7.338 | 0.453 |
|  | Completion of the administration of antibiotics within 3 hours | % | 0.160 | 0.052 | 0.489 | 0.001 |
|  | Respiratory support |  |  |  |  |  |
|  | Mechanical ventilation | % | 4.774 | 1.604 | 14.205 | 0.005 |
|  | High-flow nasal oxygen | % | 0.715 | 0.223 | 2.296 | 0.573 |
|  | Additional ICU support |  |  |  |  |  |
|  | Vasopressors/inotropes | % | 1.565 | 0.570 | 4.297 | 0.385 |
|  | Renal replacement therapy | % | 1.403 | 0.624 | 3.153 | 0.413 |
|  | Fresh frozen plasma transfusion | % | 1.289 | 0.518 | 3.209 | 0.585 |
|  | Surgical source control | % | 0.261 | 0.071 | 0.962 | 0.044 |
|  | Non-surgical source control | % | 0.426 | 0.188 | 0.969 | 0.042 |
|  | Constant |  | 1.670 |  |  | 0.608 |
| 6 | University affiliation |  |  |  |  |  |
|  | No | % | - | - | - | - |
|  | Yes | % | 1.638 | 0.702 | 3.824 | 0.254 |
|  | Training programme in ICU |  |  |  |  |  |
|  | No | % | - | - | - | - |
|  | Yes | % | 0.243 | 0.082 | 0.717 | 0.010 |
|  | Comorbidities |  |  |  |  |  |
|  | Cardiovascular disease | % | 2.235 | 0.976 | 5.121 | 0.057 |
|  | Chronic neurological disease | % | 0.208 | 0.056 | 0.770 | 0.019 |
|  | SOFA |  |  |  |  |  |
|  | 0 - 3 | % | - | - | - | 0.124 |
|  | 4-7 | % | 0.629 | 0.194 | 2.041 | 0.440 |
|  | 8-9 | % | 1.575 | 0.377 | 6.580 | 0.534 |
|  | 10 - 11 | % | 0.990 | 0.248 | 3.956 | 0.988 |
|  | ≥12 | % | 3.500 | 0.717 | 17.095 | 0.122 |
|  | APACHE II |  |  |  |  |  |
|  | 0 - 9 | % | - | - | - | 0.282 |
|  | 10 - 14 | % | 0.336 | 0.071 | 1.587 | 0.168 |
|  | 15 - 19 | % | 0.547 | 0.121 | 2.473 | 0.434 |
|  | 20 - 24 | % | 0.980 | 0.216 | 4.443 | 0.980 |
|  | 25 - 29 | % | 1.640 | 0.254 | 10.604 | 0.603 |
|  | ≥ 30 | % | 0.620 | 0.108 | 3.571 | 0.592 |
|  | Site of Infection |  |  |  |  |  |
|  | Urinary tract | % | 0.220 | 0.057 | 0.852 | 0.028 |
|  | Skin or cutaneous sites | % | 1.952 | 0.566 | 6.734 | 0.290 |
|  | Pathogens detection |  |  |  |  |  |
|  | Gram negative bacteria | % | 1.486 | 0.682 | 3.240 | 0.319 |
|  | Completion of the sepsis bundle within 1 hour | % | 1.570 | 0.380 | 6.495 | 0.533 |
|  | Completion of the sepsis bundle within 3 hours | % | 1.751 | 0.415 | 7.389 | 0.446 |
|  | Completion of the administration of antibiotics within 3 hours | % | 0.161 | 0.053 | 0.490 | 0.001 |
|  | Respiratory support |  |  |  |  |  |
|  | Mechanical ventilation | % | 4.694 | 1.596 | 13.803 | 0.005 |
|  | High-flow nasal oxygen | % | 0.717 | 0.223 | 2.302 | 0.576 |
|  | Additional ICU support |  |  |  |  |  |
|  | Vasopressors/inotropes | % | 1.559 | 0.568 | 4.275 | 0.389 |
|  | Renal replacement therapy | % | 1.403 | 0.624 | 3.153 | 0.413 |
|  | Fresh frozen plasma transfusion | % | 1.282 | 0.515 | 3.192 | 0.593 |
|  | Surgical source control | % | 0.260 | 0.071 | 0.958 | 0.043 |
|  | Non-surgical source control | % | 0.428 | 0.188 | 0.972 | 0.043 |
|  | Constant |  | 1.585 |  |  | 0.634 |
| 7 | University affiliation |  |  |  |  |  |
|  | No | % | - | - | - | - |
|  | Yes | % | 1.598 | 0.688 | 3.710 | 0.275 |
|  | Training programme in ICU |  |  |  |  |  |
|  | No | % | - | - | - | - |
|  | Yes | % | 0.258 | 0.090 | 0.739 | 0.012 |
|  | Comorbidities |  |  |  |  |  |
|  | Cardiovascular disease | % | 2.238 | 0.978 | 5.120 | 0.056 |
|  | Chronic neurological disease | % | 0.208 | 0.056 | 0.769 | 0.019 |
|  | SOFA |  |  |  |  |  |
|  | 0 - 3 | % | - | - | - | 0.090 |
|  | 4-7 | % | 0.637 | 0.197 | 2.064 | 0.452 |
|  | 8-9 | % | 1.608 | 0.386 | 6.704 | 0.514 |
|  | 10 - 11 | % | 0.994 | 0.248 | 3.987 | 0.994 |
|  | ≥12 | % | 3.810 | 0.801 | 18.130 | 0.093 |
|  | APACHE II |  |  |  |  |  |
|  | 0 - 9 | % | - | - | - | 0.294 |
|  | 10 - 14 | % | 0.338 | 0.071 | 1.595 | 0.170 |
|  | 15 - 19 | % | 0.547 | 0.121 | 2.465 | 0.432 |
|  | 20 - 24 | % | 0.972 | 0.215 | 4.400 | 0.971 |
|  | 25 - 29 | % | 1.604 | 0.248 | 10.361 | 0.620 |
|  | ≥ 30 | % | 0.610 | 0.107 | 3.487 | 0.578 |
|  | Site of Infection |  |  |  |  |  |
|  | Urinary tract | % | 0.227 | 0.059 | 0.871 | 0.031 |
|  | Skin or cutaneous sites | % | 1.982 | 0.573 | 6.856 | 0.280 |
|  | Pathogens detection |  |  |  |  |  |
|  | Gram negative bacteria | % | 1.511 | 0.696 | 3.283 | 0.297 |
|  | Completion of the sepsis bundle within 1 hour | % | 1.592 | 0.381 | 6.652 | 0.524 |
|  | Completion of the sepsis bundle within 3 hours | % | 1.726 | 0.405 | 7.348 | 0.460 |
|  | Completion of the administration of antibiotics within 3 hours | % | 0.159 | 0.052 | 0.483 | 0.001 |
|  | Respiratory support |  |  |  |  |  |
|  | Mechanical ventilation | % | 4.747 | 1.613 | 13.975 | 0.005 |
|  | High-flow nasal oxygen | % | 0.688 | 0.217 | 2.183 | 0.526 |
|  | Additional ICU support |  |  |  |  |  |
|  | Vasopressors/inotropes | % | 1.559 | 0.567 | 4.284 | 0.389 |
|  | Renal replacement therapy | % | 1.442 | 0.646 | 3.222 | 0.372 |
|  | Surgical source control | % | 0.258 | 0.069 | 0.957 | 0.043 |
|  | Non-surgical source control | % | 0.440 | 0.195 | 0.992 | 0.048 |
|  | Constant |  | 1.544 |  |  | 0.652 |
| 8 | University affiliation |  |  |  |  |  |
|  | No | % | - | - | - | - |
|  | Yes | % | 1.620 | 0.698 | 3.757 | 0.261 |
|  | Training programme in ICU |  |  |  |  |  |
|  | No | % | - | - | - | - |
|  | Yes | % | 0.242 | 0.086 | 0.683 | 0.007 |
|  | Comorbidities |  |  |  |  |  |
|  | Cardiovascular disease | % | 2.208 | 0.966 | 5.047 | 0.060 |
|  | Chronic neurological disease | % | 0.211 | 0.057 | 0.777 | 0.019 |
|  | SOFA |  |  |  |  |  |
|  | 0 - 3 | % | - | - | - | 0.098 |
|  | 4-7 | % | 0.625 | 0.193 | 2.024 | 0.433 |
|  | 8-9 | % | 1.535 | 0.374 | 6.298 | 0.552 |
|  | 10 - 11 | % | 0.992 | 0.247 | 3.981 | 0.990 |
|  | ≥12 | % | 3.644 | 0.772 | 17.204 | 0.102 |
|  | APACHE II |  |  |  |  |  |
|  | 0 - 9 | % | - | - | - | 0.297 |
|  | 10 - 14 | % | 0.353 | 0.076 | 1.653 | 0.186 |
|  | 15 - 19 | % | 0.572 | 0.129 | 2.545 | 0.463 |
|  | 20 - 24 | % | 1.030 | 0.231 | 4.580 | 0.969 |
|  | 25 - 29 | % | 1.715 | 0.269 | 10.926 | 0.568 |
|  | ≥ 30 | % | 0.658 | 0.117 | 3.698 | 0.635 |
|  | Site of Infection |  |  |  |  |  |
|  | Urinary tract | % | 0.233 | 0.061 | 0.885 | 0.032 |
|  | Skin or cutaneous sites | % | 2.042 | 0.589 | 7.075 | 0.260 |
|  | Pathogens detection |  |  |  |  |  |
|  | Gram negative bacteria | % | 1.495 | 0.689 | 3.243 | 0.309 |
|  | Completion of the sepsis bundle within 1 hour | % | 1.501 | 0.364 | 6.191 | 0.574 |
|  | Completion of the sepsis bundle within 3 hours | % | 1.802 | 0.425 | 7.645 | 0.424 |
|  | Completion of the administration of antibiotics within 3 hours | % | 0.161 | 0.053 | 0.490 | 0.001 |
|  | Respiratory support |  |  |  |  |  |
|  | Mechanical ventilation | % | 5.086 | 1.753 | 14.759 | 0.003 |
|  | Additional ICU support |  |  |  |  |  |
|  | Vasopressors/inotropes | % | 1.509 | 0.554 | 4.113 | 0.421 |
|  | Renal replacement therapy | % | 1.489 | 0.673 | 3.296 | 0.326 |
|  | Surgical source control | % | 0.273 | 0.074 | 0.999 | 0.050 |
|  | Non-surgical source control | % | 0.447 | 0.199 | 1.007 | 0.052 |
|  | Constant |  | 1.412 |  |  | 0.718 |
| 9 | University affiliation |  |  |  |  |  |
|  | No | % | - | - | - | - |
|  | Yes | % | 1.637 | 0.708 | 3.786 | 0.249 |
|  | Training programme in ICU |  |  |  |  |  |
|  | No | % | - | - | - | - |
|  | Yes | % | 0.240 | 0.085 | 0.677 | 0.007 |
|  | Comorbidities |  |  |  |  |  |
|  | Cardiovascular disease | % | 2.171 | 0.953 | 4.949 | 0.065 |
|  | Chronic neurological disease | % | 0.218 | 0.060 | 0.796 | 0.021 |
|  | SOFA |  |  |  |  |  |
|  | 0 - 3 | % | - | - | - | 0.104 |
|  | 4-7 | % | 0.612 | 0.189 | 1.979 | 0.413 |
|  | 8-9 | % | 1.493 | 0.366 | 6.090 | 0.576 |
|  | 10 - 11 | % | 0.928 | 0.235 | 3.659 | 0.915 |
|  | ≥12 | % | 3.455 | 0.739 | 16.162 | 0.115 |
|  | APACHE II |  |  |  |  |  |
|  | 0 - 9 | % | - | - | - | 0.276 |
|  | 10 - 14 | % | 0.352 | 0.075 | 1.641 | 0.184 |
|  | 15 - 19 | % | 0.552 | 0.124 | 2.456 | 0.436 |
|  | 20 - 24 | % | 1.052 | 0.237 | 4.675 | 0.947 |
|  | 25 - 29 | % | 1.705 | 0.268 | 10.849 | 0.572 |
|  | ≥ 30 | % | 0.665 | 0.118 | 3.735 | 0.643 |
|  | Site of Infection |  |  |  |  |  |
|  | Urinary tract | % | 0.240 | 0.063 | 0.909 | 0.036 |
|  | Skin or cutaneous sites | % | 2.065 | 0.596 | 7.152 | 0.253 |
|  | Pathogens detection |  |  |  |  |  |
|  | Gram negative bacteria | % | 1.473 | 0.681 | 3.185 | 0.325 |
|  | Completion of the sepsis bundle within 3 hours | % | 2.524 | 1.079 | 5.900 | 0.033 |
|  | Completion of the administration of antibiotics within 3 hours | % | 0.161 | 0.053 | 0.489 | 0.001 |
|  | Respiratory support |  |  |  |  |  |
|  | Mechanical ventilation | % | 5.044 | 1.747 | 14.564 | 0.003 |
|  | Additional ICU support |  |  |  |  |  |
|  | Vasopressors/inotropes | % | 1.503 | 0.554 | 4.080 | 0.424 |
|  | Renal replacement therapy | % | 1.490 | 0.673 | 3.297 | 0.325 |
|  | Surgical source control | % | 0.281 | 0.077 | 1.027 | 0.055 |
|  | Non-surgical source control | % | 0.450 | 0.200 | 1.013 | 0.054 |
|  | Constant |  | 1.475 |  |  | 0.683 |
| 10 | University affiliation |  |  |  |  |  |
|  | No | % | - | - | - | - |
|  | Yes | % | 1.613 | 0.701 | 3.712 | 0.261 |
|  | Training programme in ICU |  |  |  |  |  |
|  | No | % | - | - | - | - |
|  | Yes | % | 0.258 | 0.094 | 0.711 | 0.009 |
|  | Comorbidities |  |  |  |  |  |
|  | Cardiovascular disease | % | 2.098 | 0.925 | 4.760 | 0.076 |
|  | Chronic neurological disease | % | 0.205 | 0.057 | 0.747 | 0.016 |
|  | SOFA |  |  |  |  |  |
|  | 0 - 3 | % | - | - | - | 0.084 |
|  | 4-7 | % | 0.636 | 0.199 | 2.032 | 0.445 |
|  | 8-9 | % | 1.602 | 0.403 | 6.374 | 0.504 |
|  | 10 - 11 | % | 1.071 | 0.288 | 3.992 | 0.918 |
|  | ≥12 | % | 3.875 | 0.856 | 17.532 | 0.079 |
|  | APACHE II |  |  |  |  |  |
|  | 0 - 9 | % | - | - | - | 0.290 |
|  | 10 - 14 | % | 0.403 | 0.090 | 1.794 | 0.233 |
|  | 15 - 19 | % | 0.575 | 0.131 | 2.530 | 0.464 |
|  | 20 - 24 | % | 1.176 | 0.272 | 5.085 | 0.828 |
|  | 25 - 29 | % | 1.872 | 0.300 | 11.697 | 0.502 |
|  | ≥ 30 | % | 0.737 | 0.135 | 4.028 | 0.725 |
|  | Site of Infection |  |  |  |  |  |
|  | Urinary tract | % | 0.246 | 0.065 | 0.927 | 0.038 |
|  | Skin or cutaneous sites | % | 2.084 | 0.605 | 7.176 | 0.244 |
|  | Pathogens detection |  |  |  |  |  |
|  | Gram negative bacteria | % | 1.497 | 0.693 | 3.235 | 0.304 |
|  | Completion of the sepsis bundle within 3 hours | % | 2.563 | 1.095 | 5.999 | 0.030 |
|  | Completion of the administration of antibiotics within 3 hours | % | 0.165 | 0.055 | 0.500 | 0.001 |
|  | Respiratory support |  |  |  |  |  |
|  | Mechanical ventilation | % | 5.716 | 2.047 | 15.962 | 0.001 |
|  | Additional ICU support |  |  |  |  |  |
|  | Renal replacement therapy | % | 1.559 | 0.711 | 3.416 | 0.267 |
|  | Surgical source control | % | 0.294 | 0.082 | 1.054 | 0.060 |
|  | Non-surgical source control | % | 0.447 | 0.199 | 1.006 | 0.052 |
|  | Constant |  | 1.376 |  |  | 0.736 |
| 11 | University affiliation |  |  |  |  |  |
|  | No | % | - | - | - | - |
|  | Yes | % | 1.626 | 0.709 | 3.726 | 0.251 |
|  | Training programme in ICU |  |  |  |  |  |
|  | No | % | - | - | - | - |
|  | Yes | % | 0.261 | 0.095 | 0.714 | 0.009 |
|  | Comorbidities |  |  |  |  |  |
|  | Cardiovascular disease | % | 2.147 | 0.953 | 4.838 | 0.065 |
|  | Chronic neurological disease | % | 0.208 | 0.058 | 0.745 | 0.016 |
|  | SOFA |  |  |  |  |  |
|  | 0 - 3 | % | - | - | - | 0.086 |
|  | 4-7 | % | 0.628 | 0.197 | 1.998 | 0.430 |
|  | 8-9 | % | 1.576 | 0.399 | 6.235 | 0.516 |
|  | 10 - 11 | % | 1.017 | 0.275 | 3.759 | 0.980 |
|  | ≥12 | % | 3.718 | 0.828 | 16.687 | 0.086 |
|  | APACHE II |  |  |  |  |  |
|  | 0 - 9 | % | - | - | - | 0.347 |
|  | 10 - 14 | % | 0.428 | 0.098 | 1.876 | 0.260 |
|  | 15 - 19 | % | 0.610 | 0.140 | 2.654 | 0.510 |
|  | 20 - 24 | % | 1.173 | .273 | 5.035 | 0.830 |
|  | 25 - 29 | % | 1.803 | .293 | 11.101 | 0.525 |
|  | ≥ 30 | % | 0.755 | 0.139 | 4.114 | 0.745 |
|  | Site of Infection |  |  |  |  |  |
|  | Urinary tract | % | 0.251 | 0.067 | 0.944 | 0.041 |
|  | Skin or cutaneous sites | % | 1.896 | 0.566 | 6.350 | 0.300 |
|  | Completion of the sepsis bundle within 3 hours | % | 2.708 | 1.168 | 6.279 | 0.020 |
|  | Completion of the administration of antibiotics within 3 hours | % | 0.150 | 0.050 | 0.453 | 0.001 |
|  | Respiratory support |  |  |  |  |  |
|  | Mechanical ventilation | % | 6.051 | 2.175 | 16.832 | 0.001 |
|  | Additional ICU support |  | 1.625 | 0.746 | 3.542 | 0.222 |
|  | Renal replacement therapy | % | 0.308 | 0.086 | 1.102 | 0.070 |
|  | Surgical source control | % | 0.485 | 0.219 | 1.073 | 0.074 |
|  | Non-surgical source control | % | 6.051 | 2.175 | 16.832 | 0.575 |
|  | Constant |  | 1.682 |  |  | 0.001 |
| 12 | University affiliation |  |  |  |  |  |
|  | No | % | - | - | - | - |
|  | Yes | % | 1.707 | 0.761 | 3.832 | 0.195 |
|  | Training programme in ICU |  |  |  |  |  |
|  | No | % | - | - | - | - |
|  | Yes | % | 0.279 | 0.104 | 0.749 | 0.011 |
|  | Comorbidities |  |  |  |  |  |
|  | Cardiovascular disease | % | 2.328 | 1.044 | 5.194 | 0.039 |
|  | Chronic neurological disease | % | 0.266 | 0.079 | 0.894 | 0.032 |
|  | SOFA |  |  |  |  |  |
|  | 0 - 3 | % | - | - | - | 0.007 |
|  | 4-7 | % | .602 | .205 | 1.766 | 0.355 |
|  | 8-9 | % | 1.776 | .495 | 6.372 | 0.378 |
|  | 10 - 11 | % | 1.110 | .323 | 3.809 | 0.869 |
|  | ≥12 | % | 5.369 | 1.378 | 20.918 | 0.015 |
|  | Site of Infection |  |  |  |  |  |
|  | Urinary tract | % | .248 | .067 | .918 | 0.037 |
|  | Skin or cutaneous sites | % | 2.285 | .718 | 7.275 | 0.162 |
|  | Completion of the sepsis bundle within 3 hours | % | 2.548 | 1.142 | 5.683 | 0.022 |
|  | Completion of the administration of antibiotics within 3 hours | % | .172 | .058 | .510 | 0.002 |
|  | Respiratory support |  |  |  |  |  |
|  | Mechanical ventilation | % | 6.601 | 2.485 | 17.535 | <0.001 |
|  | Additional ICU support |  |  |  |  |  |
|  | Renal replacement therapy | % | 1.565 | .731 | 3.350 | 0.249 |
|  | Surgical source control | % | .305 | .090 | 1.031 | 0.056 |
|  | Non-surgical source control | % | .433 | .200 | .941 | 0.035 |
|  | Constant |  | 1.012 |  |  | 0.989 |
| 13 | University affiliation |  |  |  |  |  |
|  | No | % | - | - | - | - |
|  | Yes | % | 1.726 | 0.771 | 3.865 | 0.185 |
|  | Training programme in ICU |  |  |  |  |  |
|  | No | % | - | - | - | - |
|  | Yes | % | 0.288 | 0.109 | 0.761 | 0.012 |
|  | Comorbidities |  |  |  |  |  |
|  | Cardiovascular disease | % | 2.413 | 1.087 | 5.359 | 0.030 |
|  | Chronic neurological disease | % | 0.245 | 0.073 | 0.816 | 0.022 |
|  | SOFA |  |  |  |  |  |
|  | 0 - 3 | % | - | - | - | 0.001 |
|  | 4-7 | % | 0.632 | 0.216 | 1.848 | 0.402 |
|  | 8-9 | % | 2.103 | 0.608 | 7.273 | 0.240 |
|  | 10 - 11 | % | 1.282 | 0.383 | 4.290 | 0.687 |
|  | ≥12 | % | 6.701 | 1.803 | 24.912 | 0.005 |
|  | Site of Infection |  |  |  |  |  |
|  | Urinary tract | % | 0.259 | 0.070 | 0.958 | 0.043 |
|  | Skin or cutaneous sites | % | 2.320 | 0.745 | 7.225 | 0.147 |
|  | Completion of the sepsis bundle within 3 hours | % | 2.492 | 1.122 | 5.536 | 0.025 |
|  | Completion of the administration of antibiotics within 3 hours | % | 0.176 | 0.060 | 0.517 | 0.002 |
|  | Respiratory support |  |  |  |  |  |
|  | Mechanical ventilation | % | 7.170 | 2.717 | 18.923 | <0.001 |
|  | Additional ICU support |  |  |  |  |  |
|  | Surgical source control | % | 0.283 | 0.084 | 0.951 | 0.041 |
|  | Non-surgical source control | % | 0.464 | .217 | 0.990 | 0.047 |
|  | Constant |  | 0.969 |  |  | 0.969 |
| 14 | Training programme in ICU |  |  |  |  |  |
|  | No | % | - | - | - | - |
|  | Yes | % | 0.344 | 0.135 | 0.878 | 0.026 |
|  | Comorbidities |  |  |  |  |  |
|  | Cardiovascular disease | % | 2.284 | 1.035 | 5.041 | 0.041 |
|  | Chronic neurological disease | % | 0.213 | 0.065 | 0.699 | 0.011 |
|  | SOFA |  |  |  |  |  |
|  | 0 - 3 | % | - | - | - | 0.001 |
|  | 4-7 | % | 0.660 | 0.229 | 1.899 | 0.441 |
|  | 8-9 | % | 2.335 | 0.696 | 7.833 | 0.170 |
|  | 10 - 11 | % | 1.509 | 0.468 | 4.864 | 0.491 |
|  | ≥12 | % | 7.718 | 2.120 | 28.103 | 0.002 |
|  | Site of Infection |  |  |  |  |  |
|  | Urinary tract | % | 0.288 | 0.079 | 1.045 | 0.058 |
|  | Skin or cutaneous sites | % | 2.411 | 0.772 | 7.525 | 0.130 |
|  | Completion of the sepsis bundle within 3 hours | % | 2.675 | 1.213 | 5.899 | 0.015 |
|  | Completion of the administration of antibiotics within 3 hours | % | 0.156 | 0.053 | 0.454 | 0.001 |
|  | Respiratory support |  |  |  |  |  |
|  | Mechanical ventilation | % | 8.258 | 3.216 | 21.205 | <0.001 |
|  | Additional ICU support |  |  |  |  |  |
|  | Surgical source control | % | 0.310 | 0.094 | 1.022 | 0.054 |
|  | Non-surgical source control | % | 0.456 | 0.215 | 0.969 | 0.041 |
|  | Constant |  | 0.956 |  |  | 0.956 |
| 15 | Training programme in ICU |  |  |  |  |  |
|  | No | % | - | - | - | - |
|  | Yes | % | 0.309 | 0.122 | 0.783 | 0.013 |
|  | Comorbidities |  |  |  |  |  |
|  | Cardiovascular disease | % | 2.293 | 1.039 | 5.060 | 0.040 |
|  | Chronic neurological disease | % | 0.196 | 0.060 | 0.636 | 0.007 |
|  | SOFA |  |  |  |  |  |
|  | 0 - 3 | % | - | - | - | <0.001 |
|  | 4-7 | % | 0.633 | 0.224 | 1.794 | 0.390 |
|  | 8-9 | % | 2.461 | 0.742 | 8.167 | 0.141 |
|  | 10 - 11 | % | 1.520 | 0.475 | 4.860 | 0.480 |
|  | ≥12 | % | 7.381 | 2.050 | 26.577 | 0.002 |
|  | Site of Infection |  |  |  |  |  |
|  | Urinary tract | % | 0.294 | 0.083 | 1.048 | 0.059 |
|  | Completion of the sepsis bundle within 3 hours | % | 0.294 | 0.083 | 1.048 | 0.017 |
|  | Completion of the administration of antibiotics within 3 hours | % | 0.294 | 0.083 | 1.048 | <0.001 |
|  | Respiratory support |  |  |  |  |  |
|  | Mechanical ventilation | % | 7.861 | 3.116 | 19.830 | <0.001 |
|  | Additional ICU support |  |  |  |  |  |
|  | Surgical source control | % | 0.331 | 0.102 | 1.073 | 0.065 |
|  | Non-surgical source control | % | 0.488 | 0.233 | 1.023 | 0.057 |
|  | Constant |  | 1.279 |  |  | 0.750 |

**Table S12.** Hospital and intensive care unit characteristics according to intensive care unit survivability of patients with sepsis

| Variable | All cases  n=252 | Survived  n=168 | Died  n=84 | P |
| --- | --- | --- | --- | --- |
| Participating hospital, no. (%) |  |  |  | - |
| 115 People's | 25 (9.9) | 7 (4.2) | 18 (21.4) |  |
| Bach Mai | 26 (10.3) | 16 (9.5) | 10 (11.9) |  |
| Bai Chay | 14 (5.6) | 10 (6.0 ) | 4 (4.8) |  |
| Can Tho | 7 (2.8) | 4 (2.4 ) | 3 (3.6) |  |
| Cho Ray | 41 (16.3) | 22 (13.1) | 19 (22.6) |  |
| Da Nang | 12 (4.8) | 6 (3.6) | 6 (7.1) |  |
| Dong Da | 9 (3.6) | 6 (3.6) | 3 (3.6) |  |
| Hanoi Medical University | 12 (4.8) | 6 (3.6) | 6 (7.1) |  |
| Hue | 39 (15.5) | 31 (18.5) | 8 (9.5) |  |
| Saint Paul | 9 (3.6) | 9 (5.4) | 0 |  |
| Thai Nguyen | 2 (0.8) | 1 (0.6) | 1 (1.2) |  |
| Thanh Nhan | 1 (0.4) | 1 (0.6) | 0 |  |
| Vietnam–Czechoslovakia Friendship | 48 (19.0) | 42 (25.0) | 6 (7.1) |  |
| Vinmec Times City International | 7 (2.8) | 7 (4.2) | 0 |  |
| **Hospital characteristics** | | | | |
| Type of hospital, no. (%) |  |  |  | - |
| Rural | 0 | 0 | 0 |  |
| Urban | 252 (100) | 168 (100) | 84 (100) |  |
| University affiliation, no. (%) |  |  |  | 0.003 |
| No | 153 (60.7) | 113 (67.3) | 40 (47.6) |  |
| Yes | 99 (39.3) | 55 (32.7) | 44 (52.4) |  |
| **ICU characteristics** | | | | |
| Nature of ICU, no. (%) |  |  |  | - |
| Open | 0 | 0 | 0 |  |
| Closed | 252 (100) | 168 (100) | 84 (100) |  |
| Type of ICU, no. (%) |  |  |  | 0.857 |
| Medical | 110 (43.7) | 74 (44.0) | 36 (42.9) |  |
| Surgical | 0 | 0 | 0 |  |
| Mixed | 142 (56.3) | 94 (56.0) | 48 (57.1) |  |
| Nurse to patient ratio, no. (%) |  |  |  | 0.124 |
| 1 or more nurses : 1 patient | 7 (2.8) | 7 (4.2) | 0 |  |
| 1 nurse : 2 patients | 187 (74.2) | 120 (71.4) | 67 (79.8) |  |
| 1 nurse : 3 patients | 0 | 0 | 0 |  |
| 1 nurse : 4 or more patients | 58 (23.0) | 41 (24.4) | 17 (20.2) |  |
| Intensivist to patient ratio, no. (%) |  |  |  | 0.077 |
| 1 intensivist : 5 or fewer patients | 165 (65.5) | 105 (62.5) | 60 (71.4) |  |
| 1 intensivist : 6 to 8 patients | 75 (29.8) | 57 (33.9) | 18 (21.4) |  |
| 1 intensivist : 9 to 11 patients | 0 | 0 | 0 |  |
| 1 intensivist : 12 to 14 patients | 12 (4.8) | 6 (3.6) | 6 (7.1) |  |
| Training programme in ICU, no. (%) |  |  |  | 0.014 |
| No | 50 (19.8) | 26 (15.5) | 24 (28.6) |  |
| Yes | 202 (80.2) | 142 (84.5) | 60 (71.4) |  |

**Table S13.** Baseline characteristics according to intensive care unit survivability of patients with sepsis

| Variable | All cases  n=252 | Survived  n=168 | Died  n=84 | P |
| --- | --- | --- | --- | --- |
| Age (year), median (IQR) | 65 (52-76.75) | 65 (52-76) | 65 (52-77) | 0.971 |
| Age (year), no. (%) |  |  |  | 0.844 |
| < 20 | 3 (1.2) | 2 (1.2) | 1 (1.2) |  |
| 20 - 39 | 19 (7.5) | 11 (6.5) | 8 (9.5) |  |
| 40 - 59 | 74 (29.4) | 49 (29.2) | 25 (29.8) |  |
| ≥ 60 | 156 (61.9) | 106 (63.1) | 50 (59.5) |  |
| Sex (male), no. (%) | 162 (64.3) | 104 (61.9) | 58 (69.0) | 0.265 |
| Collection batch, no. (%) |  |  |  | 0.034 |
| Collection 1 (Jan) | 80 (31.7) | 61 (36.3) | 19 (22.6) |  |
| Collection 2 (April) | 62 (24.6) | 33 (19.6) | 29 (34.5) |  |
| Collection 3 (July) | 54 (21.4) | 35 (20.8) | 19 (22.6) |  |
| Collection 4 (Oct) | 56 (22.2) | 39 (23.2) | 17 (20.2) |  |
| Admission type, no. (%) |  |  |  | 0.393 |
| Medical | 236 (93.7) | 155 (92.3) | 81 (96.4) |  |
| Elective surgical | 2 (0.8) | 2 (1.2) | 0 |  |
| Unscheduled surgical | 14 (5.6) | 11 (6.5) | 3 (3.6) |  |
| Admission source, no. (%) |  |  |  | 0.351 |
| Emergency department | 138 (54.8) | 94 (56.0) | 44 (52.4) |  |
| Operating room | 4 (1.6) | 3 (1.8) | 1 (1.2) |  |
| General wards | 56 (22.2) | 39 (23.2) | 17 (20.2) |  |
| Other ICUs or HDU | 16 (6.3) | 12 (7.1) | 4 (4.8) |  |
| Inter-hospital transfer | 37 (14.7) | 20 (11.9) | 17 (20.2) |  |
| Others | 1 (0.4) | 0 | 1 (1.2) |  |
| Comorbidities, no. (%) |  |  |  |  |
| Cardiovascular disease | 78 (31.0) | 47 (28.0) | 31 (36.9) | 0.148 |
| Chronic lung disease | 30 (11.9) | 21 (12.5) | 9 (10.7) | 0.680 |
| Chronic neurological disease | 36 (14.3) | 28 (16.7) | 8 (9.5) | 0.127 |
| Chronic kidney disease | 23 (9.1) | 16 (9.5) | 7 (8.3) | 0.757 |
| Peptic ulcer disease | 9 (3.6) | 6 (3.6) | 3 (3.6) | >0.999 |
| Chronic liver disease | 27 (10.7) | 17 (10.1) | 10 (11.9) | 0.670 |
| Diabetes mellitus | 67 (26.6) | 44 (26.2) | 23 (27.4) | 0.840 |
| HIV infection | 0 | 0 | 0 | - |
| Connective tissue disease | 3 (1.2) | 2 (1.2) | 1 (1.2) | >0.999 |
| Immunosuppression | 10 (4.0) | 7 (4.2) | 3 (3.6) | >0.999 |
| Haematological malignancies | 5 (2.0) | 3 (1.8) | 2 (2.4) | >0.999 |
| Solid malignant tumours | 12 (4.8) | 6 (3.6) | 6 (7.1) | 0.222 |

**Table S14.** Clinical and laboratory characteristics and severity of illness according to intensive care unit survivability of patients with sepsis

| Variable | All cases  n=252 | Survived  n=168 | Died  n=84 | P |
| --- | --- | --- | --- | --- |
| **Vital signs** (on admission into ICU) | | | | |
| GCS, median (IQR) | 13 (9-15) | 14 (10-15) | 10 (8-14) | <0.001 |
| HR (beats per min), median (IQR) | 110 (95.25-125.75) | 109 (92-121) | 111.5 (100-130) | 0.008 |
| Temperature (^o^C), mean (SD) | 37.79 (1.01) | 37.82 (1.07) | 37.72 (0.88) | 0.485 |
| MBP (mmHg), mean(SD) | 75.82 (22.08) | 78.95 (22.80) | 69.54 (19.21) | 0.001 |
| SBP (mmHg), mean (SD) | 106.45 (29.96) | 110.64 (29.48) | 98.08 (29.33) | 0.002 |
| RR (breaths per min), median (IQR) | 25 (22-30) | 25 (22-30) | 25 (20.25-30) | >0.999 |
| **Blood investigations** | | | | |
| Total WBC (x10^9^/L), mean (SD) | 15.73 (9.20) | 15.70 (8.64) | 15.79 (10.28) | 0.941 |
| PLT (x10^9^/L), mean (SD) | 185.98 (137.85) | 203.72 (131.99) | 150.49 (143.17) | 0.004 |
| Hb (g/dL), mean (SD) | 11.14 (2.59) | 11.33 (2.62) | 10.77 (2.50) | 0.104 |
| Hct (%), mean (SD) | 34.31 (7.75) | 34.85 (7.76) | 33.24 (7.67) | 0.122 |
| K^+^ (mmol/L), mean (SD) | 3.89 (0.79) | 3.89 (0.80) | 3.87 (0.77) | 0.838 |
| Na^+^ (mmol/L), mean (SD) | 136.05 (8.24) | 135.21 (8.72) | 137.74 (6.92) | 0.021 |
| Creatinine (µmol/L), mean (SD) | 187.85 (151.92) | 188.47 (169.24) | 186.60 (110.29) | 0.927 |
| Bilirubin (µmol/l), mean (SD) | 32.80 (61.49) | 31.40 (69.33) | 35.52 (42.65) | 0.629 |
| pH, mean (SD) | 7.37 (0.50) | 7.40 (0.61) | 7.32 (0.13) | 0.249 |
| PaO_2_ (mmHg), mean (SD) | 116.17 (74.28) | 111.80 (64.31) | 124.49 (90.14) | 0.206 |
| FiO_2_, mean (SD) | 0.50 (0.22) | 0.45 (0.20) | 0.57 (0.24) | <0.001 |
| PaO_2_/FiO_2_ ratio, mean (SD) | 262.48 (149.58) | 273.45 (149.45) | 241.73 (148.49) | 0.116 |
| **Severity of illness scores** | | | | |
| qSOFA, median (IQR) | 2 (1-2) | 2 (1-2) | 2 (2-3) | 0.001 |
| qSOFA, no. (%) |  |  |  | 0.036 |
| 0 - 1 | 69 (27.4) | 53 (31.5) | 16 (19.0) |  |
| 2 - 3 | 183 (72.6) | 115 (68.5) | 68 (81.0) |  |
| SIRS, median (IQR) | 3 (2-4) | 3 (3-3.75) | 3 (2-4) | 0.792 |
| SOFA, median (IQR) | 7 (4.75-10) | 6 (4-9) | 10 (7-12.75) | <0.001 |
| SOFA, no. (%) |  |  |  | <0.001 |
| 0 - 1 | 0 | 0 | 0 |  |
| 2 - 3 | 46 (18.4) | 38 (22.9) | 8 (9.5) |  |
| 4 - 5 | 36 (14.4) | 29 (17.5) | 7 (8.3) |  |
| 6 - 7 | 58 (23.2) | 46 (27.7) | 12 (14.3) |  |
| 8 - 9 | 32 (12.8) | 20 (12.0) | 12 (14.3) |  |
| 10 - 11 | 38 (15.2) | 21 (12.7) | 17 (20.2) |  |
| 12 - 14 | 29 (11.6) | 9 (5.4) | 20 (23.8) |  |
| > 14 | 11 (4.4) | 3 (1.8) | 8 (9.5) |  |
| SOFA, no. (%) |  |  |  | <0.001 |
| 0 - 6 | 109 (43.6) | 89 (53.6) | 20 (23.8) |  |
| 7 - 9 | 63 (25.2) | 44 (26.5) | 19 (22.6) |  |
| 10 - 12 | 38 (15.2) | 21 (12.7) | 17 (20.2) |  |
| 13 - 14 | 29 (11.6) | 9 (5.4) | 20 (23.8) |  |
| > 14 | 11 (4.4) | 3 (1.8) | 8 (9.5) |  |
| SOFA, no. (%) |  |  |  | <0.001 |
| 0 - 3 | 46 (18.4) | 38 (22.9) | 8 (9.5) |  |
| 4 - 7 | 94 (37.6) | 75 (45.2) | 19 (22.6) |  |
| 8 - 9 | 32 (12.8) | 20 (12.0) | 12 (14.3) |  |
| 10 - 11 | 38 (15.2) | 21 (12.7) | 17 (20.2) |  |
| ≥ 12 | 40 (16.0) | 12 (7.2) | 28 (33.3) |  |
| APACHE II, median (IQR) | 18 (13-24) | 16 (12-22) | 22 (16.25-27) | <0.001 |
| APACHE II, no. (%) |  |  |  | <0.001 |
| 0 - 4 | 3 (1.2) | 3 (1.8) | 0 |  |
| 5 - 9 | 22 (8.7) | 17 (10.1) | 5 (6.0) |  |
| 10 - 14 | 61 (24.2) | 52 (31.0) | 9 (10.7) |  |
| 15 - 19 | 52 (20.6) | 36 (21.4) | 16 (19.0) |  |
| 20 - 24 | 58 (23.0) | 32 (19.0) | 26 (31.0) |  |
| 25 - 29 | 28 (11.1) | 11 (6.5) | 17 (20.2) |  |
| 30 - 34 | 19 (7.5) | 13 (7.7) | 6 (7.1) |  |
| > 34 | 9 (3.6) | 4 (2.4) | 5 (6.0) |  |
| APACHE II, no. (%) |  |  |  | <0.001 |
| 0 - 9 | 25 (9.9) | 20 (11.9) | 5 (6.0) |  |
| 10 - 14 | 61 (24.2) | 52 (31.0) | 9 (10.7_ |  |
| 15 - 19 | 52 (20.6) | 36 (21.4) | 16 (19.0) |  |
| 20 - 24 | 58 (23.0) | 32 (19.0) | 26 (31.0) |  |
| 25 - 29 | 28 (11.1) | 11 (6.5) | 17 (20.2) |  |
| ≥ 30 | 28 (11.1) | 17 (10.1) | 11 (13.1) |  |
| Sheptic Shock | 74 (29.4) | 43 (25.6) | 31 (36.9) | 0.063 |

**Table S15.** Sites of infection and microbiology according to intensive care unit survivability of patients with sepsis

| Variable | All cases  n=252 | Survived  n=168 | Died  n=84 | P |
| --- | --- | --- | --- | --- |
| **Site of Infection** | | | | |
| Respiratory, no. (%) | 143 (56.7) | 93 (55.4) | 50 (59.5) | 0.529 |
| Urinary tract, no. (%) | 37 (14.7) | 31 (18.5) | 6 (7.1) | 0.017 |
| Abdominal, no. (%) | 61 (24.2) | 37 (22.0) | 24 (28.6) | 0.253 |
| Neurological, no. (%) | 12 (4.8) | 9 (5.4) | 3 (3.6) | 0.756 |
| Bones or joints, no. (%) | 2 (0.8) | 2 (1.2) | 0 | 0.554 |
| Skin or cutaneous sites, no. (%) | 19 (7.5) | 9 (5.4) | 10 (11.9) | 0.063 |
| Intravascular catheter, no. (%) | 1 (0.4) | 1 (0.6) | 0 | >0.999 |
| Infective endocarditis, no. (%) | 1 (0.4) | 0 | 1 (1.2) | 0.333 |
| Primary bacteraemia, no. (%) | 7 (2.8) | 6 (3.6) | 1 (.2) | 0.430 |
| Systemic, no. (%) | 6 (2.4) | 4 (2.4) | 2 (2.4) | >0.999 |
| Others, no. (%) | - | - | - | - |
| **Microbiology** | | | | |
| No pathogens detected, no. (%) | 67 (26.6) | 50 (29.8) | 17 (20.2) | 0.107 |
| Gram negative bacteria, no. (%) | 156 (61.9) | 101 (60.1) | 55 (65.5) | 0.409 |
| *Klebsiella pneumonia* | 27 (10.7) | 17 (10.1) | 10 (11.9) | 0.666 |
| *Acinetobacter baumannii* | 45 (17.9) | 24 (14.3) | 21 (25.0) | 0.036 |
| *Escherichia coli* | 44 (17.5) | 31 (18.5) | 13 (15.5) | 0.557 |
| *Pseudomonas aeruginosa* | 24 (9.5) | 18 (10.7) | 6 (7.1) | 0.363 |
| *Stenotrophomonas maltophilia* | 2 (0.8) | 1 (0.6) | 1 (1.2) | >0.999 |
| *Proteus species* | 47 (18.7) | 30 (17.9) | 17 (20.2) | 0.647 |
| *Enterobacter cloacae* | 3 (1.2) | 3 (1.8) | 0 | 0.553 |
| *Bulkholderia pseudomallei* | 1 (0.4) | 1 (0.6) | 0 | >0.999 |
| *Other* | 0 | 0 | 0 | - |
| Gram positive bacteria, no. (%) | 34 (13.5) | 23 (13.7) | 11 (13.1) | 0.896 |
| *Enterococcus* | 5 (2.0) | 5 (3.0) | 0 | 0.173 |
| *MSSA* | 5 (2.0) | 3 (1.8) | 2 (2.4) | >0.999 |
| *MRSA* | 10 (4.0) | 7 (4.2) | 3 (3.6) | >0.999 |
| *Other Streptococcus species* | 12 (4.8) | 6 (3.6) | 6 (7.1) | 0.209 |
| *Streptococcus pneumonia* | 2 (0.8) | 2 (1.2) | 0 | 0.554 |
| Fungi, no. (%) | 7 (2.8) | 4 (2.4) | 3 (3.6) | 0.689 |
| *Candida species* | 7 (2.8) | 4 (2.4) | 3 (3.6) | 0.689 |
| *Aspergillus species* | 0 | 0 | 0 | - |
| *Others* | 0 | 0 | 0 | - |
| Viruses, no. (%) | 2 (0.8) | 0 | 2 (2.4) | 0.110 |
| *Influenza* | 1 (0.4) | 0 | 1 (1.2) | 0.333 |
| *Others* | 0 | 0 | 0 | - |
| *Dengue* | 1 (0.4) | 0 | 1 (1.2) | 0.333 |
| Other pathogens, no. (%) |  |  |  |  |
| *Anaerobes* | 0 | 0 | 0 | - |
| *Mycobacterium tuberculosis* | 4 (1.6) | 3 (1.8) | 1 (1.2) | >0.999 |
| *Malaria* | 0 | 0 | 0 | - |

**Table S16.** Completion of sepsis bundle elements according to intensive care unit survivability of patients with sepsis

| Variable | All cases  n=252 | Survived  n=168 | Died  n=84 | P |
| --- | --- | --- | --- | --- |
| **Timing of antibiotics administration** | | | | |
| Performed within 24 hours, no. (%) |  |  |  | 0.686 |
| 0-60 minutes | 173 (76.9) | 120 (77.4) | 53 (75.7) |  |
| 61-120 minutes | 21 (9.3) | 14 (9.0) | 7 (10.0) |  |
| 121-180 minutes | 14 (6.2) | 11 (7.1) | 3 (4.3) |  |
| >180 minutes | 17 (7.6) | 10 (6.5) | 7 (10.0) |  |
| Not performed within 24 hours, no. (%) | 0 | 0 | 0 | - |
| Timing of antibiotics administration, median (IQR), minutes | 30 (11-60) | 35 (15-60) | 30 (10-61.25) | 0.590 |
| **Timing of obtaining blood cultures** | | | | |
| Performed within 24 hours, no. (%) | n=197 | n=128 | n=69 | 0.545 |
| 0-60 minutes | 135 (68.5) | 87 (68.0) | 48 (69.6) |  |
| 61-120 minutes | 14 (7.1) | 7 (5.5) | 7 (10.1) |  |
| 121-180 minutes | 10 (5.1) | 7 (5.5) | 3 (4.3) |  |
| >180 minutes | 38 (19.3) | 27 (21.1) | 11 (15.9) |  |
| Not performed within 24 hours, no. (%) | 0 | 0 | 0 | - |
| Timing of obtaining blood cultures, median (IQR), minutes | 30.0 (15-114.5) | 30 (15-133.5) | 30 (10-90) | 0.371 |
| **Timing of obtaining lactate measurement** | | | | |
| Performed within 24 hours, no. (%) | n=198 | n=135 | n=63 | 0.827 |
| 0-60 minutes | 141 (71.2) | 95 (70.4) | 46 (73.0) |  |
| 61-120 minutes | 10 (5.1) | 6 (4.4) | 4 (6.3) |  |
| 121-180 minutes | 6 (3.0) | 5 (3.7) | 1 (1.6) |  |
| >180 minutes | 41 (20.7) | 29 (21.5) | 12 (19.0) |  |
| Not performed within 24 hours, no. (%) | 0 | 0 | 0 | - |
| Timing of obtaining lactate measurement, median (IQR), minutes | 30 (10-92) | 30 (11-159) | 30 (10-75) | 0.381 |

**Table S17.** Completion of the sepsis bundle of care and the initial administration of antibiotics according to intensive care unit survivability of patients with sepsis

| Variable | All cases  n=252 | Survived  n=168 | Died  n=84 | P |
| --- | --- | --- | --- | --- |
| Completion of the sepsis bundle within 1 hour, no. (%), n=241 | 87 (36.1) | 59 (36.6) | 28 (35.0) | 0.802 |
| Completion of the initial administration of antibiotics within 1 hour, no. (%), n=241 | 173 (71.8) | 120 (74.5) | 53 (66.3) | 0.178 |
| Permutations of the completed elements within 1 hour, no. (%) | n=241 | n=161 | n=80 | 0.311 |
| No elements completed | 20 (8.3) | 13 (8.1) | 7 (8.8) |  |
| Antibiotics only | 44 (18.3) | 32 (19.9) | 12 (15.0) |  |
| Blood cultures only | 13 (5.4) | 9 (5.6) | 4 (5.0) |  |
| Lactate only | 23 (9.5) | 12 (7.5) | 11 (13.8) |  |
| Antibiotics + Lactate | 17 (7.1) | 15 (9.3) | 2 (2.5) |  |
| Antibiotics + Blood cultures | 25 (10.4) | 14 (8.7) | 11 (13.8) |  |
| Blood cultures + Lactate | 12 (5.0) | 7 (4.3) | 5 (6.3) |  |
| Antibiotics + Blood cultures + Lactate | 87 (36.1) | 59 (36.6) | 28 (35.0) |  |
| Completion of the sepsis bundle within 3 hours, no. (%), n=241 | 108 (44.8) | 73 (45.3) | 35 (43.8) | 0.815 |
| Completion of the initial administration of antibiotics within 3 hours, no. (%), n=241 | 205 (85.1) | 143 (88.8) | 62 (77.5) | 0.020 |
| Permutation of the completed elements of 3-hour sepsis bundle, no. (%) | n=241 | n=161 | n=80 | 0.089 |
| No elements completed | 8 (3.3) | 4 (2.5) | 4 (5.0) |  |
| Antibiotics only | 37 (15.4) | 29 (18.0) | 8 (10.0) |  |
| Blood cultures only | 5 (2.1) | 2 (1.2) | 3 (3.8) |  |
| Lactate only | 16 (6.6) | 9 (5.6) | 7 (8.8) |  |
| Antibiotics + Lactate | 24 (10.0) | 20 (12.4) | 4 (5.0) |  |
| Antibiotics + Blood cultures | 36 (14.9) | 21 (13.0) | 15 (18.8) |  |
| Blood cultures + Lactate | 7 (2.9) | 3 (1.9) | 4 (5.0) |  |
| Antibiotics + Blood cultures + Lactate | 108 (44.8) | 73 (45.3) | 35 (43.8) |  |

**Table S18.** Life-sustaining treatments during ICU stay and outcomes according to intensive care unit survivability of patients with sepsis

| Variable | All cases  n=252 | Survived  n=168 | Died  n=84 | P |
| --- | --- | --- | --- | --- |
| **Life-sustaining treatments during ICU stay** | | | | |
| Respiratory support, no. (%) and median (IQR), days |  |  |  |  |
| Mechanical ventilation | 173/251 (68.9) | 97/167 (58.1) | 76/84 (90.5) | <0.001 |
| Duration of mechanical ventilation | 8 (4-15) | 9 (4-15) | 7 (3-15) | 0.502 |
| Non-invasive ventilation | 20/251 (8.0) | 14 (8.4) | 6 (7.1) | 0.732 |
| Duration of non-invasive ventilation | 2 (2-3.75) | 2 (1-2) | 5 (3.5-11.25) | 0.002 |
| High-flow nasal oxygen | 38/251 (15.1) | 33/167 (19.8) | 5/84 (6.0) | 0.004 |
| Duration of high-flow nasal oxygen | 2 (1-3) | 2 (1-3) | 3 (2.25-11.25) | 0.146 |
| Additional ICU support, no. (%) |  |  |  |  |
| Vasopressors/inotropes | 163 (64.7) | 96 (57.1) | 67 (79.8) | <0.001 |
| Renal replacement therapy | 101/251 (40.2) | 48/167 (28.7) | 43/84 (63.1) | <0.001 |
| Red blood cell transfusion | 93/251 (37.1) | 55/167 (32.9) | 38/84 (45.2) | 0.057 |
| Platelet transfusion | 50/251 (19.9) | 23/167 (13.8) | 27/84 (32.1) | 0.001 |
| Fresh frozen plasma transfusion | 58/251 (23.1) | 32/167 (19.2) | 26/84 (31.0) | 0.037 |
| Surgical source control | 25/251 (10.0) | 19/167 (11.4) | 6/84 (7.1) | 0.290 |
| Non-surgical source control | 78/251 (31.1) | 59/167 (35.3) | 19/84 (22.6) | 0.040 |
| Length of of surgical source control, median (IQR), minutes | 290 (105-630) | 290 (105-630) | 430 (270-1587) | 0.241 |
| Length of of surgical source control, n (%) | n=24 | n=19 | n=5 | 0.208 |
| <12 hours | 23 (95.8) | 19 (100) | 4 (80.0) |  |
| 12-24 hours | 1 (4.2) | 0 | 1 (20.0) |  |
| >24 hours | 0 | 0 | 0 |  |
| **Outcomes** | | | | |
| Outcomes |  |  |  | <0.001 |
| Alive upon current hospital discharge, no. (%) | 150 (59.5) | 150 (89.3) | 0 |  |
| Alive upon discharge from current ICU stay, but died in current hospital stay, no. (%) | 17 (6.7) | 17 (10.1) | 0 |  |
| Alive upon discharge from current ICU stay, but still in current hospital stay after 90 days, no. (%) | 1 (0.4) | 1 (0.6) | 0 |  |
| Still in current ICU stay after 90 days, no. (%) | 0 | 0 | 0 |  |
| Died in current ICU stay, no. (%) | 84 (33.3) |  | 84 (100) |  |
| Mortality, no. (%) |  |  |  |  |
| Hospital | 101 (40.1) | 17 (10.1) | 84 (100) | <0.001 |
| Length of stay, median days (IQR) |  |  |  |  |
| Hospital | 16 (10-25) | 17 (11-26) | 13 (6-22) | 0.002 |
| ICU | 10 (6-18) | 10 (6-17) | 10 (5-20.5) | 0.688 |

**Table S19.** Factors associated with intensive care unit mortality in patients with sepsis: bivariate regression analyses

| Factor | Frequency | OR | 95.0% CI for OR | | P |
| --- | --- | --- | --- | --- | --- |
|  |  |  | Lower | Upper |  |
| Participating hospital |  |  |  |  |  |
| 115 People's | 25 | - | - | - | 0.005 |
| Bach Mai | 26 | 0.243 | 0.075 | 0.789 | 0.019 |
| Bai Chay | 14 | 0.156 | 0.036 | 0.664 | 0.012 |
| Can Tho | 7 | 0.292 | 0.052 | 1.650 | 0.163 |
| Cho Ray | 41 | 0.336 | 0.116 | 0.976 | 0.045 |
| Da Nang | 12 | 0.389 | 0.093 | 1.624 | 0.195 |
| Dong Da | 9 | 0.194 | 0.038 | 1.000 | 0.050 |
| Hanoi Medical University | 12 | 0.389 | 0.093 | 1.624 | 0.195 |
| Hue | 39 | 0.100 | 0.031 | 0.323 | <0.001 |
| Saint Paul | 9 | 0.000 | 0.000 | - | 0.999 |
| Thai Nguyen | 2 | 0.389 | 0.021 | 7.111 | 0.524 |
| Thanh Nhan | 1 | 0.000 | 0.000 | - | >0.999 |
| Vietnam–Czechoslovakia Friendship | 48 | 0.056 | 0.016 | 0.189 | <0.001 |
| Vinmec Times City International | 7 | 0.000 | 0.000 | - | 0.999 |
| **Hospital characteristics** |  |  |  |  |  |
| University affiliation |  |  |  |  |  |
| No | 153 | - | - | - | - |
| Yes | 99 | 2.260 | 1.322 | 3.862 | 0.003 |
| **ICU characteristics** |  |  |  |  |  |
| Type of ICU |  |  |  |  |  |
| Medical | 110 | - | - | - | - |
| Mixed | 142 | 1.050 | 0.619 | 1.781 | 0.857 |
| Nurse to patient ratio |  |  |  |  |  |
| 1 nurse : 4 or more patients | 58 | - | - | - | 0.660 |
| 1 or more nurses : 1 patient | 7 | 0.000 | 0.000 | - | 0.999 |
| 1 nurse : 2 patients | 187 | 1.347 | 0.710 | 2.553 | 0.362 |
| Intensivist to patient ratio |  |  |  |  |  |
| 1 intensivist : 5 or fewer patients | 165 | - | - | - | 0.082 |
| 1 intensivist : 6 to 8 patients | 75 | 0.553 | 0.298 | 1.025 | 0.060 |
| 1 intensivist : 12 or more patients | 12 | 1.750 | 0.540 | 5.668 | 0.351 |
| Training programme in ICU |  |  |  |  |  |
| No | 50 | - | - | - | - |
| Yes | 202 | 0.458 | 0.243 | 0.861 | 0.015 |
| **Baseline characteristics** |  |  |  |  |  |
| Age (year) | 252 | 0.998 | 0.983 | 1.014 | 0.802 |
| Age (year) group |  |  |  |  |  |
| < 20 | 3 | - | - | - |  |
| 20 - 39 | 19 | 1.455 | 0.112 | 18.956 | 0.775 |
| 40 - 59 | 74 | 1.020 | 0.088 | 11.805 | 0.987 |
| ≥ 60 | 156 | 0.943 | 0.084 | 10.651 | 0.962 |
| Sex (male) | 162 | 0.728 | 0.417 | 1.272 | 0.265 |
| Collection batch |  |  |  |  |  |
| Collection 1 (Jan) | 80 | - | - | - | 0.038 |
| Collection 2 (April) | 62 | 2.821 | 1.377 | 5.779 | 0.005 |
| Collection 3 (July) | 54 | 1.743 | 0.815 | 3.725 | 0.152 |
| Collection 4 (Oct) | 56 | 1.399 | 0.649 | 3.016 | 0.391 |
| Admission type |  |  |  |  |  |
| Medical | 236 | - | - | - | 0.620 |
| Elective surgical | 2 | 0.000 | 0.000 | - | 0.999 |
| Unscheduled surgical | 14 | 0.522 | 0.142 | 1.924 | 0.329 |
| Admission source |  |  |  |  |  |
| Emergency department | 138 | - | - | - | 0.615 |
| Operating room | 4 | 0.712 | 0.072 | 7.041 | 0.772 |
| General wards | 56 | 0.931 | 0.475 | 1.825 | 0.836 |
| Other ICUs or HDU | 16 | 0.712 | 0.217 | 2.333 | 0575 |
| Inter-hospital transfer | 37 | 1.816 | 0.867 | 3.802 | 0.114 |
| Others | 1 | 3451241756 | 0.000 | - | >0.999 |
| Comorbidities |  |  |  |  |  |
| Cardiovascular disease | 78 | 1.506 | 0.863 | 2.627 | 0.150 |
| Chronic lung disease | 30 | 0.840 | 0.367 | 1.924 | 0.680 |
| Chronic neurological disease | 36 | 0.526 | 0.229 | 1.212 | 0.131 |
| Chronic kidney disease | 23 | 0.864 | 0.341 | 2.188 | 0.757 |
| Peptic ulcer disease | 9 | 1.000 | 0.244 | 4.101 | >0.999 |
| Chronic liver disease | 27 | 1.200 | 0.524 | 2.750 | 0.666 |
| Diabetes mellitus | 67 | 1.063 | 0.589 | 1.917 | 0.840 |
| Connective tissue disease | 3 | 1.000 | 0.089 | 11.188 | >0.999 |
| Immunosuppression | 10 | 0.852 | 0.215 | 3.381 | 0.820 |
| Haematological malignancies | 5 | 1.341 | 0.220 | 8.186 | 0.750 |
| Solid malignant tumours | 12 | 2.077 | 0.649 | 6.648 | 0.218 |
| **Vital signs** |  |  |  |  |  |
| GCS | 251 | 0.589 | 0.794 | 0.930 | <0.001 |
| HR (beats per min) | 252 | 1.017 | 1.003 | 1.030 | 0.014 |
| Temperature (^o^C) | 252 | 0.910 | 0.700 | 1.184 | 0.484 |
| MBP (mmHg) | 252 | 0.978 | 0.965 | 0.992 | 0.002 |
| SBP (mmHg) | 252 | 0.985 | 0.976 | 0.995 | 0.002 |
| RR (breaths per min) | 252 | 0.993 | 0.949 | 1.040 | 0.774 |
| **Blood investigations** |  |  |  |  |  |
| Total WBC (x10^9^/L) | 252 | 1.001 | 0.973 | 1.030 | 0.941 |
| PLT (x10^9^/L) | 252 | 0.997 | 0.994 | 0.999 | 0.005 |
| Hb (g/dL) | 251 | 0.918 | 0.828 | 1.018 | 0.105 |
| Hct (%) | 252 | 0.973 | 0.940 | 1.007 | 0.123 |
| K^+^ (mmol/L) | 252 | 0.966 | 0.692 | 1.348 | 0.837 |
| Na^+^ (mmol/L) | 252 | 1.038 | 1.005 | 1.073 | 0.025 |
| Creatinine (µmol/L) | 252 | 1.000 | 0.998 | 1.002 | 0.926 |
| Bilirubin (µmol/l) | 232 | 1.001 | 0.997 | 1.005 | 0.631 |
| pH | 248 | 0.131 | 0.016 | 1.108 | 0.062 |
| PaO_2_ (mmHg) | 244 | 1.002 | 0.999 | 1.006 | 0.212 |
| FiO_2_ | 245 | 11.704 | 3.405 | 40.224 | <0.001 |
| PaO_2_/FiO_2_ ratio | 243 | 0.998 | 0.997 | 1.000 | 0.119 |
| **Severity of illness scores** |  |  |  |  |  |
| qSOFA | 252 | 1.768 | 1.231 | 2.540 | 0.002 |
| qSOFA |  |  |  |  |  |
| 0 - 1 | 69 | - | - | - | - |
| 2 - 3 | 183 | 1.959 | 1.039 | 3.694 | 0.038 |
| SIRS | 252 | 1.045 | 0.798 | 1.368 | 0.750 |
| SOFA | 250 | 1.244 | 1.149 | 1.346 | <0.001 |
| SOFA |  |  |  |  |  |
| 2 - 3 | 46 | - | - | - | <0.001 |
| 4 - 5 | 36 | 1.147 | 0.373 | 3.527 | 0.811 |
| 6 - 7 | 58 | 1.239 | 0.459 | 3.343 | 0.672 |
| 8 - 9 | 32 | 2.850 | 1.002 | 8.109 | 0.050 |
| 10 - 11 | 38 | 3.845 | 1.422 | 10.401 | 0.008 |
| 12 - 14 | 29 | 10.556 | 3.529 | 31.569 | <0.001 |
| > 14 | 11 | 12.667 | 2.742 | 58.517 | 0.001 |
| SOFA |  |  |  |  |  |
| 0 - 6 | 109 | - | - | - | <0.001 |
| 7 - 9 | 63 | 1.922 | 0.931 | 3.965 | 0.077 |
| 10 - 12 | 38 | 3.602 | 1.6+14 | 8.038 | 0.002 |
| 13 - 14 | 29 | 9.889 | 3.924 | 24.918 | <0.001 |
| > 14 | 11 | 11.867 | 2.889 | 48.740 | 0.001 |
| SOFA |  |  |  |  |  |
| 0 - 3 | 46 | - | - | - | <0.001 |
| 4 - 7 | 94 | 1.203 | 0.483 | 3.000 | 0.691 |
| 8 - 9 | 32 | 2.850 | 1.002 | 8.109 | 0.050 |
| 10 - 11 | 38 | 3.845 | 1.422 | 10.401 | 0.008 |
| ≥ 12 | 40 | 11.083 | 4.000 | 30.709 | <0.001 |
| APACHE II | 252 | 1.076 | 1.039 | 1.114 | <0.001 |
| APACHE II |  |  |  |  |  |
| 0 - 4 | 3 | - | - | - | 0.002 |
| 5 - 9 | 22 | 475125698.2 | 0.000 | - | 0.999 |
| 10 - 14 | 61 | 279593199.3 | 0.000 | - | 0.999 |
| 15 - 19 | 52 | 717967721.6 | 0.000 | - | 0.999 |
| 20 - 24 | 58 | 1312534741 | 0.000 | - | 0.999 |
| 25 - 29 | 28 | 2496569578 | 0.000 | - | 0.999 |
| 30 - 34 | 19 | 745581864.8 | 0.000 | - | 0.999 |
| > 34 | 9 | 2019284217 | 0.000 | - | 0.999 |
| APACHE II |  |  |  |  |  |
| 0 - 9 | 25 | - | - | - | <0.001 |
| 10 - 14 | 61 | 0.692 | 0.207 | 2.319 | 0.551 |
| 15 - 19 | 52 | 1.778 | 0.567 | 5.577 | 0.324 |
| 20 - 24 | 58 | 3.250 | 1.073 | 9.844 | 0.037 |
| 25 - 29 | 28 | 6.182 | 1.790 | 21.344 | 0.004 |
| ≥ 30 | 28 | 2.588 | 0.750 | 8.937 | 0.133 |
| **Site of Infection** |  |  |  |  |  |
| Respiratory | 143 | 1.186 | 0.697 | 2.018 | 0.529 |
| Urinary tract | 37 | 0.340 | 0.136 | 0.851 | 0.021 |
| Abdominal | 61 | 1.416 | 0.779 | 2.575 | 0.254 |
| Neurological | 12 | 0.654 | 0.172 | 2.483 | 0.533 |
| Bones or joints | 2 | 0.000 | 0.000 | - | 0.999 |
| Skin or cutaneous sites | 19 | 2.387 | 0.931 | 6.123 | 0.070 |
| Intravascular catheter | 1 | 0.000 | 0.000 | - | >0.999 |
| Infective endocarditis | 1 | 3269876834 | 0.000 | - | >0.999 |
| Primary bacteraemia | 7 | 0.325 | 0.039 | 2.747 | 0.302 |
| Systemic | 6 | 1.000 | 0.179 | 5.573 | >0.999 |
| **Microbiology** |  |  |  |  |  |
| Pathogens detection |  |  |  |  |  |
| No pathogens detected | 67 | 0.599 | 0.320 | 1.121 | 0.109 |
| Gram negative bacteria | 156 | 1.258 | 0.729 | 2.171 | 0.409 |
| Gram positive bacteria | 34 | 0.950 | 0.439 | 2.055 | 0.896 |
| Fungi | 7 | 1.519 | 0.332 | 6.946 | 0.590 |
| Viruses | 2 | 3309753384 | 0.000 | - | 0.999 |
| Other pathogens | 4 | 0.663 | 0.068 | 6.469 | 0.723 |
| **Completion of sepsis bundle elements** |  |  |  |  |  |
| Timing of antibiotics administration |  |  |  |  |  |
| 0-60 minutes | 173 | - | - | - | 0.693 |
| 61-120 minutes | 21 | 1.132 | 0.432 | 2.966 | 0.801 |
| 121-180 minutes | 14 | 0.617 | 0.165 | 2.304 | 0.473 |
| >180 minutes | 17 | 1.585 | 0.572 | 4.389 | 0.376 |
| Timing of obtaining blood cultures |  |  |  |  |  |
| 0-60 minutes | 135 | - | - | - | 0.557 |
| 61-120 minutes | 14 | 1.812 | 0.600 | 5.474 | 0.292 |
| 121-180 minutes | 10 | 0.777 | 0.192 | 3.143 | 0.723 |
| >180 minutes | 38 | 0.738 | 0.337 | 1.618 | 0.449 |
| Timing of obtaining lactate measurement |  |  |  |  |  |
| 0-60 minutes | 141 | - | - | - | 0.785 |
| 61-120 minutes | 10 | 1.377 | 0.370 | 5.119 | 0.633 |
| 121-180 minutes | 6 | 0.413 | 0.047 | 3.638 | 0.426 |
| >180 minutes | 41 | 0.855 | 0.400 | 1.826 | 0.685 |
| Completion of the sepsis bundle within 1 hour | 87 | 0.931 | 0.532 | 1.630 | 0.802 |
| Completion of the initial administration of antibiotics within 1 hour | 173 | 0.671 | 0.374 | 1.202 | 0.180 |
| Permutations of the completed elements within 1 hour |  |  |  |  |  |
| No elements completed | 20 | - | - | - | 0.368 |
| Antibiotics only | 44 | 0.696 | 0.224 | 2.163 | 0.532 |
| Blood cultures only | 13 | 0.825 | 0.185 | 3.676 | 0.801 |
| Lactate only | 23 | 1.702 | 0.497 | 5.826 | 0.397 |
| Antibiotics + Lactate | 17 | 0.248 | 0.044 | 1.408 | 0.115 |
| Antibiotics + Blood cultures | 25 | 1.459 | 0.434 | 4.901 | 0.541 |
| Blood cultures + Lactate | 12 | 1.327 | 0.305 | 5.770 | 0.706 |
| Antibiotics + Blood cultures + Lactate | 87 | 0.881 | 0.317 | 2.452 | 0.809 |
| Completion of the sepsis bundle within 3 hours | 108 | 0.938 | 0.546 | 1.609 | 0.815 |
| Completion of the initial administration of antibiotics within 3 hours | 205 | 0.434 | 0.211 | 0.889 | 0.023 |
| Permutation of the completed elements of 3-hour sepsis bundle |  |  |  |  |  |
| No elements completed | 8 | - | - | - | 0.141 |
| Antibiotics only | 37 | 0.276 | 0.056 | 1.355 | 0.113 |
| Blood cultures only | 5 | 1.500 | 0.156 | 14.420 | 0.725 |
| Lactate only | 16 | 0.778 | 0.142 | 4.265 | 0.772 |
| Antibiotics + Lactate | 24 | 0.200 | 0.035 | 1.154 | 0.072 |
| Antibiotics + Blood cultures | 36 | 0.714 | 0.154 | 3.319 | 0.668 |
| Blood cultures + Lactate | 7 | 1.333 | 0.173 | 10.254 | 0.782 |
| Antibiotics + Blood cultures + Lactate | 108 | 0.479 | 0.113 | 2.030 | 0.318 |
| **Life-sustaining treatments during ICU stay** |  |  |  |  |  |
| Respiratory support |  |  |  |  |  |
| Mechanical ventilation | 173 | 6.856 | 3.109 | 15.116 | **<0.001** |
| Duration of mechanical ventilation | 167 | 1.005 | 0.976 | 1.036 | 0.722 |
| Non-invasive ventilation | 20 | 0.841 | 0.311 | 2.273 | 0.732 |
| Duration of non-invasive ventilation | 20 | 7.408 | 1.097 | 50.015 | 0.040 |
| High-flow nasal oxygen | 38 | 0.257 | 0.096 | 0.685 | **0.007** |
| Duration of high-flow nasal oxygen | 33 | 1.369 | 0.953 | 1.967 | 0.089 |
| Additional ICU support |  |  |  |  |  |
| Vasopressors/inotropes | 163 | 2.956 | 1.600 | 5.460 | **0.001** |
| Renal replacement therapy | 101 | 4.239 | 2.432 | 7.388 | **<0.001** |
| Red blood cell transfusion | 93 | 1.682 | 0.983 | 2.879 | **0.058** |
| Platelet transfusion | 50 | 2.966 | 1.571 | 5.597 | **0.001** |
| Fresh frozen plasma transfusion | 58 | 1.891 | 1.036 | 3.453 | **0.038** |
| Surgical source control | 25 | 0.599 | 0.230 | 1.562 | **0.295** |
| Non-surgical source control | 78 | 0.535 | 0.293 | 0.977 | **0.042** |

**Table S20.** Factors associated with intensive care unit mortality in patients with sepsis: multivariate logistic regression analyses (backward elimination)

| Step | Factor | Unit | OR | 95.0% CI for OR | | P |
| --- | --- | --- | --- | --- | --- | --- |
|  |  |  |  | Lower | Upper |  |
| 1 | University affiliation |  |  |  |  |  |
|  | No | % | - | - | - | - |
|  | Yes | % | 2.037 | 0.761 | 5.455 | 0.157 |
|  | Intensivist to patient ratio |  |  |  |  |  |
|  | 1 intensivist : 5 or fewer patients | % | - | - | - | 0.291 |
|  | 1 intensivist : 6 to 8 patients | % | 2.720 | 0.774 | 9.553 | 0.119 |
|  | 1 intensivist : 12 or more patients | % | 1.330 | 0.208 | 8.520 | 0.763 |
|  | Training programme in ICU |  |  |  |  |  |
|  | No | % | - | - | - | - |
|  | Yes | % | 0.207 | 0.068 | 0.626 | 0.005 |
|  | Comorbidities |  |  |  |  |  |
|  | Cardiovascular disease | % | 1.722 | 0.710 | 4.175 | 0.229 |
|  | Chronic neurological disease | % | 0.718 | 0.187 | 2.756 | 0.630 |
|  | Solid malignant tumours | % | 6.498 | 1.450 | 29.126 | 0.014 |
|  | qSOFA |  |  |  |  |  |
|  | 0 - 1 | % | - | - | - | - |
|  | 2 - 3 | % | 1.065 | 0.411 | 2.761 | 0.898 |
|  | SOFA |  |  |  |  |  |
|  | 0 - 3 | % | - | - | - | 0.085 |
|  | 4-7 | % | 1.361 | 0.347 | 5.346 | 0.659 |
|  | 8-9 | % | 2.241 | 0.416 | 12.065 | 0.348 |
|  | 10 - 11 | % | 3.096 | 0.618 | 15.521 | 0.169 |
|  | ≥12 | % | 8.454 | 1.506 | 47.447 | 0.015 |
|  | APACHE II |  |  |  |  |  |
|  | 0 - 9 | % | - | - | - | 0.384 |
|  | 10 - 14 | % | 0.220 | 0.037 | 1.317 | 0.097 |
|  | 15 - 19 | % | 0.379 | 0.068 | 2.120 | 0.269 |
|  | 20 - 24 | % | 0.487 | 0.082 | 2.887 | 0.428 |
|  | 25 - 29 | % | 0.625 | 0.083 | 4.740 | 0.650 |
|  | ≥ 30 | % | 0.217 | 0.028 | 1.669 | 0.142 |
|  | Site of Infection |  |  |  |  |  |
|  | Urinary tract | % | 0.217 | 0.050 | 0.941 | 0.041 |
|  | Abdominal | % | 0.778 | 0.277 | 2.186 | 0.634 |
|  | Skin or cutaneous sites | % | 2.297 | 0.564 | 9.350 | 0.245 |
|  | Pathogens detection |  |  |  |  |  |
|  | No pathogens detected | % | 1.007 | 0.228 | 4.446 | 0.992 |
|  | Gram negative bacteria | % | 1.172 | 0.315 | 4.361 | 0.813 |
|  | Completion of the sepsis bundle within 1 hour | % | 1.302 | 0.162 | 10.489 | 0.804 |
|  | Completion of the administration of antibiotics within 1 hour | % | 0.718 | 0.158 | 3.255 | 0.667 |
|  | Completion of the sepsis bundle within 3 hours | % | 1.401 | .197 | 9.984 | 0.736 |
|  | Completion of the administration of antibiotics within 3 hours | % | 0.242 | 0.045 | 1.303 | 0.099 |
|  | Respiratory support |  |  |  |  |  |
|  | Mechanical ventilation | % | 3.541 | 1.074 | 11.680 | 0.038 |
|  | High-flow nasal oxygen | % | 0.168 | 0.031 | 0.901 | 0.037 |
|  | Additional ICU support |  |  |  |  |  |
|  | Vasopressors/inotropes | % | 1.226 | 0.395 | 3.804 | 0.724 |
|  | Renal replacement therapy | % | 3.347 | 1.287 | 8.703 | 0.013 |
|  | Red blood cell transfusion | % | 0.661 | 0.263 | 1.661 | 0.378 |
|  | Platelet transfusion | % | 1.279 | 0.452 | 3.619 | 0.643 |
|  | Fresh frozen plasma transfusion | % | 1.195 | 0.439 | 3.254 | 0.728 |
|  | Surgical source control | % | 0.638 | 0.161 | 2.527 | 0.522 |
|  | Non-surgical source control | % | 0.293 | 0.115 | 0.744 | 0.010 |
|  | Constant |  | 0.971 |  |  | 0.982 |
| 2 | University affiliation |  |  |  |  |  |
|  | No | % | - | - | - | - |
|  | Yes | % | 2.037 | 0.762 | 5.448 | 0.156 |
|  | Intensivist to patient ratio |  |  |  |  |  |
|  | 1 intensivist : 5 or fewer patients | % | - | - | - | 0.289 |
|  | 1 intensivist : 6 to 8 patients | % | 2.719 | 0.776 | 9.522 | 0.118 |
|  | 1 intensivist : 12 or more patients | % | 1.330 | 0.208 | 8.517 | 0.763 |
|  | Training programme in ICU |  |  |  |  |  |
|  | No | % | - | - | - | - |
|  | Yes | % | 0.207 | 0.069 | 0.626 | 0.005 |
|  | Comorbidities |  |  |  |  |  |
|  | Cardiovascular disease | % | 1.721 | 0.719 | 4.117 | 0.223 |
|  | Chronic neurological disease | % | 0.719 | 0.188 | 2.745 | 0.629 |
|  | Solid malignant tumours | % | 6.498 | 1.450 | 29.120 | 0.014 |
|  | qSOFA |  |  |  |  |  |
|  | 0 - 1 | % | - | - | - | - |
|  | 2 - 3 | % | 1.065 | 0.416 | 2.728 | 0.895 |
|  | SOFA |  |  |  |  |  |
|  | 0 - 3 | % | - | - | - | 0.085 |
|  | 4-7 | % | 1.361 | 0.348 | 5.313 | 0.658 |
|  | 8-9 | % | 2.239 | 0.418 | 11.998 | 0.347 |
|  | 10 - 11 | % | 3.091 | 0.637 | 15.002 | 0.161 |
|  | ≥12 | % | 8.451 | 1.507 | 47.388 | 0.015 |
|  | APACHE II |  |  |  |  |  |
|  | 0 - 9 | % | - | - | - | 0.384 |
|  | 10 - 14 | % | 0.220 | 0.037 | 1.316 | 0.097 |
|  | 15 - 19 | % | 0.379 | 0.068 | 2.119 | 0.269 |
|  | 20 - 24 | % | 0.487 | 0.082 | 2.885 | 0.428 |
|  | 25 - 29 | % | 0.625 | 0.083 | 4.729 | 0.649 |
|  | ≥ 30 | % | 0.217 | 0.028 | 1.666 | 0.142 |
|  | Site of Infection |  |  |  |  |  |
|  | Urinary tract | % | 0.217 | 0.050 | 0.941 | 0.041 |
|  | Abdominal | % | 0.779 | 0.281 | 2.163 | 0.632 |
|  | Skin or cutaneous sites | % | 2.293 | 0.596 | 8.824 | 0.227 |
|  | Pathogens detection |  |  |  |  |  |
|  | Gram negative bacteria | % | 1.166 | 0.497 | 2.734 | 0.724 |
|  | Completion of the sepsis bundle within 1 hour | % | 1.301 | 0.162 | 10.459 | 0.804 |
|  | Completion of the administration of antibiotics within 1 hour | % | 0.718 | 0.159 | 3.251 | 0.667 |
|  | Completion of the sepsis bundle within 3 hours | % | 1.401 | 0.197 | 9.983 | 0.736 |
|  | Completion of the administration of antibiotics within 3 hours | % | 0.242 | 0.045 | 1.298 | 0.098 |
|  | Respiratory support |  |  |  |  |  |
|  | Mechanical ventilation | % | 3.540 | 1.076 | 11.648 | 0.038 |
|  | High-flow nasal oxygen | % | 0.168 | 0.031 | 0.898 | 0.037 |
|  | Additional ICU support |  |  |  |  |  |
|  | Vasopressors/inotropes | % | 1.227 | 0.401 | 3.756 | 0.720 |
|  | Renal replacement therapy | % | 3.347 | 1.287 | 8.701 | 0.013 |
|  | Red blood cell transfusion | % | 0.660 | 0.266 | 1.641 | 0.371 |
|  | Platelet transfusion | % | 1.279 | 0.452 | 3.619 | 0.643 |
|  | Fresh frozen plasma transfusion | % | 1.194 | 0.439 | 3.249 | 0.728 |
|  | Surgical source control | % | 0.638 | 0.161 | 2.527 | 0.522 |
|  | Non-surgical source control | % | 0.293 | 0.116 | 0.742 | 0.010 |
|  | Constant |  | 0.977 |  |  | 0.983 |
| 3 | University affiliation |  |  |  |  |  |
|  | No | % | - | - | - | - |
|  | Yes | % | 2.029 | 0.761 | 5.408 | 0.157 |
|  | Intensivist to patient ratio |  |  |  |  |  |
|  | 1 intensivist : 5 or fewer patients | % | - | - | - | 0.291 |
|  | 1 intensivist : 6 to 8 patients | % | 2.707 | 0.775 | 9.453 | 0.119 |
|  | 1 intensivist : 12 or more patients | % | 1.329 | 0.208 | 8.511 | 0.764 |
|  | Training programme in ICU |  |  |  |  |  |
|  | No | % | - | - | - | - |
|  | Yes | % | .207 | 0.068 | 0.623 | 0.005 |
|  | Comorbidities |  |  |  |  |  |
|  | Cardiovascular disease | % | 1.723 | 0.721 | 4.120 | 0.221 |
|  | Chronic neurological disease | % | 0.724 | 0.191 | 2.753 | 0.636 |
|  | Solid malignant tumours | % | 6.496 | 1.450 | 29.101 | 0.014 |
|  | SOFA |  |  |  |  |  |
|  | 0 - 3 | % | - | - | - | 0.082 |
|  | 4-7 | % | 1.389 | 0.370 | 5.220 | 0.626 |
|  | 8-9 | % | 2.298 | 0.449 | 11.756 | 0.318 |
|  | 10 - 11 | % | 3.158 | 0.672 | 14.829 | 0.145 |
|  | ≥12 | % | 8.626 | 1.582 | 47.035 | 0.013 |
|  | APACHE II |  |  |  |  |  |
|  | 0 - 9 | % | - | - | - | 0.371 |
|  | 10 - 14 | % | 0.218 | 0.037 | 1.288 | 0.093 |
|  | 15 - 19 | % | 0.376 | 0.068 | 2.087 | 0.263 |
|  | 20 - 24 | % | 0.484 | 0.082 | 2.860 | 0.424 |
|  | 25 - 29 | % | 0.623 | 0.083 | 4.704 | 0.647 |
|  | ≥ 30 | % | 0.216 | 0.028 | 1.653 | 0.140 |
|  | Site of Infection |  |  |  |  |  |
|  | Urinary tract | % | 0.217 | 0.050 | 0.943 | 0.041 |
|  | Abdominal | % | 0.780 | 0.281 | 2.166 | 0.634 |
|  | Skin or cutaneous sites | % | 2.290 | 0.595 | 8.809 | 0.228 |
|  | Pathogens detection |  |  |  |  |  |
|  | Gram negative bacteria | % | 1.154 | 0.499 | 2.671 | 0.737 |
|  | Completion of the sepsis bundle within 1 hour | % | 1.316 | 0.166 | 10.461 | 0.795 |
|  | Completion of the administration of antibiotics within 1 hour | % | 0.718 | 0.159 | 3.243 | 0.666 |
|  | Completion of the sepsis bundle within 3 hours | % | 1.391 | 0.197 | 9.843 | 0.741 |
|  | Completion of the administration of antibiotics within 3 hours | % | .243 | 0.045 | 1.301 | 0.098 |
|  | Respiratory support |  |  |  |  |  |
|  | Mechanical ventilation | % | 3.573 | 1.094 | 11.664 | 0.035 |
|  | High-flow nasal oxygen | % | 0.166 | 0.031 | 0.889 | 0.036 |
|  | Additional ICU support |  |  |  |  |  |
|  | Vasopressors/inotropes | % | 1.229 | 0.401 | 3.760 | 0.718 |
|  | Renal replacement therapy | % | 3.346 | 1.287 | 8.699 | 0.013 |
|  | Red blood cell transfusion | % | 0.661 | 0.266 | 1.643 | 0.373 |
|  | Platelet transfusion | % | 1.286 | 0.456 | 3.627 | 0.634 |
|  | Fresh frozen plasma transfusion | % | 1.197 | 0.441 | 3.251 | 0.725 |
|  | Surgical source control | % | 0.636 | 0.160 | 2.525 | 0.520 |
|  | Non-surgical source control | % | 0.293 | 0.116 | 0.743 | 0.010 |
|  | Constant |  | 1.007 |  |  | 0.995 |
| 4 | University affiliation |  |  |  |  |  |
|  | No | % | - | - | - | - |
|  | Yes | % | 2.056 | 0.777 | 5.442 | 0.147 |
|  | Intensivist to patient ratio |  |  |  |  |  |
|  | 1 intensivist : 5 or fewer patients | % | - | - | - | 0.279 |
|  | 1 intensivist : 6 to 8 patients | % | 2.732 | 0.785 | 9.508 | 0.114 |
|  | 1 intensivist : 12 or more patients | % | 1.290 | 0.204 | 8.169 | 0.787 |
|  | Training programme in ICU |  |  |  |  |  |
|  | No | % | - | - | - | - |
|  | Yes | % | 0.205 | 0.068 | 0.619 | 0.005 |
|  | Comorbidities |  |  |  |  |  |
|  | Cardiovascular disease | % | 1.715 | 0.719 | 4.094 | 0.224 |
|  | Chronic neurological disease | % | .735 | 0.194 | 2.778 | 0.650 |
|  | Solid malignant tumours | % | 6.549 | 1.457 | 29.435 | 0.014 |
|  | SOFA |  |  |  |  |  |
|  | 0 - 3 | % | - | - | - | 0.084 |
|  | 4-7 | % | 1.366 | 0.366 | 5.101 | 0.642 |
|  | 8-9 | % | 2.269 | 0.445 | 11.561 | 0.324 |
|  | 10 - 11 | % | 3.100 | 0.664 | 14.469 | 0.150 |
|  | ≥12 | % | 8.402 | 1.562 | 45.198 | 0.013 |
|  | APACHE II |  |  |  |  |  |
|  | 0 - 9 | % | - | - | - | 0.373 |
|  | 10 - 14 | % | 0.220 | 0.037 | 1.299 | 0.095 |
|  | 15 - 19 | % | 0.372 | 0.067 | 2.067 | 0.258 |
|  | 20 - 24 | % | 0.490 | 0.083 | 2.885 | 0.430 |
|  | 25 - 29 | % | 0.612 | 0.081 | 4.610 | 0.634 |
|  | ≥ 30 | % | 0.214 | 0.028 | 1.641 | 0.138 |
|  | Site of Infection |  |  |  |  |  |
|  | Urinary tract | % | 0.218 | 0.050 | .945 | 0.042 |
|  | Abdominal | % | 0.775 | 0.280 | 2.149 | 0.624 |
|  | Skin or cutaneous sites | % | 2.314 | 0.602 | 8.894 | 0.222 |
|  | Pathogens detection |  |  |  |  |  |
|  | Gram negative bacteria | % | 1.137 | 0.495 | 2.607 | 0.762 |
|  | Completion of the administration of antibiotics within 1 hour | % | .824 | 0.275 | 2.465 | 0.729 |
|  | Completion of the sepsis bundle within 3 hours | % | 1.751 | 0.705 | 4.345 | 0.227 |
|  | Completion of the administration of antibiotics within 3 hours | % | 0.217 | 0.051 | 0.914 | 0.037 |
|  | Respiratory support |  |  |  |  |  |
|  | Mechanical ventilation | % | 3.544 | 1.087 | 11.555 | 0.036 |
|  | High-flow nasal oxygen | % | 0.165 | 0.031 | 0.882 | 0.035 |
|  | Additional ICU support |  |  |  |  |  |
|  | Vasopressors/inotropes | % | 1.237 | 0.405 | 3.776 | 0.709 |
|  | Renal replacement therapy | % | 3.391 | 1.312 | 8.767 | 0.012 |
|  | Red blood cell transfusion | % | 0.659 | 0.266 | 1.638 | 0.370 |
|  | Platelet transfusion | % | 1.271 | 0.453 | 3.567 | 0.649 |
|  | Fresh frozen plasma transfusion | % | 1.223 | 0.456 | 3.275 | 0.689 |
|  | Surgical source control | % | 0.651 | 0.165 | 2.562 | 0.539 |
|  | Non-surgical source control | % | 0.291 | 0.115 | 0.736 | 0.009 |
|  | Constant |  | 1.024 |  |  | 0.983 |
| 5 | University affiliation |  |  |  |  |  |
|  | No | % | - | - | - | - |
|  | Yes | % | 2.065 | 0.781 | 5.460 | 0.144 |
|  | Intensivist to patient ratio |  |  |  |  |  |
|  | 1 intensivist : 5 or fewer patients | % | - | - | - | 0.253 |
|  | 1 intensivist : 6 to 8 patients | % | 2.803 | 0.816 | 9.630 | 0.102 |
|  | 1 intensivist : 12 or more patients | % | 1.288 | 0.205 | 8.095 | 0.787 |
|  | Training programme in ICU |  |  |  |  |  |
|  | No | % | - | - | - | - |
|  | Yes | % | .205 | 0.068 | .616 | 0.005 |
|  | Comorbidities |  |  |  |  |  |
|  | Cardiovascular disease | % | 1.726 | 0.724 | 4.112 | 0.218 |
|  | Chronic neurological disease | % | 0.730 | 0.194 | 2.746 | 0.641 |
|  | Solid malignant tumours | % | 6.453 | 1.440 | 28.918 | 0.015 |
|  | SOFA |  |  |  |  |  |
|  | 0 - 3 | % | - | - | - | 0.085 |
|  | 4-7 | % | 1.353 | 0.363 | 5.047 | 0.652 |
|  | 8-9 | % | 2.255 | 0.443 | 11.472 | 0.327 |
|  | 10 - 11 | % | 3.078 | 0.660 | 14.353 | 0.152 |
|  | ≥12 | % | 8.334 | 1.551 | 44.795 | 0.013 |
|  | APACHE II |  |  |  |  |  |
|  | 0 - 9 | % | - | - | - | 0.381 |
|  | 10 - 14 | % | 0.223 | 0.038 | 1.306 | 0.096 |
|  | 15 - 19 | % | 0.382 | 0.070 | 2.095 | 0.268 |
|  | 20 - 24 | % | 0.491 | 0.084 | 2.875 | 0.430 |
|  | 25 - 29 | % | 0.610 | 0.082 | 4.561 | 0.630 |
|  | ≥ 30 | % | 0.217 | 0.028 | 1.650 | 0.140 |
|  | Site of Infection |  |  |  |  |  |
|  | Urinary tract | % | 0.215 | 0.050 | 0.931 | 0.040 |
|  | Abdominal | % | 0.752 | 0.277 | 2.044 | 0.576 |
|  | Skin or cutaneous sites | % | 2.256 | 0.596 | 8.533 | 0.231 |
|  | Completion of the administration of antibiotics within 1 hour | % | 0.819 | 0.275 | 2.437 | 0.720 |
|  | Completion of the sepsis bundle within 3 hours | % | 1.775 | 0.718 | 4.387 | 0.214 |
|  | Completion of the administration of antibiotics within 3 hours | % | 0.213 | 0.051 | 0.890 | 0.034 |
|  | Respiratory support |  |  |  |  |  |
|  | Mechanical ventilation | % | 3.628 | 1.122 | 11.725 | 0.031 |
|  | High-flow nasal oxygen | % | 0.164 | 0.031 | 0.874 | 0.034 |
|  | Additional ICU support |  |  |  |  |  |
|  | Vasopressors/inotropes | % | 1.232 | 0.404 | 3.750 | 0.714 |
|  | Renal replacement therapy | % | 3.436 | 1.333 | 8.857 | 0.011 |
|  | Red blood cell transfusion | % | 0.675 | 0.276 | 1.654 | 0.390 |
|  | Platelet transfusion | % | 1.256 | 0.449 | 3.516 | 0.664 |
|  | Fresh frozen plasma transfusion | % | 1.240 | 0.465 | 3.304 | 0.667 |
|  | Surgical source control | % | 0.668 | 0.171 | 2.610 | 0.562 |
|  | Non-surgical source control | % | 0.294 | 0.116 | 0.741 | 0.009 |
|  | Constant |  | 1.086 |  |  | 0.940 |
| 6 | University affiliation |  |  |  |  |  |
|  | No | % | - | - | - | - |
|  | Yes | % | 2.047 | 0.775 | 5.405 | 0.148 |
|  | Intensivist to patient ratio |  |  |  |  |  |
|  | 1 intensivist : 5 or fewer patients | % | - | - | - | 0.266 |
|  | 1 intensivist : 6 to 8 patients | % | 2.730 | 0.803 | 9.280 | 0.108 |
|  | 1 intensivist : 12 or more patients | % | 1.325 | 0.215 | 8.178 | 0.762 |
|  | Training programme in ICU |  |  |  |  |  |
|  | No | % | - | - | - | - |
|  | Yes | % | 0.205 | 0.068 | 0.618 | 0.005 |
|  | Comorbidities |  |  |  |  |  |
|  | Cardiovascular disease | % | 1.757 | 0.741 | 4.163 | 0.201 |
|  | Chronic neurological disease | % | 0.714 | 0.191 | 2.678 | 0.618 |
|  | Solid malignant tumours | % | 6.243 | 1.401 | 27.824 | 0.016 |
|  | SOFA |  |  |  |  |  |
|  | 0 - 3 | % | - | - | - | 0.084 |
|  | 4-7 | % | 1.345 | 0.361 | 5.008 | 0.659 |
|  | 8-9 | % | 2.253 | 0.443 | 11.459 | 0.328 |
|  | 10 - 11 | % | 3.112 | 0.667 | 14.511 | 0.148 |
|  | ≥12 | % | 8.315 | 1.548 | 44.661 | 0.014 |
|  | APACHE II |  |  |  |  |  |
|  | 0 - 9 | % | - | - | - | 0.230 |
|  | 10 - 14 | % | 0.230 | 0.040 | 1.326 | 0.389 |
|  | 15 - 19 | % | 0.389 | 0.072 | 2.107 | 0.499 |
|  | 20 - 24 | % | 0.499 | 0.086 | 2.879 | 0.608 |
|  | 25 - 29 | % | 0.608 | 0.082 | 4.499 | 0.214 |
|  | ≥ 30 | % | 0.214 | 0.028 | 1.623 | 0.230 |
|  | Site of Infection |  |  |  |  |  |
|  | Urinary tract | % | 0.214 | 0.049 | 0.927 | 0.039 |
|  | Abdominal | % | 0.772 | 0.288 | 2.073 | 0.608 |
|  | Skin or cutaneous sites | % | 2.233 | 0.594 | 8.403 | 0.235 |
|  | Completion of the sepsis bundle within 3 hours | % | 1.772 | 0.717 | 4.380 | 0.215 |
|  | Completion of the administration of antibiotics within 3 hours | % | 0.182 | 0.057 | 0.576 | 0.004 |
|  | Respiratory support |  |  |  |  |  |
|  | Mechanical ventilation | % | 3.585 | 1.108 | 11.602 | 0.033 |
|  | High-flow nasal oxygen | % | 0.166 | 0.031 | 0.879 | 0.035 |
|  | Additional ICU support |  |  |  |  |  |
|  | Vasopressors/inotropes | % | 1.246 | 0.409 | 3.793 | 0.699 |
|  | Renal replacement therapy | % | 3.392 | 1.320 | 8.721 | 0.011 |
|  | Red blood cell transfusion | % | 0.677 | 0.277 | 1.655 | 0.392 |
|  | Platelet transfusion | % | 1.236 | 0.443 | 3.450 | 0.686 |
|  | Fresh frozen plasma transfusion | % | 1.258 | 0.473 | 3.341 | 0.646 |
|  | Surgical source control | % | 0.658 | 0.169 | 2.560 | 0.546 |
|  | Non-surgical source control | % | 0.292 | 0.116 | 0.734 | 0.009 |
|  | Constant |  | 1.076 |  |  | 0.946 |
| 7 | University affiliation |  |  |  |  |  |
|  | No | % | - | - | - | - |
|  | Yes | % | 2.023 | 0.769 | 5.320 | 0.153 |
|  | Intensivist to patient ratio |  |  |  |  |  |
|  | 1 intensivist : 5 or fewer patients | % | - | - | - | 0.253 |
|  | 1 intensivist : 6 to 8 patients | % | 2.766 | 0.819 | 9.341 | 0.101 |
|  | 1 intensivist : 12 or more patients | % | 1.324 | 0.215 | 8.160 | 0.762 |
|  | Training programme in ICU |  |  |  |  |  |
|  | No | % | - | - | - | - |
|  | Yes | % | 0.214 | 0.072 | 0.630 | 0.005 |
|  | Comorbidities |  |  |  |  |  |
|  | Cardiovascular disease | % | 1.731 | 0.733 | 4.087 | 0.211 |
|  | Chronic neurological disease | % | 0.696 | 0.187 | 2.587 | 0.588 |
|  | Solid malignant tumours | % | 6.165 | 1.388 | 27.385 | 0.017 |
|  | SOFA |  |  |  |  |  |
|  | 0 - 3 | % | - | - | - | 0.057 |
|  | 4-7 | % | 1.343 | 0.361 | 4.994 | 0.659 |
|  | 8-9 | % | 2.372 | 0.478 | 11.763 | 0.291 |
|  | 10 - 11 | % | 3.331 | 0.743 | 14.927 | 0.116 |
|  | ≥12 | % | 8.777 | 1.668 | 46.178 | 0.010 |
|  | APACHE II |  |  |  |  |  |
|  | 0 - 9 | % | - | - | - | 0.398 |
|  | 10 - 14 | % | 0.250 | 0.046 | 1.358 | 0.108 |
|  | 15 - 19 | % | 0.400 | 0.075 | 2.146 | 0.285 |
|  | 20 - 24 | % | 0.528 | 0.094 | 2.963 | 0.468 |
|  | 25 - 29 | % | 0.637 | 0.088 | 4.631 | 0.656 |
|  | ≥ 30 | % | 0.230 | 0.031 | 1.674 | 0.147 |
|  | Site of Infection |  |  |  |  |  |
|  | Urinary tract | % | 0.219 | 0.051 | 0.942 | 0.041 |
|  | Abdominal | % | 0.773 | 0.288 | 2.073 | 0.609 |
|  | Skin or cutaneous sites | % | 2.261 | 0.603 | 8.480 | 0.226 |
|  | Completion of the sepsis bundle within 3 hours | % | 1.781 | 0.720 | 4.407 | 0.212 |
|  | Completion of the administration of antibiotics within 3 hours | % | 0.184 | 0.058 | 0.580 | 0.004 |
|  | Respiratory support |  |  |  |  |  |
|  | Mechanical ventilation | % | 3.871 | 1.271 | 11.790 | 0.017 |
|  | High-flow nasal oxygen | % | 0.164 | 0.031 | 0.871 | 0.034 |
|  | Renal replacement therapy | % | 3.502 | 1.380 | 8.889 | 0.008 |
|  | Red blood cell transfusion | % | 0.693 | 0.287 | 1.675 | 0.415 |
|  | Platelet transfusion | % | 1.207 | 0.436 | 3.343 | 0.718 |
|  | Fresh frozen plasma transfusion | % | 1.245 | 0.471 | 3.287 | 0.659 |
|  | Surgical source control | % | 0.668 | 0.173 | 2.578 | 0.558 |
|  | Non-surgical source control | % | 0.290 | 0.115 | 0.730 | 0.009 |
|  | Constant |  | 1.035 |  |  | 0.974 |
| 8 | University affiliation |  |  |  |  |  |
|  | No | % | - | - | - | - |
|  | Yes | % | 2.088 | 0.809 | 5.389 | 0.128 |
|  | Intensivist to patient ratio |  |  |  |  |  |
|  | 1 intensivist : 5 or fewer patients | % | - | - | - | 0.255 |
|  | 1 intensivist : 6 to 8 patients | % | 2.740 | 0.812 | 9.244 | 0.104 |
|  | 1 intensivist : 12 or more patients | % | 1.265 | 0.210 | 7.636 | 0.798 |
|  | Training programme in ICU |  |  |  |  |  |
|  | No | % | - | - | - | - |
|  | Yes | % | 0.209 | 0.071 | 0.613 | 0.004 |
|  | Comorbidities |  |  |  |  |  |
|  | Cardiovascular disease | % | 1.722 | 0.729 | 4.067 | 0.215 |
|  | Chronic neurological disease | % | 0.688 | 0.185 | 2.556 | 0.577 |
|  | Solid malignant tumours | % | 6.257 | 1.421 | 27.565 | 0.015 |
|  | SOFA |  |  |  |  |  |
|  | 0 - 3 | % | - | - | - | 0.049 |
|  | 4-7 | % | 1.352 | 0.363 | 5.031 | 0.653 |
|  | 8-9 | % | 2.442 | 0.495 | 12.048 | 0.273 |
|  | 10 - 11 | % | 3.411 | 0.765 | 15.212 | 0.108 |
|  | ≥12 | % | 9.051 | 1.735 | 47.208 | 0.009 |
|  | APACHE II |  |  |  |  |  |
|  | 0 - 9 | % | - | - | - | 0.402 |
|  | 10 - 14 | % | 0.248 | 0.046 | 1.346 | 0.106 |
|  | 15 - 19 | % | 0.391 | 0.073 | 2.086 | 0.271 |
|  | 20 - 24 | % | 0.513 | 0.092 | 2.863 | 0.447 |
|  | 25 - 29 | % | 0.626 | 0.086 | 4.542 | 0.644 |
|  | ≥ 30 | % | 0.227 | 0.031 | 1.653 | 0.143 |
|  | Site of Infection |  |  |  |  |  |
|  | Urinary tract | % | 0.223 | 0.052 | 0.956 | 0.043 |
|  | Abdominal | % | 0.757 | 0.284 | 2.020 | 0.579 |
|  | Skin or cutaneous sites | % | 2.288 | 0.614 | 8.522 | 0.217 |
|  | Completion of the sepsis bundle within 3 hours | % | 1.820 | 0.741 | 4.470 | 0.191 |
|  | Completion of the administration of antibiotics within 3 hours | % | 0.182 | 0.058 | 0.572 | 0.004 |
|  | Respiratory support |  |  |  |  |  |
|  | Mechanical ventilation | % | 3.937 | 1.293 | 11.982 | 0.016 |
|  | High-flow nasal oxygen | % | 0.165 | 0.031 | 0.872 | 0.034 |
|  | Renal replacement therapy | % | 3.542 | 1.397 | 8.978 | 0.008 |
|  | Red blood cell transfusion | % | .724 | 0.310 | 1.689 | 0.454 |
|  | Fresh frozen plasma transfusion | % | 1.289 | 0.499 | 3.329 | 0.601 |
|  | Surgical source control | % | 0.673 | 0.173 | 2.610 | 0.566 |
|  | Non-surgical source control | % | 0.286 | 0.114 | 0.716 | 0.008 |
|  | Constant |  | 1.046 |  |  | 0.967 |
| 9 | University affiliation |  |  |  |  |  |
|  | No | % | - | - | - | - |
|  | Yes | % | 2.011 | 0.790 | 5.119 | 0.143 |
|  | Intensivist to patient ratio |  |  |  |  |  |
|  | 1 intensivist : 5 or fewer patients | % | - | - | - | 0.265 |
|  | 1 intensivist : 6 to 8 patients | % | 2.710 | 0.808 | 9.087 | 0.106 |
|  | 1 intensivist : 12 or more patients | % | 1.359 | 0.230 | 8.038 | 0.735 |
|  | Training programme in ICU |  |  |  |  |  |
|  | No | % | - | - | - | - |
|  | Yes | % | 0.220 | 0.077 | 0.632 | 0.005 |
|  | Comorbidities |  |  |  |  |  |
|  | Cardiovascular disease | % | 1.741 | 0.741 | 4.094 | 0.203 |
|  | Chronic neurological disease | % | 0.677 | 0.182 | 2.515 | 0.561 |
|  | Solid malignant tumours | % | 6.221 | 1.412 | 27.416 | 0.016 |
|  | SOFA |  |  |  |  |  |
|  | 0 - 3 | % | - | - | - | 0.037 |
|  | 4-7 | % | 1.341 | 0.361 | 4.981 | 0.661 |
|  | 8-9 | % | 2.490 | 0.506 | 12.252 | 0.262 |
|  | 10 - 11 | % | 3.352 | 0.751 | 14.966 | 0.113 |
|  | ≥12 | % | 9.570 | 1.855 | 49.369 | 0.007 |
|  | APACHE II |  |  |  |  |  |
|  | 0 - 9 | % | - | - | - | 0.411 |
|  | 10 - 14 | % | 0.253 | 0.047 | 1.371 | 0.111 |
|  | 15 - 19 | % | 0.389 | 0.073 | 2.079 | 0.269 |
|  | 20 - 24 | % | 0.513 | 0.092 | 2.858 | 0.446 |
|  | 25 - 29 | % | 0.614 | 0.085 | 4.445 | 0.629 |
|  | ≥ 30 | % | 0.224 | 0.031 | 1.618 | 0.138 |
|  | Site of Infection |  |  |  |  |  |
|  | Urinary tract | % | 0.239 | 0.058 | 0.990 | 0.048 |
|  | Abdominal | % | 0.777 | 0.293 | 2.062 | 0.612 |
|  | Skin or cutaneous sites | % | 2.282 | 0.613 | 8.497 | 0.219 |
|  | Completion of the sepsis bundle within 3 hours | % | 1.813 | 0.739 | 4.449 | 0.194 |
|  | Completion of the administration of antibiotics within 3 hours | % | 0.180 | 0.058 | 0.565 | 0.003 |
|  | Respiratory support |  |  |  |  |  |
|  | Mechanical ventilation | % | 4.002 | 1.315 | 12.176 | 0.015 |
|  | High-flow nasal oxygen | % | 0.163 | 0.031 | 0.856 | 0.032 |
|  | Renal replacement therapy | % | 3.581 | 1.417 | 9.050 | 0.007 |
|  | Red blood cell transfusion | % | 0.762 | 0.334 | 1.741 | 0.520 |
|  | Surgical source control | % | 0.659 | 0.169 | 2.565 | 0.548 |
|  | Non-surgical source control | % | 0.292 | 0.117 | 0.729 | 0.008 |
|  | Constant |  | 1.033 |  |  | 0.976 |
| 10 | University affiliation |  |  |  |  |  |
|  | No | % | - | - | - | - |
|  | Yes | % | 1.982 | 0.780 | 5.033 | 0.150 |
|  | Intensivist to patient ratio |  |  |  |  |  |
|  | 1 intensivist : 5 or fewer patients | % | - | - | - | 0.280 |
|  | 1 intensivist : 6 to 8 patients | % | 2.643 | 0.792 | 8.820 | 0.114 |
|  | 1 intensivist : 12 or more patients | % | 1.337 | 0.224 | 7.965 | 0.750 |
|  | Training programme in ICU |  |  |  |  |  |
|  | No | % | - | - | - | - |
|  | Yes | % | 0.214 | 0.075 | 0.613 | 0.004 |
|  | Comorbidities |  |  |  |  |  |
|  | Cardiovascular disease | % | 1.830 | 0.793 | 4.221 | 0.156 |
|  | Chronic neurological disease | % | 0.681 | 0.182 | 2.543 | 0.568 |
|  | Solid malignant tumours | % | 6.292 | 1.442 | 27.450 | 0.014 |
|  | SOFA |  |  |  |  |  |
|  | 0 - 3 | % | - | - | - | 0.041 |
|  | 4-7 | % | 1.276 | 0.351 | 4.642 | 0.712 |
|  | 8-9 | % | 2.410 | 0.495 | 11.744 | 0.276 |
|  | 10 - 11 | % | 3.112 | 0.721 | 13.424 | 0.128 |
|  | ≥12 | % | 8.879 | 1.776 | 44.393 | 0.008 |
|  | APACHE II |  |  |  |  |  |
|  | 0 - 9 | % | - | - | - | 0.423 |
|  | 10 - 14 | % | 0.267 | 0.050 | 1.415 | 0.121 |
|  | 15 - 19 | % | 0.401 | 0.076 | 2.119 | 0.282 |
|  | 20 - 24 | % | 0.532 | 0.097 | 2.934 | 0.469 |
|  | 25 - 29 | % | 0.641 | 0.090 | 4.585 | 0.658 |
|  | ≥ 30 | % | 0.231 | 0.032 | 1.657 | 0.145 |
|  | Site of Infection |  |  |  |  |  |
|  | Urinary tract | % | 0.263 | 0.067 | 1.037 | 0.056 |
|  | Skin or cutaneous sites | % | 2.394 | 0.648 | 8.842 | 0.190 |
|  | Completion of the sepsis bundle within 3 hours | % | 1.810 | 0.739 | 4.436 | 0.194 |
|  | Completion of the administration of antibiotics within 3 hours | % | 0.182 | 0.058 | 0.569 | 0.003 |
|  | Respiratory support |  |  |  |  |  |
|  | Mechanical ventilation | % | 4.027 | 1.326 | 12.226 | 0.014 |
|  | High-flow nasal oxygen | % | 0.172 | 0.033 | 0.890 | 0.036 |
|  | Renal replacement therapy | % | 3.335 | 1.384 | 8.035 | 0.007 |
|  | Red blood cell transfusion | % | 0.773 | 0.339 | 1.762 | 0.540 |
|  | Surgical source control | % | 0.601 | 0.162 | 2.228 | 0.446 |
|  | Non-surgical source control | % | 0.300 | 0.121 | 0.744 | 0.009 |
|  | Constant |  | 0.997 |  |  | 0.997 |
| 11 | University affiliation |  |  |  |  |  |
|  | No | % | - | - | - | - |
|  | Yes | % | 2.124 | 0.860 | 5.247 | 0.103 |
|  | Intensivist to patient ratio |  |  |  |  |  |
|  | 1 intensivist : 5 or fewer patients | % | - | - | - | 0.264 |
|  | 1 intensivist : 6 to 8 patients | % | 2.697 | 0.805 | 9.029 | 0.108 |
|  | 1 intensivist : 12 or more patients | % | 1.294 | 0.218 | 7.686 | 0.776 |
|  | Training programme in ICU |  |  |  |  |  |
|  | No | % | - | - | - | - |
|  | Yes | % | 0.219 | 0.077 | .620 | 0.004 |
|  | Comorbidities |  |  |  |  |  |
|  | Cardiovascular disease | % | 1.747 | 0.772 | 3.952 | 0.181 |
|  | Solid malignant tumours | % | 6.650 | 1.541 | 28.697 | 0.011 |
|  | SOFA |  |  |  |  |  |
|  | 0 - 3 | % | - | - | - | 0.032 |
|  | 4-7 | % | 1.260 | 0.347 | 4.581 | 0.726 |
|  | 8-9 | % | 2.303 | 0.479 | 11.068 | 0.298 |
|  | 10 - 11 | % | 3.063 | 0.712 | 13.183 | 0.133 |
|  | ≥12 | % | 9.239 | 1.860 | 45.905 | 0.007 |
|  | APACHE II |  |  |  |  |  |
|  | 0 - 9 | % | - | - | - | 0.433 |
|  | 10 - 14 | % | 0.256 | 0.048 | 1.356 | 0.109 |
|  | 15 - 19 | % | 0.385 | 0.073 | 2.028 | 0.260 |
|  | 20 - 24 | % | 0.498 | 0.091 | 2.713 | 0.420 |
|  | 25 - 29 | % | 0.574 | 0.083 | 3.962 | 0.573 |
|  | ≥ 30 | % | 0.218 | 0.031 | 1.554 | 0.129 |
|  | Site of Infection |  |  |  |  |  |
|  | Urinary tract | % | 0.254 | 0.065 | 0.993 | 0.049 |
|  | Skin or cutaneous sites | % | 2.522 | 0.686 | 9.274 | 0.164 |
|  | Completion of the sepsis bundle within 3 hours | % | 1.840 | 0.755 | 4.484 | 0.180 |
|  | Completion of the administration of antibiotics within 3 hours | % | 0.183 | 0.059 | 0.572 | 0.003 |
|  | Respiratory support |  |  |  |  |  |
|  | Mechanical ventilation | % | 3.939 | 1.307 | 11.869 | 0.015 |
|  | High-flow nasal oxygen | % | 0.167 | 0.032 | 0.861 | 0.032 |
|  | Renal replacement therapy | % | 3.498 | 1.472 | 8.310 | 0.005 |
|  | Red blood cell transfusion | % | 0.756 | 0.332 | 1.721 | 0.505 |
|  | Surgical source control | % | 0.618 | 0.167 | 2.294 | 0.472 |
|  | Non-surgical source control | % | 0.291 | 0.118 | 0.718 | 0.007 |
|  | Constant |  | 0.968 |  |  | 0.976 |
| 12 | University affiliation |  |  |  |  |  |
|  | No | % | - | - | - | - |
|  | Yes | % | 2.093 | 0.849 | 5.159 | 0.108 |
|  | Intensivist to patient ratio |  |  |  |  |  |
|  | 1 intensivist : 5 or fewer patients | % | - | - | - | 0.281 |
|  | 1 intensivist : 6 to 8 patients | % | 2.633 | 0.783 | 8.849 | 0.118 |
|  | 1 intensivist : 12 or more patients | % | 1.258 | 0.211 | 7.502 | 0.801 |
|  | Training programme in ICU |  |  |  |  |  |
|  | No | % | - | - | - | - |
|  | Yes | % | 0.220 | 0.078 | 0.623 | 0.004 |
|  | Comorbidities |  |  |  |  |  |
|  | Cardiovascular disease | % | 1.769 | 0.784 | 3.993 | 0.170 |
|  | Solid malignant tumours | % | 6.288 | 1.480 | 26.711 | 0.013 |
|  | SOFA |  |  |  |  |  |
|  | 0 - 3 | % | - | - | - | 0.034 |
|  | 4-7 | % | 1.202 | 0.331 | 4.368 | 0.780 |
|  | 8-9 | % | 2.304 | 0.479 | 11.094 | 0.298 |
|  | 10 - 11 | % | 2.972 | 0.691 | 12.771 | 0.143 |
|  | ≥12 | % | 8.776 | 1.781 | 43.237 | 0.008 |
|  | APACHE II |  |  |  |  |  |
|  | 0 - 9 | % | - | - | - | 0.401 |
|  | 10 - 14 | % | 0.249 | 0.047 | 1.312 | 0.101 |
|  | 15 - 19 | % | 0.394 | 0.075 | 2.070 | 0.271 |
|  | 20 - 24 | % | 0.504 | 0.093 | 2.741 | 0.428 |
|  | 25 - 29 | % | 0.602 | 0.088 | 4.123 | 0.605 |
|  | ≥ 30 | % | 0.224 | 0.032 | 1.584 | 0.134 |
|  | Site of Infection |  |  |  |  |  |
|  | Urinary tract | % | 0.259 | 0.067 | 0.996 | 0.049 |
|  | Skin or cutaneous sites | % | 2.544 | 0.696 | 9.302 | 0.158 |
|  | Completion of the sepsis bundle within 3 hours | % | 1.853 | 0.761 | 4.512 | 0.174 |
|  | Completion of the administration of antibiotics within 3 hours | % | 0.188 | 0.061 | 0.581 | 0.004 |
|  | Respiratory support |  |  |  |  |  |
|  | Mechanical ventilation | % | 3.711 | 1.250 | 11.016 | 0.018 |
|  | High-flow nasal oxygen | % | 0.174 | 0.034 | 0.891 | 0.036 |
|  | Renal replacement therapy | % | 3.183 | 1.405 | 7.211 | 0.006 |
|  | Surgical source control | % | 0.591 | 0.162 | 2.163 | 0.427 |
|  | Non-surgical source control | % | 0.288 | 0.117 | 0.709 | 0.007 |
|  | Constant |  | 0.959 |  |  | 0.968 |
| 13 | University affiliation |  |  |  |  |  |
|  | No | % | - | - | - | - |
|  | Yes | % | 2.029 | 0.829 | 4.963 | 0.121 |
|  | Intensivist to patient ratio |  |  |  |  |  |
|  | 1 intensivist : 5 or fewer patients | % | - | - | - | 0.261 |
|  | 1 intensivist : 6 to 8 patients | % | 2.722 | 0.809 | 9.155 | 0.106 |
|  | 1 intensivist : 12 or more patients | % | 1.295 | 0.221 | 7.593 | 0.775 |
|  | Training programme in ICU |  |  |  |  |  |
|  | No | % | - | - | - | - |
|  | Yes | % | .214 | 0.076 | 0.606 | 0.004 |
|  | Comorbidities |  |  |  |  |  |
|  | Cardiovascular disease | % | 1.805 | 0.802 | 4.059 | 0.153 |
|  | Solid malignant tumours | % | 6.304 | 1.498 | 26.535 | 0.012 |
|  | SOFA |  |  |  |  |  |
|  | 0 - 3 | % | - | - | - | 0.035 |
|  | 4-7 | % | 1.195 | 0.328 | 4.349 | 0.787 |
|  | 8-9 | % | 2.290 | 0.479 | 10.953 | 0.299 |
|  | 10 - 11 | % | 2.939 | 0.686 | 12.601 | 0.147 |
|  | ≥12 | % | 8.672 | 1.763 | 42.647 | 0.008 |
|  | APACHE II |  |  |  |  |  |
|  | 0 - 9 | % | - | - | - | 0.356 |
|  | 10 - 14 | % | 0.232 | 0.044 | 1.216 | 0.084 |
|  | 15 - 19 | % | 0.379 | 0.072 | 1.983 | 0.250 |
|  | 20 - 24 | % | 0.505 | 0.093 | 2.748 | 0.429 |
|  | 25 - 29 | % | 0.574 | 0.084 | 3.896 | 0.570 |
|  | ≥ 30 | % | 0.217 | 0.030 | 1.547 | 0.127 |
|  | Site of Infection |  |  |  |  |  |
|  | Urinary tract | % | 0.256 | 0.067 | 0.972 | 0.045 |
|  | Skin or cutaneous sites | % | 2.449 | 0.681 | 8.810 | 0.170 |
|  | Completion of the sepsis bundle within 3 hours | % | 1.896 | 0.782 | 4.598 | 0.157 |
|  | Completion of the administration of antibiotics within 3 hours | % | 0.192 | 0.063 | 0.589 | 0.004 |
|  | Respiratory support |  |  |  |  |  |
|  | Mechanical ventilation | % | 3.733 | 1.255 | 11.106 | 0.018 |
|  | High-flow nasal oxygen | % | 0.183 | 0.036 | 0.924 | 0.040 |
|  | Renal replacement therapy | % | 3.296 | 1.460 | 7.439 | 0.004 |
|  | Non-surgical source control | % | 0.292 | 0.119 | 0.716 | 0.007 |
|  | Constant |  | 0.917 |  |  | 0.935 |
| 14 | University affiliation |  |  |  |  |  |
|  | No | % | - | - | - | - |
|  | Yes | % | 1.847 | 0.786 | 4.343 | 0.159 |
|  | Intensivist to patient ratio |  |  |  |  |  |
|  | 1 intensivist : 5 or fewer patients | % | - | - | - | 0.129 |
|  | 1 intensivist : 6 to 8 patients | % | 3.193 | 1.033 | 9.871 | 0.044 |
|  | 1 intensivist : 12 or more patients | % | 1.463 | 0.284 | 7.547 | 0.649 |
|  | Training programme in ICU |  |  |  |  |  |
|  | No | % | - | - | - | - |
|  | Yes | % | 0.245 | 0.090 | 0.667 | 0.006 |
|  | Comorbidities |  |  |  |  |  |
|  | Cardiovascular disease | % | 1.901 | 0.855 | 4.227 | 0.115 |
|  | Solid malignant tumours | % | 5.848 | 1.340 | 25.528 | 0.019 |
|  | SOFA |  |  |  |  |  |
|  | 0 - 3 | % | - | - | - | 0.010 |
|  | 4-7 | % | 1.039 | 0.307 | 3.513 | 0.952 |
|  | 8-9 | % | 2.241 | 0.512 | 9.812 | 0.284 |
|  | 10 - 11 | % | 2.699 | 0.668 | 10.906 | 0.164 |
|  | ≥12 | % | 8.265 | 1.934 | 35.314 | 0.004 |
|  | Site of Infection |  |  |  |  |  |
|  | Urinary tract | % | 0.234 | 0.063 | 0.875 | 0.031 |
|  | Skin or cutaneous sites | % | 3.219 | 0.944 | 10.980 | 0.062 |
|  | Completion of the sepsis bundle within 3 hours | % | 1.911 | 0.797 | 4.580 | 0.147 |
|  | Completion of the administration of antibiotics within 3 hours | % | 0.221 | 0.074 | 0.665 | 0.007 |
|  | Respiratory support |  |  |  |  |  |
|  | Mechanical ventilation | % | 3.626 | 1.278 | 10.289 | 0.015 |
|  | High-flow nasal oxygen | % | 0.165 | 0.031 | 0.882 | 0.035 |
|  | Renal replacement therapy | % | 2.932 | 1.344 | 6.398 | 0.007 |
|  | Non-surgical source control | % | 0.284 | 0.118 | 0.682 | 0.005 |
|  | Constant |  | 0.335 |  |  | 0.220 |
| 15 | Intensivist to patient ratio |  |  |  |  |  |
|  | 1 intensivist : 5 or fewer patients | % | - | - | - | 0.081 |
|  | 1 intensivist : 6 to 8 patients | % | 3.504 | 1.165 | 10.540 | 0.026 |
|  | 1 intensivist : 12 or more patients | % | 1.954 | 0.397 | 9.609 | 0.410 |
|  | Training programme in ICU |  |  |  |  |  |
|  | No | % | - | - | - | - |
|  | Yes | % | 0.311 | 0.121 | 0.798 | 0.015 |
|  | Comorbidities |  |  |  |  |  |
|  | Cardiovascular disease | % | 1.742 | 0.798 | 3.805 | 0.164 |
|  | Solid malignant tumours | % | 5.142 | 1.167 | 22.658 | 0.030 |
|  | SOFA |  |  |  |  |  |
|  | 0 - 3 | % | - | - | - | 0.004 |
|  | 4-7 | % | 1.068 | 0.322 | 3.545 | 0.914 |
|  | 8-9 | % | 2.602 | 0.619 | 10.936 | 0.192 |
|  | 10 - 11 | % | 3.123 | 0.798 | 12.233 | 0.102 |
|  | ≥12 | % | 9.531 | 2.288 | 39.693 | 0.002 |
|  | Site of Infection |  |  |  |  |  |
|  | Urinary tract | % | 0.254 | 0.068 | 0.947 | 0.041 |
|  | Skin or cutaneous sites | % | 3.331 | 0.970 | 11.439 | 0.056 |
|  | Completion of the sepsis bundle within 3 hours | % | 1.965 | 0.822 | 4.696 | 0.129 |
|  | Completion of the administration of antibiotics within 3 hours | % | 0.194 | 0.065 | 0.581 | 0.003 |
|  | Respiratory support |  |  |  |  |  |
|  | Mechanical ventilation | % | 4.178 | 1.517 | 11.509 | 0.006 |
|  | High-flow nasal oxygen | % | 0.157 | 0.030 | 0.827 | 0.029 |
|  | Renal replacement therapy | % | 2.913 | 1.338 | 6.343 | 0.007 |
|  | Non-surgical source control | % | 0.268 | 0.113 | 0.636 | 0.003 |
|  | Constant |  | 0.335 |  |  | 0.220 |
| 16 | Intensivist to patient ratio |  |  |  |  |  |
|  | 1 intensivist : 5 or fewer patients | % | - | - | - | 0.083 |
|  | 1 intensivist : 6 to 8 patients | % | 3.406 | 1.155 | 10.046 | 0.026 |
|  | 1 intensivist : 12 or more patients | % | 1.925 | 0.388 | 9.546 | 0.423 |
|  | Training programme in ICU |  |  |  |  |  |
|  | No | % | - | - | - | - |
|  | Yes | % | 0.274 | 0.110 | 0.682 | 0.005 |
|  | Comorbidities |  |  |  |  |  |
|  | Solid malignant tumours | % | 4.517 | 1.069 | 19.083 | 0.040 |
|  | SOFA |  |  |  |  |  |
|  | 0 - 3 | % | - | - | - | 0.005 |
|  | 4-7 | % | 1.108 | 0.336 | 3.656 | 0.866 |
|  | 8-9 | % | 2.550 | 0.612 | 10.620 | 0.198 |
|  | 10 - 11 | % | 2.904 | 0.749 | 11.264 | 0.123 |
|  | ≥12 | % | 9.454 | 2.285 | 39.110 | 0.002 |
|  | Site of Infection |  |  |  |  |  |
|  | Urinary tract | % | 0.229 | 0.063 | 0.841 | 0.026 |
|  | Skin or cutaneous sites | % | 3.193 | 0.925 | 11.028 | 0.066 |
|  | Completion of the sepsis bundle within 3 hours | % | 1.984 | 0.835 | 4.713 | 0.121 |
|  | Completion of the administration of antibiotics within 3 hours | % | 0.182 | 0.061 | 0.548 | 0.002 |
|  | Respiratory support |  |  |  |  |  |
|  | Mechanical ventilation | % | 4.178 | 1.529 | 11.413 | 0.005 |
|  | High-flow nasal oxygen | % | 0.169 | 0.033 | 0.855 | 0.032 |
|  | Renal replacement therapy | % | 2.970 | 1.373 | 6.424 | 0.006 |
|  | Non-surgical source control | % | 0.288 | 0.123 | 0.674 | 0.004 |
|  | Constant |  | 0.464 |  |  | 0.366 |
| 17 | Intensivist to patient ratio |  |  |  |  |  |
|  | 1 intensivist : 5 or fewer patients | % | - | - | - | 0.012 |
|  | 1 intensivist : 6 to 8 patients | % | 4.533 | 1.621 | 12.677 | 0.004 |
|  | 1 intensivist : 12 or more patients | % | 2.748 | 0.591 | 12.768 | 0.197 |
|  | Training programme in ICU |  |  |  |  |  |
|  | No | % | - | - | - | - |
|  | Yes | % | 0.274 | 0.111 | 0.672 | 0.005 |
|  | Comorbidities |  |  |  |  |  |
|  | Solid malignant tumours | % | 4.695 | 1.073 | 20.543 | 0.040 |
|  | SOFA |  |  |  |  |  |
|  | 0 - 3 | % | - | - | - | 0.005 |
|  | 4-7 | % | 1.278 | 0.393 | 4.156 | 0.684 |
|  | 8-9 | % | 3.179 | 0.779 | 12.966 | 0.107 |
|  | 10 - 11 | % | 3.664 | 0.975 | 13.766 | 0.054 |
|  | ≥12 | % | 9.800 | 2.374 | 40.455 | 0.002 |
|  | Site of Infection |  |  |  |  |  |
|  | Urinary tract | % | 0.252 | 0.070 | 0.910 | 0.035 |
|  | Skin or cutaneous sites | % | 3.028 | 0.876 | 10.468 | 0.080 |
|  | Completion of the administration of antibiotics within 3 hours | % | 0.258 | 0.096 | 0.694 | 0.007 |
|  | Respiratory support |  |  |  |  |  |
|  | Mechanical ventilation | % | 3.890 | 1.445 | 10.474 | 0.007 |
|  | High-flow nasal oxygen | % | 0.144 | 0.029 | 0.727 | 0.019 |
|  | Renal replacement therapy | % | 2.816 | 1.318 | 6.016 | 0.008 |
|  | Non-surgical source control | % | 0.292 | 0.126 | 0.678 | 0.004 |
|  | Constant |  | 0.408 |  |  | 0.287 |

**Table S21.** Hospital and intensive care unit characteristics according to hospital survivability of patients with septic shock

| Variable | All cases  n=74 | Survived  n=35 | Died  n=39 | P |
| --- | --- | --- | --- | --- |
| Participating hospital, no. (%) |  |  |  | 0.129 |
| 115 People's | 12 (16.2) | 2 (5.7) | 10 (25.6) |  |
| Bach Mai | 5 (6.8) | 3 (8.6) | 2 (5.1) |  |
| Bai Chay | 1 (1.4) | 1 (2.9) | 0 (0.0) |  |
| Can Tho | 4 (5.4) | 0 (0.0) | 4 (10.3) |  |
| Cho Ray | 18 (24.3) | 9 (25.7) | 9 (23.1) |  |
| Da Nang | 6 (8.1) | 3 (8.6) | 3 (7.7) |  |
| Dong Da | 1 (1.4) | 1 (2.9) | 0 (0.0) |  |
| Hanoi Medical University | 2 (2.7) | 2 (5.7) | 0 (0.0) |  |
| Hue | 8 (10.8) | 4 (11.4) | 4 (10.3) |  |
| Saint Paul | 0 | 0 | 0 |  |
| Thai Nguyen | 2 (2.7) | 1 (2.9) | 1 (2.6) |  |
| Thanh Nhan | 0 | 0 | 0 |  |
| Vietnam–Czechoslovakia Friendship | 15 (20.3) | 9 (25.7) | 6 (15.4) |  |
| Vinmec Times City International | 0 | 0 | 0 |  |
| **Hospital characteristics** | | | | |
| Type of hospital, no. (%) |  |  |  | - |
| Rural | 0 | 0 | 0 |  |
| Urban | 74 (100) | 35 (100) | 39 (100) |  |
| University affiliation, no. (%) |  |  |  | 0.835 |
| No | 39 (52.7) | 18 (51.4) | 21 (53.8) |  |
| Yes | 35 (47.3) | 17 (48.6) | 18 (46.2) |  |
| **ICU characteristics** | | | | |
| Nature of ICU, no. (%) |  |  |  | - |
| Open | 0 | 0 | 0 |  |
| Closed | 74 (100) | 35 (100) | 39 (100) |  |
| Type of ICU, no. (%) |  |  |  | 0.166 |
| Medical | 38 (51.4) | 15 (42.9) | 23 (59.0) |  |
| Surgical | 0 | 0 | 0 |  |
| Mixed | 36 (48.6) | 20 (57.1) | 16 (41.0) |  |
| Nurse to patient ratio, no. (%) |  |  |  | 0.411 |
| 1 or more nurses : 1 patient | 0 | 0 | 0 |  |
| 1 nurse : 2 patients | 56 (75.7) | 28 (80.0) | 28 (71.8) |  |
| 1 nurse : 3 patients | 0 | 0 | 0 |  |
| 1 nurse : 4 or more patients | 18 (24.3) | 7 (20.0) | 11 (28.2) |  |
| Intensivist to patient ratio, no. (%) |  |  |  | 0.922 |
| 1 intensivist : 5 or fewer patients | 55 (74.3) | 25 (71.4) | 30 (76.9) |  |
| 1 intensivist : 6 to 8 patients | 13 (17.6) | 7 (20.0) | 6 (15.4) |  |
| 1 intensivist : 9 to 11 patients | 0 | 0 | 0 |  |
| 1 intensivist : 12 or more patients | 6 (8.1) | 3 (8.6) | 3 (7.7) |  |
| Training programme in ICU, no. (%) |  |  |  | 0.005 |
| No | 17 (23.0) | 3 (8.6) | 14 (35.9) |  |
| Yes | 57 (77.0) | 32 (91.4) | 25 (64.1) |  |

**Table S22.** Baseline characteristics according to hospital survivability of patients with septic shock

| Variable | All cases  n= 74 | Survived  n= 35 | Died  n= 39 | P |
| --- | --- | --- | --- | --- |
| Age (year), median (IQR) | 64 (47.75-77) | 66 (53-76) | 63 (46-82) | 0.841 |
| Age (year), no. (%) |  |  |  | 0.727 |
| < 20 | 1 (1.4) | 0 (0.0) | 1 (2.6) |  |
| 20 - 39 | 7 (9.5) | 3 (8.6) | 4 (10.3) |  |
| 40 - 59 | 22 (29.7) | 9 (25.7) | 33 (33.3) |  |
| ≥ 60 | 44 (59.5) | 23 (65.7) | 21 (53.8) |  |
| Sex (male), no. (%) | 41 (55.4) | 18 (51.4) | 23 (59.0) | 0.514 |
| Collection batch, no. (%) |  |  |  | 0.008 |
| Collection 1 (Jan) | 24 (32.4) | 18 (51.4) | 6 (15.4) |  |
| Collection 2 (April) | 22 (29.7) | 6 (17.1) | 16 (41.0) |  |
| Collection 3 (July) | 16 (21.6) | 6 (17.1) | 10 (25.6) |  |
| Collection 4 (Oct) | 12 (16.2) | 5 (14.3) | 7 (17.9) |  |
| Admission type, no. (%) |  |  |  | 0.183 |
| Medical | 69 (93.2) | 31 (88.6) | 38 (97.4) |  |
| Elective surgical | 0 | 0 | 0 |  |
| Unscheduled surgical | 5 (6.8) | 4 (11.4) | 1 (2.6) |  |
| Admission source, no. (%) |  |  |  | 0.945 |
| Emergency department | 41 (55.4) | 18 (51.4) | 23 (59.0) |  |
| Operating room | 2 (2.7) | 1 (2.9) | 1 (2.6) |  |
| General wards | 12 (16.2) | 6 (17.1) | 6 (15.4) |  |
| Other ICUs or HDU | 4 (5.4) | 2 (5.7) | 2 (5.1) |  |
| Inter-hospital transfer | 14 (18.9) | 8 (22.9) | 6 (15.4) |  |
| Others | 1 (1.4) | 0 (0.0) | 1 (2.6) |  |
| Comorbidities, no. (%) |  |  |  |  |
| Cardiovascular disease | 20 (27.0) | 7 (20.0) | 13 (33.3) | 0.197 |
| Chronic lung disease | 5 (6.8) | 3 (8.6) | 2 (5.1) | 0.662 |
| Chronic neurological disease | 5 (6.8) | 5 (14.3) | 0 (0.0) | 0.020 |
| Chronic kidney disease | 7 (9.5) | 3 (8.6) | 4 (10.3) | <0.999 |
| Peptic ulcer disease | 3 (4.1) | 1 (2.9) | 2 (5.1) | <0.999 |
| Chronic liver disease | 7 (9.5) | 1 (2.9) | 6 (15.4) | 0.111 |
| Diabetes mellitus | 19 (25.7) | 9 (25.7) | 10 (25.6) | 0.994 |
| HIV infection | 0 | 0 | 0 |  |
| Connective tissue disease | 0 | 0 | 0 |  |
| Immunosuppression | 5 (6.8) | 3 (8.6) | 2 (5.1) | 0.662 |
| Haematological malignancies | 2 (2.7) | 1 (2.9) | 1 (2.6) | >0.999 |
| Solid malignant tumours | 2 (2.7) | 1 (2.9) | 1 (2.6) | >0.999 |

**Table S23.** Clinical and laboratory characteristics and severity of illness according to hospital survivability of patients with septic shock

| Variable | All cases  n= 74 | Survived  n= 35 | Died  n= 39 | P |
| --- | --- | --- | --- | --- |
| **Vital signs** (on admission into ICU) | | | | |
| GCS, median (IQR) | 11 (7.5-14.5) | 12.5 (9-15) | 9 (7-13) | 0.016 |
| HR (beats per min), median (IQR) | 120 (100-130.5) | 112 (90-128) | 124 (110-134) | 0.020 |
| Temperature (^o^C), mean (SD) | 37.74 (1.01) | 37.47 (1.04) | 37.98 (0.94) | 0.025 |
| MBP (mmHg), mean(SD) | 53.45 (10.08) | 55.03 (6.67) | 52.03 (12.29) | 0.312 |
| SBP (mmHg), mean (SD) | 78.15 (18.22) | 80.06 (13.40) | 76.44 (21.70) | 0.481 |
| RR (breaths per min), median (IQR) | 25 (21-30) | 24 (20-30) | 25 (22-30 | 0.704 |
| **Blood investigations** | | | | |
| Total WBC (x10^9^/L), mean (SD) | 17.62 (10.09) | 19.73 (9.35) | 15.74 (10.46) | 0.039 |
| PLT (x10^9^/L), mean (SD) | 185.96 (147.33) | 224.37 (143.43) | 151.49 (143.93) | 0.003 |
| Hb (g/dL), mean (SD) | 11.37 (2.75) | 11.50 (2.92) | 11.25 (2.62) | 0.701 |
| Hct (%), mean (SD) | 35.04 (7.98) | 35.30 (8.70) | 34.82 (7.37) | 0.673 |
| K^+^ (mmol/L), mean (SD) | 3.91 (0.96) | 3.98 (0.90) | 3.85 (0.93) | 0.380 |
| Na^+^ (mmol/L), mean (SD) | 136.5 (7.88) | 137.71 (7.53) | 135.41 (8.12) | 0.340 |
| Creatinine (µmol/L), mean (SD) | 210.25 (145.01) | 205.06 (171.28) | 214.90 (118.73) | 0.183 |
| Bilirubin (µmol/l), mean (SD) | 31.64  (47.21) | 23.64 (38.80) | 38.99 (53.27) | 0.124 |
| pH, mean (SD) | 7.28 (0.14) | 7.32 (0.13) | 7.24 (0.14) | 0.008 |
| PaO_2_ (mmHg), mean (SD) | 118.67 (83.64) | 107.81 (55.73) | 127.86 (101.33) | 0.668 |
| FiO_2_, mean (SD) | 0.56 (0.25) | 0.47 (0.20) | 0.64 (0.26) | 0.004 |
| PaO_2_/FiO_2_ ratio, mean (SD) | 236.23 (144.23) | 241.10 (118.31) | 232.12  (164.45) | 0.452 |
| **Severity of illness scores** | | | | |
| qSOFA, median (IQR) | 2 (2-3) | 2 (2-3) | 3 (2-3) | 0.039 |
| qSOFA, no. (%) |  |  |  | 0.183 |
| 0 - 1 | 5 (6.8) | 4 (11.4) | 1 (2.6) |  |
| 2 - 3 | 69 (93.2) | 31 (88.6) | 38 (97.4) |  |
| SIRS, median (IQR) | 3 (2-4) | 3 (2-3) | 3 (2-4) | 0.140 |
| SOFA, median (IQR) | 10 (7-12.25) | 9 (7-10) | 11 (9-14) | 0.005 |
| SOFA, no. (%) |  |  |  | 0.006 |
| 0 - 1 | 0 | 0 | 0 |  |
| 2 - 3 | 5 (6.8) | 1 (2.9) | 4 (10.3) |  |
| 4 - 5 | 4 (5.4) | 4 (11.4) | 0 (0.0) |  |
| 6 - 7 | 14 (18.9) | 10 (28.6) | 4 (10.3) |  |
| 8 - 9 | 12 (16.2) | 8 (22.9) | 4 (10.3) |  |
| 10 - 11 | 16 (21.6) | 7 (20.0) | 9 (23.1) |  |
| 12 - 14 | 15 (20.3) | 4 (11.4) | 11 (28.2) |  |
| > 14 | 8 (10.8) | 1 (2.9) | 7 (17.9) |  |
| SOFA, no. (%) |  |  |  | 0.016 |
| 0 - 6 | 11 (14.9) | 6 (17.1) | 5 (12.8) |  |
| 7 - 9 | 24 (32.4) | 17 (48.6) | 7 (17.9) |  |
| 10 - 12 | 16 (21.6) | 7 (20.0) | 9 (23.1) |  |
| 13 - 14 | 15 (20.3) | 4 (11.4) | 11 (28.2) |  |
| > 14 | 8 (10.8) | 1 (2.9) | 7 (17.9) |  |
| SOFA |  |  |  | 0.003 |
| 0 - 3 | 5 (6.8) | 1 (2.9) | 4 (10.3) |  |
| 4 - 7 | 18 (24.3) | 14 (40.0) | 4 (10.3) |  |
| 8 - 9 | 12 (16.2) | 8 (22.9) | 4 (10.3) |  |
| 10 - 11 | 16 (21.6) | 7 (20.0) | 9 (23.1) |  |
| ≥ 12 | 23(31.1) | 5 (14.3) | 18 (46.2) |  |
| APACHE II, median (IQR) | 23 (16.75-28) | 20 (14-24) | 26 (22-31) | 0.001 |
| APACHE II, no. (%) |  |  |  | 0.065 |
| 0 - 4 | 0 | 0 | 0 |  |
| 5 - 9 | 2 (2.7) | 2 (5.7) | 0 (0.0) |  |
| 10 - 14 | 12 (16.2) | 9 (25.7) | 3 (7.7) |  |
| 15 - 19 | 9 (12.2) | 6 (17.1) | 3 (7.7) |  |
| 20 - 24 | 22 (29.7) | 10 (28.6) | 12 (30.8) |  |
| 25 - 29 | 13 (17.6) | 4 (11.4) | 9 (23.1) |  |
| 30 - 34 | 9 (12.2) | 2 (5.7) | 7 (17.9) |  |
| > 34 | 7 (9.5) | 2 (5.7) | 5 (12.8) |  |
| APACHE II, no. (%) |  |  |  | 0.034 |
| 0 - 9 | 2 (2.7) | 2 (5.7) | 0 (0.0) |  |
| 10 - 14 | 12 (16.2) | 9 (25.7) | 3 (7.7) |  |
| 15 - 19 | 9 (12.2) | 6 (17.1) | 3 (7.7) |  |
| 20 - 24 | 22 (29.7) | 10 (28.6) | 12 (30.8) |  |
| 25 - 29 | 13 (17.6) | 4 (11.4) | 9 (23.1) |  |
| ≥ 30 | 16 (21.6) | 4 (11.4) | 12 (30.8) |  |

**Table S24.** Sites of infection and microbiology according to hospital survivability of patients with septic shock

| Variable | All cases  n= 74 | Survived  n= 35 | Died  n= 39 | P |
| --- | --- | --- | --- | --- |
| **Site of Infection** | | | | |
| Respiratory, no. (%) | 38 (51.4) | 16 (45.7) | 22 (56.4) | 0.358 |
| Urinary tract, no. (%) | 10 (13.5) | 7 (20.0) | 3 (7.7) | 0.176 |
| Abdominal, no. (%) | 26 (35.1) | 12 (34.3) | 14 (35.9) | 0.885 |
| Neurological, no. (%) | 2 (2.7) | 1 (2.9) | 1 (2.6) | >0.999 |
| Bones or joints, no. (%) | 1 (1.4) | 1 (2.9) | 0 (0.0) | 0.473 |
| Skin or cutaneous sites, no. (%) | 6 (8.1) | 2 (5.7) | 4 (10.3) | 0.677 |
| Intravascular catheter, no. (%) | 0 | 0 | 0 | - |
| Infective endocarditis, no. (%) | 0 | 0 | 0 | - |
| Primary bacteraemia, no. (%) | 2 (2.7) | 1 (2.9) | 1 (2.6) | >0.999 |
| Systemic, no. (%) | 3 (4.1) | 1 (2.9) | 2 (5.1) | >0.999 |
| Others, no. (%) | - | - | - | - |
| **Microbiology** | | | | |
| No pathogens detected, no. (%) | 23 (31.1) | 15 (42.9) | 8 (20.5) | 0.038 |
| Gram negative bacteria, no. (%) | 43 (58.1) | 18 (51.4) | 25 (64.1) | 0.270 |
| *Klebsiella pneumonia* | 6 (8.1) | 3 (8.6) | 3 (7.7) | >0.999 |
| *Acinetobacter baumannii* | 12 (16.2) | 4 (11.4) | 8 (20.5) | 0.290 |
| *Escherichia coli* | 16 (21.6) | 7 (20.0) | 9 (23.1) | 0.748 |
| *Pseudomonas aeruginosa* | 5 (6.8) | 2 (5.7) | 3 (7.7) | >0.999 |
| *Stenotrophomonas maltophilia* | 0 | 0 | 0 | - |
| *Proteus species* | 10 (13.5) | 4 (11.5) | 6 (15.4) | 0.740 |
| *Enterobacter cloacae* | 0 | 0 | 0 | - |
| *Bulkholderia pseudomallei* | 0 | 0 | 0 | - |
| *Others* | 0 | 0 | 0 | - |
| Gram positive bacteria, no. (%) | 6 (8.1) | 2 (5.7) | 4 (10.3) | 0.677 |
| *Enterococcus* | 1 (1.4) | 1 (2.9) | 0 (0.0) | 0.473 |
| *MSSA* | 0 | 0 | 0 | - |
| *MRSA* | 3 (4.1) | 1 (2.9) | 2 (5.1) | >0.999 |
| *Other Streptococcus species* | 2 (2.7) | 0 (0.0) | 2 (5.1) | 0.495 |
| *Streptococcus pneumonia* | 0 | 0 | 0 | - |
| Fungi, no. (%) | 0 | 0 | 0 | - |
| *Candida species* | 0 | 0 | 0 | - |
| *Aspergillus species* | 0 | 0 | 0 | - |
| *Others* | 0 | 0 | 0 | - |
| Viruses, no. (%) |  |  |  |  |
| *Influenza* | 1 (1.4) | 0 (0.0) | 1 (2.6) | >0.999 |
| *Others* | 0 | 0 | 0 | - |
| *Dengue* | 1 (1.4) | 0 (0.0) | 1 (2.6) | >0.999 |
| Other pathogens, no. (%) |  |  |  |  |
| *Anaerobes* | 0 | 0 | 0 | - |
| *Mycobacterium tuberculosis* | 2 (2.7) | 1 (2.9) | 1 (2.6) | >0.999 |
| *Malaria* | 0 | 0 | 0 | - |

**Table S25.** Completion of sepsis bundle elements according to hospital survivability of patients with septic shock

| Variable | All cases  n=74 | Survived  n= 35 | Died  n= 39 | P |
| --- | --- | --- | --- | --- |
| **Timing of antibiotics administration** | | | | |
| Performed within 24 hours, no. (%) | n=68 | n=32 | n=36 | 0.913 |
| 0-60 minutes | 51 (75.0) | 23 (71.9) | 28 (77.8) |  |
| 61-120 minutes | 10 (14.7) | 5 (15.6) | 5 (13.9) |  |
| 121-180 minutes | 2 (2.9) | 1 (3.1) | 1 (2.8) |  |
| >180 minutes | 5 (7.4) | 3 (9.4) | 2 (5.6) |  |
| Not performed within 24 hours, no. (%) | 0 | 0 | 0 | - |
| Timing of antibiotics administration, median (IQR), minutes | 30 (6.25-63.75) | 37.5 (0-76.75) | 30 (10-58.75) | 0.848 |
| **Timing of obtaining blood cultures** | | | | |
| Performed within 24 hours, no. (%) | n=54 | n=22 | n=32 | 0.588 |
| 0 - 60 minutes | 29 (53.7) | 10 (45.5) | 19 (59.4) |  |
| 61 - 120 minutes | 9 (16.7) | 4 (18.2) | 5 (15.6) |  |
| 121 - 180 minutes | 1 (1.9) | 0 (0.0) | 1 (3.1) |  |
| > 180 minutes | 15 (27.8) | 8 (36.4) | 7 (21.9) |  |
| Not performed within 24 hours, no. (%) | 0 | 0 | 0 | - |
| Timing of obtaining blood cultures, median (IQR), minutes | 55 (19.25-233.25) | 74.5 (30-366.75) | 30 (15-152.75) | 0.084 |
| **Timing of obtaining lactate measurement** | | | | |
| Performed within 24 hours, no. (%) | n=62 | n=30 | n=32 | 0.962 |
| 0 - 60 minutes | 38 (61.3) | 18 (60.0) | 20 (62.5) |  |
| 61 - 120 minutes | 5 (8.1) | 2 (6.7) | 3 (9.4) |  |
| 121 - 180 minutes | 1 (1.6) | 1 (3.3) | 0 (0.0) |  |
| > 180 minutes | 18 (29.0) | 9 (30.0) | 9 (28.1) |  |
| Not performed within 24 hours, no. (%) | 0 | 0 | 0 | - |
| Timing of obtaining lactate measurement, median (IQR), minutes | 30 (13-313.75) | 52.5 (15-317.75) | 22.5 (10.75-325.5) | 0.481 |
| **Timing of initiating vasopressors administration** | | | | |
| Performed within 24 hours, no. (%) | n=66 | n=31 | n=35 | 0.285 |
| 0 - 60 minutes | 48 (72.7) | 19 (61.3) | 29 (82.9) |  |
| 61 - 120 minutes | 7 (10.6) | 5 (16.1) | 2 (5.7) |  |
| 121 - 180 minutes | 5 (7.6) | 3 (9.7) | 2 (5.7) |  |
| > 180 minutes | 6 (9.1) | 4 (12.9) | 2 (5.7) |  |
| Not performed within 24 hours, no. (%) | 0 | 0 | 0 | - |

**Table S26.** Completion of the sepsis bundle of care and the initial administration of antibiotics according to hospital survivability of patients with septic shock

| Variable | All cases  n= 74 | Survived  n= 35 | Died  n= 39 | P |
| --- | --- | --- | --- | --- |
| Completion of the sepsis bundle within 1 hour, no. (%), n=72 | 20 (27.8) | 8 (22.9) | 12 (32.4) | 0.365 |
| Completion of the initial administration of antibiotics within 1 hour, no. (%), n=72 | 51 (70.8) | 23 (65.7) | 28 (75.7) | 0.353 |
| Permutations of the completed elements within 1 hour | n=72 | n=35 | n=37 | 0.628 |
| No elements completed | 9 (12.5) | 6 (17.1) | 3 (8.1) |  |
| Antibiotics only | 17 (23.6) | 9 (25.7) | 8 (21.6) |  |
| Blood cultures only | 1 (1.4) | 0 (0.0) | 1 (2.7) |  |
| Lactate only | 10 (13.9) | 6 (17.1) | 4 (10.8) |  |
| Antibiotics + Lactate | 7 (9.7) | 4 (11.4) | 3 (8.1) |  |
| Antibiotics + Blood cultures | 7 (9.7) | 2 (5.7) | 5 (13.5) |  |
| Blood cultures + Lactate | 1 (1.4) | 0 (0.0) | 1 (2.7) |  |
| Full bundle | 20 (27.8) | 8 (22.9) | 12 (32.4) |  |
| Vasopressors, no. (%) | 48/66 (72.7) | 19/31 (61.3) | 29/35 (82.9) | 0.050 |
| Volume of fluids administered (mL), median (IQR) | 1000 (500-1000) | 750 (500-1000) | 1000 (500-1000) | 0.795 |
| Completion of the sepsis bundle within 3 hours, no. (%), n=72 | 27 (37.5) | 11 (31.4) | 16 (43.2) | 0.301 |
| Completion of the initial administration of antibiotics within 3 hours, no. (%), n=72 | 63 (87.5) | 29 (82.9) | 34 (91.9) | 0.247 |
| Permutation of the completed elements of 3-hour sepsis bundle, no. (%) | n=72 | n=35 | n=37 | 0.127 |
| No elements completed | 2 (2.8) | 1 (2.9) | 1 (2.7) |  |
| Antibiotics only | 15 (20.8) | 10 (28.6) | 5 (13.5) |  |
| Blood cultures only | 1 (1.4) | 1 (2.9) | 0 (0.0) |  |
| Lactate only | 6 (8.3) | 4 (11.4) | 2 (5.4) |  |
| Antibiotics + Lactate | 10 (13.9) | 6 (17.1) | 4 (10.8) |  |
| Antibiotics + Blood cultures | 11 (15.3) | 2 (5.7) | 9 (24.3) |  |
| Blood cultures + Lactate | 0 | 0 | 0 |  |
| Full bundle | 27 (37.5) | 11 (31.4) | 16 (43.2) |  |
| Vasopressors, no. (%) | 60/66 (90.9) | 27/31 (87.1) | 33/35 (94.3) | 0.408 |
| Volume of fluids administered (mL), median (IQR) | 1500 (900-2000) | 1500 (600-2000) | 2000 (1225-2000) | 0.117 |

**Table S27.** Life-sustaining treatments during ICU stay and outcomes according to hospital survivability of patients with septic shock

| Variable | All cases  n= 74 | Survived  n= 35 | Died  n= 39 | P |
| --- | --- | --- | --- | --- |
| **Life-sustaining treatments during ICU stay** | | | | |
| Respiratory support, no. (%) and median (IQR), days |  |  |  |  |
| Mechanical ventilation | 61 (82.4) | 23 (65.7) | 38 (97.4) | <0.001 |
| Duration of mechanical ventilation | 7 (3-13) | 9 (4-15) | 6 (2-11.75) | 0.125 |
| Non-invasive ventilation | 4 (5.4) | 3 (8.6) | 1 (2.6) | 0.339 |
| Duration of non-invasive ventilation | 2 (1.25-5.75) | 2 (1-2) | 7 (7-7) | 0.157 |
| High-flow nasal oxygen | 11 (14.9) | 8 (22.9) | 3 (7.7) | 0.067 |
| Duration of high-flow nasal oxygen | 2 (1-3) | 2.5 (1.25-3.75) | 2 (1-2) | 0.529 |
| Additional ICU support, no. (%) |  |  |  |  |
| Vasopressors/inotropes | 74 (100) | 35 (100) | 39 (100) | - |
| Renal replacement therapy | 43 (58.1) | 13 (37.1) | 30 (76.9) | 0.001 |
| Red blood cell transfusion | 31 (41.9) | 11 (31.4) | 20 (51.3) | 0.084 |
| Platelet transfusion | 18 (24.3) | 5 (14.3) | 13 (33.3) | 0.057 |
| Fresh frozen plasma transfusion | 24 (32.4) | 6 (17.1) | 18 (46.2) | 0.008 |
| Surgical source control | 8 (10.8) | 6 (17.1) | 2 (5.1) | 0.139 |
| Non-surgical source control | 13 (17.6) | 7 (20.0) | 6 (15.4) | 0.602 |
| Length of of surgical source control, median minutes (IQR) | 270 (140-552.5) | 282.5 (78.75-791.25) | 270 (270-270) | >0.999 |
| Length of of surgical source control, n (%) |  |  |  | - |
| <12 hours | 8 (100) | 6 (100) | 2 (100) |  |
| 12-24 hours | 0 | 0 | 0 |  |
| >24 hours | 0 | 0 | 0 |  |
| **Outcomes** | | | | |
| Outcomes |  |  |  | <0.001 |
| Alive upon current hospital discharge, no. (%) | 35 (47.3) | 35 (100) | 0 (0.0) |  |
| Alive upon discharge from current ICU stay, but died in current hospital stay, no. (%) | 8 (10.8) | 0 (0.0) | 8 (20.5) |  |
| Alive upon discharge from current ICU stay, but still in current hospital stay after 90 days, no. (%) | 0 | 0 | 0 |  |
| Still in current ICU stay after 90 days, no. (%) | 0 | 0 | 0 |  |
| Died in current ICU stay, no. (%) | 31 (41.9) | 0 (0.0) | 31 (79.5) |  |
| Length of stay, median days (IQR) |  |  |  |  |
| Hospital | 14 (8-20) | 18 (12-22) | 11 (6-19) | 0.018 |
| ICU | 8 (5-15.25) | 8 (6-14) | 7 (3-18) | 0.536 |

**Table S28**. Clinical and laboratory characteristics and severity of illness according to methods of mechanical ventilation for patients with septic shock

| Variable | All cases  n=74 | No mechanical ventilation  n=13 | Mechanical ventilation  n=61 | P^a^ |
| --- | --- | --- | --- | --- |
| **Vital signs** (on admission into ICU) | | | | |
| GCS, median (IQR) | 11 (7.5-14.5) | 15 (14-15) | 10 (7-13.5) | <0.001 |
| HR (beats per min), median (IQR) | 120 (100-130.5) | 100 (88-117.5) | 120 (107-132.5) | 0.008 |
| Temperature (^o^C), mean (SD) | 37.74 (1.01) | 37.77 (0.83) | 37.73 (1.05) | 0.762 |
| MBP (mmHg), mean(SD) | 53.45 (10.08) | 54.62 (5.30) | 53.20 (10.84) | 0.780 |
| SBP (mmHg), mean (SD) | 78.15 (18.22) | 76.39 (8.01) | 78.53 (19.76) | 0.530 |
| RR (breaths per min), median (IQR) | 25 (21-30) | 24 (21.5-34) | 25 (21-31) | 0.776 |
| **Blood investigations** | | | | |
| Total WBC (x10^9^/L), mean (SD) | 17.62 (10.09) | 23.73 (9.96) | 16.32 (9.70) | 0.020 |
| PLT (x10^9^/L), mean (SD) | 185.96 (147.33) | 209.39 (133.08) | 180.97 (150.74) | 0.310 |
| Hb (g/dL), mean (SD) | 11.37 (2.75) | 12.28 (3.20) | 11.18 (2.63) | 0.098 |
| Hct (%), mean (SD) | 35.04 (7.98) | 36.82 (10.03) | 34.67 (7.52) | 0.230 |
| K^+^ (mmol/L), mean (SD) | 3.91 (0.96) | 3.85 (0.69) | 3.93 (0.90) | 0.938 |
| Na^+^ (mmol/L), mean (SD) | 136.5 (7.88) | 133.62 (6.23) | 137.12 (8.10) | 0.092 |
| Creatinine (µmol/L), mean (SD) | 210.25 (145.01) | 186.73 (128.54) | 215.26 (148.77) | 0.435 |
| Bilirubin (µmol/l), mean (SD) | 31.64  (47.21) | 41.31 (61.99) | 29.68 (44.02) | 0.286 |
| pH, mean (SD) | 7.28 (0.14) | 7.35 (0.11) | 7.27 (0.14) | 0.033 |
| PaO_2_ (mmHg), mean (SD) | 118.67 (83.64) | 81.64 (26.17) | 125.34 (88.70) | 0.069 |
| FiO_2_, mean (SD) | 0.56 (0.25) | 0.38 (0.12) | 0.60 (0.25) | 0.002 |
| PaO_2_/FiO_2_ ratio, mean (SD) | 236.23 (144.23) | 227.28 (85.79) | 237.85 (152.88) | 0.869 |
| **Severity of illness scores** | | | | |
| SOFA, median (IQR) | 10 (7-12.25) | 7 (5.5-8) | 10 (8.5-13) | <0.001 |
| APACHE II, median (IQR) | 23 (16.75-28) | 14 (11.5-19.5) | 24 (20.5-30) | <0.001 |
| **Life-sustaining treatments during ICU stay** | | | | |
| Additional ICU support, no. (%) |  |  |  |  |
| Vasopressors/inotropes | 74 (100) | 13 (100) | 61 (100) | - |
| Renal replacement therapy | 43 (58.1) | 3 (23.1) | 40 (65.6) | 0.005 |
| Red blood cell transfusion | 31 (41.9) | 1 (7.7) | 30 (49.2) | 0.006 |
| Platelet transfusion | 18 (24.3) | 0 | 18 (29.5) | 0.030 |
| Fresh frozen plasma transfusion | 24 (32.4) | 1 (7.7) | 23 (37.7) | 0.049 |
| Surgical source control | 8 (10.8) | 2 (15.4) | 6 (9.8) | 0.624 |
| Non-surgical source control | 13 (17.6) | 1 (7.7) | 12 (19.7) | 0.442 |
| **Outcome** | | | | |
| Length of stay, median days (IQR) |  |  |  |  |
| ICU | 8 (5-15.25) | 6 (5.5-7) | 12 (4.5-17.5) | 0.051 |
| Hospital | 14 (8-20) | 12 (9-17.5) | 15 (8-22.5) | 0.330 |
| Mortality, no. (%) |  |  |  |  |
| ICU | 31 (41.9) | 0 | 31 (50.8) | 0.001 |
| Hospital | 39 (52.7) | 1 (7.7) | 38 (62.3) | <.001 |
| ^a^ Comparison between no renal replacement therapy and renal replacement therapy.  Abbreviations: **APACHE II**, acute physiologic assessment and chronic health evaluation II; **FiO_2_**, fraction of inspired oxygen; **GCS**, Glasgow coma scale; **Hb**, hemoglobin; **Hct**, hematocrit; **HDU**, high dependency unit; **ICU**, intensive care unit; **IQR**, interquartile range; **MBP**, mean blood pressure; **no.**, number; **PaO_2_**, partial pressure of oxygen; **PLT**, platelet; **qSOFA**, quick sequential organ failure assessment; **RR**, respiratory rate; **SBP**, systolic blood pressure; **SD**, standard deviation; **SIRS**, systemic inflammatory response syndrome; **SOFA**, sequential organ failure assessment; **WBC**, white blood cell. | | | | |

**Table S29**. Clinical and laboratory characteristics and severity of illness according to methods of renal replacement therapy for patients with septic shock

| Variable | All casesn=74 | No renal replacement therapy  n=31 | Renal replacement therapy  n=43 | P^a^ |
| --- | --- | --- | --- | --- |
| **Vital signs** (on admission into ICU) | | | | |
| GCS, median (IQR) | 11 (7.5-14.5) | 12 (8.75-15) | 10 (6-14) | 0.079 |
| HR (beats per min), median (IQR) | 120 (100-130.5) | 110 (90-130) | 120 (110-132) | 0.122 |
| Temperature (^o^C), mean (SD) | 37.74 (1.01) | 37.83 (1.09) | 37.67 (0.96) | 0.656 |
| MBP (mmHg), mean(SD) | 53.45 (10.08) | 54.42 (5.97) | 52.74 (12.24) | 0.724 |
| SBP (mmHg), mean (SD) | 78.15 (18.22) | 80.19 (14.96) | 76.67 (20.30) | 0.802 |
| RR (breaths per min), median (IQR) | 25 (21-30) | 24 (20-30) | 26 (22-30) | 0.339 |
| **Blood investigations** | | | | |
| Total WBC (x10^9^/L), mean (SD) | 17.62 (10.09) | 18.13 (10.83) | 17.26 (9.62) | 0.874 |
| PLT (x10^9^/L), mean (SD) | 185.96 (147.33) | 198.65 (154.02) | 176.81 (143.45) | 0.443 |
| Hb (g/dL), mean (SD) | 11.37 (2.75) | 11.23 (2.62) | 11.47 (2.86) | 0.726 |
| Hct (%), mean (SD) | 35.04 (7.98) | 34.18 (7.50) | 35.67 (8.33) | 0.543 |
| K^+^ (mmol/L), mean (SD) | 3.91 (0.96) | 3.81 (0.74) | 3.99 (0.94) | 0.649 |
| Na^+^ (mmol/L), mean (SD) | 136.5 (7.88) | 136.48 (6.72) | 136.51 (8.70) | 0.952 |
| Creatinine (µmol/L), mean (SD) | 210.25 (145.01) | 178.94 (121.27) | 232.82 (157.47) | 0.048 |
| Bilirubin (µmol/l), mean (SD) | 31.64  (47.21) | 23.95 (41.55) | 36.96 (50.55) | 0.124 |
| pH, mean (SD) | 7.28 (0.14) | 7.33 (0.12) | 7.25 (0.14) | 0.006 |
| PaO_2_ (mmHg), mean (SD) | 118.67 (83.64) | 105.49 (74.67) | 128.08 (89.18) | 0.222 |
| FiO_2_, mean (SD) | 0.56 (0.25) | 0.49 (0.21) | 0.61 (0.27) | 0.061 |
| PaO_2_/FiO_2_ ratio, mean (SD) | 236.23 (144.23) | 221.88 (93.54) | 246.49 (171.98) | 0.819 |
| **Severity of illness scores** | | | | |
| SOFA, median (IQR) | 10 (7-12.25) | 7 (7-10) | 11 (9-14) | 0.001 |
| APACHE II, median (IQR) | 23 (16.75-28) | 20 (14-26) | 24 (21-29) | 0.022 |
| **Life-sustaining treatments during ICU stay** | | | | |
| Respiratory support, no. (%) and median (IQR), days |  |  |  |  |
| Mechanical ventilation | 61 (82.4) | 21 (67.7) | 40 (93.0) | 0.005 |
| Duration of mechanical ventilation | 7 (3-13) | 6 (3-10.5) | 7 (2.75-16) | 0.456 |
| Non-invasive ventilation | 4 (5.4) | 0 | 4 (9.3) | 0.135 |
| Duration of non-invasive ventilation | 2 (1.25-5.75) | - | 2 (1.25-5.75) | - |
| High-flow nasal oxygen | 11 (14.9) | 9 (29.0) | 2 (4.7) | 0.006 |
| Duration of high-flow nasal oxygen | 2 (1-3) | 2 (1-3) | 5 (4-5) | 0.029 |
| **Outcome** | | | | |
| Length of stay, median days (IQR) |  |  |  |  |
| ICU | 8 (5-15.25) | 7 (4-14) | 8 (6-17) | 0.436 |
| Hospital | 14 (8-20) | 17 (10-20) | 13 (6-22) | 0.408 |
| Mortality, no. (%) |  |  |  |  |
| ICU | 31 (41.9) | 4 (12.9) | 27 (62.8) | <0.001 |
| Hospital | 39 (52.7) | 9 (29.0) | 30 (69.8) | 0.001 |
| ^a^ Comparison between no renal replacement therapy and renal replacement therapy.  Abbreviations: **APACHE II**, acute physiologic assessment and chronic health evaluation II; **FiO_2_**, fraction of inspired oxygen; **GCS**, Glasgow coma scale; **Hb**, hemoglobin; **Hct**, hematocrit; **HDU**, high dependency unit; **ICU**, intensive care unit; **IQR**, interquartile range; **MBP**, mean blood pressure; **no.**, number; **PaO_2_**, partial pressure of oxygen; **PLT**, platelet; **qSOFA**, quick sequential organ failure assessment; **RR**, respiratory rate; **SBP**, systolic blood pressure; **SD**, standard deviation; **SIRS**, systemic inflammatory response syndrome; **SOFA**, sequential organ failure assessment; **WBC**, white blood cell. | | | | |

**Table S30.** Factors associated with hospital mortality in patients with septic shock: bivariate regression analyses

| Factor | Frequency | OR | 95.0% CI for OR | | P |
| --- | --- | --- | --- | --- | --- |
|  |  |  | Lower | Upper |  |
| Participating hospital |  |  |  |  |  |
| 115 People's | 12 | - | - | - | 0.886 |
| Bach Mai | 5 | 0.133 | 0.013 | 1.393 | 0.092 |
| Bai Chay | 1 | 0.000 | 0.000 | - | >0.999 |
| Can Tho | 4 | 323094972.9 | 0.000 | - | 0.999 |
| Cho Ray | 18 | 0.200 | 0.034 | 1.183 | 0.076 |
| Da Nang | 6 | 0.200 | 0.022 | 1.816 | 0.153 |
| Dong Da | 1 | 0.000 | 0.000 | - | >0.999 |
| Hanoi Medical University | 2 | 0.000 | 0.000 | - | 0.999 |
| Hue | 8 | 0.200 | 0.026 | 1.562 | 0.125 |
| Thai Nguyen | 2 | 0.200 | 0.008 | 4.716 | 0.318 |
| Vietnam–Czechoslovakia Friendship | 15 | 0.133 | 0.021 | 0.836 | 0.032 |
| **Hospital characteristics** |  |  |  |  |  |
| University affiliation |  |  |  |  |  |
| No | 39 | - | - | - | - |
| Yes | 35 | 0.908 | 0.364 | 2.264 | 0.835 |
| **ICU characteristics** |  |  |  |  |  |
| Type of ICU |  |  |  |  |  |
| Medical | 38 | - | - | - |  |
| Mixed | 36 | 0.522 | 0.207 | 1.316 | 0.168 |
| Nurse to patient ratio |  |  |  |  |  |
| 1 nurse : 2 patients | 56 | - | - | - | - |
| 1 nurse : 4 or more patients | 18 | 0.636 | 0.216 | 1.879 | 0.413 |
| Intensivist to patient ratio |  |  |  |  |  |
| 1 intensivist : 5 or fewer patients | 55 | - | - | - | 0.854 |
| 1 intensivist : 6 to 8 patients | 13 | 0.714 | 0.212 | 2.402 | 0.587 |
| 1 intensivist : 12 or more patients | 6 | 0.833 | 0.154 | 4.498 | 0.832 |
| Training programme in ICU |  |  |  |  |  |
| No | 17 | - | - | - | - |
| Yes | 57 | 0.167 | 0.043 | 0.647 | 0.010 |
| **Baseline characteristics** |  |  |  |  |  |
| Age (year) | 74 | 0.996 | 0.971 | 1.021 | 0.742 |
| Age (year) group |  |  |  |  |  |
| < 20 | 1 | - | - | - | 0.841 |
| 20 - 39 | 7 | 0.000 | 0.000 | - | >0.999 |
| 40 - 59 | 22 | 0.000 | 0.000 | - | 0.999 |
| ≥ 60 | 44 | 0.000 | 0.000 | - | 0.999 |
| Sex (male) | 41 | 1.358 | 0.541 | 3.407 | 0.515 |
| Collection batch |  |  |  |  |  |
| Collection 1 (Jan) | 24 | - | - | - | 0.014 |
| Collection 2 (April) | 22 | 8.000 | 2.144 | 29.852 | 0.002 |
| Collection 3 (July) | 16 | 5.000 | 1.270 | 19.685 | 0.021 |
| Collection 4 (Oct) | 12 | 4.200 | 0.962 | 18.328 | 0.056 |
| Admission type |  |  |  |  |  |
| Medical | 69 | - | - | - | - |
| Unscheduled surgical | 5 | 0.204 | 0.022 | 1.920 | 0.165 |
| Admission source |  |  |  |  |  |
| Emergency department | 41 | - | - | - | 0.979 |
| Operating room | 2 | 0.783 | 0.046 | 13.390 | 0.866 |
| General wards | 12 | 0.783 | 0.216 | 2.840 | 0.709 |
| Other ICUs or HDU | 4 | 0.783 | 0.100 | 6.108 | 0.815 |
| Inter-hospital transfer | 14 | 0.587 | 0.172 | 1.998 | 0.394 |
| Others | 1 | 1264284677 | 0.000 | - | >0.999 |
| Comorbidities |  |  |  |  |  |
| Cardiovascular disease | 20 | 2.000 | 0.691 | 5.788 | 0.201 |
| Chronic lung disease | 5 | 0.577 | 0.091 | 3.669 | 0.560 |
| Chronic neurological disease | 5 | 0.000 | 0.000 | - | 0.999 |
| Chronic kidney disease | 7 | 1.219 | 0.253 | 5.870 | 0.805 |
| Peptic ulcer disease | 3 | 1.836 | 0.159 | 21.195 | 0.626 |
| Chronic liver disease | 7 | 6.182 | 0.705 | 54.169 | 0.100 |
| Diabetes mellitus | 19 | 0.996 | 0.350 | 2.831 | 0.994 |
| Immunosuppression | 5 | 0.577 | 0.091 | 3.669 | 0.560 |
| Haematological malignancies | 2 | 0.895 | 0.054 | 14.864 | 0.938 |
| Solid malignant tumours | 2 | 0.895 | 0.054 | 14.864 | 0.938 |
| **Vital signs** |  |  |  |  |  |
| GCS | 73 | 0.846 | 0.735 | 0.974 | 0.020 |
| HR (beats per min) | 74 | 1.029 | 0.1004 | 1.054 | 0.021 |
| Temperature (^o^C) | 74 | 1.710 | 1.039 | 2.814 | 0.035 |
| MBP (mmHg) | 74 | 0.967 | 0.917 | 1.020 | 0.214 |
| SBP (mmHg) | 74 | 1.007 | 0.939 | 1.080 | 0.846 |
| RR (breaths per min) | 74 | 0.960 | 0.915 | 1.007 | 0.093 |
| **Blood investigations** |  |  |  |  |  |
| Total WBC (x10^9^/L) | 74 | 0.996 | 0.993 | 1.000 | 0.041 |
| PLT (x10^9^/L) | 74 | 0.966 | 0.817 | 1.143 | 0.690 |
| Hb (g/dL) | 74 | 0.992 | 0.937 | 1.051 | 0.794 |
| Hct (%) | 74 | 0.830 | 0.485 | 1.421 | 0.497 |
| K^+^ (mmol/L) | 74 | 0.962 | 0.905 | 1.022 | 0.212 |
| Na^+^ (mmol/L) | 74 | 1.000 | 0.997 | 1.004 | 0.769 |
| Creatinine (µmol/L) | 71 | 1.008 | 0.996 | 1.021 | 0.198 |
| Bilirubin (µmol/l) | 73 | 0.013 | 0.000 | 0.637 | 0.029 |
| pH | 72 | 1.003 | 0.997 | 1.009 | 0.320 |
| PaO_2_ (mmHg) | 73 | 22.537 | 2.482 | 204.627 | 0.006 |
| FiO_2_ | 72 | 1.000 | 0.996 | 1.003 | 0.791 |
| PaO_2_/FiO_2_ ratio | 74 | 2.332 | 1.055 | 5.157 | 0.037 |
| **Severity of illness scores** |  |  |  |  |  |
| qSOFA | 74 | 2.332 | 1.055 | 5.157 | 0.037 |
| qSOFA |  |  |  |  |  |
| 0 - 1 | 5 | - | - | - | - |
| 2 - 3 | 69 | 4.903 | 0.521 | 46.153 | 0.165 |
| SIRS | 74 | 1.526 | 0.912 | 2.554 | 0.108 |
| SOFA | 74 | 1.181 | 1.028 | 1.357 | 0.019 |
| SOFA |  |  |  |  |  |
| 2 - 3 | 5 | - | - | - | 0.077 |
| 4 - 5 | 4 | 0.000 | 0.000 | 0 | 0.999 |
| 6 - 7 | 14 | 0.100 | 0.008 | 1.193 | 0.069 |
| 8 - 9 | 12 | 0.125 | 0.010 | 1.520 | 0.103 |
| 10 - 11 | 16 | 0.321 | 0.029 | 3.556 | 0.355 |
| 12 - 14 | 15 | 0.688 | 0.058 | 8.145 | 0.766 |
| > 14 | 8 | 1.750 | 0.084 | 36.287 | 0.718 |
| SOFA |  |  |  |  |  |
| 0 - 6 | 11 | - | - | - | 0.033 |
| 7 - 9 | 24 | 0.494 | 0.113 | 2.165 | 0.350 |
| 10 - 12 | 16 | 1.543 | 0.329 | 7.226 | 0.582 |
| 13 - 14 | 15 | 3.300 | 0.635 | 17.160 | 0.156 |
| > 14 | 8 | 8.400 | 0.756 | 93.344 | 0.083 |
| SOFA |  |  |  |  |  |
| 0 - 3 | 5 | - | - | - | 0.007 |
| 4 - 7 | 18 | 0.071 | 0.006 | 0.834 | 0.035 |
| 8 - 9 | 12 | 0.125 | 0.010 | 1.520 | 0.103 |
| 10 - 11 | 16 | 0.321 | 0.029 | 3.556 | 0.355 |
| ≥ 12 | 23 | 0.900 | 0.081 | 9.970 | 0.932 |
| APACHE II | 74 | 1.107 | 1.034 | 1.185 | 0.003 |
| APACHE II |  |  |  |  |  |
| 5 - 9 | 2 | - | - | - | 0.181 |
| 10 - 14 | 12 | 538492145.6 | 0.000 | 0 | 0.999 |
| 15 - 19 | 9 | 807738218.4 | 0.000 | 0 | 0.999 |
| 20 - 24 | 22 | 1938571724 | 0.000 | 0 | 0.999 |
| 25 - 29 | 13 | 3634821983 | 0.000 | 0 | 0.999 |
| 30 - 34 | 9 | 5654167529 | 0.000 | 0 | 0.999 |
| > 34 | 7 | 4038691092 | 0.000 | 0 | 0.999 |
| APACHE II |  |  |  |  |  |
| 0 - 9 | 2 | - | - | - | 0.116 |
| 10 - 14 | 12 | 538493066.6 | 0.000 | - | 0.999 |
| 15 - 19 | 9 | 807739600.0 | 0.000 | - | 0.999 |
| 20 - 24 | 22 | 1938575040 | 0.000 | - | 0.999 |
| 25 - 29 | 13 | 3634828200 | 0.000 | - | 0.999 |
| ≥ 30 | 16 | 4846437600 | 0.000 | - | 0.999 |
| **Site of Infection** |  |  |  |  |  |
| Respiratory | 38 | 1.537 | 0.614 | 3.849 | 0.359 |
| Urinary tract | 10 | 0.333 | 0.079 | 1.407 | 0.135 |
| Abdominal | 26 | 1.073 | 0.412 | 2.794 | 0.885 |
| Neurological | 2 | 0.895 | 0.054 | 14.864 | 0.938 |
| Bones or joints | 1 | 0.000 | 0.000 | - | >0.999 |
| Skin or cutaneous sites | 6 | 1.886 | 0.324 | 10.991 | 0.481 |
| Primary bacteraemia | 2 | 0.895 | 0.054 | 14.864 | 0.938 |
| Systemic | 3 | 1.838 | 0.159 | 21.195 | 0.626 |
| **Microbiology** |  |  |  |  |  |
| Pathogens detection |  |  |  |  |  |
| No pathogens detected | 23 | 0.344 | 0.123 | 0.960 | 0.042 |
| Gram negative bacteria | 43 | 1.687 | 0.665 | 4.280 | 0.271 |
| Gram positive bacteria | 6 | 1.886 | 0.324 | 10.991 | 0.481 |
| Viruses | 2 | 1528151899 | 0.000 | - | 0.999 |
| Other pathogens | 2 | 0.895 | 0.054 | 14.864 | 0.938 |
| **Completion of sepsis bundle elements** |  |  |  |  |  |
| Timing of antibiotics administration |  |  |  |  |  |
| 0-60 minutes | 51 | - | - | - | 0.930 |
| 61-120 minutes | 10 | 0.821 | 0.212 | 3.190 | 0.776 |
| 121-180 minutes | 2 | 0.821 | 0.049 | 13.866 | 0.891 |
| >180 minutes | 5 | 0.548 | 0.084 | 3.561 | 0.528 |
| Timing of obtaining blood cultures |  |  |  |  |  |
| 0-60 minutes | 29 | - | - | - | 0.691 |
| 61-120 minutes | 9 | 0.658 | 0.144 | 3.013 | 0.590 |
| 121-180 minutes | 1 | 850249928.6 | 0.000 | - | >0.009 |
| >180 minutes | 15 | 0.461 | 0.129 | 1.641 | 0.232 |
| Timing of obtaining lactate measurement |  |  |  |  |  |
| 0-60 minutes | 38 | - | - | - | 0.984 |
| 61-120 minutes | 5 | 1.350 | 0.202 | 9.018 | 0.757 |
| 121-180 minutes | 1 | 0.000 | 0.000 | - | >0.009 |
| >180 minutes | 18 | 0.900 | 0.293 | 2.764 | 0.854 |
| Completion of the sepsis bundle within 1 hour | 20 | 1.620 | 0.569 | 4.616 | 0.366 |
| Completion of the initial administration of antibiotics within 1 hour | 51 | 1.623 | 0.582 | 4.525 | 0.354 |
| Permutations of the completed elements within 1 hour |  |  |  |  |  |
| No elements completed | 9 | - | - | - | 0.831 |
| Antibiotics only | 17 | 1.778 | 0.331 | 9.554 | 0.502 |
| Blood cultures only | 1 | 3230949732 | 0.000 | - | >0.009 |
| Lactate only | 10 | 1.333 | 0.204 | 8.708 | 0.764 |
| Antibiotics + Lactate | 7 | 1.500 | 0.195 | 11.536 | 0.697 |
| Antibiotics + Blood cultures | 7 | 5.000 | 0.584 | 42.787 | 0.142 |
| Blood cultures + Lactate | 1 | 3230949732 | 0.000 | - | >0.009 |
| Full bundle | 20 | 3.000 | 0.576 | 15.614 | 0.192 |
| Vasopressors | 48 | 3.053 | 0.978 | 9.526 | 0.055 |
| Volume of fluids administered (mL) | 73 | 1.000 | 0.999 | 1.001 | 0.912 |
| Completion of the sepsis bundle within 3 hours | 27 | 1.662 | 0.633 | 4.366 | 0.302 |
| Completion of the initial administration of antibiotics within 3 hours | 63 | 2.345 | 0.538 | 10.217 | 0.256 |
| Permutation of the completed elements of 3-hour sepsis bundle |  |  |  |  |  |
| No elements completed | 2 | - | - | - | 0.301 |
| Antibiotics only | 15 | 0.500 | 0.026 | 9.770 | 0.648 |
| Blood cultures only | 1 | 0.000 | 0.000 | - | >0.009 |
| Lactate only | 6 | 0.500 | 0.019 | 12.898 | 0.676 |
| Antibiotics + Lactate | 10 | 0.667 | 0.032 | 14.033 | 0.794 |
| Antibiotics + Blood cultures | 11 | 4.500 | 0.190 | 106.823 | 0.352 |
| Full bundle | 27 | 1.455 | 0.082 | 25.812 | 0.798 |
| Vasopressors | 60 | 2.444 | 0.416 | 14.379 | 0.323 |
| Volume of fluids administered (mL) | 73 | 1.000 | 1.000 | 1.001 | 0.212 |
| **Life-sustaining treatments during ICU stay** |  |  |  |  |  |
| Respiratory support |  |  |  |  |  |
| Mechanical ventilation | 61 | 19.826 | 2.416 | 162.663 | 0.005 |
| Duration of mechanical ventilation | 59 | 0.974 | 0.921 | 1.030 | 0.355 |
| Non-invasive ventilation | 4 | 0.281 | 0.028 | 2.832 | 0.281 |
| High-flow nasal oxygen | 11 | 0.281 | 0.068 | 1.161 | 0.079 |
| Duration of high-flow nasal oxygen | 11 | 0.637 | 0.193 | 2.098 | 0.458 |
| Additional ICU support |  |  |  |  |  |
| Renal replacement therapy | 43 | 5.641 | 2.050 | 15.525 | 0.001 |
| Red blood cell transfusion | 31 | 2.297 | 0.888 | 5.942 | 0.086 |
| Platelet transfusion | 18 | 3.000 | 0.943 | 9.545 | 0.063 |
| Fresh frozen plasma transfusion | 24 | 4.143 | 1.405 | 12.214 | 0.010 |
| Surgical source control | 8 | 0.261 | 0.049 | 1.391 | 0.116 |
| Non-surgical source control | 13 | 0.727 | 0.219 | 2.417 | 0.603 |

**Table S31.** Factors associated with hospital mortality in patients with septic shock: multivariate logistic regression analyses (backward elimination)

| Step | Factor | Unit | OR | 95.0% CI for OR | | P |
| --- | --- | --- | --- | --- | --- | --- |
|  |  |  |  | Lower | Upper |  |
| 1 | **ICU characteristics** |  |  |  |  |  |
|  | Type of ICU |  |  |  |  |  |
|  | Medical | % | - | - | - | - |
|  | Mixed | % | 2.306 | 0.255 | 20.842 | 0.457 |
|  | Training programme in ICU |  |  |  |  |  |
|  | No | % | - | - | - | - |
|  | Yes | % | 0.145 | 0.011 | 1.917 | 0.143 |
|  | **Baseline characteristics** |  |  |  |  |  |
|  | Admission type |  |  |  |  |  |
|  | Medical | % | - | - | - | - |
|  | Unscheduled surgical | % | 0.706 | 0.019 | 26.854 | 0.851 |
|  | Comorbidities |  |  |  |  |  |
|  | Cardiovascular disease | % | 4.338 | 0.507 | 37.122 | 0.180 |
|  | Chronic liver disease | % | 16.865 | 0.277 | 1028.611 | 0.178 |
|  | **Severity of illness scores** |  |  |  |  |  |
|  | SOFA |  |  |  |  |  |
|  | 0 - 3 | % | - | - | - | 0.725 |
|  | 4-7 | % | 0.151 | 0.002 | 9.224 | 0.368 |
|  | 8-9 | % | 0.108 | 0.002 | 6.782 | 0.292 |
|  | 10 - 11 | % | 0.257 | 0.004 | 14.684 | 0.510 |
|  | ≥12 | % | 0.397 | 0.006 | 27.080 | 0.668 |
|  | **Microbiology** |  |  |  |  |  |
|  | No pathogens detected | % | 0.183 | 0.011 | 3.019 | 0.235 |
|  | Gram negative bacteria | % | 0.299 | 0.022 | 4.087 | 0.366 |
|  | **Completion of the sepsis bundle** |  |  |  |  |  |
|  | Completion of the sepsis bundle within 1 hour | % | 0.779 | 0.013 | 45.887 | 0.905 |
|  | Completion of the administration of antibiotics within 1 hour | % | 0.269 | 0.017 | 4.227 | 0.350 |
|  | Completion of the sepsis bundle within 3 hours | % | 1.627 | 0.046 | 57.661 | 0.789 |
|  | Completion of the administration of antibiotics within 3 hours | % | 19.557 | 0.409 | 935.469 | 0.132 |
|  | **Life-sustaining treatments during ICU stay** |  |  |  |  |  |
|  | Respiratory support |  |  |  |  |  |
|  | Mechanical ventilation | % | 10.891 | 0.452 | 262.687 | 0.141 |
|  | High-flow nasal oxygen | % | 1.781 | 0.127 | 24.987 | 0.668 |
|  | Additional ICU support |  |  |  |  |  |
|  | Renal replacement therapy | % | 3.502 | 0.632 | 19.413 | 0.151 |
|  | Red blood cell transfusion | % | 2.005 | 0.284 | 14.147 | 0.485 |
|  | Platelet transfusion | % | 0.880 | 0.110 | 7.032 | 0.904 |
|  | Fresh frozen plasma transfusion | % | 2.231 | 0.323 | 15.407 | 0.416 |
|  | Constant |  | 0.159 |  |  | 0.487 |
| 2 | **ICU characteristics** |  |  |  |  |  |
|  | Type of ICU |  |  |  |  |  |
|  | Medical | % | - | - | - | - |
|  | Mixed | % | 2.305 | .254 | 20.911 | 0.458 |
|  | Training programme in ICU |  |  |  |  |  |
|  | No | % | - | - | - | - |
|  | Yes | % | 0.144 | 0.011 | 1.894 | 0.140 |
|  | **Baseline characteristics** |  |  |  |  |  |
|  | Admission type |  |  |  |  |  |
|  | Medical | % | - | - | - | - |
|  | Unscheduled surgical | % | 0.686 | 0.018 | 25.658 | 0.838 |
|  | Comorbidities |  |  |  |  |  |
|  | Cardiovascular disease | % | 4.409 | 0.520 | 37.403 | 0.174 |
|  | Chronic liver disease | % | 16.280 | 0.276 | 961.495 | 0.180 |
|  | **Severity of illness scores** |  |  |  |  |  |
|  | SOFA |  |  |  |  |  |
|  | 0 - 3 | % | - | - | - | 0.704 |
|  | 4-7 | % | 0.155 | 0.003 | 9.171 | 0.370 |
|  | 8-9 | % | 0.105 | 0.002 | 6.525 | 0.285 |
|  | 10 - 11 | % | 0.253 | 0.004 | 14.563 | 0.507 |
|  | ≥12 | % | 0.406 | 0.006 | 27.534 | 0.675 |
|  | **Microbiology** |  |  |  |  |  |
|  | No pathogens detected | % | 0.177 | 0.011 | 2.747 | 0.216 |
|  | Gram negative bacteria | % | 0.293 | .022 | 3.883 | 0.352 |
|  | **Completion of the sepsis bundle** |  |  |  |  |  |
|  | Completion of the administration of antibiotics within 1 hour | % | .246 | 0.024 | 2.484 | 0.235 |
|  | Completion of the sepsis bundle within 3 hours | % | 1.346 | 0.237 | 7.652 | 0.738 |
|  | Completion of the administration of antibiotics within 3 hours | % | 21.432 | 0.606 | 758.445 | 0.092 |
|  | **Life-sustaining treatments during ICU stay** |  |  |  |  |  |
|  | Respiratory support |  |  |  |  |  |
|  | Mechanical ventilation | % | 11.217 | 0.477 | 263.929 | 0.134 |
|  | High-flow nasal oxygen | % | 1.731 | 0.129 | 23.255 | 0.679 |
|  | Additional ICU support |  |  |  |  |  |
|  | Renal replacement therapy | % | 3.464 | 0.633 | 18.954 | 0.152 |
|  | Red blood cell transfusion | % | 1.991 | 0.282 | 14.054 | 0.490 |
|  | Platelet transfusion | % | 0.862 | 0.110 | 6.775 | 0.888 |
|  | Fresh frozen plasma transfusion | % | 2.255 | 0.327 | 15.539 | 0.409 |
|  | Constant |  | 0.158 |  |  | 0.486 |
| 3 | **ICU characteristics** |  |  |  |  |  |
|  | Type of ICU |  |  |  |  |  |
|  | Medical | % | - | - | - | - |
|  | Mixed | % | 2.202 | 0.270 | 17.968 | 0.461 |
|  | Training programme in ICU |  |  |  |  |  |
|  | No | % | - | - | - | - |
|  | Yes | % | 0.148 | 0.012 | 1.864 | 0.140 |
|  | **Baseline characteristics** |  |  |  |  |  |
|  | Admission type |  |  |  |  |  |
|  | Medical | % | - | - | - | - |
|  | Unscheduled surgical | % | 0.637 | 0.019 | 20.833 | 0.800 |
|  | Comorbidities |  |  |  |  |  |
|  | Cardiovascular disease | % | 4.257 | 0.541 | 33.485 | 0.169 |
|  | Chronic liver disease | % | 15.928 | 0.285 | 889.049 | 0.177 |
|  | **Severity of illness scores** |  |  |  |  |  |
|  | SOFA |  |  |  |  |  |
|  | 0 - 3 | % | - | - | - | 0.703 |
|  | 4-7 | % | 0.154 | 0.003 | 9.237 | 0.370 |
|  | 8-9 | % | 0.106 | 0.002 | 6.661 | 0.288 |
|  | 10 - 11 | % | 0.253 | 0.004 | 14.805 | 0.508 |
|  | ≥12 | % | 0.388 | 0.006 | 25.522 | 0.658 |
|  | **Microbiology** |  |  |  |  |  |
|  | No pathogens detected | % | 0.176 | 0.011 | 2.736 | 0.214 |
|  | Gram negative bacteria | % | 0.299 | 0.023 | 3.924 | 0.358 |
|  | **Completion of the sepsis bundle** |  |  |  |  |  |
|  | Completion of the administration of antibiotics within 1 hour | % | 0.241 | 0.024 | 2.383 | 0.224 |
|  | Completion of the sepsis bundle within 3 hours | % | 1.333 | 0.236 | 7.537 | 0.745 |
|  | Completion of the administration of antibiotics within 3 hours | % | 21.036 | 0.593 | 745.807 | 0.094 |
|  | **Life-sustaining treatments during ICU stay** |  |  |  |  |  |
|  | Respiratory support |  |  |  |  |  |
|  | Mechanical ventilation | % | 11.275 | 0.478 | 265.899 | 0.133 |
|  | High-flow nasal oxygen | % | 1.773 | 0.135 | 23.303 | 0.663 |
|  | Additional ICU support |  |  |  |  |  |
|  | Renal replacement therapy | % | 3.456 | 0.630 | 18.947 | 0.153 |
|  | Red blood cell transfusion | % | 1.912 | 0.295 | 12.373 | 0.496 |
|  | Fresh frozen plasma transfusion | % | 2.158 | 0.345 | 13.479 | 0.411 |
|  | Constant |  | 0.164 |  |  | 0.495 |
| 4 | **ICU characteristics** |  |  |  |  |  |
|  | Type of ICU |  |  |  |  |  |
|  | Medical | % | - | - | - | - |
|  | Mixed | % | 1.967 | 0.296 | 13.067 | 0.484 |
|  | Training programme in ICU |  |  |  |  |  |
|  | No | % | - | - | - | - |
|  | Yes | % | 0.157 | 0.013 | 1.867 | 0.143 |
|  | **Baseline characteristics** |  |  |  |  |  |
|  | Comorbidities |  |  |  |  |  |
|  | Cardiovascular disease | % | 4.127 | 0.536 | 31.784 | 0.174 |
|  | Chronic liver disease | % | 15.007 | 0.282 | 798.770 | 0.182 |
|  | **Severity of illness scores** |  |  |  |  |  |
|  | SOFA |  |  |  |  |  |
|  | 0 - 3 | % | - | - | - | 0.654 |
|  | 4-7 | % | 0.142 | 0.002 | 8.204 | 0.346 |
|  | 8-9 | % | 0.108 | 0.002 | 6.691 | 0.290 |
|  | 10 - 11 | % | 0.248 | 0.004 | 14.465 | 0.501 |
|  | ≥12 | % | 0.420 | 0.007 | 26.439 | 0.681 |
|  | **Microbiology** |  |  |  |  |  |
|  | No pathogens detected | % | 0.167 | 0.011 | 2.553 | 0.198 |
|  | Gram negative bacteria | % | 0.282 | 0.022 | 3.540 | 0.326 |
|  | **Completion of the sepsis bundle** |  |  |  |  |  |
|  | Completion of the administration of antibiotics within 1 hour | % | 0.255 | 0.027 | 2.379 | 0.231 |
|  | Completion of the sepsis bundle within 3 hours | % | 1.350 | 0.239 | 7.635 | 0.734 |
|  | Completion of the administration of antibiotics within 3 hours | % | 21.882 | 0.652 | 734.876 | 0.085 |
|  | **Life-sustaining treatments during ICU stay** |  |  |  |  |  |
|  | Respiratory support |  |  |  |  |  |
|  | Mechanical ventilation | % | 11.212 | 0.490 | 256.434 | 0.130 |
|  | High-flow nasal oxygen | % | 1.949 | 0.164 | 23.171 | 0.597 |
|  | Additional ICU support |  |  |  |  |  |
|  | Renal replacement therapy | % | 3.442 | 0.628 | 18.870 | 0.155 |
|  | Red blood cell transfusion | % | 1.731 | 0.318 | 9.424 | 0.525 |
|  | Fresh frozen plasma transfusion | % | 2.178 | 0.351 | 13.503 | 0.403 |
|  | Constant |  | 0.163 |  |  | 0.492 |
| 5 | **ICU characteristics** |  |  |  |  |  |
|  | Type of ICU |  |  |  |  |  |
|  | Medical | % | - | - | - | - |
|  | Mixed | % | 1.938 | 0.298 | 12.616 | 0.489 |
|  | Training programme in ICU |  |  |  |  |  |
|  | No | % | - | - | - | - |
|  | Yes | % | 0.167 | 0.015 | 1.863 | 0.146 |
|  | **Baseline characteristics** |  |  |  |  |  |
|  | Comorbidities |  |  |  |  |  |
|  | Cardiovascular disease | % | 4.341 | 0.585 | 32.202 | 0.151 |
|  | Chronic liver disease | % | 13.980 | 0.287 | 680.248 | 0.183 |
|  | **Severity of illness scores** |  |  |  |  |  |
|  | SOFA |  |  |  |  |  |
|  | 0 - 3 | % | - | - | - | 0.659 |
|  | 4-7 | % | 0.138 | 0.002 | 8.439 | 0.346 |
|  | 8-9 | % | 0.104 | 0.002 | 6.835 | 0.289 |
|  | 10 - 11 | % | 0.256 | 0.004 | 15.572 | 0.515 |
|  | ≥12 | % | 0.392 | 0.006 | 25.591 | 0.660 |
|  | **Microbiology** |  |  |  |  |  |
|  | No pathogens detected | % | 0.160 | 0.011 | 2.423 | 0.186 |
|  | Gram negative bacteria | % | 0.302 | 0.025 | 3.674 | 0.348 |
|  | **Completion of the sepsis bundle** |  |  |  |  |  |
|  | Completion of the administration of antibiotics within 1 hour | % | 0.240 | 0.027 | 2.144 | 0.201 |
|  | Completion of the administration of antibiotics within 3 hours | % | 25.842 | 0.918 | 727.402 | 0.056 |
|  | **Life-sustaining treatments during ICU stay** |  |  |  |  |  |
|  | Respiratory support |  |  |  |  |  |
|  | Mechanical ventilation | % | 11.165 | 0.488 | 255.222 | 0.131 |
|  | High-flow nasal oxygen | % | 2.080 | 0.179 | 24.211 | 0.559 |
|  | Additional ICU support |  |  |  |  |  |
|  | Renal replacement therapy | % | 3.576 | 0.657 | 19.459 | 0.140 |
|  | Red blood cell transfusion | % | 1.794 | 0.334 | 9.639 | 0.496 |
|  | Fresh frozen plasma transfusion | % | 2.200 | 0.357 | 13.581 | 0.396 |
|  | Constant |  | 0.147 |  |  | 0.469 |
| 6 | **ICU characteristics** |  |  |  |  |  |
|  | Type of ICU |  |  |  |  |  |
|  | Medical | % | - | - | - | - |
|  | Mixed | % | 2.547 | 0.488 | 13.287 | 0.267 |
|  | Training programme in ICU |  |  |  |  |  |
|  | No | % | - | - | - | - |
|  | Yes | % | 0.097 | 0.011 | .852 | 0.035 |
|  | **Baseline characteristics** |  |  |  |  |  |
|  | Comorbidities |  |  |  |  |  |
|  | Cardiovascular disease | % | 4.115 | 0.658 | 25.742 | 0.131 |
|  | Chronic liver disease | % | 21.393 | .544 | 840.557 | 0.102 |
|  | **Microbiology** |  |  |  |  |  |
|  | No pathogens detected | % | 0.156 | 0.013 | 1.929 | 0.148 |
|  | Gram negative bacteria | % | 0.315 | 0.029 | 3.438 | 0.343 |
|  | **Completion of the sepsis bundle** |  |  |  |  |  |
|  | Completion of the administration of antibiotics within 1 hour | % | .343 | 0.048 | 2.449 | 0.286 |
|  | Completion of the administration of antibiotics within 3 hours | % | 17.680 | 0.984 | 317.518 | 0.051 |
|  | **Life-sustaining treatments during ICU stay** |  |  |  |  |  |
|  | Respiratory support |  |  |  |  |  |
|  | Mechanical ventilation | % | 12.784 | 0.831 | 196.676 | 0.068 |
|  | High-flow nasal oxygen | % | 1.816 | 0.188 | 17.549 | 0.606 |
|  | Additional ICU support |  |  |  |  |  |
|  | Renal replacement therapy | % | 2.979 | 0.667 | 13.302 | 0.153 |
|  | Red blood cell transfusion | % | 2.042 | 0.430 | 9.699 | 0.369 |
|  | Fresh frozen plasma transfusion | % | 2.745 | 0.602 | 12.520 | 0.192 |
|  | Constant |  | 0.045 |  |  | 0.160 |
| 7 | **ICU characteristics** |  |  |  |  |  |
|  | Type of ICU |  |  |  |  |  |
|  | Medical | % | - | - | - | - |
|  | Mixed | % | 2.840 | 0.572 | 14.106 | 0.202 |
|  | Training programme in ICU |  |  |  |  |  |
|  | No | % | - | - | - | - |
|  | Yes | % | 0.094 | 00.011 | 0.834 | 0.034 |
|  | **Baseline characteristics** |  |  |  |  |  |
|  | Comorbidities |  |  |  |  |  |
|  | Cardiovascular disease | % | 4.521 | 0.735 | 27.787 | 0.103 |
|  | Chronic liver disease | % | 26.271 | 0.640 | 1078.064 | 0.085 |
|  | **Microbiology** |  |  |  |  |  |
|  | No pathogens detected | % | 0.163 | 0.014 | 1.910 | 0.149 |
|  | Gram negative bacteria | % | 0.339 | 0.033 | 3.473 | 0.362 |
|  | **Completion of the sepsis bundle** |  |  |  |  |  |
|  | Completion of the administration of antibiotics within 1 hour | % | 0.369 | 0.054 | 2.527 | 0.310 |
|  | Completion of the administration of antibiotics within 3 hours | % | 16.196 | 0.901 | 291.242 | 0.059 |
|  | **Life-sustaining treatments during ICU stay** |  |  |  |  |  |
|  | Respiratory support |  |  |  |  |  |
|  | Mechanical ventilation | % | 9.801 | 0.824 | 116.578 | 0.071 |
|  | Additional ICU support |  |  |  |  |  |
|  | Renal replacement therapy | % | 2.678 | 0.652 | 11.000 | 0.172 |
|  | Red blood cell transfusion | % | 2.120 | 0.449 | 10.011 | 0.343 |
|  | Fresh frozen plasma transfusion | % | 2.741 | 0.602 | 12.479 | 0.192 |
|  | Constant |  | 0.060 |  |  | 0.174 |
| 8 | **ICU characteristics** |  |  |  |  |  |
|  | Type of ICU |  |  |  |  |  |
|  | Medical | % | - | - | - | - |
|  | Mixed | % | 2.717 | 0.555 | 13.285 | 0.217 |
|  | Training programme in ICU |  |  |  |  |  |
|  | No | % | - | - | - | - |
|  | Yes | % | 0.095 | 0.011 | 0.813 | 0.032 |
|  | **Baseline characteristics** |  |  |  |  |  |
|  | Comorbidities |  |  |  |  |  |
|  | Cardiovascular disease | % | 4.457 | 0.732 | 27.124 | 0.105 |
|  | Chronic liver disease | % | 19.870 | 0.490 | 806.207 | 0.114 |
|  | **Microbiology** |  |  |  |  |  |
|  | No pathogens detected | % | 0.405 | 0.098 | 1.671 | 0.212 |
|  | **Completion of the sepsis bundle** |  |  |  |  |  |
|  | Completion of the administration of antibiotics within 1 hour | % | 0.316 | 0.046 | 2.148 | 0.239 |
|  | Completion of the administration of antibiotics within 3 hours | % | 14.482 | 0.912 | 229.942 | 0.058 |
|  | **Life-sustaining treatments during ICU stay** |  |  |  |  |  |
|  | Respiratory support |  |  |  |  |  |
|  | Mechanical ventilation | % | 9.616 | 0.849 | 108.973 | 0.068 |
|  | Additional ICU support |  |  |  |  |  |
|  | Renal replacement therapy | % | 3.015 | 0.749 | 12.144 | 0.120 |
|  | Red blood cell transfusion | % | 2.064 | 0.446 | 9.554 | 0.354 |
|  | Fresh frozen plasma transfusion | % | 2.805 | 0.627 | 12.552 | 0.177 |
|  | Constant |  | 0.030 |  |  | 0.057 |
| 9 | **ICU characteristics** |  |  |  |  |  |
|  | Type of ICU |  |  |  |  |  |
|  | Medical | % | - | - | - | - |
|  | Mixed | % | 2.759 | 0.575 | 13.229 | 0.205 |
|  | Training programme in ICU |  |  |  |  |  |
|  | No | % | - | - | - | - |
|  | Yes | % | 0.102 | 0.013 | 0.829 | 0.033 |
|  | **Baseline characteristics** |  |  |  |  |  |
|  | Comorbidities |  |  |  |  |  |
|  | Cardiovascular disease | % | 3.140 | 0.626 | 15.744 | 0.164 |
|  | Chronic liver disease | % | 14.722 | 0.347 | 624.376 | 0.160 |
|  | **Microbiology** |  |  |  |  |  |
|  | No pathogens detected | % | 0.355 | 0.089 | 1.422 | 0.143 |
|  | **Completion of the sepsis bundle** |  |  |  |  |  |
|  | Completion of the administration of antibiotics within 1 hour | % | 0.328 | 0.047 | 2.308 | 0.263 |
|  | Completion of the administration of antibiotics within 3 hours | % | 13.354 | 0.857 | 208.096 | 0.064 |
|  | **Life-sustaining treatments during ICU stay** |  |  |  |  |  |
|  | Respiratory support |  |  |  |  |  |
|  | Mechanical ventilation | % | 11.428 | 1.085 | 120.401 | 0.043 |
|  | Additional ICU support |  |  |  |  |  |
|  | Renal replacement therapy | % | 3.141 | 0.771 | 12.787 | 0.110 |
|  | Fresh frozen plasma transfusion | % | 3.068 | 0.714 | 13.189 | 0.132 |
|  | Constant |  | 0.037 |  |  | 0.064 |
| 10 | **ICU characteristics** |  |  |  |  |  |
|  | Type of ICU |  |  |  |  |  |
|  | Medical | % | - | - | - | - |
|  | Mixed | % | 2.258 | 0.507 | 10.060 | 0.285 |
|  | Training programme in ICU |  |  |  |  |  |
|  | No | % | - | - | - | - |
|  | Yes | % | 0.111 | 0.014 | 0.894 | 0.039 |
|  | **Baseline characteristics** |  |  |  |  |  |
|  | Comorbidities |  |  |  |  |  |
|  | Cardiovascular disease | % | 3.162 | 0.640 | 15.624 | 0.158 |
|  | Chronic liver disease | % | 18.711 | 0.350 | 999.357 | 0.149 |
|  | **Microbiology** |  |  |  |  |  |
|  | No pathogens detected | % | .420 | 0.112 | 1.578 | 0.199 |
|  | **Completion of the sepsis bundle** |  |  |  |  |  |
|  | Completion of the administration of antibiotics within 3 hours | % | 5.323 | 0.613 | 46.249 | 0.130 |
|  | **Life-sustaining treatments during ICU stay** |  |  |  |  |  |
|  | Respiratory support |  |  |  |  |  |
|  | Mechanical ventilation | % | 10.358 | 1.013 | 105.907 | 0.049 |
|  | Additional ICU support |  |  |  |  |  |
|  | Renal replacement therapy | % | 2.401 | 0.643 | 8.966 | 0.193 |
|  | Fresh frozen plasma transfusion | % | 3.440 | 0.811 | 14.600 | 0.094 |
|  | Constant |  | 0.045 |  |  | 0.074 |
| 11 | **ICU characteristics** |  |  |  |  |  |
|  | Training programme in ICU |  |  |  |  |  |
|  | No | % | - | - | - | - |
|  | Yes | % | 0.198 | .034 | 1.142 | 0.070 |
|  | **Baseline characteristics** |  |  |  |  |  |
|  | Comorbidities |  |  |  |  |  |
|  | Cardiovascular disease | % | 2.625 | 0.591 | 11.654 | 0.204 |
|  | Chronic liver disease | % | 10.523 | 0.278 | 398.480 | 0.204 |
|  | **Microbiology** |  |  |  |  |  |
|  | No pathogens detected | % | .427 | .117 | 1.561 | 0.198 |
|  | **Completion of the sepsis bundle** |  |  |  |  |  |
|  | Completion of the administration of antibiotics within 3 hours | % | 4.252 | 0.518 | 34.897 | 0.178 |
|  | **Life-sustaining treatments during ICU stay** |  |  |  |  |  |
|  | Respiratory support |  |  |  |  |  |
|  | Mechanical ventilation | % | 9.785 | 0.982 | 97.511 | 0.052 |
|  | Additional ICU support |  |  |  |  |  |
|  | Renal replacement therapy | % | 2.291 | 0.629 | 8.345 | 0.209 |
|  | Fresh frozen plasma transfusion | % | 3.219 | 0.786 | 13.182 | 0.104 |
|  | Constant |  | 0.061 |  |  | 0.092 |
| 12 | **ICU characteristics** |  |  |  |  |  |
|  | Training programme in ICU |  |  |  |  |  |
|  | No | % | - | - | - | - |
|  | Yes | % | 0.171 | 0.031 | 0.936 | 0.042 |
|  | **Baseline characteristics** |  |  |  |  |  |
|  | Comorbidities |  |  |  |  |  |
|  | Cardiovascular disease | % | 2.240 | 0.521 | 9.624 | 0.275 |
|  | Chronic liver disease | % | 11.737 | .389 | 354.584 | 0.157 |
|  | **Microbiology** |  |  |  |  |  |
|  | No pathogens detected | % | 0.386 | 0.107 | 1.390 | 0.145 |
|  | **Completion of the sepsis bundle** |  |  |  |  |  |
|  | Completion of the administration of antibiotics within 3 hours | % | 5.488 | 0.752 | 40.041 | 0.093 |
|  | **Life-sustaining treatments during ICU stay** |  |  |  |  |  |
|  | Respiratory support |  |  |  |  |  |
|  | Mechanical ventilation | % | 13.415 | 1.357 | 132.587 | 0.026 |
|  | Additional ICU support |  |  |  |  |  |
|  | Fresh frozen plasma transfusion | % | 3.689 | 0.943 | 14.433 | 0.061 |
|  | Constant |  | 0.069 |  |  | 0.100 |
| 13 | **ICU characteristics** |  |  |  |  |  |
|  | Training programme in ICU |  |  |  |  |  |
|  | No | % | - | - | - | - |
|  | Yes | % | 0.172 | 0.033 | 0.897 | 0.037 |
|  | **Baseline characteristics** |  |  |  |  |  |
|  | Comorbidities |  |  |  |  |  |
|  | Chronic liver disease | % | 10.841 | 0.397 | 296.224 | 0.158 |
|  | **Microbiology** |  |  |  |  |  |
|  | No pathogens detected | % | 0.380 | 0.107 | 1.350 | 0.135 |
|  | **Completion of the sepsis bundle** |  |  |  |  |  |
|  | Completion of the administration of antibiotics within 3 hours | % | 5.718 | 0.775 | 42.208 | 0.087 |
|  | **Life-sustaining treatments during ICU stay** |  |  |  |  |  |
|  | Respiratory support |  |  |  |  |  |
|  | Mechanical ventilation | % | 14.121 | 1.419 | 140.486 | 0.024 |
|  | Additional ICU support |  |  |  |  |  |
|  | Fresh frozen plasma transfusion | % | 3.353 | 0.890 | 12.634 | 0.074 |
|  | Constant |  | 0.078 |  |  | 0.117 |
| 14 | **ICU characteristics** |  |  |  |  |  |
|  | Training programme in ICU |  |  |  |  |  |
|  | No | % | - | - | - | - |
|  | Yes | % | 0.176 | 0.034 | 0.899 | 0.037 |
|  | **Microbiology** |  |  |  |  |  |
|  | No pathogens detected | % | .361 | 0.101 | 1.285 | 0.116 |
|  | **Completion of the sepsis bundle** |  |  |  |  |  |
|  | Completion of the administration of antibiotics within 3 hours | % | 4.977 | 0.782 | 31.680 | 0.089 |
|  | **Life-sustaining treatments during ICU stay** |  |  |  |  |  |
|  | Respiratory support |  |  |  |  |  |
|  | Mechanical ventilation | % | 12.006 | 1.318 | 109.341 | 0.027 |
|  | Additional ICU support |  |  |  |  |  |
|  | Fresh frozen plasma transfusion | % | 4.271 | 1.179 | 15.472 | 0.027 |
|  | Constant |  | 0.108 |  |  | 0.147 |
| 15 | **ICU characteristics** |  |  |  |  |  |
|  | Training programme in ICU |  |  |  |  |  |
|  | No | % | - | - | - | - |
|  | Yes | % | 0.161 | 0.033 | 0.795 | 0.025 |
|  | **Completion of the sepsis bundle** |  |  |  |  |  |
|  | Completion of the administration of antibiotics within 3 hours | % | 3.536 | 0.577 | 21.649 | 0.172 |
|  | **Life-sustaining treatments during ICU stay** |  |  |  |  |  |
|  | Respiratory support |  |  |  |  |  |
|  | Mechanical ventilation | % | 10.702 | 1.222 | 93.754 | 0.032 |
|  | Additional ICU support |  |  |  |  |  |
|  | Fresh frozen plasma transfusion | % | 5.010 | 1.419 | 17.685 | 0.012 |
|  | Constant |  | 0.117 |  |  | 0.157 |
| 16 | **ICU characteristics** |  |  |  |  |  |
|  | Training programme in ICU |  |  |  |  |  |
|  | No | % | - | - | - | - |
|  | Yes | % | 0.165 | 0.035 | 0.768 | 0.022 |
|  | **Life-sustaining treatments during ICU stay** |  |  |  |  |  |
|  | Respiratory support |  |  |  |  |  |
|  | Mechanical ventilation | % | 12.005 | 1.355 | 106.387 | 0.026 |
|  | Additional ICU support |  |  |  |  |  |
|  | Fresh frozen plasma transfusion | % | 4.361 | 1.296 | 14.671 | 0.017 |
|  | Constant |  | 0.328 |  |  | 0.364 |

**Table S32.** Hospital and intensive care unit characteristics according to intensive care unit survivability of patients with septic shock

| Variable | All cases  n= 74 | Survived  n= 43 | Died  n= 31 | P |
| --- | --- | --- | --- | --- |
| Participating hospital, no. (%) |  |  |  | 0.155 |
| 115 People's | 12 (16.2) | 2 (4.7) | 10 (32.3) |  |
| Bach Mai | 5 (6.8) | 3 (7.0) | 2 (6.5) |  |
| Bai Chay | 1 (1.4) | 1 (2.3) | 0 (0.0) |  |
| Can Tho | 4 (5.4) | 3 (7.0) | 1 (3.2) |  |
| Cho Ray | 18 (24.3) | 11 (25.6) | 7 (22.6) |  |
| Da Nang | 6 (8.1) | 3 (7.0) | 3 (9.7) |  |
| Dong Da | 1 (1.4) | 1 (2.3) | 0 (0.0) |  |
| Hanoi Medical University | 2 (2.7) | 2 (4.7) | 0 (0.0) |  |
| Hue | 8 (10.8) | 6 (14.0) | 2 (6.5) |  |
| Saint Paul | 0 | 0 | 0 |  |
| Thai Nguyen | 2 (2.7) | 1 (2.3) | 1 (3.2) |  |
| Thanh Nhan | 0 | 0 | 0 |  |
| Vietnam–Czechoslovakia Friendship | 15 (20.3) | 10 (23.3) | 5 (16.1) |  |
| Vinmec Times City International | 0 | 0 | 0 |  |
| **Hospital characteristics** |  |  |  |  |
| Type of hospital, no. (%) |  |  |  |  |
| Rural |  |  |  |  |
| Urban | 74 (100) | 43 (1000) | 31 (1000) | - |
| University affiliation, no. (%) |  |  |  | 0433 |
| No | 39 (52.7) | 21 (48.8) | 18 (58.1) |  |
| Yes | 35 (47.3) | 22 (51.2) | 13 (41.9) |  |
| Nature of ICU, no. (%) |  |  |  | - |
| Open | 0 | 0 | 0 |  |
| Closed | 74 (100) | 43 (100) | 31 (100) |  |
| Type of ICU, no. (%) |  |  |  | 0.146 |
| Medical | 38 (51.4) | 19 (44.2) | 19 (61.3) |  |
| Surgical | 0 | 0 | 0 |  |
| Mixed | 36 (48.6) | 24 (55.8) | 12 (38.7) |  |
| Nurse to patient ratio, no. (%) |  |  |  | 0.398 |
| 1 or more nurses : 1 patient | 0 | 0 | 0 |  |
| 1 nurse : 2 patients | 56 (75.7) | 31 (72.1) | 25 (80.6) |  |
| 1 nurse : 3 patients | 0 | 0 | 0 |  |
| 1 nurse : 4 or more patients | 18 (24.3) | 12 (27.9) | 6 (19.4) |  |
| Intensivist to patient ratio, no. (%) |  |  |  | 0.716 |
| 1 intensivist : 5 or fewer patients | 55 (74.3) | 31 (72.1) | 24 (77.4) |  |
| 1 intensivist : 6 to 8 patients | 13 (17.6) | 9 (20.9) | 4 (12.9) |  |
| 1 intensivist : 9 to 11 patients | 0 | 0 | 0 |  |
| 1 intensivist : 12 or more patients | 6 (8.1) | 3 (7.0) | 3 (9.7) |  |
| Training programme in ICU, no. (%) |  |  |  | 0.030 |
| No | 17 (23.0) | 6 (14.0) | 11 (35.5) |  |
| Yes | 57 (77.0) | 37 (86.0) | 20 (64.5) |  |

**Table S33.** Baseline characteristics according to intensive care unit survivability of patients with septic shock

| Variable | All cases  n= 74 | Survived  n= 43 | Died  n= 31 | P |
| --- | --- | --- | --- | --- |
| Age (year), median (IQR) | 64 (47.75-77) | 66 (51.77) | 63 (46.77) | 0.649 |
| Age (year), no. (%) |  |  |  | 0.716 |
| < 20 | 1 (1.4) | 0 (0.0) | 1 (3.2) |  |
| 20 - 39 | 7 (9.5) | 4 (9.3) | 3 (9.7) |  |
| 40 - 59 | 22 (29.7) | 12 (27.9) | 10 (32.3) |  |
| ≥ 60 | 44 (59.5) | 27 (62.8) | 17 (54.8) |  |
| Sex (male), no. (%) | 41 (55.4) | 21 (48.8) | 20 (64.5) | 0.181 |
| Collection batch, no. (%) |  |  |  | 0.067 |
| Collection 1 (Jan) | 24 (32.4) | 19 (44.2) | 5 (16.1) |  |
| Collection 2 (April) | 22 (29.7) | 10 (23.3) | 12 (38.7) |  |
| Collection 3 (July) | 16 (21.6) | 7 (16.3) | 9 (29.0) |  |
| Collection 4 (Oct) | 12 (16.2) | 7 (16.3) | 5 (16.1) |  |
| Admission type, no. (%) |  |  |  | 0.392 |
| Medical | 69 (93.2) | 39 (90.7) | 30 (96.8) |  |
| Elective surgical | 0 | 0 | 0 |  |
| Unscheduled surgical | 5 (6.8) | 4 (9.3) | 1 (3.2) |  |
| Admission source, no. (%) |  |  |  | 0.799 |
| Emergency department | 41 (55.4) | 22 (51.2) | 19 (61.3) |  |
| Operating room | 2 (2.7) | 1 (2.3) | 1 (3.2) |  |
| General wards | 12 (16.2) | 8 (18.6) | 4 (12.9) |  |
| Other ICUs or HDU | 4 (5.4) | 3 (7.0) | 1 (3.2) |  |
| Inter-hospital transfer | 14 (18.9) | 9 (20.9) | 5 (16.1) |  |
| Others | 1 (1.4) | 0 (0.0) | 1 (3.2) |  |
| Comorbidities, no. (%) |  |  |  |  |
| Cardiovascular disease | 20 (27.0) | 8 (18.6) | 12 (38.7) | 0.055 |
| Chronic lung disease | 5 (6.8) | 3 (7.0) | 2 (6.5) | >0.999 |
| Chronic neurological disease | 5 (6.8) | 5 (11.6) | 0 (0.0) | 0.070 |
| Chronic kidney disease | 7 (9.5) | 4 (9.3) | 3 (9.7) | >0.999 |
| Peptic ulcer disease | 3 (4.1) | 1 (2.3) | 2 (6.5) | 0.568 |
| Chronic liver disease | 7 (9.5) | 1 (2.3) | 6 (19.4) | 0.019 |
| Diabetes mellitus | 19 (25.7) | 12 (27.9) | 7 (22.6) | 0.605 |
| HIV infection | 0 | 0 | 0 | - |
| Connective tissue disease | 0 | 0 | 0 | - |
| Immunosuppression | 5 (6.8) | 3 (7.0) | 2 (6.5) | >0.999 |
| Haematological malignancies | 2 (2.7) | 1 (2.3) | 1 (3.2) | >0.999 |
| Solid malignant tumours | 2 (2.7) | 1 (2.3) | 1 (3.2) | >0.999 |

**Table S34.** Clinical and laboratory characteristics and severity of illness according to intensive care unit survivability of patients with septic shock

| Variable | All cases  n= 74 | Survived  n= 43 | Died  n= 31 | P |
| --- | --- | --- | --- | --- |
| **Vital signs** (on admission into ICU) | | | | |
| GCS, median (IQR) | 11 (7.5-14.5) | 12 (7.75-15) | 10 (6-13) | 0.044 |
| HR (beats per min), median (IQR) | 120 (100-130.5) |  |  |  |
| Temperature (^o^C), mean (SD) | 37.74 (1.01) | 37.63 (1.08) | 37.88 (0.90) | 0.231 |
| MBP (mmHg), mean(SD) | 53.45 (10.08) | 55.19 (6.37) | 51.03 (13.41) | 0.274 |
| SBP (mmHg), mean (SD) | 78.15 (18.22) | 82.30 (16.15) | 72.39 (19.60) | 0.084 |
| RR (breaths per min), median (IQR) | 25 (21-30) | 24 (20-30) | 25 (22-32) | 0.442 |
| **Blood investigations** | | | | |
| Total WBC (x10^9^/L), mean (SD) | 17.62 (10.09) | 18.70 (8.89) | 16.13 (11.53) | 0.145 |
| PLT (x10^9^/L), mean (SD) | 185.96 (147.33) | 222.58 (147.68) | 135.16 (133.04) | 0.001 |
| Hb (g/dL), mean (SD) | 11.37 (2.75) | 11.33 (2.81) | 11.42 (2.71) | 0.848 |
| Hct (%), mean (SD) | 35.04 (7.98) | 34.57 (8.33) | 35.70 (7.55) | 0.595 |
| K^+^ (mmol/L), mean (SD) | 3.91 (0.96) | 3.92 (0.87) | 3.90 (0.86) | 0.891 |
| Na^+^ (mmol/L), mean (SD) | 136.5 (7.88) | 136.58 (8.68 ) | 136.39 (6.75) | 0.917 |
| Creatinine (µmol/L), mean (SD) | 210.25 (145.01) | 204.80 (160.18) | 217.80 (123.05) | 0.218 |
| Bilirubin (µmol/l), mean (SD) | 31.64  (47.21) | 23.04 (35.87) | 42.75 (57.46) | 0.166 |
| pH, mean (SD) | 7.28 (0.14) | 7.31 (0.14) | 7.24 (0.14) | 0.006 |
| PaO_2_ (mmHg), mean (SD) | 118.67 (83.64) | 113.11 (75.80) | 126.01 (93.80) | 0.532 |
| FiO_2_, mean (SD) | 0.56 (0.25) | 0.51 (0.23) | 0.63 (0.26) | 0.042 |
| PaO_2_/FiO_2_ ratio, mean (SD) | 236.23 (144.23) | 230.13 (125.31) | 244.31 (167.87) | 0.959 |
| **Severity of illness scores** | | | | |
| qSOFA, median (IQR) | 2 (2-3) | 2 (2-3) | 3 (2-3) | 0.003 |
| qSOFA, no. (%) |  |  |  | 0.070 |
| 0 - 1 | 5 (6.8) | 5 (11.6) | 0 (0.0) |  |
| 2 - 3 | 69 (93.2) | 38 (88.4) | 31 (100) |  |
| SIRS, median (IQR) | 3 (2-4) | 3(2-3) | 3 (2-4) | 0.204 |
| SOFA, median (IQR) | 10 (7-12.25) | 9 (7-10) | 12 (10-14) | <0.001 |
| SOFA, no. (%) |  |  |  | 0.003 |
| 0 - 1 | 0 | 0 | 0 |  |
| 2 - 3 | 5 (6.8) | 4 (9.3) | 1 (3.2) |  |
| 4 - 5 | 4 (5.4) | 4 (9.3) | 0 (0.0) |  |
| 6 - 7 | 14 (18.9) | 11 (25.6) | 3 (9.7) |  |
| 8 - 9 | 12 (16.2) | 10 (23.3) | 2 (6.5) |  |
| 10 - 11 | 16 (21.6) | 8 (18.6) | 8 (25.8) |  |
| 12 - 14 | 15 (20.3) | 4 (9.3) | 11 (35.5) |  |
| > 14 | 8 (10.8) | 6 (19.4) | 2 (4.7) |  |
| SOFA, no. (%) |  |  |  | 0.001 |
| 0 - 3 | 5 (6.8) | 4 (9.3) | 1 (3.2) |  |
| 4 - 7 | 18 (24.3) | 15 (34.9) | 3 (9.7) |  |
| 8 - 9 | 12 (16.2) | 10 (23.3) | 2 (6.5) |  |
| 10 - 11 | 16 (21.6) | 8 (18.6) | 8 (25.8) |  |
| ≥ 12 | 23(31.1) | 6 (14.0) | 17 (54.8) |  |
| APACHE II, median (IQR) | 23 (16.75-28) | 20 (14-25) | 26 (22-31) | 0.001 |
| APACHE II, no. (%) |  |  |  | 0.031 |
| 0 - 4 | 0 | 0 | 0 |  |
| 5 - 9 | 2 (2.7) | 2 (4.7) | 0 (0.0) |  |
| 10 - 14 | 12 (16.2) | 10 (23.3) | 2 (6.5) |  |
| 15 - 19 | 9 (12.2) | 8 (18.6) | 1 (3.2) |  |
| 20 - 24 | 22 (29.7) | 12 (27.9) | 10 (32.3) |  |
| 25 - 29 | 13 (17.6) | 4 (9.3) | 9 (29.0) |  |
| 30 - 34 | 9 (12.2) | 4 (9.3) | 5 (16.1) |  |
| > 34 | 7 (9.5) | 3 (7.0) | 4 (12.9) |  |

**Table S35.** Sites of infection and microbiology according to intensive care unit survivability of patients with septic shock

| Variable | All cases  n= 74 | Survived  n= 43 | Died  n=31 | P |
| --- | --- | --- | --- | --- |
| **Site of Infection** | | | | |
| Respiratory, no. (%) | 38 (51.4) | 21 (48.8) | 17 (54.8) | 0.610 |
| Urinary tract, no. (%) | 10 (13.5) | 8 (18.6) | 2 (6.5) | 0.177 |
| Abdominal, no. (%) | 26 (35.1) | 14 (32.6) | 12 (38.7) | 0.584 |
| Neurological, no. (%) | 2 (207) | 1 (2.3) | 1 (3.2) | >0.999 |
| Bones or joints, no. (%) | 1 (1.4) | 1 (2.3) | 0 (0.0) | >0.999 |
| Skin or cutaneous sites, no. (%) | 6 (8.1) | 4 (9.3) | 2 (6.5) | >0.999 |
| Intravascular catheter, no. (%) | 0 | 0 | 0 |  |
| Infective endocarditis, no. (%) | 0 | 0 | 0 |  |
| Primary bacteraemia, no. (%) | 2 (2.7) | 1 (2.3) | 1 (3.2) | >0.999 |
| Systemic, no. (%) | 3 (4.1) | 1 (2.3) | 2 (6.5) | 0.568 |
| Others, no. (%) | - | - | - | - |
| **Microbiology** | | | | |
| No pathogens detected, no. (%) | 23 (31.1) | 16 (37.2) | 7 (22.6) | 0.180 |
| Gram negative bacteria, no. (%) | 43 (58.1) | 24 (55.8) | 19 (61.3) | 0.638 |
| *Klebsiella pneumonia* | 6 (8.1) | 3 (7.0) | 3 (9.7) | 0.690 |
| *Acinetobacter baumannii* | 12 (16.2) | 6 (14.0) | 6 (19.4) | 0.534 |
| *Escherichia coli* | 16 (21.6) | 10 (23.3) | 6 (19.4) | 0.688 |
| *Pseudomonas aeruginosa* | 5 (6.8) | 3 (7.0) | 2 (6.5) | >0.999 |
| *Stenotrophomonas maltophilia* | 0 | 0 | 0 | - |
| *Proteus species* | 10 (13.5) | 7 (16.3) | 3 (9.7) | 0.505 |
| *Enterobacter cloacae* | 0 | 0 | 0 | - |
| *Others* | 0 | 0 | 0 | - |
| Gram positive bacteria, no. (%) | 6 (8.1) | 3 (7.0) | 3 (9.7) | 0.690 |
| *Enterococcus* | 1 (1.4) | 1 (2.3) | 0 (0.0) | >0.999 |
| *MSSA* | 0 | 0 | 0 | - |
| *MRSA* | 3 (4.1) | 2 (4.7) | 1 (3.2) | >0.999 |
| *Other Streptococcus species* | 2 (2.7) | 0 (0.0) | 2 (6.5) | 0.172 |
| *Streptococcus pneumonia* | 0 | 0 | 0 | - |
| Fungi, no. (%) |  |  |  |  |
| *Candida species* | 0 | 0 | 0 | - |
| *Aspergillus species* | 0 | 0 | 0 | - |
| *Others* | 0 | 0 | 0 | - |
| Viruses, no. (%) | 2 (2.7) | 0 | 2 (6.5) | 0.172 |
| *Influenza* | 1 (1.4) | 0 (0.0) | 1 (3.2) | 0.419 |
| *Others* | 0 | 0 | 0 | - |
| *Dengue* | 1 (1.4) | 0 (0.0) | 1 (3.2) | 0.419 |
| Other pathogens, no. (%) |  |  |  |  |
| *Anaerobes* | 0 | 0 | 0 | - |
| *Mycobacterium tuberculosis* | 2 (2.7) | 1 (2.3) | 1 (3.2) | >0.999 |
| *Malaria* | 0 | 0 | 0 | - |

**Table S36.** Completion of sepsis bundle elements according to intensive care unit survivability of patients with septic shock

| Variable | All cases  n= 74 | Survived  n= 43 | Died  n=31 | P |
| --- | --- | --- | --- | --- |
| **Timing of antibiotics administration** | | | | |
| Performed within 24 hours, no. (%) | n=68 | n=38 | n=30 | 0.954 |
| 0-60 minutes | 51 (75.0) | 29 (76.3) | 22 (73.3) |  |
| 61-120 minutes | 10 (14.7) | 5 (13.2) | 5 (16.7) |  |
| 121-180 minutes | 2 (2.9) | 1 (2.6) | 1 (3.3) |  |
| >180 minutes | 5 (7.4) | 3 (7.9) | 2 (6.7) |  |
| Not performed within 24 hours, no. (%) | 0 | 0 | 0 | - |
| Timing of antibiotics administration, median (IQR), minutes | 30 (6.25-63.75) | 32.5 (0-63.25) | 30 (10-71.25) | 0.965 |
| **Timing of obtaining blood cultures** | | | | |
| Performed within 24 hours, no. (%) | n=54 | n=27 | n=27 | 0.699 |
| 0-60 minutes | 29 (53.7) | 16 (48.1) | 16 (59.3) |  |
| 61-120 minutes | 9 (16.7) | 4 (14.8) | 5 (18.5) |  |
| 121-180 minutes | 1 (1.9) | 1 (3.7) | 0 (0.0) |  |
| >180 minutes | 15 (27.8) | 9 (33.3) | 6 (22.2) |  |
| Not performed within 24 hours, no. (%) | 0 | 0 | 0 | - |
| Timing of obtaining blood cultures, median (IQR), minutes | 55 (19.25-233.25) | 73 (30-342.) | 30 (15-110) | 0.161 |
| **Timing of obtaining lactate measurement** | | | | |
| Performed within 24 hours, no. (%) | n=62 | n=36 | n=26 | 0.657 |
| 0-60 minutes | 38 (61.3) | 21 (58.3) | 17 (65.4) |  |
| 61-120 minutes | 5 (8.1) | 2 (5.6) | 3 (11.5) |  |
| 121-180 minutes | 1 (1.6) | 1 (2.8) | 0 (0.0) |  |
| >180 minutes | 18 (29.0) | 12 (33.3) | 6 (23.1) |  |
| Not performed within 24 hours, no. (%) | 0 | 0 | 0 | - |
| Timing of obtaining lactate measurement, median (IQR), minutes | 30 (13-313.75) | 52.5 (15-361.25) | 22.5 (8.75-138.75) | 0.194 |

**Table S37.** Completion of the sepsis bundle of care and the initial administration of antibiotics according to intensive care unit survivability of patients with septic shock

| Variable | All cases  n= 72 | Survived  n= 41 | Died  n= 31 | P |
| --- | --- | --- | --- | --- |
| Completion of the sepsis bundle within 1 hour. no. (%) | 20 (27.8) | 10 (24.4) | 10 (32.3) | 0.460 |
| Completion of the initial administration of antibiotics within 1 hour. no. (%) | 51 (70.8) | 29 (70.7) | 22 (71.0) | 0.983 |
| Permutations of the completed elements within 1 hour, no. (%) |  |  |  | 0.732 |
| No elements completed | 9 (12.5) | 6 (14.6) | 3 (9.7) |  |
| Antibiotics only | 17 (23.6) | 11 (26.8) | 6 (19.4) |  |
| Blood cultures only | 1 (1.4) | 0 (0.0) | 1 (3.2) |  |
| Lactate only | 10 (13.9) | 6 (14.6) | 4 (12.9) |  |
| Antibiotics + Lactate | 7 (9.7) | 5 (12.2) | 2 (6.5) |  |
| Antibiotics + Blood cultures | 7 (9.7) | 3 (7.3) | 4 (12.9) |  |
| Blood cultures + Lactate | 1 (1.4) | 0 (0.0) | 1 (3.2) |  |
| Antibiotics + Blood cultures + Lactate | 20 (27.8) | 10 (24.4) | 10 (32.3) |  |
| Completion of the sepsis bundle within 3 hours. no. (%) | 27 (37.5) | 13 (31.7) | 14 (45.2) | 0.243 |
| Completion of the initial administration of antibiotics within 3 hours. no. (%) | 63 (87.5) | 35 (85.4) | 28 (90.3) | 0.529 |
| Permutations of the completed elements within 3 hours, no. (%) |  |  |  | 0.388 |
| No elements completed | 2 (2.8) | 1 (2.4) | 1 (3.2) |  |
| Antibiotics only | 15 (20.8) | 11 (26.8) | 4 (12.9) |  |
| Blood cultures only | 1 (1.4) | 1 (2.4) | 0 (0.0) |  |
| Lactate only | 6 (8.3) | 4 (9.8) | 2 (6.5) |  |
| Antibiotics + Lactate | 10 (13.9) | 7 (17.1) | 3 (9.7) |  |
| Antibiotics + Blood cultures | 11 (15.3) | 4 (9.8) | 7 (22.6) |  |
| Blood cultures + Lactate | 0 | 0 | 0 |  |
| Antibiotics + Blood cultures + Lactate | 27 (37.5) | 13 (31.7) | 14 (45.2) |  |

**Table S38.** Life-sustaining treatments during ICU stay and outcomes according to intensive care unit survivability of patients with septic shock

| Variable | All cases  n= 74 | Survived  n= 43 | Died  n= 31 | P |
| --- | --- | --- | --- | --- |
| **Life-sustaining treatments during ICU stay** | | | | |
| Respiratory support, no. (%) and median (IQR), days |  |  |  |  |
| Mechanical ventilation | 61 (82.4) | 30 (69.8) | 31 (100) | 0.001 |
| Duration of mechanical ventilation | 7 (3-13) | 2 (1-2) | 6.5 (2-13.25) | 0.403 |
| Non-invasive ventilation | 4 (5.4) | 3 (7.0) | 1 (3.2) | 0.635 |
| Duration of non-invasive ventilation | 2 (1.25-5.75) | 2 (1-2) | 7 (7-7) | 0.157 |
| High-flow nasal oxygen | 11 (14.9) | 10 (23.3) | 1 (3.2) | 0.020 |
| Duration of high-flow nasal oxygen | 2 (1-3) | 2 (1-3.25) | 3 (3-3) | 0.515 |
| Additional ICU support, no. (%) |  |  |  |  |
| Vasopressors/inotropes | 74 (100) | 43 (100) | 31 (100) | - |
| Renal replacement therapy | 43 (58.1) | 16 (37.2) | 27 (87.1) | <0.001 |
| Red blood cell transfusion | 31 (41.9) | 16 (37.2) | 15 (48.4) | 0.336 |
| Platelet transfusion | 18 (24.3) | 7 (16.3) | 11 (35.5) | 0.057 |
| Fresh frozen plasma transfusion | 24 (32.4) | 8 (18.6) | 16 (51.6) | 0.003 |
| Surgical source control | 8 (10.8) | 6 (14.0) | 2 (6.5) | 0.455 |
| Non-surgical source control | 13 (17.6) | 9 (20.9) | 4 (12.9) | 0.371 |
| Length of of surgical source control, median minutes (IQR) | 270 (140-552.5) | 282.5 (78.75-791.25) | 270 (270-270) | >0.999 |
| Length of of surgical source control, n (%) |  |  |  | - |
| <12 hours | 8 (100) | 6 (100) | 2(100) |  |
| 12-24 hours | 0 | 0 | 0 |  |
| >24 hours | 0 | 0 | 0 |  |
| **Outcomes** |  |  |  |  |
| Outcomes |  |  |  | <0.001 |
| Alive upon current hospital discharge, no. (%) | 35 (47.3) | 35 (81.4) | 0 (0.0) |  |
| Alive upon discharge from current ICU stay, but died in current hospital stay, no. (%) | 8 (10.8) | 8 (18.6) | 0 (0.0) |  |
| Alive upon discharge from current ICU stay, but still in current hospital stay after 90 days, no. (%) | 0 | 0 | 0 |  |
| Still in current ICU stay after 90 days, no. (%) | 0 | 0 | 0 |  |
| Died in current ICU stay, no. (%) | 31 (41.9) | 0 (0.0) | 31 (100) |  |
| Mortality, no. (%) |  |  |  |  |
| Hospital | 39 (52.7) | 8 (18.6) | 31 (100) | <0.001 |
| Length of stay, median days (IQR) |  |  |  |  |
| Hospital | 14 (8-20) | 18 (12-24) | 10 (3-18) | 0.001 |
| ICU | 8 (5-15.25) | 11 (6-16) | 7 (2-14) | 0.108 |

**Table S39.** Factors associated with intensive care unit mortality in patients with septic shock: bivariate regression analyses

| Factor | Frequency | OR | 95.0% CI for OR | | P |
| --- | --- | --- | --- | --- | --- |
|  |  |  | Lower | Upper |  |
| Participating hospital |  |  |  |  |  |
| 115 People's | 12 | - | - | - |  |
| Bach Mai | 5 | 0.133 | 0.013 | 1.393 | 0.092 |
| Bai Chay | 1 | 0.000 | 0.000 | - | >0.999 |
| Can Tho | 4 | 0.067 | 0.004 | 1.017 | 0.051 |
| Cho Ray | 18 | 0.127 | 0.021 | 0.762 | 0.024 |
| Da Nang | 6 | 0.200 | 0.022 | 1.816 | 0.153 |
| Dong Da | 1 | 0.000 | 0.000 | - | >0.999 |
| Hanoi Medical University | 2 | 0.000 | 0.000 | - | 0.016 |
| Hue | 8 | 0.067 | 0.007 | 0.605 | 0.318 |
| Thai Nguyen | 2 | 0.200 | 0.008 | 4.716 | 0.015 |
| Vietnam–Czechoslovakia Friendship | 15 | 0.100 | 0.016 | 0.642 | 0.038 |
| **Hospital characteristics** |  |  |  |  |  |
| University affiliation |  |  |  |  |  |
| No | 39 | - | - | - | - |
| Yes | 35 | 0.689 | 0.272 | 1.749 | 0.434 |
| **ICU characteristics** |  |  |  |  |  |
| Type of ICU |  |  |  |  |  |
| Medical | 38 | - | - | - |  |
| Mixed | 36 | 0.500 | 0.195 | 1.281 | 0.149 |
| Nurse to patient ratio |  |  |  |  |  |
| 1 nurse : 4 or more patients | 56 | - | - | - |  |
| 1 or more nurses : 1 patient | 18 | 1.613 | 0.530 | 4.907 | 0.400 |
| Intensivist to patient ratio |  |  |  |  |  |
| 1 intensivist : 5 or fewer patients | 55 | - | - | - | 0.645 |
| 1 intensivist : 6 to 8 patients | 13 | 0.574 | 0.158 | 2.091 | 0.400 |
| 1 intensivist : 12 or more patients | 6 | 1.292 | 0.239 | 6.977 | 0.766 |
| Training programme in ICU |  |  |  |  |  |
| No | 17 | - | - | - |  |
| Yes | 57 | 0.295 | 0.095 | 0.916 | 0.035 |
| **Baseline characteristics** |  |  |  |  |  |
| Age (year) | 74 | 0.992 | 0.967 | 1.017 | 0.520 |
| Age (year) group |  |  |  |  |  |
| < 20 | 1 | - | - | - | 0.962 |
| 20 - 39 | 7 | 1.000 | 0.000 | - | >0.999 |
| 40 - 59 | 22 | 1.000 | 0.000 | - | >0.999 |
| ≥ 60 | 44 | 1.000 | 0.000 | - | >0.999 |
| Sex (male) | 41 | 1.905 | 0.738 | 4.916 | 0.183 |
| Collection batch |  |  |  |  |  |
| Collection 1 (Jan) | 24 | - | - | - | 0.083 |
| Collection 2 (April) | 22 | 4.560 | 1.250 | 16.634 | 0.022 |
| Collection 3 (July) | 16 | 4.886 | 1.211 | 19.714 | 0.026 |
| Collection 4 (Oct) | 12 | 2.714 | 0.598 | 12.317 | 0.196 |
| Admission type |  |  |  |  |  |
| Medical | 69 | - | - | - |  |
| Unscheduled surgical | 5 | 0.325 | 0.035 | 3.060 | 0.326 |
| Admission source |  |  |  |  |  |
| Emergency department | 41 | - | - | - | 0.924 |
| Operating room | 2 | 1.158 | 0.68 | 19.798 | 0.919 |
| General wards | 12 | 0.579 | 0.150 | 2.229 | 0.427 |
| Other ICUs or HDU | 4 | 0.386 | 0.037 | 4.027 | 0.426 |
| Inter-hospital transfer | 14 | 0.643 | 0.184 | 2.254 | 0.490 |
| Others | 1 | 1870549843 | 0.000 | - | >0.999 |
| Comorbidities |  |  |  |  |  |
| Cardiovascular disease | 20 | 2.763 | 0.962 | 7.933 | 0.059 |
| Chronic lung disease | 5 | 0.920 | 0.144 | 5.860 | 0.929 |
| Chronic neurological disease | 5 | 0.000 | 0.000 | - | 0.999 |
| Chronic kidney disease | 7 | 1.045 | 0.217 | 5.040 | 0.957 |
| Peptic ulcer disease | 3 | 2.897 | 0.251 | 33.454 | 0.394 |
| Chronic liver disease | 7 | 10.080 | 1.146 | 88.648 | 0.037 |
| Diabetes mellitus | 19 | 0.753 | 0.257 | 2.205 | 0.605 |
| Immunosuppression | 5 | 0.920 | 0.144 | 5.860 | 0.929 |
| Haematological malignancies | 2 | 1.400 | 0.084 | 23.280 | 0.815 |
| Solid malignant tumours | 2 | 1.400 | 0.084 | 23.280 | 0.815 |
| **Vital signs** |  |  |  |  |  |
| GCS | 73 | 0.878 | 0.766 | 1.007 | 0.063 |
| HR (beats per min) | 74 | 1.046 | 1.017 | 1.075 | 0.002 |
| Temperature (^o^C) | 74 | 1.287 | 0.809 | 2.049 | 0.287 |
| MBP (mmHg) | 74 | 0.956 | 0.905 | 1.009 | 0.101 |
| SBP (mmHg) | 74 | 0.965 | 0.934 | 0.997 | 0.032 |
| RR (breaths per min) | 74 | 1.028 | 0.957 | 1.104 | 0.453 |
| **Blood investigations** |  |  |  |  |  |
| Total WBC (x10^9^/L) | 74 | 0.974 | 0.929 | 1.022 | 0.280 |
| PLT (x10^9^/L) | 74 | 0.995 | 0.991 | 0.999 | 0.018 |
| Hb (g/dL) | 74 | 1.012 | 0.854 | 1.198 | 0.893 |
| Hct (%) | 74 | 1.018 | 0.960 | 1.080 | 0.544 |
| K^+^ (mmol/L) | 74 | 0.977 | 0.569 | 1.678 | 0.934 |
| Na^+^ (mmol/L) | 74 | 0.997 | 0.940 | 1.057 | 0.916 |
| Creatinine (µmol/L) | 74 | 1.001 | 0.997 | 1.004 | 0.703 |
| Bilirubin (µmol/l) | 71 | 1.010 | 0.998 | 1.023 | 0.113 |
| pH | 73 | 0.021 | 0.001 | 0.870 | 0.042 |
| PaO_2_ (mmHg) | 72 | 1.002 | 0.996 | 1.008 | 0.518 |
| FiO_2_ | 73 | 6.776 | 0.970 | 47.319 | 0.054 |
| PaO_2_/FiO_2_ ratio | 72 | 1.001 | 0.997 | 1.004 | 0.679 |
| **Severity of illness scores** |  |  |  |  |  |
| qSOFA | 74 | 3.869 | 1.570 | 9.535 | 0.003 |
| qSOFA |  |  |  |  |  |
| 0 - 1 | 5 | - | - | - |  |
| 2 - 3 | 69 | 1.32E+9 | 0.000 | - | 0.999 |
| SIRS | 74 | 1.469 | 0.872 | 2.474 | 0.148 |
| SOFA | 74 | 1.349 | 1.138 | 1.600 | 0.001 |
| SOFA |  |  |  |  |  |
| 2 - 3 | 5 | - | - | - | 0.027 |
| 4 - 5 | 4 | 0.000 | 0.000 | - | 0.999 |
| 6 - 7 | 14 | 1.091 | 0.086 | 13.778 | 0.946 |
| 8 - 9 | 12 | 0.800 | 0.056 | 11.504 | 0.870 |
| 10 - 11 | 16 | 4.000 | 0.363 | 44.113 | 0.258 |
| 12 - 14 | 15 | 11.000 | 0.928 | 130.324 | 0.057 |
| > 14 | 8 | 12.000 | 0.796 | 180.974 | 0.073 |
| SOFA |  |  |  |  |  |
| 0 - 6 | 11 | - | - | - | 0.003 |
| 7 - 9 | 24 | 0.900 | 0.139 | 5.844 | 0.912 |
| 10 - 12 | 16 | 4.500 | 0.730 | 27.739 | 0.105 |
| 13 - 14 | 15 | 12.375 | 1.828 | 83.767 | 0.010 |
| > 14 | 8 | 13.500 | 1.473 | 123.743 | 0.021 |
| SOFA |  |  |  |  |  |
| 0 - 3 | 5 | - | - | - | 0.003 |
| 4 - 7 | 18 | 0.800 | 0.065 | 9.919 | 0.862 |
| 8 - 9 | 12 | 0.800 | 0.056 | 11.504 | 0.870 |
| 10 - 11 | 16 | 4.000 | 0.363 | 11.113 | 0.258 |
| ≥ 12 | 23 | 11.333 | 1.048 | 122.549 | 0.046 |
| APACHE II | 74 | 1.106 | 1.034 | 1.184 | 0.003 |
| APACHE II |  |  |  |  |  |
| 5 - 9 | 2 | - | - | - | 0.122 |
| 10 - 14 | 12 | 323094693.0 | 0.000 | - | 0.999 |
| 15 - 19 | 9 | 201934183.1 | 0.000 | - | 0.999 |
| 20 - 24 | 22 | 1346227887 | 0.000 | - | 0.999 |
| 25 - 29 | 13 | 3634815296 | 0.000 | - | 0.999 |
| 30 - 34 | 9 | 2019341831 | 0.000 | - | 0.999 |
| > 34 | 7 | 2153964620 | 0.000 | - | 0.999 |
| APACHE II |  |  |  |  |  |
| 0 - 9 | 2 | - | - | - | 0.074 |
| 10 - 14 | 12 | 323094626.8 | 0.000 | - | 0.999 |
| 15 - 19 | 9 | 201934141.8 | 0.000 | - | 0.999 |
| 20 - 24 | 22 | 1346227612 | 0.000 | - | 0.999 |
| 25 - 29 | 13 | 3634814552 | 0.000 | - | 0.999 |
| ≥ 30 | 16 | 2077036887 | 0.000 | - | 0.999 |
| **Site of Infection** |  |  |  |  |  |
| Respiratory | 38 | 1.272 | 0.504 | 3.212 | 0.611 |
| Urinary tract | 10 | 0.302 | 0.059 | 1.533 | 0.149 |
| Abdominal | 26 | 1.308 | 0.499 | 3.430 | 0.585 |
| Neurological | 2 | 1.400 | 0.084 | 23.280 | 0.815 |
| Bones or joints | 1 | 0.000 | 0.000 | - | >0.999 |
| Skin or cutaneous sites | 6 | 0.672 | 0.115 | 3.924 | 0.659 |
| Primary bacteraemia | 2 | 1.400 | 0.084 | 23.280 | 0.815 |
| Systemic | 3 | 2.897 | 0.251 | 33.454 | 0.394 |
| **Microbiology** |  |  |  |  |  |
| Pathogens detection |  |  |  |  |  |
| No pathogens detected | 23 | 0.492 | 0.173 | 1.399 | 0.183 |
| Gram negative bacteria | 43 | 1.253 | 0.489 | 3.210 | 0.638 |
| Gram positive bacteria | 6 | 1.429 | 0.268 | 7.601 | 0.676 |
| Viruses | 2 | 2395359282 | 0.000 | - | 0.999 |
| Other pathogens | 2 | 1.400 | 0.084 | 23.280 | 0.815 |
| **Completion of sepsis bundle elements** |  |  |  |  |  |
| Timing of antibiotics administration |  |  |  |  |  |
| 0-60 minutes | 51 | - | - | - | 0.974 |
| 61-120 minutes | 10 | 1.318 | 0.339 | 5.125 | 0.690 |
| 121-180 minutes | 2 | 1.318 | 0.78 | 22.263 | 0.848 |
| >180 minutes | 5 | 0.879 | 0.135 | 5.719 | 0.892 |
| Timing of obtaining blood cultures |  |  |  |  |  |
| 0-60 minutes | 29 | - | - | - | 0.804 |
| 61-120 minutes | 9 | 1.016 | 0.226 | 4.573 | 0.984 |
| 121-180 minutes | 1 | 0.000 | 0.000 | 0 | >0.999 |
| >180 minutes | 15 | 0.542 | 0.153 | 1.921 | 0.343 |
| Timing of obtaining lactate measurement |  |  |  |  |  |
| 0-60 minutes | 38 | - | - | - | 0.732 |
| 61-120 minutes | 5 | 1.853 | 0.277 | 12.389 | 0.525 |
| 121-180 minutes | 1 | 0.000 | 0.000 | - | >0.999 |
| >180 minutes | 18 | 0.618 | 0.192 | 1.990 | 0.420 |
| Completion of the sepsis bundle within 1 hour | 20 | 1.476 | 0.523 | 4.163 | 0.462 |
| Completion of the initial administration of antibiotics within 1 hour | 51 | 1.011 | 0.362 | 2.824 | 0.983 |
| Permutations of the completed elements within 1 hour |  |  |  |  |  |
| No elements completed | 9 | - | - | - | 0.944 |
| Antibiotics only | 17 | 1.091 | 0.198 | 6.007 | 0.920 |
| Blood cultures only | 1 | 3230949729 | 0.000 | - | >0.999 |
| Lactate only | 10 | 1.333 | 0.204 | 8.708 | 0.764 |
| Antibiotics + Lactate | 7 | 0.800 | 0.093 | 6.848 | 0.839 |
| Antibiotics + Blood cultures | 7 | 2.667 | 0.347 | 20.508 | 0.346 |
| Blood cultures + Lactate | 1 | 3230949729 | 0.000 | - | >0.999 |
| Full bundle | 20 | 2.000 | 0.388 | 10.309 | 0.407 |
| Completion of the sepsis bundle within 3 hours | 27 | 1.774 | 0.675 | 4.660 | 0.245 |
| Completion of the initial administration of antibiotics within 3 hours | 63 | 1.600 | 0.367 | 6.975 | 0.532 |
| Permutation of the completed elements of 3-hour sepsis bundle |  |  |  |  |  |
| No elements completed | 2 | - | - | - | 0.529 |
| Antibiotics only | 15 | 0.364 | 0.018 | 7.295 | 0.508 |
| Blood cultures only | 1 | 0.000 | 0.000 | - | >0.999 |
| Lactate only | 6 | 0.500 | 0.500 | 12.898 | 0.676 |
| Antibiotics + Lactate | 10 | 0.429 | 0.429 | 9.364 | 0.590 |
| Antibiotics + Blood cultures | 11 | 1.750 | 1.750 | 36.287 | 0.718 |
| Full bundle | 27 | 1.077 | 1.077 | 19.046 | 0.960 |
| **Life-sustaining treatments during ICU stay** |  |  |  |  |  |
| Respiratory support |  |  |  |  |  |
| Mechanical ventilation | 61 | 1669323700 | 0.000 | - | 0.998 |
| Duration of mechanical ventilation | 59 | 0.995 | 0.943 | 1.051 | 0.869 |
| Non-invasive ventilation | 4 | 0.444 | 0.044 | 4.487 | 0.492 |
| High-flow nasal oxygen | 11 | 0.110 | 0.013 | 0.911 | 0.041 |
| Duration of high-flow nasal oxygen | 11 | 1.241 | 0.341 | 4.523 | 0.743 |
| Additional ICU support |  |  |  |  |  |
| Renal replacement therapy | 43 | 11.391 | 3.368 | 38.529 | <0.001 |
| Red blood cell transfusion | 31 | 1.582 | 0.620 | 4.039 | 0.337 |
| Platelet transfusion | 18 | 2.829 | 0.947 | 8.446 | 0.062 |
| Fresh frozen plasma transfusion | 24 | 4.667 | 1.646 | 13.232 | 0.004 |
| Surgical source control | 8 | 0.425 | 0.080 | 2.265 | 0.316 |
| Non-surgical source control | 13 | 0.560 | 0.155 | 2.016 | 0.375 |

**Table S40.** Factors associated with intensive care unit mortality in patients with septic shock: multivariate logistic regression analyses (backward elimination)

| Step | Factor | Unit | OR | 95.0% CI for OR | | P |
| --- | --- | --- | --- | --- | --- | --- |
|  |  |  |  | Lower | Upper |  |
| 1 | **ICU characteristics** |  |  |  |  |  |
|  | Type of ICU |  |  |  |  |  |
|  | Medical | % | - | - | - | - |
|  | Mixed | % | 0.582 | 0.034 | 10.079 | 0.710 |
|  | Training programme in ICU |  |  |  |  |  |
|  | No | % | - | - | - | - |
|  | Yes | % | 0.000 | 0.000 | - | 0.995 |
|  | **Baseline characteristics** |  |  |  |  |  |
|  | Comorbidities |  |  |  |  |  |
|  | Cardiovascular disease | % | 5.014 | 0.483 | 52.056 | 0.177 |
|  | Chronic liver disease | % | 5.644 | 0.011 | 2926.677 | 0.587 |
|  | **Severity of illness scores** |  |  |  |  |  |
|  | SOFA |  |  |  |  |  |
|  | 0 - 3 | % | - | - | - | 0.647 |
|  | 4-7 | % | 0.000 | 0.000 | - | >0.999 |
|  | 8-9 | % | 0.000 | 0.000 | - | 0.999 |
|  | 10 - 11 | % | 0.003 | 0.000 | - | >0.999 |
|  | ≥12 | % | 0.005 | 0.000 | - | >0.999 |
|  | **Site of Infection** |  |  |  |  |  |
|  | Urinary tract | % | 0.000 | 0.000 | - | 0.995 |
|  | **Completion of sepsis bundle** |  |  |  |  |  |
|  | Completion of the sepsis bundle within 1 hour | % | 0.000 | 0.000 | - | 0.995 |
|  | Completion of the administration of antibiotics within 1 hour | % | 0.000 | 0.000 | - | 0.995 |
|  | Completion of the sepsis bundle within 3 hours | % | 599758895911.178 | 0.000 | - | 0.996 |
|  | Completion of the administration of antibiotics within 3 hours | % | 22107316.665 | 0.000 | - | 0.996 |
|  | **Life-sustaining treatments during ICU stay** |  |  |  |  |  |
|  | Respiratory support |  |  |  |  |  |
|  | High-flow nasal oxygen | % | 220894634611.812 | 0.000 | - | 0.996 |
|  | Additional ICU support |  |  |  |  |  |
|  | Renal replacement therapy | % | 3145076359033321000000000.000 | 0.000 | - | 0.995 |
|  | Platelet transfusion | % | 1782686629601.350 | 0.000 | - | 0.995 |
|  | Fresh frozen plasma transfusion | % | 5.630 | 0.363 | - | 0.217 |
|  | Constant |  | 0.000 |  |  | >0.999 |
| 2 | **ICU characteristics** |  |  |  |  |  |
|  | Type of ICU |  |  |  |  |  |
|  | Medical | % | - | - | - | - |
|  | Mixed | % | 0.391 | 0.021 | 7.380 | 0.531 |
|  | Training programme in ICU |  |  |  |  |  |
|  | No | % | - | - | - | - |
|  | Yes | % | 0.000 | 0.000 | - | 0.996 |
|  | **Baseline characteristics** |  |  |  |  |  |
|  | Comorbidities |  |  |  |  |  |
|  | Cardiovascular disease | % | 4.500 | 0.419 | 48.290 | 0.214 |
|  | Chronic liver disease | % | 19.430 | 0.018 | 21319.133 | 0.406 |
|  | **Severity of illness scores** |  |  |  |  |  |
|  | SOFA |  |  |  |  |  |
|  | 0 - 3 | % | - | - | - | 0.556 |
|  | 4-7 | % | 3.232 | 0.000 | - | >0.999 |
|  | 8-9 | % | 0.000 | 0.000 | - | >0.999 |
|  | 10 - 11 | % | 68.543 | 0.000 | - | >0.999 |
|  | ≥12 | % | 109.979 | 0.000 | - | >0.999 |
|  | **Site of Infection** |  |  |  |  |  |
|  | Urinary tract | % | 0.000 | 0.000 | - | 0.995 |
|  | **Completion of sepsis bundle** |  |  |  |  |  |
|  | Completion of the sepsis bundle within 1 hour | % | 0.000 | 0.000 | - | 0.996 |
|  | Completion of the administration of antibiotics within 1 hour | % | 0.000 | 0.000 | - | 0.996 |
|  | Completion of the sepsis bundle within 3 hours | % | 56211742.668 | 0.000 | - | 0.996 |
|  | **Life-sustaining treatments during ICU stay** |  |  |  |  |  |
|  | Respiratory support |  |  |  |  |  |
|  | High-flow nasal oxygen | % | 3197576.126 | 0.000 | - | 0.997 |
|  | Additional ICU support |  |  |  |  |  |
|  | Renal replacement therapy | % | 17446837159202350.000 | 0.000 | - | 0.996 |
|  | Platelet transfusion | % | 254221758.473 | 0.000 | - | 0.996 |
|  | Fresh frozen plasma transfusion | % | 7.538 | 0.480 | 118.469 | 0.151 |
|  | Constant |  | 0.138 |  |  | >0.999 |
| 3 | **ICU characteristics** |  |  |  |  |  |
|  | Type of ICU |  |  |  |  |  |
|  | Medical | % | - | - | - | - |
|  | Mixed | % | 0.372 | 0.020 | 7.047 | 0.510 |
|  | Training programme in ICU |  |  |  |  |  |
|  | No | % | - | - | - | - |
|  | Yes | % | 0.003 | 0.000 | 1.352 | 0.063 |
|  | **Baseline characteristics** |  |  |  |  |  |
|  | Comorbidities |  |  |  |  |  |
|  | Cardiovascular disease | % | 5.244 | 0.517 | 53.235 | 0.161 |
|  | Chronic liver disease | % | 25.611 | 0.011 | 60693.057 | 0.413 |
|  | **Severity of illness scores** |  |  |  |  |  |
|  | SOFA |  |  |  |  |  |
|  | 0 - 3 | % | - | - | - | 0.227 |
|  | 4-7 | % | 42.041 | 0.000 | 3.187E+53 | 0.951 |
|  | 8-9 | % | 0.515 | 0.000 | 3.560E+51 | 0.991 |
|  | 10 - 11 | % | 997.373 | 0.000 | 7.934E+54 | 0.910 |
|  | ≥12 | % | 1679.577 | 0.000 | 1.364E+55 | 0.903 |
|  | **Site of Infection** |  |  |  |  |  |
|  | Urinary tract | % | 0.000 | 0.000 | 0.544 | 0.035 |
|  | **Completion of sepsis bundle** |  |  |  |  |  |
|  | Completion of the sepsis bundle within 1 hour | % | 0.014 | 0.000 | 4.484 | 0.147 |
|  | Completion of the administration of antibiotics within 1 hour | % | 0.011 | 0.000 | 3.977 | 0.133 |
|  | Completion of the sepsis bundle within 3 hours | % | 27.589 | 0.250 | 3045.993 | 0.167 |
|  | **Life-sustaining treatments during ICU stay** |  |  |  |  |  |
|  | Renal replacement therapy | % | 6754.838 | 1.216 | 37522700.047 | 0.045 |
|  | Platelet transfusion | % | 183.686 | 0.270 | 124797.596 | 0.117 |
|  | Fresh frozen plasma transfusion | % | 8.437 | 0.533 | 133.553 | 0.130 |
|  | Constant |  | 0.012 |  |  | 0.942 |
| 4 | **ICU characteristics** |  |  |  |  |  |
|  | Training programme in ICU |  |  |  |  |  |
|  | No | % | - | - | - | - |
|  | Yes | % | 0.003 | 0.000 | 0.763 | 0.040 |
|  | **Baseline characteristics** |  |  |  |  |  |
|  | Comorbidities |  |  |  |  |  |
|  | Cardiovascular disease | % | 6.015 | 0.587 | 61.689 | 0.131 |
|  | Chronic liver disease | % | 15.960 | 0.002 | 119333.120 | 0.543 |
|  | **Severity of illness scores** |  |  |  |  |  |
|  | SOFA |  |  |  |  |  |
|  | 0 - 3 | % | - | - | - | 0.168 |
|  | 4-7 | % | 39.027 | 0.000 | 1.269E+40 | 0.935 |
|  | 8-9 | % | .452 | 0.000 | 1.323E+38 | 0.986 |
|  | 10 - 11 | % | 560.861 | 0.000 | 1.886E+41 | 0.889 |
|  | ≥12 | % | 659.828 | 0.000 | 2.191E+41 | 0.886 |
|  | **Site of Infection** |  |  |  |  |  |
|  | Urinary tract | % | 0.000 | 0.000 | 0.395 | 0.026 |
|  | **Completion of sepsis bundle** |  |  |  |  |  |
|  | Completion of the sepsis bundle within 1 hour | % | 0.018 | 0.000 | 3.903 | 0.143 |
|  | Completion of the administration of antibiotics within 1 hour | % | 0.015 | 0.000 | 3.493 | 0.131 |
|  | Completion of the sepsis bundle within 3 hours | % | 28.436 | 0.323 | 2501.517 | 0.143 |
|  | **Life-sustaining treatments during ICU stay** |  |  |  |  |  |
|  | Renal replacement therapy | % | 3804.177 | 1.506 | 9611062.409 | 0.039 |
|  | Platelet transfusion | % | 89.612 | 0.353 | 22766.632 | 0.112 |
|  | Fresh frozen plasma transfusion | % | 8.100 | 0.547 | 119.892 | 0.128 |
|  | Constant |  | 0.016 |  |  | 0.927 |
| 5 | **ICU characteristics** |  |  |  |  |  |
|  | Training programme in ICU |  |  |  |  |  |
|  | No | % | - | - | - | - |
|  | Yes | % | 0.002 | 0.000 | 0.723 | 0.038 |
|  | **Baseline characteristics** |  |  |  |  |  |
|  | Comorbidities |  |  |  |  |  |
|  | Cardiovascular disease | % | 7.013 | 0.693 | 70.930 | 0.099 |
|  | **Severity of illness scores** |  |  |  |  |  |
|  | SOFA |  |  |  |  |  |
|  | 0 - 3 | % | - | - | - | 0.174 |
|  | 4-7 | % | 60.091 | 0.000 | 9.590E+46 | 0.939 |
|  | 8-9 | % | .435 | 0.000 | 6.402E+44 | 0.987 |
|  | 10 - 11 | % | 682.765 | 0.000 | 1.133E+48 | 0.902 |
|  | ≥12 | % | 833.554 | 0.000 | 1.368E+48 | 0.899 |
|  | **Site of Infection** |  |  |  |  |  |
|  | Urinary tract | % | 0.000 | 0.000 | 0.316 | 0.022 |
|  | **Completion of sepsis bundle** |  |  |  |  |  |
|  | Completion of the sepsis bundle within 1 hour | % | 0.023 | 0.000 | 4.686 | 0.164 |
|  | Completion of the administration of antibiotics within 1 hour | % | 0.014 | 0.000 | 3.369 | 0.127 |
|  | Completion of the sepsis bundle within 3 hours | % | 24.989 | 0.292 | 2141.642 | 0.156 |
|  | **Life-sustaining treatments during ICU stay** |  |  |  |  |  |
|  | Renal replacement therapy | % | 4866.983 | 1.587 | 14928102.705 | 0.038 |
|  | Platelet transfusion | % | 86.471 | 0.320 | 23395.314 | 0.119 |
|  | Fresh frozen plasma transfusion | % | 10.711 | 0.795 | 144.323 | 0.074 |
|  | Constant |  | 0.013 |  |  | 0.935 |
| 6 | **ICU characteristics** |  |  |  |  |  |
|  | Training programme in ICU |  |  |  |  |  |
|  | No | % | - | - | - | - |
|  | Yes | % | 0.083 | 0.009 | 0.759 | 0.028 |
|  | **Baseline characteristics** |  |  |  |  |  |
|  | Comorbidities |  |  |  |  |  |
|  | Cardiovascular disease | % | 2.451 | 0.477 | 12.590 | 0.283 |
|  | **Site of Infection** |  |  |  |  |  |
|  | Urinary tract | % | 0.039 | 0.003 | 0.529 | 0.015 |
|  | **Completion of sepsis bundle** |  |  |  |  |  |
|  | Completion of the sepsis bundle within 1 hour | % | 0.108 | 0.004 | 3.322 | 0.203 |
|  | Completion of the administration of antibiotics within 1 hour | % | 0.411 | 0.052 | 3.269 | 0.401 |
|  | Completion of the sepsis bundle within 3 hours | % | 15.515 | 0.866 | 277.933 | 0.063 |
|  | **Life-sustaining treatments during ICU stay** |  |  |  |  |  |
|  | Renal replacement therapy | % | 28.146 | 3.437 | 230.515 | 0.002 |
|  | Platelet transfusion | % | 7.365 | 0.985 | 55.065 | 0.052 |
|  | Fresh frozen plasma transfusion | % | 4.826 | 0.965 | 24.149 | 0.055 |
|  | Constant |  | 0.335 |  |  | 0.361 |
| 7 | **ICU characteristics** |  |  |  |  |  |
|  | Training programme in ICU |  |  |  |  |  |
|  | No | % | - | - | - | - |
|  | Yes | % | 0.080 | 0.008 | 0.770 | 0.029 |
|  | **Baseline characteristics** |  |  |  |  |  |
|  | Comorbidities |  |  |  |  |  |
|  | Cardiovascular disease | % | 2.420 | 0.469 | 12.494 | 0.291 |
|  | **Site of Infection** |  |  |  |  |  |
|  | Urinary tract | % | 0.034 | 0.003 | 0.448 | 0.010 |
|  | **Completion of sepsis bundle** |  |  |  |  |  |
|  | Completion of the sepsis bundle within 1 hour | % | 0.058 | 0.002 | 1.370 | 0.078 |
|  | Completion of the sepsis bundle within 3 hours | % | 23.678 | 1.472 | 380.798 | 0.026 |
|  | **Life-sustaining treatments during ICU stay** |  |  |  |  |  |
|  | Renal replacement therapy | % | 22.053 | 3.084 | 157.696 | 0.002 |
|  | Platelet transfusion | % | 6.376 | 0.900 | 45.187 | 0.064 |
|  | Fresh frozen plasma transfusion | % | 5.537 | 1.153 | 26.588 | 0.033 |
|  | Constant |  | 0.223 |  |  | 0.194 |
| 8 | **ICU characteristics** |  |  |  |  |  |
|  | Training programme in ICU |  |  |  |  |  |
|  | No | % | - | - | - | - |
|  | Yes | % | 0.069 | 0.008 | 0.597 | 0.015 |
|  | **Site of Infection** |  |  |  |  |  |
|  | Urinary tract | % | 0.030 | 0.002 | 0.378 | 0.007 |
|  | **Completion of sepsis bundle** |  |  |  |  |  |
|  | Completion of the sepsis bundle within 1 hour | % | 0.052 | 0.002 | 1.228 | 0.067 |
|  | Completion of the sepsis bundle within 3 hours | % | 31.895 | 1.975 | 515.061 | 0.015 |
|  | **Life-sustaining treatments during ICU stay** |  |  |  |  |  |
|  | Renal replacement therapy | % | 21.256 | 3.076 | 146.885 | 0.002 |
|  | Platelet transfusion | % | 6.434 | 0.900 | 46.000 | 0.064 |
|  | Fresh frozen plasma transfusion | % | 5.575 | 1.168 | 26.622 | 0.031 |
|  | Constant |  | 0.305 |  |  | 0.253 |
| 9 | **ICU characteristics** |  |  |  |  |  |
|  | Training programme in ICU |  |  |  |  |  |
|  | No | % | - | - | - | - |
|  | Yes | % | 0.018 | 0.001 | 0.399 | 0.011 |
|  | **Severity of illness scores** |  |  |  |  |  |
|  | SOFA |  |  |  |  |  |
|  | 0 - 3 | % | - | - | - | 0.050 |
|  | 4-7 | % | 15.932 | 0.000 | 2151791.550 | 0.646 |
|  | 8-9 | % | 1.832 | 0.000 | 167042.041 | 0.917 |
|  | 10 - 11 | % | 106.223 | 0.001 | 14246611.260 | 0.439 |
|  | ≥12 | % | 172.207 | 0.001 | 19823861.082 | 0.387 |
|  | **Site of Infection** |  |  |  |  |  |
|  | Urinary tract | % | 0.003 | 0.000 | 0.122 | 0.002 |
|  | **Completion of sepsis bundle** |  |  |  |  |  |
|  | Completion of the sepsis bundle within 1 hour | % | 0.024 | 0.000 | 1.750 | 0.088 |
|  | Completion of the sepsis bundle within 3 hours | % | 40.920 | 1.241 | 1349.055 | 0.037 |
|  | **Life-sustaining treatments during ICU stay** |  |  |  |  |  |
|  | Renal replacement therapy | % | 58.558 | 2.701 | 1269.534 | 0.010 |
|  | Platelet transfusion | % | 7.443 | 0.398 | 139.132 | 0.179 |
|  | Fresh frozen plasma transfusion | % | 8.665 | 0.911 | 82.429 | 0.060 |
|  | Constant |  | 0.016 |  |  | 0.480 |
| 10 | **ICU characteristics** |  |  |  |  |  |
|  | Training programme in ICU |  |  |  |  |  |
|  | No | % | - | - | - | - |
|  | Yes | % | 0.044 | 0.003 | 0.558 | 0.016 |
|  | **Severity of illness scores** |  |  |  |  |  |
|  | SOFA |  |  |  |  |  |
|  | 0 - 3 | % | - | - | - | 0.032 |
|  | 4-7 | % | 3.084 | 0.003 | 3052.897 | 0.749 |
|  | 8-9 | % | 0.643 | 0.001 | 441.057 | 0.894 |
|  | 10 - 11 | % | 24.717 | 0.025 | 24625.717 | 0.363 |
|  | ≥12 | % | 64.451 | 0.070 | 59369.234 | 0.232 |
|  | **Site of Infection** |  |  |  |  |  |
|  | Urinary tract | % | 0.007 | 0.000 | 0.177 | 0.003 |
|  | **Completion of sepsis bundle** |  |  |  |  |  |
|  | Completion of the sepsis bundle within 1 hour | % | 0.070 | 0.002 | 2.858 | 0.160 |
|  | Completion of the sepsis bundle within 3 hours | % | 18.815 | 0.967 | 365.964 | 0.053 |
|  | **Life-sustaining treatments during ICU stay** |  |  |  |  |  |
|  | Renal replacement therapy | % | 31.386 | 2.814 | 350.117 | 0.005 |
|  | Fresh frozen plasma transfusion | % | 9.257 | 1.025 | 83.615 | 0.047 |
|  | Constant |  | 0.059 |  |  | 0.379 |
| 11 | **ICU characteristics** |  |  |  |  |  |
|  | Training programme in ICU |  |  |  |  |  |
|  | No | % | - | - | - | - |
|  | Yes | % | 0.046 | 0.004 | 0.548 | 0.015 |
|  | **Severity of illness scores** |  |  |  |  |  |
|  | SOFA |  |  |  |  |  |
|  | 0 - 3 | % | - | - | - | 0.021 |
|  | 4-7 | % | 3.864 | 0.010 | 1443.832 | 0.655 |
|  | 8-9 | % | 0.799 | 0.003 | 207.310 | 0.937 |
|  | 10 - 11 | % | 20.841 | 0.057 | 7654.246 | 0.314 |
|  | ≥12 | % | 76.594 | 0.212 | 27694.813 | 0.149 |
|  | **Site of Infection** |  |  |  |  |  |
|  | Urinary tract | % | 0.006 | 0.000 | 0.152 | 0.002 |
|  | **Completion of sepsis bundle** |  |  |  |  |  |
|  | Completion of the sepsis bundle within 3 hours | % | 3.869 | 0.590 | 25.379 | 0.159 |
|  | **Life-sustaining treatments during ICU stay** |  |  |  |  |  |
|  | Renal replacement therapy | % | 14.252 | 2.210 | 91.914 | 0.005 |
|  | Fresh frozen plasma transfusion | % | 8.511 | 1.050 | 68.999 | 0.045 |
|  | Constant |  | 0.091 |  |  | 0.367 |
| 12 | **ICU characteristics** |  |  |  |  |  |
|  | Training programme in ICU |  |  |  |  |  |
|  | No | % | - | - | - | - |
|  | Yes | % | 0.049 | 0.004 | 0.540 | 0.014 |
|  | **Severity of illness scores** |  |  |  |  |  |
|  | SOFA |  |  |  |  |  |
|  | 0 - 3 | % | - | - | - | 0.021 |
|  | 4-7 | % | 6.896 | 0.019 | 2551.882 | 0.522 |
|  | 8-9 | % | .862 | 0.003 | 249.998 | 0.959 |
|  | 10 - 11 | % | 38.146 | 0.095 | 15273.108 | 0.234 |
|  | ≥12 | % | 91.697 | 0.226 | 37200.843 | 0.140 |
|  | **Site of Infection** |  |  |  |  |  |
|  | Urinary tract | % | 0.007 | 0.000 | 0.161 | 0.002 |
|  | **Life-sustaining treatments during ICU stay** |  |  |  |  |  |
|  | Renal replacement therapy | % | 14.414 | 2.426 | 85.656 | 0.003 |
|  | Fresh frozen plasma transfusion | % | 9.574 | 1.208 | 75.876 | 0.032 |
|  | Constant |  | 0.085 |  |  | 0.366 |

**Table S41**. Data on total number of patients during the study dates stratified by seasons

| Partacipating hospital | Collection 1 (9th January, 2019) | | | Collection 2 (3rd April, 2019) | | | Collection 3 (3rd July, 2019) | | | Collection 4 (9th October, 2019) | | |
| --- | --- | --- | --- | --- | --- | --- | --- | --- | --- | --- | --- | --- |
|  | Total number of admitted patients in hospital | Number of ICU patients | Number of ICU patients with sepsis | Total number of admitted patients in hospital | Number of ICU patients | Number of ICU patients with sepsis | Total number of admitted patients in hospital | Number of ICU patients | Number of ICU patients with sepsis | Total number of admitted patients in hospital | Number of ICU patients | Number of ICU patients with sepsis |
| 115 People's Hospital | 1890 | 33 | 6 | 1949 | 34 | 7 | 2257 | 35 | 8 | 1860 | 33 | 4 |
| Bach Mai Hospital | 5745 | 40 | 8 | 4752 | 69 | 5 | 4738 | 67 | 7 | 4576 | 58 | 6 |
| Bai Chay General Hospital | 693 | 26 | 4 | 828 | 21 | 4 | 900 | 20 | 4 | 792 | 20 | 2 |
| Can Tho Central General Hospital | Not available | Not available | 3 | Not available | Not available | 3 | Not available | Not available | 1 | Not available | Not available | 0 |
| Cho Ray Hospital | 2628 | 26 | 11 | 2792 | 25 | 11 | 2926 | 28 | 8 | 2898 | 26 | 11 |
| Da Nang Hospital | 2834 | 73 | 5 | 2765 | 56 | 3 | 2545 | 28 | 2 | 2450 | 12 | 2 |
| Dong Da General Hospital | 247 | 16 | 3 | 340 | 13 | 2 | 289 | 16 | 2 | 350 | 17 | 2 |
| Hanoi Medical University Hospital | 429 | 15 | 3 | 511 | 15 | 3 | 545 | 9 | 1 | 523 | 15 | 5 |
| Hue Central General Hospital | 3393 | 88 | 15 | 3141 | 83 | 11 | 3005 | 76 | 6 | 2967 | 64 | 7 |
| Saint Paul General Hospital | 885 | 34 | 2 | 745 | 39 | 3 | 683 | 34 | 2 | 548 | 23 | 2 |
| Thai Nguyen Central General Hospital | 1070 | 27 | 2 | Not available | Not available | 0 | Not available | Not available | 0 | Not available | Not available | 0 |
| Thanh Nhan General Hospital | 847 | 43 | 1 | Not available | Not available | 0 | Not available | Not available | 0 | Not available | Not available | 0 |
| Vietnam–Czechoslovakia Friendship Hospital | 1600 | 35 | 14 | 1990 | 42 | 10 | 1618 | 32 | 12 | 1709 | 41 | 12 |
| Vinmec Times City International Hospital | 163 | 7 | 3 | 213 | 8 | 0 | 184 | 5 | 1 | 191 | 11 | 3 |
| Total number of patients | 22424 | 463 | 77^a^ | 20026 | 405 | 59^b^ | 19690 | 327 | 53^b^ | 18864 | 320 | 56^b^ |
| ^a^ Did not include patients with sepsis who were admitted to the Can Tho Central General Hospital.  ^b^ Did not include patients with sepsis who were admitted to the Can Tho Central General, the Thai Nguyen Central General and the Thanh Nhan General Hospitals. | | | | | | | | | | | | |

**Table S42**. Hospital and intensive care unit characteristics according to study dates

| Characteristics | All patients  (n=252) | Collection 1 (9th January, 2019)  (n=80) | Collection 2 (3rd April, 2019)  (n=62) | Collection 3 (3rd July, 2019)  (n=54) | Collection 4 (9th October, 2019)  (n=56) | P^a^ |
| --- | --- | --- | --- | --- | --- | --- |
| **Hospital characteristics** |  |  |  |  |  |  |
| Type of hospital, no. (%) |  |  |  |  |  | - |
| Rural | 0 | 0 | 0 | 0 | 0 |  |
| Urban | 252 (100) | 80 (100) | 62 (100) | 54 (100) | 56 (100) |  |
| University affiliation, no. (%) |  |  |  |  |  | 0.869 |
| No | 153 (60.7) | 49 (61.2) | 37 (59.7) | 35 (64.8) | 32 (57.1) |  |
| Yes | 99 (39.3) | 31 (38.8) | 25 (40.3) | 19 (35.2) | 24 (42.9) |  |
| **ICU characteristics** |  |  |  |  |  |  |
| Nature of ICU, no. (%) |  |  |  |  |  | - |
| Open | 0 | 0 | 0 | 0 | 0 |  |
| Closed | 252 (100) | 80 (100) | 62 (100) | 54 (100) | 56 (100) |  |
| Type of ICU, no. (%) |  |  |  |  |  | 0.668 |
| Medical | 110 (43.7) | 33 (41.2) | 28 (45.2) | 27 (50.0) | 22 (39.2) |  |
| Surgical | 0 | 0 | 0 | 0 | 0 |  |
| Mixed | 142 (56.3) | 47 (58.8) | 34 (54.8) | 27 (50.0) | 34 (60.7) |  |
| Nurse to patient ratio, no. (%) |  |  |  |  |  | 0.190 |
| 1 or more nurses : 1 patient | 7 (2.8) | 3 (3.8) | 0 (0.0) | 1 (1.9) | 3 (5.4) |  |
| 1 nurse : 2 patients | 187 (74.2) | 54 (67.5) | 45 (72.6) | 44 (81.5) | 44 (78.6) |  |
| 1 nurse : 3 patients | 0 | 0 | 0 | 0 | 0 |  |
| 1 nurse : 4 or more patients | 58 (23.0) | 23 (28.8) | 17 (27.4) | 9 (16.7) | 9 (16.1) |  |
| Intensivist to patient ratio, no. (%) |  |  |  |  |  | 0.967 |
| 1 intensivist : 5 or fewer patients | 165 (65.5) | 49 (61.2) | 40 (64.5) | 37 (68.5) | 39 (69.6) |  |
| 1 intensivist : 6 to 8 patients | 75 (29.8) | 26 (32.5) | 19 (30.6) | 15 (27.8) | 15 (26.8) |  |
| 1 intensivist : 9 to 11 patients | 0 | 0 | 0 | 0 | 0 |  |
| 1 intensivist : 12 or more patients | 12 (4.8) | 5 (6.2) | 3 (4.8) | 2 (3.7) | 2 (3.6) |  |
| Training programme in ICU, no. (%) |  |  |  |  |  | 0.439 |
| No | 50 (19.8) | 14 (17.5) | 15 (24.2) | 13 (24.1) | 8 (14.3) |  |
| Yes | 202 (80.2) | 66 (82.5) | 47 (75.8) | 41 (75.9) | 48 (85.7) |  |
| ^a^ Comparison between collection 1 (9th January, 2019), collection 2 (3rd April, 2019), collection 3 (3rd July, 2019) and collection 4 (9th October, 2019).  Abbreviations: **ICU**, intensive care unit; **no.**, number. | | | | | | |

**Table S43**. Baseline characteristics of the study population according to study dates

| Characteristics | All patients  (n=252) | Collection 1 (9th January, 2019)  (n=80) | Collection 2 (3rd April, 2019)  (n=62) | Collection 3 (3rd July, 2019)  (n=54) | Collection 4 (9th October, 2019)  (n=56) | P^a^ |
| --- | --- | --- | --- | --- | --- | --- |
| Age (year), median (IQR) | 65 (52-76.75) | 65.5 (54-76.75) | 70 (55.5-77) | 60 (49-70) | 65.5 (48.25-81.75) | 0.078 |
| Age (year), no. (%) |  |  |  |  |  | 0.359 |
| < 20 | 3 (1.2) | 0 (0.0) | 1 (1.6) | 2 (3.7) | 0 (0.0) |  |
| 20 - 39 | 19 (7.5) | 4 (5.0) | 5 (8.1) | 5 (9.3) | 5 (8.9) |  |
| 40 - 59 | 74 (29.4) | 27 (33.8) | 13 (21.0) | 19 (35.2) | 15 (26.8) |  |
| ≥ 60 | 156 (61.9) | 49 (61.2) | 43 (69.4) | 28 (51.9) | 36 (64.3) |  |
| Sex (male), no. (%) | 162 (64.3) | 48 (60.0) | 40 (64.5) | 38 (70.4) | 36 (64.3) | 0.679 |
| Admission type, no. (%) |  |  |  |  |  | 0.360 |
| Medical | 236 (93.7) | 73 (91.2) | 60 (96.8) | 51 (94.4) | 52 (92.9) |  |
| Elective surgical | 2 (0.8) | 0 (0.0) | 1 (1.6) | 0 (0.0) | 1 (1.8) |  |
| Unscheduled surgical | 14 (5.6) | 7 (8.8) | 1 (1.6) | 3 (5.6) | 3 (5.4) |  |
| Admission source, no. (%) |  |  |  |  |  | 0.247 |
| Emergency department | 138 (54.8) | 52 (65.0) | 25 (40.3) | 32 (59.3) | 29 (51.8) |  |
| Operating room | 4 (1.6) | 2 (2.5) | 1 (1.6) | 0 (0.0) | 1 (1.8) |  |
| General wards | 56 (22.2) | 9 (11.2) | 22 (35.5) | 11 (20.4) | 14 (25.0) |  |
| Other ICUs or HDU | 16 (6.3) | 6 (7.5) | 3 (4.8) | 3 (5.6) | 4 (7.1) |  |
| Inter-hospital transfer | 37 (14.7) | 10 (12.5) | 11 (17.7) | 8 (14.8) | 8 (14.3) |  |
| Others | 1 (0.4) | 1 (1.2) | 0 (0.0) | 0 (0.0) | 0 (0.0) |  |
| Comorbidities, no. (%) |  |  |  |  |  |  |
| Cardiovascular disease | 78 (31.0) | 28 (35.0) | 27 (43.5) | 13 (24.1) | 10 (17.9) | 0.012 |
| Chronic lung disease | 30 (11.9) | 12 (15.0) | 8 (12.9) | 3 (5.6) | 7 (12.5) | 0.410 |
| Chronic neurological disease | 36 (14.3) | 12 (15.0) | 12 (19.4) | 7 (13.0) | 5 (8.9) | 0.436 |
| Chronic kidney disease | 23 (9.1) | 8 (10.0) | 3 (4.8) | 9 (16.7) | 3 (5.4) | 0.106 |
| Peptic ulcer disease | 9 (3.6) | 3 (3.8) | 3 (4.8) | 1 (1.9) | 2 (3.6) | 0.938 |
| Chronic liver disease | 27 (10.7) | 8 (10.0) | 4 (6.5) | 9 (16.7) | 6 (10.7) | 0.359 |
| Diabetes mellitus | 67 (26.6) | 24 (30.0) | 18 (29.0) | 13 (24.1) | 12 (21.4) | 0.658 |
| Connective tissue disease | 3 (1.2) | 0 (0.0) | 0 (0.0) | 1 (1.9) | 2 (3.6) | 0.127 |
| Immunosuppression | 10 (4.0) | 3 (3.8) | 4 (6.5) | 3 (5.6) | 0 (0.0) | 0.248 |
| Haematological malignancies | 5 (2.0) | 0 (0.0) | 2 (3.2) | 1 (1.9) | 2 (3.6) | 0.339 |
| Solid malignant tumours | 12 (4.8) | 2 (2.5) | 4 (6.5) | 0 (0.0) | 6 (10.7) | 0.030 |
| ^a^ Comparison between collection 1 (9th January, 2019), collection 2 (3rd April, 2019), collection 3 (3rd July, 2019) and collection 4 (9th October, 2019).  Abbreviations: HDU, high dependency unit; ICU, intensive care unit; IQR, interquartile range; no., number. | | | | | | |

**Table S44**. Clinical and laboratory characteristics and severity of illness according to according to study dates

| Characteristics | All patients  (n=252) | Collection 1 (9th January, 2019)  (n=80) | Collection 2 (3rd April, 2019)  (n=62) | Collection 3 (3rd July, 2019)  (n=54) | Collection 4 (9th October, 2019)  (n=56) | P^a^ |
| --- | --- | --- | --- | --- | --- | --- |
| **Vital signs** (Upon admission into ICU) |  |  |  |  |  |  |
| GCS, median (IQR) | 13 (9-15) | 14 (9-15) | 12 (10-15) | 12 (8-15) | 13 (10-15) | 0.220 |
| HR (beats per min), median (IQR) | 110 (95.25-125.75) | 109 (95.25) | 116 (98.75-130.5) | 110 (96.75-126) | 110 (92-120.75) | 0.272 |
| Temperature (oC), mean (SD) | 37.79 (1.01) | 37.55 (1.00) | 37.84 (0.96) | 37.84 (1.02) | 38.01 (1.05) | 0.031 |
| MBP (mmHg), mean(SD) | 75.82 (22.08) | 72.65 (17.19) | 75.57 (21.99) | 79.49 (26.86) | 77.07 (23.24) | 0.484 |
| SBP (mmHg), mean (SD) | 106.45 (29.96) | 102.7 (26.78) | 107.42 (30.67) | 109.24 (36.58) | 108.05 (23.50) | 0.538 |
| RR (breaths per min), median (IQR) | 25 (22-30) | 24.5 (20.25-28) | 24 (21.5-28) | 26 (22-30) | 28 (24-30) | 0.002 |
| **Blood investigations** | | | | | | |
| Total WBC (x10^9^/L), mean (SD) | 15.73 (9.20) | 18.59 (10.19) | 14.82 (7.98) | 14.40 (9.71) | 13.93 (7.58) | 0.032 |
| PLT (x10^9^/L), mean (SD) | 185.98 (137.85) | 198.05(124.51) | 188.36 (148.84) | 180.54 (157.77) | 171.34 (124.19) | 0.353 |
| Hb (g/dL), mean (SD) | 11.14 (2.59) | 11.34 (3.03) | 11.02 (2.36) | 10.81 (2.69) | 11.32 (2.04) | 0.606 |
| Hct (%), mean (SD) | 34.31 (7.75) | 35.03 (8.90) | 34.31 (6.82) | 33.41 (8.40) | 34.16 (6.25) | 0.713 |
| K^+^ (mmol/L), mean (SD) | 3.89 (0.79) | 3.68 (0.84) | 3.95 (0.82) | 3.84 (0.78) | 3.87 (0.70) | 0.906 |
| Na^+^ (mmol/L), mean (SD) | 136.05 (8.24) | 136.74 (9.90) | 135.95 (7.80) | 136.11 (6.41) | 135.13 (7.77) | 0.814 |
| Creatinine (µmol/L), mean (SD) | 187.85 (151.92) | 178.87 (132.80) | 177.20 (126.97) | 236.49 (216.70) | 165.56 (117.46) | 0.493 |
| Bilirubin (µmol/l), mean (SD) | 32.80 (61.49) | 37.51 (67.34) | 24.14 (27.91) | 37.91 (92.75) | 31.03 (39.12) | 0.442 |
| pH, mean (SD) | 7.37 (0.50) | 7.34 (0.12) | 7.48 (0.99) | 7.32 (0.15) | 7.36 (0.10) | 0.696 |
| PaO_2_ (mmHg), mean (SD) | 116.17 (74.28) | 106.78 (59.35) | 126.86 (102.93) | 118.37 (67.88) | 115.69 (61.54) | 0.780 |
| FiO_2_, mean (SD) | 0.50 (0.22) | 0.48 (0.22) | 0.51 (0.24) | 0.53 (0.25) | 0.47 (0.14) | 0.566 |
| PaO_2_/FiO_2_ ratio, mean (SD) | 262.48 (149.58) | 246.99 (116.50) | 271.91 (181.24) | 275.05 (167.10) | 261.67 (136.28) | 0.949 |
| **Severity of illness scores** | | | | | | |
| qSOFA, median (IQR) | 2 (1-2) | 2 (1-2) | 2 (1-2.25) | 2 (2-3) | 2 ((1.25-3) | 0.328 |
| qSOFA, no. (%) |  |  |  |  |  | 0.586 |
| 0 - 1 | 69 (27.4) | 26 (32.5) | 17 (27.4) | 12 (22.2) | 14 (25.0) |  |
| 2 - 3 | 183 (72.6) | 54 (67.5) | 45 (72.6) | 42 (77.8) | 42 (75.0) |  |
| SIRS, median (IQR) | 3 (2-4) | 3 (2-3) | 3 (2-3.25) | 3 (2-3.25) | 3 (3-4) | 0.136 |
| SOFA, median (IQR) | 7 (4.75-10) | 7 (5-10) | 6 (3-10) | 8 (6-11) | 7 (4.25-9) | 0.283 |
| SOFA, no. (%) |  |  |  |  |  | 0.057 |
| 0 - 1 | 0 | 0 | 0 | 0 | 0 |  |
| 2 - 3 | 46 (18.4) | 16 (20.3) | 16 (26.2) | 6 (11.1) | 8 (14.3) |  |
| 4 - 5 | 36 (14.4) | 14 (17.7) | 9 (14.8) | 5 (9.3) | 8 (14.3) |  |
| 6 - 7 | 58 (23.2) | 18 (22.8) | 11 (18.0) | 14 (25.9) | 15 (26.8) |  |
| 8 - 9 | 32 (12.8) | 10 (12.7) | 2 (3.3) | 7 (13.0) | 13 (23.2) |  |
| 10 - 11 | 38 (15.2) | 8 (10.1) | 10 (16.4) | 13 (24.1) | 7 (12.5) |  |
| 12 - 14 | 29 (11.6) | 11 (13.9) | 10 (16.4) | 4 (7.4) | 4 (7.1) |  |
| > 14 | 11 (4.4) | 2 (2.5) | 3 (4.9) | 5 (9.3) | 1 (1.8) |  |
| APACHE II, median (IQR) | 18 (13-24) | 16.5 (12-23) | 19 (14-24) | 19.5 (12.75-26) | 19.5 (14-23) | 0.407 |
| APACHE II, no. (%) |  |  |  |  |  | 0.092 |
| 0 - 4 | 3 (1.2) | 3 (3.8) | 0 | 0 | 0 |  |
| 5 - 9 | 22 (8.7) | 6 (7.5) | 6 (9.7) | 6 (11.1) | 4 (7.1) |  |
| 10 - 14 | 61 (24.2) | 26 (32.5) | 12 (19.4) | 12 (22.2) | 11 (19.6) |  |
| 15 - 19 | 52 (20.6) | 15 (18.8) | 15 (24.2) | 9 (16.7) | 13 (23.2) |  |
| 20 - 24 | 58 (23.0) | 14 (17.5) | 15 (24.2) | 11 (20.4) | 18 (32.1) |  |
| 25 - 29 | 28 (11.1) | 9 (11.3) | 5 (8.1) | 10 (18.5) | 4 (7.1) |  |
| 30 - 34 | 19 (7.5) | 6 (7.5) | 3 (4.8) | 4 (7.4) | 6 (10.7) |  |
| > 34 | 9 (3.6) | 1 (1.3) | 6 (9.7) | 2 (3.7) | 0 |  |
| Septic shock (SS) | 74 (29.4) | 24 (30.0) | 22 (35.5) | 16 (29.6) | 12 (21.4) | 0.417 |
| ^a^ Comparison between collection 1 (9th January, 2019), collection 2 (3rd April, 2019), collection 3 (3rd July, 2019) and collection 4 (9th October, 2019).  Abbreviations: **APACHE II**, acute physiologic assessment and chronic health evaluation II; **FiO_2_**, fraction of inspired oxygen; **GCS**, Glasgow coma scale; **Hb**, hemoglobin; **Hct**, hematocrit; **HDU**, high dependency unit; **ICU**, intensive care unit; **IQR**, interquartile range; **MBP**, mean blood pressure; **no.**, number; **PaO_2_**, partial pressure of oxygen; **PLT**, platelet; **qSOFA**, quick sequential organ failure assessment; **RR**, respiratory rate; **SBP**, systolic blood pressure; **SD**, standard deviation; **SIRS**, systemic inflammatory response syndrome; **SOFA**, sequential organ failure assessment; **WBC**, white blood cell. | | | | | | |

**Table S45**. Sites of infection and microbiology according to according to study dates

| Characteristics | All patients  (n=252) | Collection 1 (9th January, 2019)  (n=80) | Collection 2 (3rd April, 2019)  (n=62) | Collection 3 (3rd July, 2019)  (n=54) | Collection 4 (9th October, 2019)  (n=56) | P |
| --- | --- | --- | --- | --- | --- | --- |
| **Site of infection** |  |  |  |  |  |  |
| Respiratory, no. (%) | 143 (56.7) | 46 (57.5) | 34 (54.8) | 31 (57.4) | 32 (57.1) | 0.989 |
| Urinary tract, no. (%) | 37 (14.7) | 13 (16.3) | 11 (17.7) | 7 (13.0) | 6 (10.7) | 0.693 |
| Abdominal, no. (%) | 61 (24.2) | 22 (27.5) | 15 (24.2) | 14 (25.9) | 10 (17.9) | 0.617 |
| Neurological, no. (%) | 12 (4.8) | 3 (3.8) | 1 (1.6) | 5 (9.3) | 3 (5.4) | 0.290 |
| Bones or joints, no. (%) | 2 (0.8) | 0 | 0 | 0 | 2 (3.6) | 0.094 |
| Skin or cutaneous sites, no. (%) | 19 (7.5) | 4 (5.0) | 5 (8.1) | 5 (9.3) | 5 (8.9) | 0.723 |
| Intravascular catheter, no. (%) | 1 (0.4) | 1 (1.3) | 0 | 0 | 0 | >0.999 |
| Infective endocarditis, no. (%) | 1 (0.4) | 0 | 1 (1.6) | 0 | 0 | 0.683 |
| Primary bacteraemia, no. (%) | 7 (2.8) | 1 (1.3) | 3 (4.8) | 1 (1.9) | 2 (3.6) | 0.638 |
| Systemic, no. (%) | 6 (2.4) | 3 (3.8) | 1 (1.6) | 2 (3.7) | 0 | 0.521 |
| **Microbiology** |  |  |  |  |  |  |
| No pathogens detected, no. (%) | 67 (26.6) | 26 (32.5) | 14 (22.6) | 13 (24.1) | 14 (25.0) | 0.534 |
| Gram negative bacteria, no. (%) | 156 (61.9) | 48 (60.0) | 37 (59.7) | 33 (61.1) | 38 (37.9) | 0.775 |
| *Klebsiella pneumonia* | 27 (10.7) | 2 (2.5) | 7 (11.3) | 9 (16.7) | 9 (16.1) | 0.025 |
| *Acinetobacter baumannii* | 45 (17.9) | 15 (18.8) | 11(17.7) | 9 (16.7) | 10 (17.9) | 0.992 |
| *Escherichia coli* | 44 (17.5) | 15 (18.8) | 11 (17.7) | 9 (16.7) | 9 (16.1) | 0.979 |
| *Pseudomonas aeruginosa* | 24 (9.5) | 9 (11.3) | 5 (8.1) | 5 (9.3) | 5 (8.9) | 0.928 |
| *Stenotrophomonas maltophilia* | 2 (0.8) | 0 | 0 | 1 (1.9) | 1 (1.8) | 0.249 |
| *Proteus* species | 47 (18.7) | 15 (18.8) | 12 (19.4) | 12 (22.2) | 8 (14.3) | 0.758 |
| *Enterobacter cloacae* | 3 (1.2) | 1 (1.3) | 2 (3.2) | 0 | 0 | 0.424 |
| *Bulkholderia pseudomallei* | 1 (0.4) | 0 | 1 (1.6) | 0 | 0 | 0.683 |
| Others | 0 | 0 | 0 | 0 | 0 | - |
| Gram positive bacteria, no. (%) | 34 (13.5) | 6 (7.5) | 11 (17.7) | 8 (14.8) | 9 (16.1) | 0.282 |
| *Enterococcus* | 5 (2.0) | 3 (3.8) | 0 | 1 (1.9) | 1 (1.8) | 0.487 |
| MSSA | 5 (2.0) | 1 (1.3) | 1 (1.6) | 1 (1.9) | 2 (3.6) | 0.871 |
| MRSA | 10 (4.0) | 1 (1.3) | 4 (6.5) | 2 (3.7) | 3 (5.4) | 0.404 |
| Other *Streptococcus* species | 12 (4.8) | 1 (1.3) | 5 (8.1) | 4 (7.4) | 2 (3.6) | 0.168 |
| *Streptococcus pneumonia* | 2 (0.8) | 0 | 1 (1.6) | 0 | 1 (1.8) | 0.565 |
| Fungi, no. (%) | 7 (2.8) | 3 (3.8) | 2 (3.2) | 2 (3.7) | 0 | 0.556 |
| *Candida* species | 7 (2.8) | 3 (3.8) | 2 (3.2) | 2 (3.7) | 0 | 0.556 |
| *Aspergillus* species | 0 | 0 | 0 | 0 | 0 | - |
| Others | 0 | 0 | 0 | 0 | 0 | - |
| Viruses, no. (%) | 2 (0.8) | 0 | 0 | 2 (3.7) | 0 | 0.045 |
| Influenza | 1 (0.4) | 0 | 0 | 1 (1.9) | 0 | 0.214 |
| Others | 0 | 0 | 0 | 0 | 0 | - |
| Dengue | 1 (0.4) | 0 | 0 | 1 (1.9) | 0 | 0.214 |
| Other pathogens, no. (%) |  |  |  |  |  |  |
| Anaerobes | 0 | 0 | 0 | 0 | 0 | - |
| Mycobacterium tuberculosis | 4 (1.6) | 3 (3.8) | 1 (1.6) | 0 | 0 | 0.345 |
| Malaria | 0 | 0 | 0 | 0 | 0 | - |
| ^a^ Comparison between collection 1 (9th January, 2019), collection 2 (3rd April, 2019), collection 3 (3rd July, 2019) and collection 4 (9th October, 2019).  Abbreviations: **MRSA**, methicillin-resistant Staphylococcus aureus; **MSSA**, methicillin-susceptible Staphylococcus aureus; **no.**, number. | | | | | | |

**Table S46. Breakdown of missing data**

| Variables | Number of patients with missing data |
| --- | --- |
| **Hospital characteristics** |  |
| Type of hospital | 0 |
| University affiliation | 0 |
| **ICU characteristics** |  |
| Nature of ICU | 0 |
| Type of ICU | 0 |
| Nurse to patient ratio | 0 |
| Intensivist to patient ratio | 0 |
| Training programme in ICU | 0 |
| **Baseline characteristics** |  |
| Age (year) | 0 |
| Sex | 0 |
| Collection batch | 0 |
| Admission type | 0 |
| Admission source | 0 |
| Comorbidities | 0 |
| **Vital signs** (on admission into ICU) |  |
| GCS | 1 |
| HR (beats per min) | 0 |
| Temperature (^o^C) | 0 |
| MBP (mmHg), mean(SD) | 0 |
| SBP (mmHg), mean (SD) | 0 |
| RR (breaths per min), median (IQR) | 0 |
| **Blood investigations** |  |
| Total WBC (x10^9^/L) | 0 |
| PLT (x10^9^/L) | 0 |
| Hb (g/dL) | 1 |
| Hct (%) | 0 |
| K^+^ (mmol/L) | 0 |
| Na^+^ (mmol/L) | 0 |
| Creatinine (µmol/L) | 0 |
| Bilirubin (µmol/l) | 20 |
| pH, mean (SD) | 3 |
| PaO_2_ (mmHg) | 8 |
| FiO_2_ (mmHg) | 7 |
| PaO_2_/FiO_2_ ratio | 9 |
| **Severity of illness scores** |  |
| qSOFA | 0 |
| SIRS | 0 |
| SOFA | 2 |
| APACHE II | 0 |
| **Site of Infection** |  |
| Respiratory | 0 |
| Urinary tract | 0 |
| Abdominal | 0 |
| Neurological | 0 |
| Bones or joints | 0 |
| Skin or cutaneous sites | 0 |
| Intravascular catheter | 0 |
| Infective endocarditis | 0 |
| Primary bacteraemia | 0 |
| Systemic | 0 |
| **Measurements around time zero** |  |
| Blood culture | 0 |
| Lactate measurement | 0 |
| Antibiotic administration | 0 |
| Fluid bolus | 2 |
| **Resources used in ICU** (anytime during ICU stay) |  |
| Vasopressors/Intropes | 0 |
| Mechanical ventilation | 1 |
| Noninvasive ventilation | 1 |
| High-flow nasal cannula | 1 |
| Renal replacement therapy | 1 |
| Red blood cell transfusion | 1 |
| Platelet transfusion | 1 |
| Fresh frozen plasma transfusion | 1 |
| Surgical source control | 1 |
| Non-surgical source control | 1 |
| **In-hospital time course** (DD/MM/YY (HHMM)) |  |
| Admission date to the hospital | 0 |
| Admission date to the ICU | 0 |
| Time zero | 0 |
| Time of blood culture | 2 |
| Time of lactate measurement | 1 |
| Time of antibiotic administration | 2 |
| Time of starting vasopressor | 3 |
| Time of first source control measure | 0 |
| Discharge date from current ICU stay or death date in your current ICU stay | 1 |
| Discharge date from current hospital stay or death date in your current hospital stay | 1 |
